# A chromosome-level genome assembly of *Artocarpus nanchuanensis* (Moraceae), an extremely endangered fruit tree

**DOI:** 10.1093/gigascience/giac042

**Published:** 2022-06-14

**Authors:** Jiaoyu He, Shanfei Bao, Junhang Deng, Qiufu Li, Shiyu Ma, Yiran Liu, Yanru Cui, Yuqi Zhu, Xia Wei, Xianping Ding, Kehui Ke, Chaojie Chen

**Affiliations:** Key Laboratory of Bio-Resources and Eco-Environment of Ministry of Education, College of Life Sciences, Sichuan University, Chengdu 610065, Sichuan, P.R. China; Chongqing Jinfo Shan Advanced Research Institute, Chongqing 408400, P.R. China; Bio-resource Research and Utilization Joint Key Laboratory of Sichuan and Chongqing, Sichuan and Chongqing 408400, P.R. China; Key Laboratory of Bio-Resources and Eco-Environment of Ministry of Education, College of Life Sciences, Sichuan University, Chengdu 610065, Sichuan, P.R. China; Chongqing Jinfo Shan Advanced Research Institute, Chongqing 408400, P.R. China; Bio-resource Research and Utilization Joint Key Laboratory of Sichuan and Chongqing, Sichuan and Chongqing 408400, P.R. China; Key Laboratory of Bio-Resources and Eco-Environment of Ministry of Education, College of Life Sciences, Sichuan University, Chengdu 610065, Sichuan, P.R. China; Chongqing Jinfo Shan Advanced Research Institute, Chongqing 408400, P.R. China; Bio-resource Research and Utilization Joint Key Laboratory of Sichuan and Chongqing, Sichuan and Chongqing 408400, P.R. China; Key Laboratory of Bio-Resources and Eco-Environment of Ministry of Education, College of Life Sciences, Sichuan University, Chengdu 610065, Sichuan, P.R. China; Chongqing Jinfo Shan Advanced Research Institute, Chongqing 408400, P.R. China; Bio-resource Research and Utilization Joint Key Laboratory of Sichuan and Chongqing, Sichuan and Chongqing 408400, P.R. China; Key Laboratory of Bio-Resources and Eco-Environment of Ministry of Education, College of Life Sciences, Sichuan University, Chengdu 610065, Sichuan, P.R. China; Chongqing Jinfo Shan Advanced Research Institute, Chongqing 408400, P.R. China; Bio-resource Research and Utilization Joint Key Laboratory of Sichuan and Chongqing, Sichuan and Chongqing 408400, P.R. China; Key Laboratory of Bio-Resources and Eco-Environment of Ministry of Education, College of Life Sciences, Sichuan University, Chengdu 610065, Sichuan, P.R. China; Chongqing Jinfo Shan Advanced Research Institute, Chongqing 408400, P.R. China; Bio-resource Research and Utilization Joint Key Laboratory of Sichuan and Chongqing, Sichuan and Chongqing 408400, P.R. China; Key Laboratory of Bio-Resources and Eco-Environment of Ministry of Education, College of Life Sciences, Sichuan University, Chengdu 610065, Sichuan, P.R. China; Chongqing Jinfo Shan Advanced Research Institute, Chongqing 408400, P.R. China; Bio-resource Research and Utilization Joint Key Laboratory of Sichuan and Chongqing, Sichuan and Chongqing 408400, P.R. China; Key Laboratory of Bio-Resources and Eco-Environment of Ministry of Education, College of Life Sciences, Sichuan University, Chengdu 610065, Sichuan, P.R. China; Chongqing Jinfo Shan Advanced Research Institute, Chongqing 408400, P.R. China; Bio-resource Research and Utilization Joint Key Laboratory of Sichuan and Chongqing, Sichuan and Chongqing 408400, P.R. China; Wood Comprehensive Factory of Chengdu, Sichuan 610081, P.R. China; Key Laboratory of Bio-Resources and Eco-Environment of Ministry of Education, College of Life Sciences, Sichuan University, Chengdu 610065, Sichuan, P.R. China; Chongqing Jinfo Shan Advanced Research Institute, Chongqing 408400, P.R. China; Bio-resource Research and Utilization Joint Key Laboratory of Sichuan and Chongqing, Sichuan and Chongqing 408400, P.R. China; Key Laboratory of Bio-Resources and Eco-Environment of Ministry of Education, College of Life Sciences, Sichuan University, Chengdu 610065, Sichuan, P.R. China; Chongqing Jinfo Shan Advanced Research Institute, Chongqing 408400, P.R. China; Bio-resource Research and Utilization Joint Key Laboratory of Sichuan and Chongqing, Sichuan and Chongqing 408400, P.R. China; Biomarker Technologies Corporation, Beijing 101300, China; Biomarker Technologies Corporation, Beijing 101300, China

**Keywords:** A. nanchuanensis, sequencing, Illumina, Nanopore, Hi-C, genome assembly, gene annotation, gene family

## Abstract

*Artocarpus nanchuanensis* (Moraceae), which is naturally distributed in China, is a representative and extremely endangered tree species. In this study, we obtained a high-quality chromosome-scale genome assembly and annotation information for *A. nanchuanensis* using integrated approaches, including Illumina, Nanopore sequencing platform, and Hi-C. A total of 128.71 Gb of raw Nanopore reads were generated from 20-kb libraries, and 123.38 Gb of clean reads were obtained after filtration with 160.34× coverage depth and a 17.48-kb average read length. The final assembled *A. nanchuanensis* genome was 769.44 Mb with a 2.09 Mb contig N50, and 99.62% (766.50 Mb) of the assembled data was assigned to 28 pseudochromosomes.

In total, 39,596 genes (95.10%, 39,596/41,636) were successfully annotated, and 129 metabolic pathways were detected. Plants disease resistance/insect resistance genes, plant–pathogen interaction metabolic pathways, and abundant biosynthesis pathways of vitamins, flavonoid, and gingerol were detected. Unigene reveals the basis of species-specific functions, and gene family in contraction and expansion generally implies strong functional differences in the evolution. Compared with other related species, a total of 512 unigenes, 309 gene families in contraction, and 559 gene families in expansion were detected in *A. nanchuanensis*.

This *A. nanchuanensis* genome information provides an important resource to expand our understanding of the unique biological processes, nutritional and medicinal benefits, and evolutionary relationship of this species. The study of gene function and metabolic pathway in *A. nanchuanensis* may reveal the theoretical basis of a special trait in *A. nanchuanensis* and promote the study and utilization of its rare medicinal value.

## Introduction

*Artocarpus nanchuanensis* (NCBI:txid1745975), which is mainly distributed in Chongqing Nanchuan, is part of a new generation of southern urban greening tree species; this species has high quality and excellent fast-growing characteristics, which allow it to live in acidic soil and environments with heavy atmospheric pollution due to it strong ability to resist pollution and disease [1, 2]. The fruit of *A. nanchuanensis* contains a variety of polysaccharides, amino acids, trace elements, and vitamins, which have a good control effect on constipation and other intestinal diseases [2]. The fruit and bark have been used in the treatment of skin diseases in Chongqing Nanchuan for a long time. These features have attracted the attention of researchers [1] and promoted the steady progress of relevant research. As research has developed, high-quality genome data are needed for this valuable species to promote studies of the molecular mechanisms related to its nutritional and medicinal value, as well as those of individual genome structure, genome evolution, and species diversity.

In the draft genome sequence of the mulberry tree *Morus notabilis*, 78.34 Gb of high-quality data were obtained and assembled into a 330.79-Mb mulberry genome with a 390,115-bp scaffold N50 and 34,476-bp contig N50 [3]. The assembled genome of *Broussonetia papyrifera* was 386.83 Mb with a 29.48-Mb scaffold N50 and 171.17-kb contig N50 [4]. The genome data analysis of *M. notabilis* and *B. papyrifera* provides a theoretical basis for the study of fiber development, lignin and flavonoid metabolism, nitrogen metabolism, important metal tolerance functions, and stress resistance evolution, but the genomic details of *A. nanchuanensis* remain unknown.

**Figure 1:**
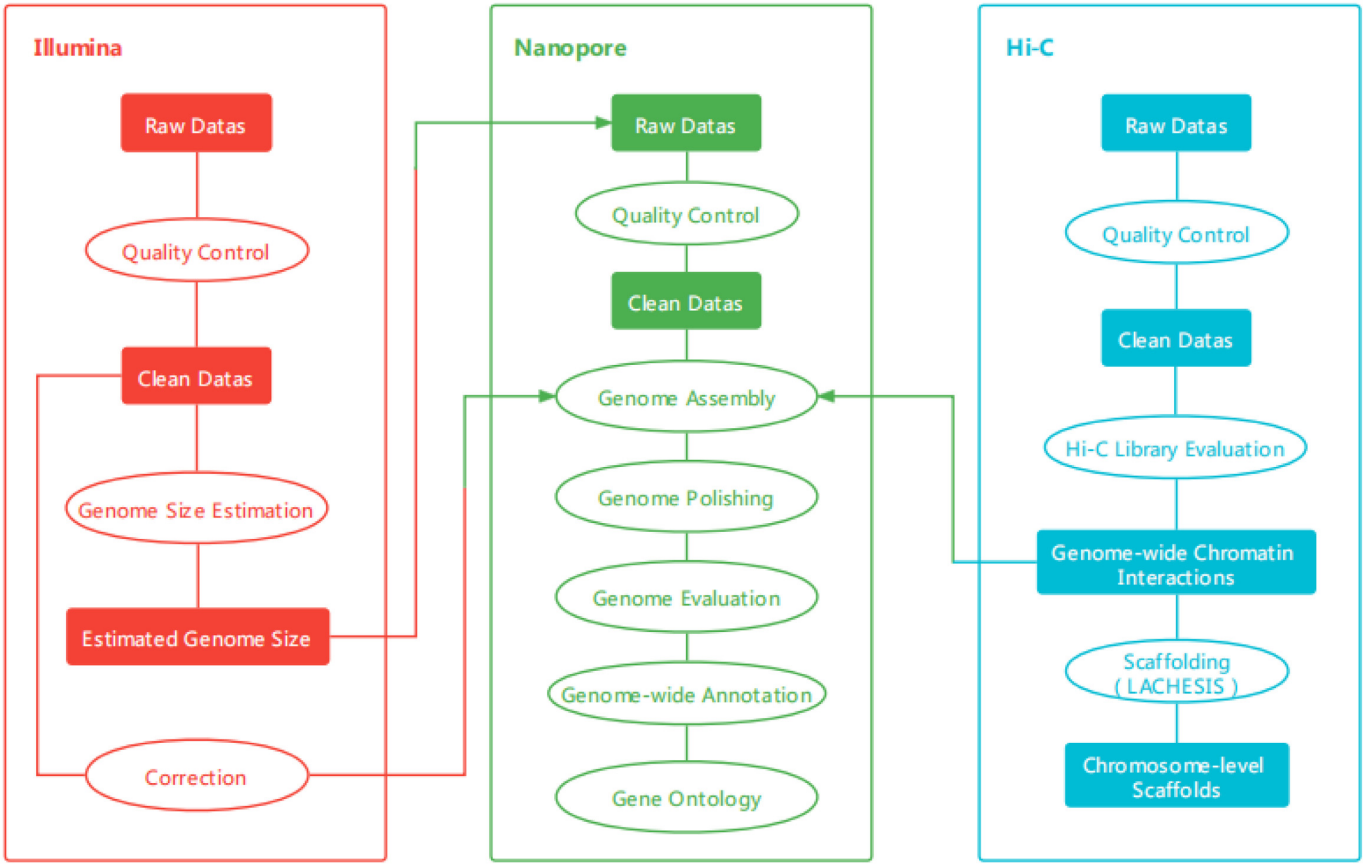
The flowchart of *A. nanchuanensis* genome assembly and annotation process.

To protect this species and make full use of its rare value, we applied a combined strategy involving Illumina sequencing, Nanopore single-molecule sequencing, and high-throughput/resolution chromosome conformation capture (Hi-C) technologies to generate sequencing data for the chromosomal genome construction and annotation of *A. nanchuanensis* [5–8] (Fig. 1). These genomic data provide not only the necessary resources for the determination of genome size but also convenience for research on reproduction and species evolution based on speciation and the local environment, which is beneficial to studies on the medicinal and economically valuable traits.

## Materials and methods

### Samples and DNA, RNA extraction

**Figure 2:**
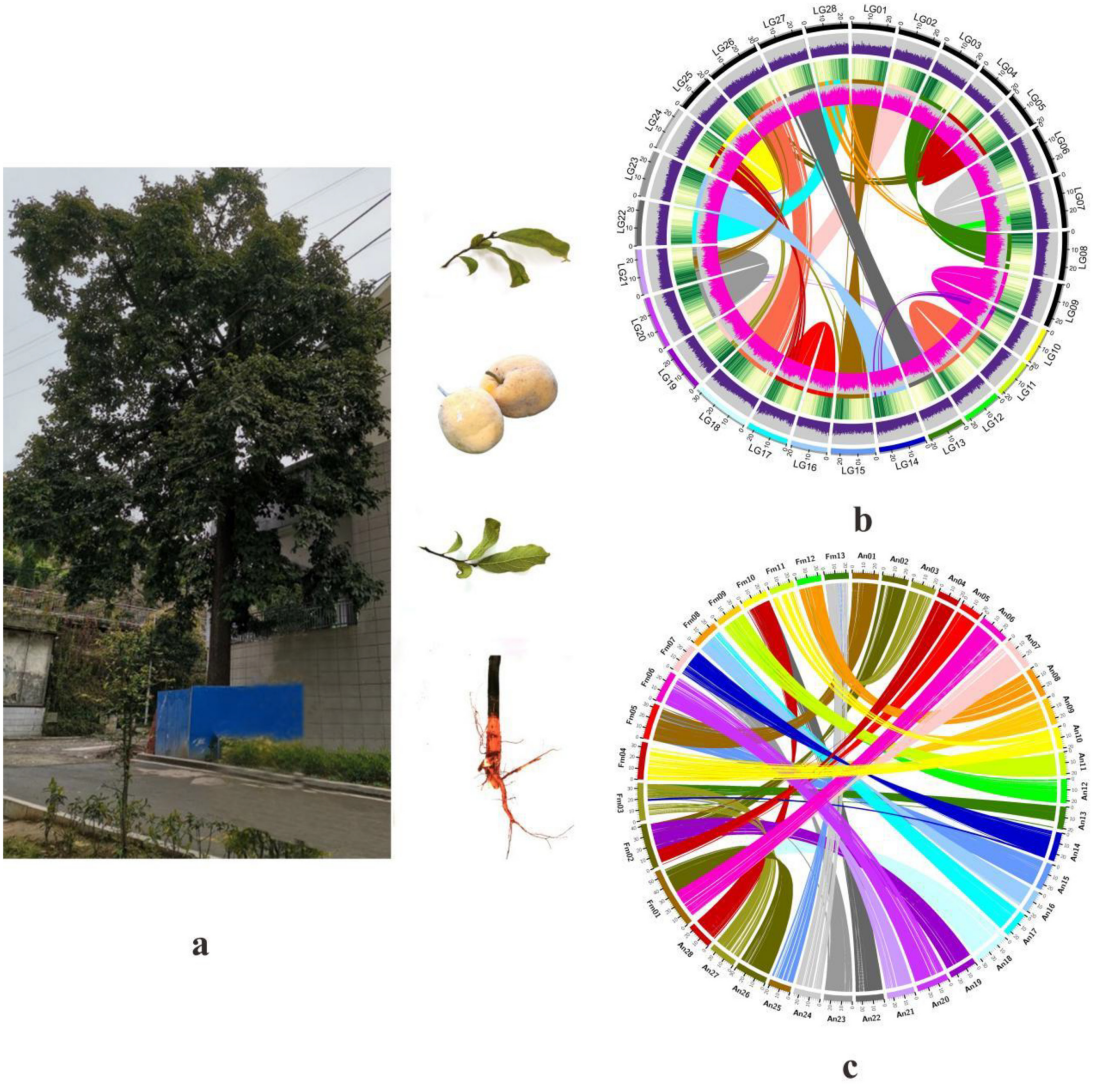
The *A. nanchuanensis* sample and genomic interaction analysis.

The oldest *A. nanchuanensis* tree surviving in Nanchuan district was selected as the sampling source (Fig. 2). Its fruits, young leaves, and roots were preserved in liquid nitrogen until DNA, RNA extraction.

For genome sequencing, DNA was extracted from 100-mg young leaves by the Cetyltrimethylammonium Bromide (CTAB) method [9]. The concentration and purity of the extracted DNA from the sample were detected by NanoDrop and Qubit, the integrity of the DNA was checked on pulsed field electrophoresis [10], and the extracted high-quality DNA was prepared for subsequent sequencing [10].

The leaves, fruits, and roots in the same growth stages were uniformly mixed, and a 100-mg mixture was used for RNA extraction by the Polysaccharides & Polyphenolics-rich RNAprep Pure Plant Kit (Tiangen, Beijing, P.R. China). The quality and concentration of the RNA were detected by Nanodrop. High-quality messenger RNA (mRNA) was purified by mRNA capture beads, and first-strand synthesis reaction buffer, random primers, and reverse transcription reagents were added to purified mRNA for mRNA fragmentation and complementary DNA synthesis. The synthesized and purified complementary DNA was incubated with end-repair reaction buffer and end-repair enzyme mix for end-repair and 3'-end A addition in the PCR instrument. The joint, ligase, and Uracil-Specific Excision Reagent (USER) enzymes were added to the reaction products for joint connection and joint opening, and magnetic beads were used for fragment selection. Finally, the selected fragments were amplified by PCR, and the products were purified for sequencing.

### Library construction and high-throughput sequencing

An ONT library with a 20-kb fragment length was constructed following the manufacturer's protocol. The large segments of the extracted DNA were filtered by the BluePippin™ System, and the large segments of DNA, ONT Template Preparation Kit (SQK-LSK109), Nanopore, Beijing, P.R. China and NEB Next FFPE DNA Repair Mix Kit, NEB, Shanghai, P.R. China were used to prepare a library. The high-quality library was sequenced on the ONT PromethION Beta platform (PromethION, RRID:SCR_017987) with a corresponding R9 flow cell and ONT sequencing reagent kit (EXP-FLP001.PRO.6).

An Illumina sequencing library was prepared for genome size estimation, genome assembly correction, and evaluation. The paired-end (PE) library with a 350-bp insertion size was prepared for the Illumina platform according to the manufacturers’ protocols (Illumina, San Diego, CA, USA) and subjected to PE (2 × 150 bp) sequencing on an Illumina NovaSeq 6000 sequencing platform (Illumina; RRID:SCR_016387)). For RNA, the joint and low-quality bases were filtered out with the fastp parameters (-q 10 -u 50 -y -g -Y 10 -e 20 -l 100 -b 150 -B 150 2), and the ribosomal RNA (rRNA) was filtered by soap (parameters: soap -a 1.fq -b 2.fq -D/share/nas2/database/sRNA_database/current/ncRNA_integer.fasta. index -o out.pe -2 out.se -m 100 -x 1000 -u unmap.fa). For DNA, the joint and low-quality bases were filtered out with the fastp parameters (-q 10 -u 50 -y -g -Y 10 -e 20 -l 100 -b 150 -B 150). The filtered clean reads were used for subsequent analysis.

Hi-C fragment libraries were constructed with 300- to 700-bp insertion sizes, as illustrated in Rao et al. [11], and sequenced by sequencing by synthesis using the Illumina platform. Briefly, adapter sequences of raw reads were trimmed, and low-quality PE reads were removed to generate clean data.

### Genome assembly and quality assessment

Nanopore next-generation clean sequencing data were obtained by Canu v1.5 [12] software (RRID:SCR_015880). In the correction step, Canu v1.5 first selected longer seed reads with the settings “genomeSize = 780000000” and “corOutCoverage = 50.” SMARTdenovo [13] (default parameters) software was used to assemble the corrected data, and then the next-generation sequencing data were used to conduct three rounds of correction by Racon v1.4.21 (RRID:SCR_017642; default parameters) [14] and Pilon [15] v1.22 (RRID:SCR_014731; parameters: –mindepth 10 –changes –threads 4 –fix bases) software. The assembly results were evaluated by the read alignment rate, core gene integrity, and BUSCO evaluation. BWA [16] software (RRID:SCR_010910) was used to align short sequences on the reference genome. The CEGMA [17] v2.5 (default parameters) database and BUSCO v4.0.6 (RRID:SCR_015008; parameters: odb10, -c 24 -e 1e-3) [18] were used to evaluate the completeness of the assembly.

### Chromosomal-level genome assembly using Hi-C data

Before chromosome assembly, we first performed a preassembly for error correction of scaffolds, which required splitting scaffolds into segments of 50 kb on average. The Hi-C data were mapped to these segments using BWA (version 0.7.10-r789, default parameters) software. Only uniquely alignable read pairs whose mapping quality was greater than 20 were retained for further analysis. Invalid read pairs, including dangling-end and self-cycle, religation, and dumped products, were filtered by HiC-Prov2.8.1 (default parameters) [19]. The uniquely mapped data were retained to perform assembly with LACHESIS [20] software (RRID:SCR_017644). Any 2 segments that showed inconsistent connections with information from the raw scaffold were checked manually. These corrected scaffolds were assembled by LACHESIS. Parameters for running LACHESIS included CLUSTER_MIN_RE_SITES = 5, CLUSTER_MAXLINK_D ENSITY = 2, CLUSTER_ NONINFORMATIVE_RATIO = 2, ORDER_MIN_N_R ES_IN_TRUN = 5, and ORDER_MIN_N_RES_IN_ SHREDS = 5. After this step, placement and orientation errors exhibiting obvious discrete chromatin interaction patterns were manually adjusted.

### Genome annotation analysis

Due to the relatively poor conservation of interspecies repeat sequences, it is necessary to construct a unique repeat sequence database for predicting repeat sequences of specific species. LTR_FINDER [21] v1.05 (RRID:SCR_015247; default parameters) and RepeatScout [22] v1.0.5 (RRID:SCR_014653; default parameters) were used to construct the repetitive sequence database of *A. nanchuanensis* based on structure prediction and *de novo*sequencing theory. Then, the database was classified by PASTEClassifier v1.0 (RRID:SCR_017645; default parameters) [23] and merged with Repbase19.06 [24] (null) as the final repetitive sequence database. Finally, RepeatMasker [25] (RRID:SCR_012954; parameters: -nolow -no_is -norna -engine wublast -qq -frag 20,000) software was used to predict the repetitive sequences in the *A. nanchuanensis* genome based on the constructed repetitive sequence database.

The structures of coding genes were predicted by *ab initio* prediction, homologous species prediction, and unigene prediction using 3 different strategies. GENSCAN [26] v3.1 (RRID:SCR_013362), Augustus [27] v2.4 (RRID:SCR_008417), GlimmerHMM [28] v3.0.4 (RRID:SCR_002654), GeneID [29] v1.4, and SNAP [30] (version 2006–07-28) were used for *ab initio* prediction with default parameters. GeMoMa [31, 32] v1.3.1 (RRID:SCR_017646; default parameters) was used for homologous species prediction; Hisat [33] v2.0.4 (RRID:SCR_015530; parameters –max-intronlen 20,000, –min-intronlen 20) and Stringtie [34] v1.2.3 (RRID:SCR_016323; default parameters) were used for assembly based on reference transcripts. TransDecoder v2.0 (RRID:SCR_017647) and GeneMarkS-T [35] v5.1 (RRID:SCR_017648) were used for gene prediction with default parameters. PASA [36] v2.0.2 (RRID:SCR_014656; parameters: -align_tools gmap, -maxIntronLen 20,000) was used to predict unigene sequences based on the assembly data of nonparametric transcriptome. Finally, EVM [37] v1.1.1 (default parameters) was used to integrate the prediction results obtained by the above 3 methods, and PASA v2.0.2 (parameters: -align_tools gmap, -maxIntronLen 20,000) was used to modify the prediction results.

Noncoding RNAs were predicted by different strategies based on their structural characteristics. Rfam [38] v12.1 (RRID:SCR_007891; parameters: 1e-5) was used to identify microRNAs and rRNAs, and tRNAscan-SE [39] v1.3.1 (RRID:SCR_010835; parameters: 1e-5) was used to identify transfer RNAs (tRNAs).

The predicted protein sequences were compared with GenBlastA [40] v1.0.4 (RRID:SCR_020951; parameter: e-value -e 1e-5), and immature stop codons and transcoding mutations in the gene sequences were searched to obtain pseudogenes by GeneWise [41] 2.4.1 (RRID:SCR_015054; default parameters).

The predicted gene sequences were aligned to the nonredundant protein sequences [42], eukaryotic orthologous groups of proteins (KOG) [43], Gene Ontology (GO) [44], KEGG [45], TrEMBL [46], and other functional databases by BLAST [47] v2.2.31 (parameters: -evalue 1e-5), to perform KEGG pathway, KOG functional, GO functional, and other gene functional annotation analyses.

### Gene family and phylogenetic analysis

The protein sequences of *A. nanchuanensis* and their related species (*Arabidopsis thaliana* [48], *Amborella trichopoda* [49], *Populus trichocarpa* [50], *Actinidia chinensis* [51], *Vitis vinifera* [52], *Morus notabilis Schneid* [3], and *Theobroma cacao* [53]) were aligned to analyze gene replication within the species, the evolution between species, and the classification of species-specific genes. OrthoMCL [54] v2.0.9 (parameters: PercentMatchCutoff 50, EvalueExponentCutoff -5) software was used to classify the protein sequences of *A. nanchuanensis, A. thaliana, A. trichopoda, P. trichocarpa, A. chinensis, V. vinifera, M. notabilis*, and *T. cacao* to determine unique gene families in *A. nanchuanensis*.

PHYML [55] (RRID:SCR_014629; version: 20151210, parameters: -gapRatio 0.5 -badRatio 0.25 -model HKY85 -bootstrap 1000) was used to construct the evolutionary tree based on the single-copy protein sequences of *A. nanchuanensis* and 7 other species to study the evolutionary relationships among species. TimeTree (RRID:SCR_021162) [56] was used to select the known taxa for time calibration, and Mcmctree (parameter: default) was used to estimate the time of interspecies differentiation. CAFE 4.2 [57] (RRID:SCR_005983; parameter: lambda -l 0.002) was used to conduct gene family contraction and expansion analysis. The Branch model of the CodeML [58] module in PAML 4.7a (parameters: noisy = 3, verbose = 1, runmode = 0, seqtype = 1, CodonFreq = 2, clock = 0, aaDist = 0, model = 2, NSsites = 2, icode = 0, Mgene = 0, fix_kappa = 0, kappa = .3, fix_omega = 0, omega = 1, ncatG = 2, getSE = 0, RateAncestor = 0, Small_Diff = .45e-6, cleandata = 1, and fix_blength = 0) was used to analyze the selection pressure of single-copy genes and conduct the functional annotation and enrichment analysis.

LTR_FINDER v1.07 (RRID:SCR_015247; parameter: default) and PS SCAN [59] (version: 3.8.31, parameter: default) software were applied to search for long terminal repeat (LTR) sequences in the genome with scores greater than or equal to 6 points, and the repeated results were filtered with LTR_FINDER. The LTR flanking sequences were compared with MUSCLE [60] (version: 3.8.31, parameter: default), and the distance was calculated by DistMat software using a Kimura model with a 7.3 * 10^–9^ molecular clock.

## Results and discussion

### Initial characterization of the *A. nanchuanensis* genome

A total of 51.76 Gb of high-quality *A. nanchuanensis* data were obtained from the Illumina sequencing platform with an approximate 68× sequencing depth, and the genome size was calculated to be 761.07 Mb. Based on 4 ^ K/genome >200, a k-mer distribution map of K = 17 was constructed (Supplementary Fig. 1). The amount of repeated sequences content was estimated to be approximately 55.80%, and the heterozygosity was estimated to be approximately 0.93%, indicating that the *A. nanchuanensis* genome was highly heterozygotic and complex. Details are shown in Table 1.

**Table 1:** Sequence statistics of *Artocarpus nanchuanensis*

Illumina		Nanopore		Hi-C	
Data*	51.76 Gb	Data*	123.38 Gb	Data*	137.5 Gb
Depth/genome coverage	68.01×	Depth/genome coverage	160.34×	Depth	62×
Total k-mer	45,202,482,693	MaxLen	216,661 bp	Total Read Pairs	458,907,479
Genome	761.07 Mb	SeqNum	7,057,335	Genome	769.44 Mb
Heterozygosity	0.93%	N50Len	19,177 bp	Contig N50	1.78 Mb
Repeated	55.80%	N90Len	11,029 bp	Scaffold N50	25.15 Mb
Mapping rate	99.41%				

Data* mean the data have been filtered to be clean data. Depth/genome coverage means depth of sequencing data. MaxLen means the longest read length of sequencing data. SeqNum means the total read number of sequencing data. N50Len means the N50 length of sequencing data reads. N90Len means the N90 length of sequencing data reads.

A total of 128.71 Gb of reads were generated by the Nanopore platform, and 123.38 Gb of clean data were obtained after quality control. The average read length reached 17.48 kb, the N50 read length was 19.18 kb, and the total sequencing depth was approximately 160.34×. Clean data obtained by filtering out the low-quality data reached 7,057,335 reads. Details are shown in Table 1.

The total sequencing depth of the Illumina and Nanopore platforms was 228.35×.

### Genome assembly and completeness evaluation

After sequencing by Nanopore 3-generation sequencing, correction by Canu, assembly by SMARTdenovo, and polishing by Racon, Pilon software, a total of 769.44 Mb of *A. nanchuanensis* genome sequences was generated with 1,087 contigs, a 2.09-Mb contig N50, and a 402-kb contig N90 (Table 2). The contig N50/N90 and scaffold N50/N90 of *M. notabilis* were 34,476 bp/2,231 bp and 390,115 bp/11,563 bp, contig N50/N90 and scaffold N50/N90 of *B. papyrifera* were 171.17 kb/38.90 kb and 29.48 Mb/17.97 Mb [4], and contig N50/N90 of *F. microcarpa* were 907,868 bp/113,961 bp (Table 3) [61]. Compared with other reported Moraceae plants, the genome size of *A. nanchuanensis* is bigger (Table 3).

**Table 2:** Nanopore and Hi-C genome assembly statistics of *Artocarpus nanchuanensis*

Nanopore assembly results		Hi-C assembly results	
Contig number	1,087	Scaffold/contig number	809/1,364
Contig length	769,440,982 bp	Scaffold/contig length (bp)	769,496,482/769,440,982
Contig N50	2,094,024 bp	Scaffold/contig N50 (bp)	25,150,906/1,778,064
Contig N90	402,757 bp	Scaffold /contig N90 (bp)	20,179,149/200,000
Contig max	8,879,419 bp	Scaffold/contig max (bp)	32,505,427/8,646,128
		Gap total length (bp)	55,500
		GC content (%)	32.34

Contig represents the contig after error correction. Scaffold represents the scaffold generated after connection, and scaffold length exceeds 1 kb. Scaffold/contig number represents the number of scaffold and contig in the scaffold. Scaffold/contig length represents the length of scaffold and contig in the scaffold. Scaffold/contig N50 represents length of scaffold N50 and contig N50. Scaffold/contig N90 represents length of scaffold N90 and contig N90. Scaffold/contig max represents the length of the longest scaffold and longest contig. GC content represents the GC content percentage.

**Table 3:** Genome assembly quality comparison of *A. nanchuanensis* and its related Moraceae plants

	*Morus notabilis*(*Morus Linn*)	*Ficus microcarpa*(*Ficus*)	*A. nanchuanensis*(*Artocarpus*)
Sequencing technology	Illumina HiSeq 2000	Illumina, PacBio RS II, Hi-C	Illumina, Nanopore, Hi-C
Sequencing depth	236.82× (78.34 Gb, Illumina)	86.55× (36.87 Gb, Pacbio)	160.34× (123.83 Gb, Nanopore)
Contig/scaffold N50	34,476 bp/390,115 bp	907,868 bp/none	2.09 Mb/25.15 Mb
Contig/scaffold N90	2,231 bp/11,563 bp	113,961 bp/none	402.76 kb/20.18 Mb
Annotated genes	29,338	29,416	41,636
Repeat composition	127.98 Mb	198.23 Mb	422.78Mb
Unique k-mer in genome	2,198,905	5,732,202	26,038,922
k-mer in genome and reads	303168,905	425,981,208	769,420,329
QV	34.6019	31.9049	27.6449
Error rate	0.000346583	0.000644926	0.00171993
Solid k-mer in genome	210,228,161	250,755,719	520,815,448
Total solid k-mer in reads	220,698,486	317,069,158	756,078,105
Complete (%)	95.2558	79.0855	68.8838

Statistical alignment analysis of second-generation sequencing reads showed that clean reads located on the reference genome accounted for 99.41% of the total clean reads (363,371,475/365,545,724). The paired-end sequences of the correct size that were located on the reference genome accounted for 93.56% of the total clean reads (341,995,184/365,545,724). The core gene integrity assessment was performed by CEGMA v2.59. Here, 445 Core eukaryotic genes (CEGs) were present in assembly, accounting for 97.16% of all CEGs (445/458), while 232 highly conserved CEGs were present in the assembly, accounting for 93.55% of all CEGs (232/248). The database in BUSCO v4.0.6 contains 1,614 conserved core genes, and the number of complete genes present in the assembly is 1,583 (98.08%); details are shown in Supplementary Fig. 2.

### Hybrid assembly, scaffolding, and chromosome anchoring

**Figure 3:**
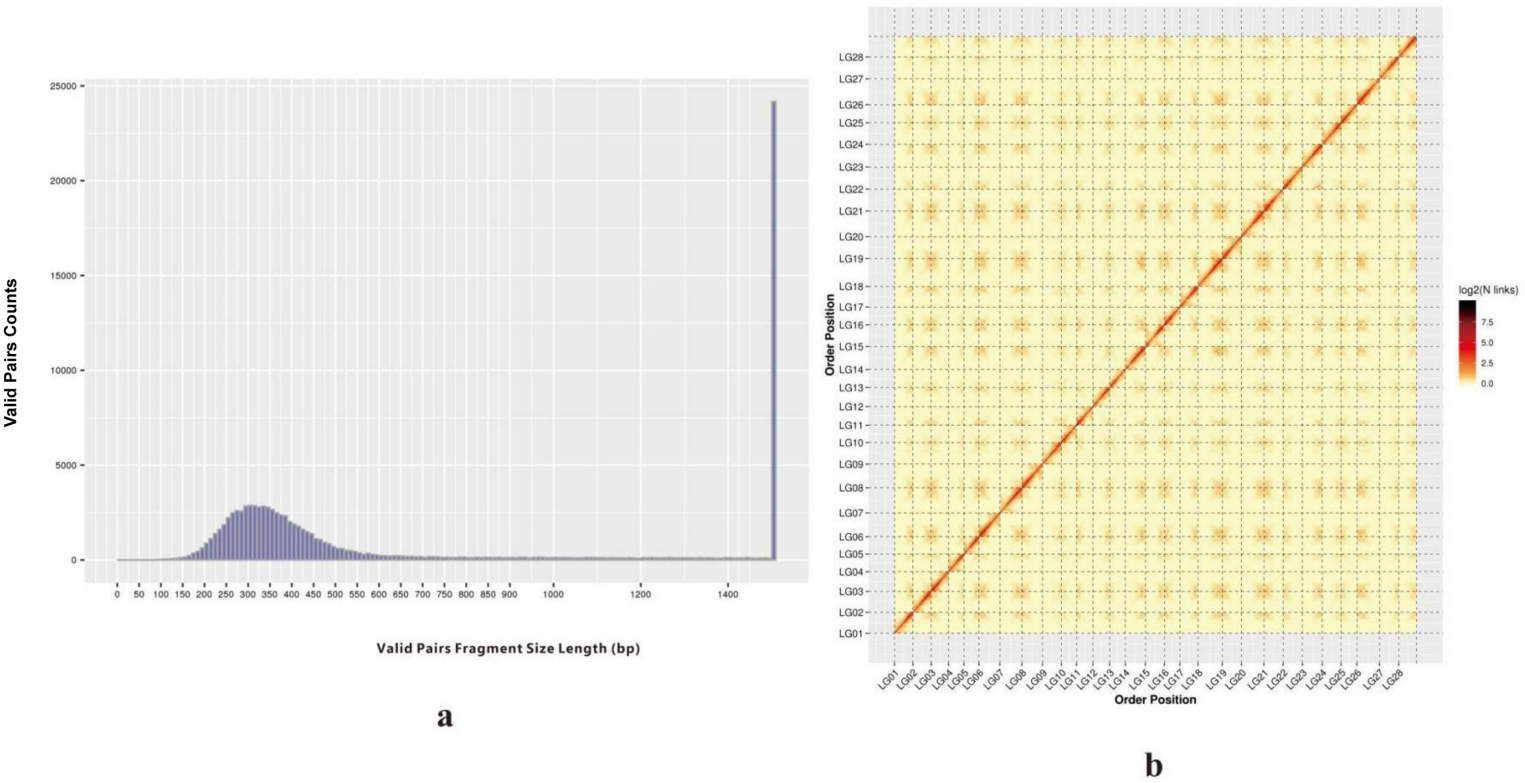
The analysis of Hi-C library construction and heatmap.

We obtained 137.5 Gb of clean Hi-C data (approximately 62× depth of the estimated genome). The clean Hi-C reads accounted for 179-fold coverage of the 769.44-Mb genome estimated by the Illumina platform for subsequent analysis (Table 1). To assess the quality of Hi-C data, we performed an insertion fragment length assessment, which showed a relatively narrow unimodal length distribution with the highest peak at approximately 300 bp, indicating that the dispersion degree of the inserted fragment length was small, the inserted fragment size was normal, and the purification of magnetic beads during library construction worked efficiently (Fig. 3). A total of 728,487,984 paired reads were genome-related mapping reads, accounting for 79.37% of the clean data. A total of 236,274,160 paired reads were uniquely mapped on the genome assembly, including 56,964,635 valid Hi-C paired reads. Details are shown in Supplementary Tables 1 and 2. Alignment efficiency, insert fragment length, and effective Hi-C data volume evaluation all indicated that the Hi-C libraries were constructed well.

After Hi-C assembly and manual adjustment, a total of 766.50 Mb of genomic sequences were located on 28 chromosomes through scaffold correction, clustered, ordered, and orientated, accounting for 99.62% of all genomic sequences, and the corresponding number of sequences was 1,336 (97.95%). Among the sequences located on the chromosome, the sequence length based on order and direction was 697.71 Mb, accounting for 91.02% of the total length of the sequences on the chromosomes (Table 4). The contig N50 and scaffold N50 were 1.78 Mb and 25.15 Mb, respectively, after error correction (Table 1). The final pseudochromosomes were constructed after manual adjustment.

**Table 4:** The Hi-C assembly statistics table of *A. nanchuanensis*

Group	Cluster number	Cluster length (bp)	Order number	Order length (bp)
LG01	46	26,514,107	24	24,676,255
LG02	41	26,638,661	16	24,489,134
LG03	30	24,254,703	16	23,044,270
LG04	34	22,404,888	13	20,644,200
LG05	33	21,646,681	16	20,177,649
LG06	35	29,133,579	18	27,822,153
LG07	69	32,924,820	27	29,467,719
LG08	45	29,858,101	20	27,605,363
LG09	77	29,556,483	29	25,185,028
LG10	45	22,896,788	20	20,243,522
LG11	67	25,833,105	20	21,750,724
LG12	37	24,385,337	15	22,370,729
LG13	47	23,481,896	24	21,098,278
LG14	46	29,162,015	19	26,857,340
LG15	61	28,431,484	30	25,341,045
LG16	32	21,965,556	16	20,879,538
LG17	41	25,915,114	19	24,032,910
LG18	49	34,941,454	27	32,502,827
LG19	54	29,520,137	21	25,685,935
LG20	50	32,513,478	18	29,815,261
LG21	50	28,639,915	21	25,613,043
LG22	42	27,392,871	24	25,873,084
LG23	42	28,655,389	16	26,447,344
LG24	52	27,753,222	24	25,148,606
LG25	46	23,720,417	16	21,152,151
LG26	63	33,995,937	28	30,220,329
LG27	58	28,458,315	24	25,577,647
LG28	44	25,907,258	22	23,985,053
Total (ratio %)	1336 (97.95%)	766,501,711 (99.62%)	583 (43.64%)	697,707,137 (91.02%)

The statistics do not include 100 Ns added by artificially connected pseudochromosomes.

The genomes of *A. nanchuanensis* and *Ficus microcarpa* were compared to verify the accuracy of the overlap across the 28 chromosomes, and the collinearity circle diagram indicates a high similarity of gene order between them (Fig. 2). A heatmap was drawn to evaluate the structure and quality of Hi-C assembly (Fig. 3). The figure indicated that the 28 pseudochromosomes could be distinguished easily, and the interaction signal intensity at the diagonal was significantly stronger than that at other locations within each pseudochromosome.

### Gene prediction and annotation

A total of 422.78 Mb (54.94%) of repeat sequences was detected; among these repeat elements, LTRs were the predominant type, whereas Class I/LTR/Copia and Class I/LTR/Gypsy accounted for 19.17% (147.52 Mb) and 16.86% (129.74 Mb). The details of the repeat sequences are shown in Supplementary Table 3.

A total of 41,636 protein-coding genes were predicted with a 3,797.54-bp average gene length, a 1,509.16-bp average exon length, and a 2,288.38-bp average intron length by *ab initio–*based, homologue-based, and RNA-seq–based methods; 27,262 genes were both obtained by the above 3 prediction methods (Table 5, Supplementary Fig. 3 and Supplementary Table 4). Based on GenBlastA v1.0.4 and GeneWise2.4.1, 1,905 pseudogenes were obtained, and their total length and average length were 4,825,668 kb and 2,533.16 kb, respectively (Supplementary Table 5).

**Table 5:** The prediction analysis of *A. nanchuanensis* coding gene

Prediction style and proportion			
Gene number	41,636	Coding DNA sequence (CDS) length	50,445,441 bp
Gene length	158,114,419 bp	CDS average length	1,211.58 bp
Gene average length	3,797.54 bp	CDS number	226,727
Exon length	62,835,343 bp	CDS average number	5.45
Exon average length	1,509.16 bp	Intron length	95,279,076 bp
Exon number	233,559	Intron average length	2,288.38 bp
Exon average number	5.61	Intron number	191,923
		Intron average number	4.61

A total of 39,596 genes were successfully annotated in the functional databases, accounting for 95.10% (39,596/41,636) of the predicted genes; details are shown in Supplementary Table 7. According to the noncoding RNA prediction results, the number of microRNAs was 138, belonging to 24 RNA families; there were 409 rRNAs, belonging to 4 RNA families; and there were 512 tRNAs, belonging to 24 families (Supplementary Table 6).

**Figure 4:**
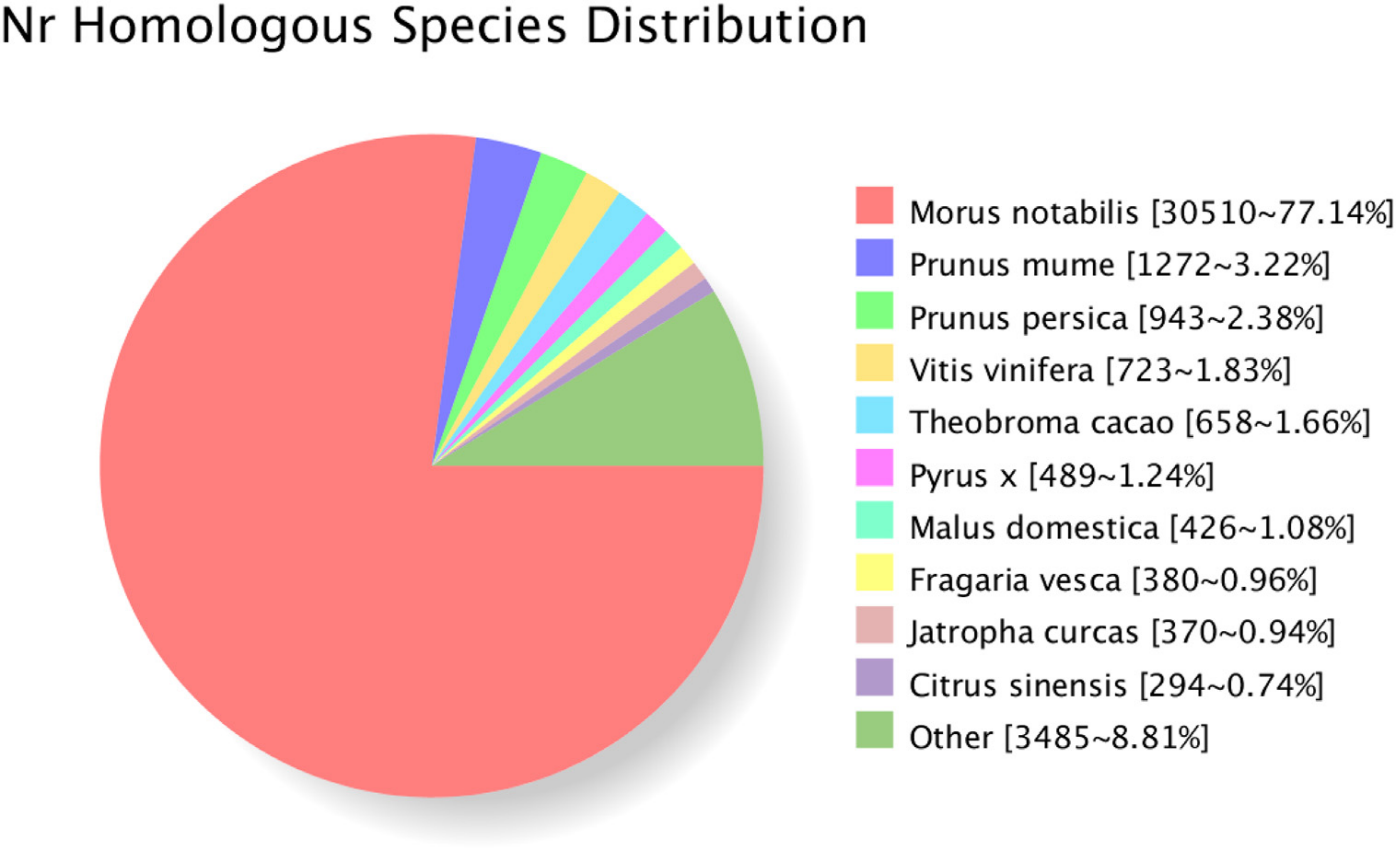
The Nr homologous species distribution of *A. nanchuanensis*.

The number of homologous genes between *A. nanchuanensis* and *M. notabilis* was 30,510, accounting for 77.14%, based on the Nr homologous species distribution, indicating high homology (Fig. 4). The KOG database is based on the phylogenetic relationships of protein-coding genes in bacteria, algae, and eukaryotes with complete genomes and classifies the gene products based on linear homology and at the functional level. A total of 21,567 (51.80%) *A. nanchuanensis* genes were annotated in the KOG database (Supplementary Table 7), and the annotation classification details are shown in Supplementary Fig. 4. The top 3 overexpressed genes were mainly involved in posttranslational modification, protein turnover, chaperones, signal transduction mechanisms, and transcription. The GO database was used to define and describe the genes and proteins, according to their involvement in biological processes, the components that make up cells, and the molecular functions they perform (Supplementary Fig. 5). Annotated gene number and repeat sequence size of *A. nanchuanensis* were 41,636 and 422.78 Mb, which are bigger than that previously reported for*M. notabilis* (29,338, 127.98 Mb), *F. microcarpa* (29,416, 198.23 Mb), and *B. papyrifera* (30,512, 190.23 Mb) [3,4, 61], indicating the high quality of sequencing and annotation for *A. nanchuanensis* (Table 3).

Nucleotide-binding site and leucine-rich repeat (NBS-LRR) has been well known as a major plant disease resistance gene; the gene numbers of NBS-LRR in papaya, watermelon, arabidopsis, grape, tomato, and notabilis were 55, 44, 166, 504, 251, and 142, respectively, while the gene number of *A. nanchuanensis* was 316 [3, 51]. As particular *NBS-LRR* genes recognize specific pathogen effectors, the number of *NBS-LRR* genes may represent good potential for pathogen recognition, which is consistent with the strong resistance to disease of *A. nanchuanensis*. To minimize the dangers of insect infestation, plants evolved a defense mechanism by expressing plant protease inhibitors (PIs) to interfere with digestive systems of insects, and 8 Glu *Streptomyces griseus* protease inhibitor genes were detected in *A. nanchuanensis* [50]. *PI* and *NBS-LRR* genes are reasonably important for defense response in *A. nanchuanensis* ancient species.

KEGG is the main public database of the pathway, and 129 metabolic pathways of *A. nanchuanensis* were finally obtained. Plant–pathogen interaction metabolic pathways may closely relate to the resistance of disease and insect pests. Abundant biosynthesis pathways of vitamins, flavonoid, and gingerol may reveal the theoretical basis of the rare medicinal value of *A. nanchuanensis*.

### Comparative genomics

**Figure 5:**
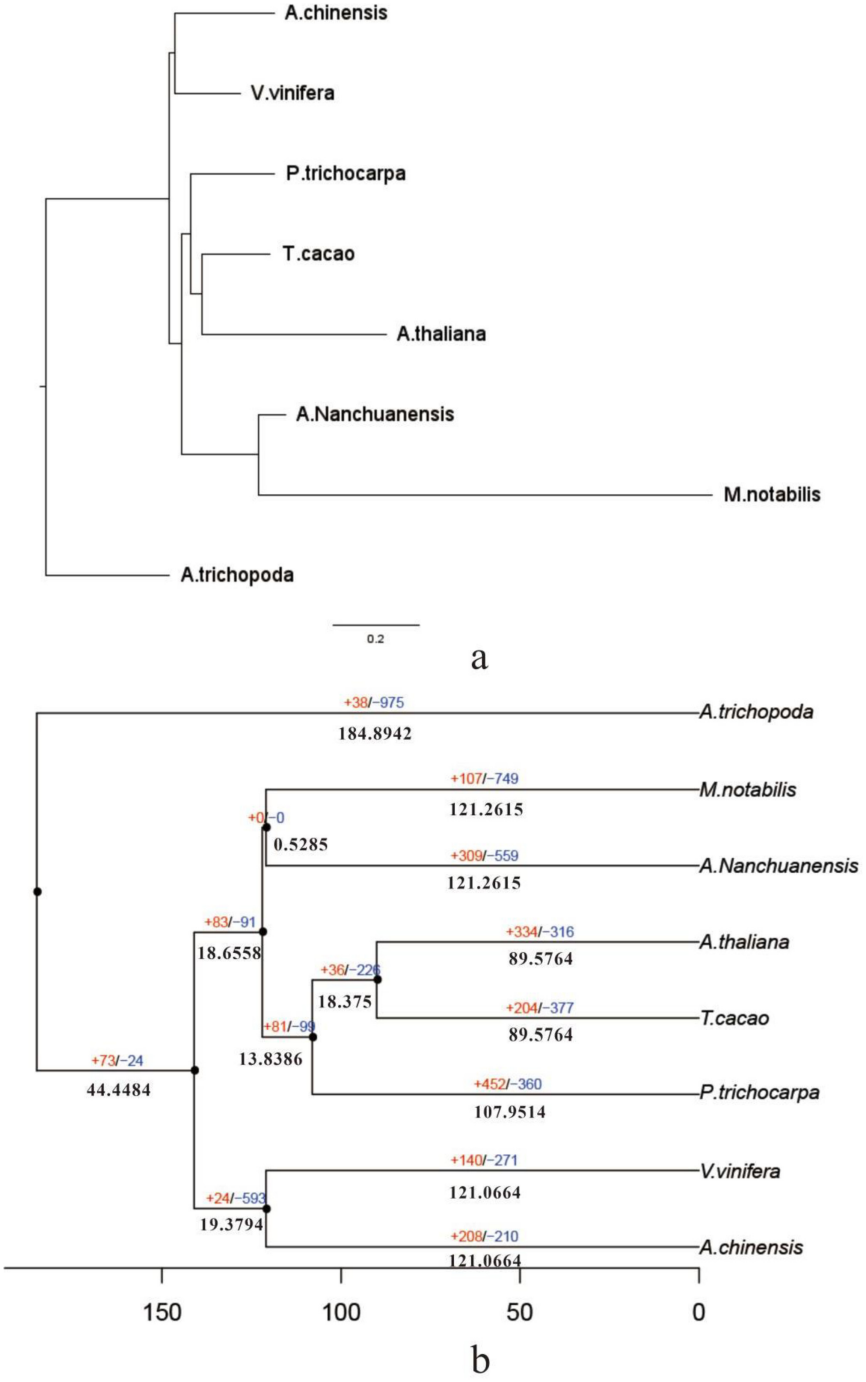
The phylogenetic and gene family analysis of *A. nanchuanensis* and related species.

The protein sequences between *A. nanchuanensis* and its related species (*A. thaliana, A. trichopoda, P. trichocarpa, A. chinensis, V. vinifera, M. notabilis*, and *T. cacao*) were compared, and 33,925 genes out of 41,636 *A. nanchuanensis* predicted genes were clustered into 15,436 gene families, of which 512 were unique to *A. nanchuanensis* (Table 6 and Supplementary Fig. 6). In the phylogenetic tree of *A. nanchuanensis* and its related species, *A. nanchuanensis* diverged from *M. notabilis* approximately 0.5285 million years ago (Mya) by Mcmctree estimation, as well as diverged from *A. chinensis* and *V. vinifera* approximately 19.3794 Mya and from *A. thaliana,T. cacao,and P. trichocarpa* approximately 18.6558 Mya, which supports the close relationship between *A. nanchuanensis*and*M. notabilis* (Fig. 5). This result was confirmed by the analysis of homologous species distribution, transversions at fourfold degenerate sites (4DTv), and chromosome gene order.

**Table 6:** Statistical classification of gene families

Name	Total gene	Cluster	Total family	Unifamily
*A. thaliana*	27,369	23,106	12,753	726
*A. trichopoda*	16,986	15,058	11,147	254
*P. trichocarpa*	41,335	33,270	14,725	950
*A. chinensis*	39,040	25,888	12,648	1,327
*V. vinifera*	26,346	19,238	12,682	665
*M. notabilis*	26,965	20,423	14,794	524
*T. cacao*	21,432	20,070	13,810	176
*A. nanchuanensis*	41,636	33,925	15,436	512

Total gene: the number of total genes. Cluster: the number of genes involved in family classification. Total family number: the number of gene families that can be divided. Unifamily: the number of unique gene families.

In the evolutionary process, gene families in contraction and expansion generally imply strong functional changes. According to the evolutionary relationships among species and the results of gene family clustering, 309 gene families in contraction and 559 gene families in expansion were detected in *A. nanchuanensis* after divergence from mulberry (Fig. 5). These gene families in contraction are mainly related to the F-box domain, cystatin domain, protein kinase domain, and ring finger domain functions (Table 7). Refers to the common ancestor, except for *A. thaliana* and *P. trichocarpa*," the number of gene families in contraction is bigger than that of expansion among other species, suggesting that more gene families in most species experienced contraction than expansion during adaptive evolution, and the living environment of *A. thaliana* and *P. trichocarpa* may be challengeable, expanding their gene family to cope with the living environment.

**Table 7:** The annotation of protein gene family

Gene family	Pfam	Function
GF_12 673	PF00646.28	F-box domain
GF_10 548	PF00031.16	Cystatin domain
GF_8	PF00069.20	Protein kinase domain
GF_13 176	PF13639.1	Ring finger domain

Gene family refers to the gene family cluster. Pfam refers to the ID of protein family alignment to the Pfam database. Function indicates the function of the protein family that can be aligned.

**Figure 6:**
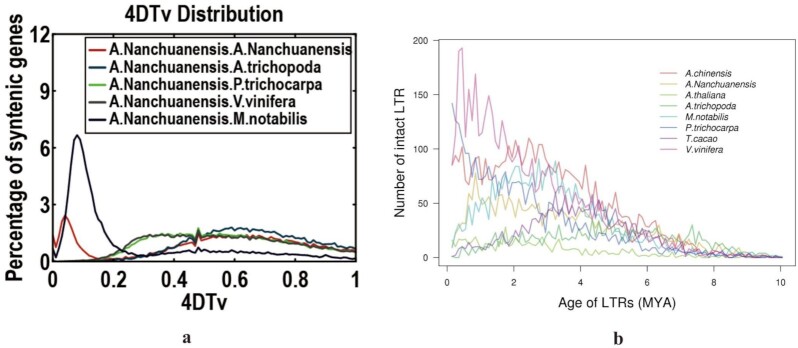
The 4DTv distribution and LTR insertion time analysis among *A. nanchuanensis* and other related species.

EVM0035972.1, EVM0031735.1, EVM0026117.1, and EVM0015119.1 were found to be rapidly evolving genes, and details on these genes and their annotated functions are shown in Table 8 and Supplementary Fig. 7. 4DTv are neutral genetic distances that can be used to estimate the relative timing of evolutionary events [62]. According to the homologous gene pairs between 2 species or within species themselves, the ratio of each homologous gene to the 4DTv mutation site was calculated, and a 4DTV distribution map was made (Fig. 6). The peak of the 4DTv distribution among *A. nanchuanensis* and *M. notabilis* was closer to the currentRefers to the common ancestor, except for *A. thaliana* and *P. trichocarpa* than that of *A. nanchuanensis* and other species, indicating that the differentiation time of *A. nanchuanensis* and *M. notabilis* appeared recently, suggesting a closer genetic relationship between them. In ancient times, the 4DTv distribution curves of *A. nanchuanensis* and other species were similar, reflecting that these species might share similar whole-genome duplication events. Moreover, the 4DTv distribution of *A. nanchuanensis* had a small peak at 0.05, which suggests that some genomic fragments duplicated recently.

**Table 8:** The rapidly evolving genes selected by CodeML

GeneID	*P*value	Sites
EVM0035972.1	0.05	298,G,0.993**
EVM0031735.1	0.06	74,E,0.984*
EVM0026117.1	0.35	68,K,0.997**
EVM0015119.1	0.00	232,E,0.990**

GeneID means the ID of gene, ω0 indicates the ka/ks for the studied species, ω1 is the average ka/ks for other species, and ω2 is ka/ks for the whole evolutionary tree. * represents a posteriori probability ≥0.95, ** represents a posteriori probability ≥0.99.

LTR accumulation is able to reflect that the species may cope with some environmental stresses on its survival [21]. LTR insertion time among *A. nanchuanensis* and 7 other related species shows that the living environment of *A. nanchuanensis* is relatively stable. The narrow peak of LTR insertion time around 1 Mya indicated some environment stress or environment change has been imposed on *A. nanchuanensis* and its living environment (Fig. 6).

## Conclusion

In this study, a high-quality genome assembly and annotation information for *A. nanchuanensis* were first reported, resulting in the first reference genome for the *Artocarpus* genus. A total of 123.38 Gb of clean reads were obtained and a 769.44-Mb genome was assembled, which was larger than that of the sequenced *M. notabilis* and *B. papyrifera*. The clean reads mapped percentage (99.41%), CEGs and highly conserved CEG present in assemblies (97.16%, 93.55%), and BUSCO conserved gene core set coverage (98.08%) indicated that the current assembly covers most of the *A. nanchuanensis* genome. The *A. nanchuanensis* genome size estimated by k-mer analysis was 761.07 Mb, and the assembly was 769.44 Mb. These data suggested that this assembly was mostly representative of the complete *A. nanchuanensis* genome and indicated the high quality of *A. nanchuanensis* genome assembly. *A. nanchuanensis* and *M. notabilis* are both composed of 7 chromosome pairs, and their high similarity in gene order indicated high continuity between *A. nanchuanensis* and *M. notabilis*,as well as the high quality of the *A. nanchuanensis* genome assembly.

*NBS-LRR, PI* plant disease and insect resistance genes were detected in the gene prediction and annotation analysis of *A. nanchuanensis*, and they represent good potential for pathogen recognition, which is consistent with the inherent strong resistance to pests and diseases of *A. nanchuanensis*. Several anti-inflammatory metabolism and anti-inflammatory substance synthesis pathways were detected, which may be related to the unique antiallergic function of *A. nanchuanensis*. Study of relevant functions and metabolic pathways reveals fruit maturation, nutrient metabolism, and disease resistance of *A. nanchuanensis*.

Gene families in contraction and expansion generally imply strong functional changes, unigenes indicate special species function, and the in-depth study of the above gene provides the research foundation for the unique features of *A. nanchuanensis*. Meanwhile, the LTR insertment analysis may indicate the stability of an ecological environment in which*A. nanchuanensis* lives. Species genome analysis not only reveals their functions and evolutionary relationships but also reflects their growth environment.

This high-quality genome of *A. nanchuanensis* will lay a solid foundation for the conservation, rational development, and utilization of critically endangered species in the future. It is a valuable resource for the genetic improvement and better understanding of *A. nanchuanensis* genomic evolution. This genome will also be invaluable in developing new varieties and addressing issues of agronomic and/or biological importance such as fruit development and maturation, nutrient metabolism of fruits, and disease resistance of *A. nanchuanensis* and related plant species.

## Data availability statement

The whole raw sequence reads produced by Illumina novaseq, Pacbio sequel II, and ONT PromethlON Beta have been deposited at the NCBI Sequence Read Archive under BioProject number PRJNA624965 and BioSample ID SAMN14589993 and SAMN26429610 for *A. nanchuanensis*. Raw sequencing data (Nanopore, Illumina, Hi-C, RNA-seq data) have been deposited in the Sequence Read Archive database as SRR11671532, SRR11659666, SRR11659674, and SRR11623450/SRR11668249. All additional supporting data and materials are available in the *GigaScience* GigaDB database [63].

## Additional Files

**Supplementary Table 1**. The clean data and genome comparison results of *A. nanchuanensis*.

**Supplementary Table 2**. The Hi-C sequencing data types and proportion of *A. nanchuanensis*.

**Supplementary Table 3**. The repeat sequences analysis of *A. nanchuanensis*.

**Supplementary Table 4**. The gene prediction results of *A. nanchuanensis*.

**Supplementary Table 5**. Pseudogene annotation statistics of *A. nanchuanensis*.

**Supplementary Table 6**. The statistical results of noncoding RNA.

**Supplementary Table 7**. Functional annotation statistics of *A. nanchuanensis*.

**Supplementary Fig. 1**. The k-mer distribution map of *A. nanchuanensis*.

**Supplementary Fig. 2**. The BUSCO genome assembly evaluation.

**Supplementary Fig. 3**. Distribution of the number of genes among the 3 methods.

**Supplementary Fig. 4**. The KOG functional annotation classification of *A. nanchuanensis*.

**Supplementary Fig. 5**. The GO secondary node annotation classification of *A. nanchuanensis*.

**Supplementary Fig. 6**. The family clustering statistics among different species.

**Supplementary Fig. 7**. The classification annotation statistics for GO.

giac042_GIGA-D-21-00106_Original_Submission

giac042_GIGA-D-21-00106_Revision_1

giac042_GIGA-D-21-00106_Revision_2

giac042_Response_to_Reviewer_Comments_Original_Submission

giac042_Response_to_Reviewer_Comments_Revision_1

giac042_Reviewer_1_Report_Original_Submission Jian-Feng Mao, Ph.D. -- 7/11/2021 Reviewed

giac042_Reviewer_1_Report_Revision_1 Jian-Feng Mao, Ph.D. -- 10/31/2021 Reviewed

giac042_Reviewer_2_Report_Original_Submission Caroline Belser -- 7/26/2021 Reviewed

giac042_Reviewer_2_Report_Revision_1 Caroline Belser -- 11/8/2021 Reviewed

giac042_Reviewer_2_Report_Revision_2 Caroline Belser -- 2/11/2022 Reviewed

giac042_Supplemental_Figures_and_Tables

## Abbreviations

4DTv: transversions at fourfold degenerate sites; BLAST: Basic Local Alignment Search Tool; bp: base pair; BUSCO: Benchmarking Universal Single-Copy Orthologs; CEGMA: Core Eukaryote Gene Mapping Approach; Gb: gigabases; GO: Gene Ontology; Hi-C: high-throughput/resolution chromosome conformation capture; kb: kilobase; KEGG: Kyoto Encyclopedia of Genes and Genomes; KOG: eukaryotic orthologous groups of proteins; LTR: long terminal repeat; Mb: megabase; mRNA: messenger RNA; Mya: million years ago; NBS-LRR: nucleotide-binding site and leucine-rich repeat; PE: paired end; PI: plant protease inhibitor; rRNA: ribosomal RNA; tRNA: transfer RNA.

## Author contributions

JH, SB, XD, KK, and CC conceived and designed the study; JH, SB, XD, JD, XW, and QL collected the samples; QL, YZ, and YL performed DNA sequencing and Hi-C experiments; YC and LF performed RNA sequencing; JH, QL, and YZ estimated the genome size, assembled the genome, and assessed the assembly quality; YC and LF performed the genome annotation and functional genomic analysis; and SX, JH, and XD wrote the manuscript. All authors read, edited, and approved the final manuscript for submission.

## Conflict of interest

The authors declare no competing interests.

## Funding

This work was supported by the Chinese Ministry of Education, Chongqing Nanchuan Biotechnology Research Institute, Sichuan and Chongqing government and Key Laboratory of Bio-Resources and Eco-Environment of Ministry of Education, College of Life Sciences, Sichuan University.
