# Supplementary material for: A chromosome-level genome assembly of Artocarpus nanchuanensis (Moraceae), an extremely endangered fruit tree
Source: Gigascience. 2022 Jun 14;11:giac042. doi: 10.1093/gigascience/giac042 (PMC9197682; doi:10.1093/gigascience/giac042)
Supplement: giac042_GIGA-D-21-00106_Original_Submission [file giac042_giga-d-21-00106_original_submission.pdf]

GigaScience

A chromosome-level genome assembly of Artocarpus nanchuanensis

--Manuscript Draft--

**Manuscript Number:**	GIGA-D-21-00106
**Full Title:**	A chromosome-level genome assembly of Artocarpus nanchuanensis
**Article Type:**	Research
**Funding Information:**	
**Abstract:**	Artocarpus.nanchuanensis (Moraceae) naturally distributed China, is an evergreen Artocarpus genus representative and extremely endangered tree species in China. In this study, we obtained a high-quality chromosome-scale genome assembly and annotation information for A.nanchuanensis using inter-grated approaches, including Illumina, Nanopore sequencing platform as well as Hi-C. A total of 128.71 gigabases (Gb) raw Nanopore Sequel reads were generated from 20 kb libraries. After filtering, 123.38 Gb clean reads were obtained with 160.34x coverage depth and the average length of reads reached 17.48Kb. The final assembled A.nanchuanensis genome was 769.44 Mb with a contig N50 of 2.09 Mb, and 99.62% (766.50 Mb) of the assembly data was assigned to 28 pseudochromosomes. Gene modelling predicted 41,636 protein-coding genes, of which 95.10% were annotated. The genome assembly integrity was evaluated by BUSCO (v4.0.6), and 98.08% conserved genes could be found in the assembly data. The disclosure of A.nanchuanensis genome sequence information provides an important resource to expand our understanding of the molecular mechanism in its unique biological processes and nutritional, medicinal benefits.
**Corresponding Author:**	Xianping Ding<br>Sichuan University<br>Chengdu , Sichuan, CHINA
**Corresponding Author Secondary Information:**	
**Corresponding Author's Institution:**	Sichuan University
**Corresponding Author's Secondary Institution:**	
**First Author:**	Jiaoyu He
**First Author Secondary Information:**	
**Order of Authors:**	Jiaoyu He
	Shanfei Bao
	Junhang Deng
	Qiufu Li
	Shiyu Ma
	Zhilin Song
	Yanru Cui
	Yiran Liu
	Xia Wei
	Xianping Ding
	Kehui Ke
	Chaojie Chen
**Order of Authors Secondary Information:**	
**Additional Information:**	

Powered by Editorial Manager® and ProduXion Manager® from Aries Systems Corporation

Question	Response
Are you submitting this manuscript to a special series or article collection?	No
**Experimental design and statistics**<br><br>Full details of the experimental design and statistical methods used should be given in the Methods section, as detailed in our Minimum Standards Reporting Checklist. Information essential to interpreting the data presented should be made available in the figure legends.<br><br>Have you included all the information requested in your manuscript?	Yes
**Resources**<br><br>A description of all resources used, including antibodies, cell lines, animals and software tools, with enough information to allow them to be uniquely identified, should be included in the Methods section. Authors are strongly encouraged to cite Research Resource Identifiers (RRIDs) for antibodies, model organisms and tools, where possible.<br><br>Have you included the information requested as detailed in our Minimum Standards Reporting Checklist?	Yes
**Availability of data and materials**<br><br>All datasets and code on which the conclusions of the paper rely must be either included in your submission or deposited in publicly available repositories (where available and ethically appropriate), referencing such data using a unique identifier in the references and in the "Availability of Data and Materials" section of your manuscript.	Yes

Powered by Editorial Manager® and ProduXion Manager® from Aries Systems Corporation

Have you have met the above requirement as detailed in our Minimum Standards Reporting Checklist?	

Manuscript Click here to access/download;Manuscript;paper_revise_6.pdf

A chromosome-level genome assembly of *Artocarpus nanchuanensis*

Jiaoyu He[1,2], Shanfei Bao[1,2], Junhang Deng[1,2], Qiufu Li[1,2], Shiyu Ma[1,2], Zhilin Song[1,2], Yiran Liu[1,2], Yanru Cui[1,2], Xia Wei[1,2], Xianping Ding[1,2]*, Kehui Ke[3], Chaojie Chen[3].

1 Key Laboratory of Bio-Resources and Eco-Environment of Ministry of Education, College of Life Sciences, Sichuan University, Chengdu 610065, Sichuan, P.R.China.

2 Chongqing Nanchuan biotechnology research institute, Bio-resource Research and Utilization Joint Key Laboratory of Sichuan and Chongqing, Sichuan and Chongqing, P.R.China.

3 Biomarker Technologies Corporation, Beijing 101300, China.

Address for Correspondence: Institute of Medical Genetics, College of Life Sciences, Sichuan University, Chengdu 610064, China.

* Corresponding author:

Institute of Medical Genetics, College of Life Sciences, Sichuan University, Chengdu 610064, China.

E-mail: brainding@scu.edu.cn

Telephone: 86-028-85413096

Fax: 86-028-85415895

Email address:

Jiaoyu He: 1061355567@qq.com; Shanfei Bao: 715714892@qq.com;

Junhang Deng: 1916358148@qq.com; Qiufu Li: lqf1192069072@126.com;

Shiyu Ma: 895686227@qq.com; Zhilin Song: 442126776@qq.com;

Yanru Cui: 512927123@qq.com; Yiran Liu: 532154290@qq.com;

Xia Wei: 531197860@qq.com; Xianping Ding: brainding@scu.edu.cn;

Kehui Ke: kehui.ke@outlook.com; Chaojie Chen: 352300595@qq.com.

Abstract

Artocarpus.nanchuanensis (Moraceae) naturally distributed China, is an evergreen Artocarpus genus representative and extremely endangered tree species in China. In this study, we obtained a high-quality chromosome-scale genome assembly and annotation information for *A.nanchuanensis* using inter-grated approaches, including Illumina, Nanopore sequencing platform as well as Hi-C. A total of 128.71 gigabases (Gb) raw Nanopore Sequel reads were generated from 20 kb libraries. After filtering, 123.38 Gb clean reads were obtained with 160.34x coverage depth and the average length of reads reached 17.48Kb. The final assembled *A.nanchuanensis* genome was 769.44 Mb with a contig N50 of 2.09 Mb, and 99.62% (766.50 Mb) of the assembly data was assigned to 28 pseudochromosomes. Gene modelling predicted 41,636 protein-coding genes, of which 95.10% were annotated. The genome assembly integrity was evaluated by BUSCO (v4.0.6), and 98.08% conserved genes could be found in the assembly data. The disclosure of *A.nanchuanensis* genome sequence information provides an important resource to expand our understanding of the molecular mechanism in its unique biological processes and nutritional, medicinal benefits.

Key words: *A.nanchuanensis*, nanopore sequencing, genome assembly, gene annotation, Hi-C

1 Introduction

A.nanchuanensis mainly distributed in Chongqing Nanchuan, is the new generation of south urban greening tree species with high quality and excellent fast-growing characteristics, can live in acidic soil and atmospheric pollution heavier environment with a strong ability to resist pollution and disease[12]. The fruit contains a variety of polysaccharide, amino acids, trace elements and vitamins, has a good control effect on the constipation and other intestinal diseases[2]. The fruit and bark have been used as the treatment of skin disease in Chongqing Nanchuan for a long time. Those features persistent cause the attention of researchers[1]. A high-quality reference genome is needed for this valuable species to promote the molecular mechanism study that related to its nutritional and medicinal value, as well as the study of individual genome structure, genome evolution and species diversity.

The genome of *Moraceae Mulberry* and *Paper Mulberry* have been made in detail. The draft genome sequence of mulberry tree, including 78.34 billion high-quality bases, were assembled into 330.79-Mb mulberry genome with a scaffold

N50 length of 390,115 bp and contig N50 length of 34,476 bp[3]. And the assembled genome of Paper Mulberry was 396.86Mb with a scaffold N50 length of 1,034,263 bp[4]. The genome data analysis of Mulberry and Paper Mulberry with the important functions of fiber development, lignin and flavonoids metabolism, nitrogen metabolism, metal tolerance and stress resistance evolution were studied, but the genome details of *A.nanchuanensis* were unrevealed.

To protect and make full use of its rare value, we applied a combined strategy involving Nanopore single molecule sequencing and high-pass chromosome conformation capture (Hi-C) technologies to generate sequencing data for chromosomal genome construction and annotation for the *A.nanchuanensis* (Fig.1), that not only provide the necessary resources for the genome size selection, but also provide convenience for research of reproduction and species evolution based on speciation and local environment, which is beneficial to the medicinal economic value traits study.

2 Materials and methods

2.1 Sample and DNA extraction

The oldest *A.nanchuanensis* tree surviving in Nanchuan district was selected as the source of sample (Fig. 2). Its fruits, young leaves and roots were preserved in liquid nitrogen until DNA extraction. The samples of genome were young leaves. The leaves and fruits in the different growth stages were uniform mixed for transcriptome analysis. The quality and concentration of genomic DNA extracted by CTAB (Cetyltrimethylammonium bromide) method were checked by 1% agarose gel electrophoresis and Qubit fluorimeter[5]. The extracted high-quality DNA was used for subsequent Nanopore and Illumina sequencing[5].

2.2 Library construction and High-throughput sequencing

ONT Library with 20-kb insertion size were constructed for the Nanopore platform according to the manufacturers' protocols. Using the appropriate method to extract the DNA from the sample as well as detect the concentration and purity of DNA by NanoDrop and Qubit; the integrity of DNA was detected by pulsed field electrophoresis and large segments were filtered by the BluePippin™ System. The large segments DNA, ONT Template prep kit (SQK-LSK109) and NEB Next FFPE DNA Repair Mix kit were used to prepare a library. High quality library is sequenced on the ONT PromethION Beta platform with Corresponding R9 cell and ONT sequencing reagents kit (EXP-FLP001.PRO.6).

Illumina sequencing library was prepared for the following genome size estimation, genome assembly correction and evaluation. The paired-end (PE) library with 350 bp insertion size was prepared for the Illumina platform according to the manufacturers' protocols (San Diego, 112 CA, USA) and subjected to PE (2 × 150 bp) sequencing on an Illumina novaseq platform (Illumina, San Diego, CA, USA). The low-quality bases, adapter sequences, and duplicated sequences reads were filtered out to obtain the clean reads for subsequent analysis.

The Hi-C fragment libraries was constructed with 300-700 bp insertion size as illustrated in Rao et al[6], and sequencing by sequencing By Synthesis (SBS) technique through Illumina platform. Briefly, adapter sequences of raw reads were trimmed and low-quality PE reads were removed for clean data.

2.3 Genome assembly and quality assessment

Nanopore three-generation sequencing clean data was obtained by Canu[7] software. In the correction step, Canu first selects longer seed reads with the settings 'genomeSize=780000000' and 'corOutCoverage=50'. SMARTdenovo software was used to assemble the corrected data, then the three-generation and second-generation sequencing data were used to conduct three rounds calibration by Racon[8] and Pilon[9] software respectively. The assembly results were evaluated by the reads alignment rate, core gene integrity, and BUSCO evaluation. BWA[10] software was used to compare the short sequences obtained from second-generation sequencing with the reference genome. CEGMA v2.5[11] (default parameters) database and the BUSCO v4.0.6[12] software were used to evaluated the integrity of the assembled genome.

2.4 Chromosomal-level genome assembly using Hi-C data

Before chromosomes assembly, we first performed a preassembly for error correction of scaffolds which required the splitting of scaffolds into segments of 50 kb on average. The Hi-C data were mapped to these segments using BWA (version 0.7.10-r789, default parameters) software. Only uniquely alignable pairs reads whose mapping quality more than 20 were remained for further analysis. Invalid read pairs, including Dangling-End and Self-cycle, Re-ligation and Dumped products, were filtered by HiC-Prov2.8.1[13]. The uniquely mapped data were retained to perform assembly by LACHESIS[14] software. Any two segments which showed inconsistent connection with information from the raw scaffold were checked manually. These corrected scaffolds were assembled by LACHESIS. Parameters for running LACHESIS included: CLUSTER_MIN_RE_SITES = 5； CLUSTER_MAXLINK_D

ENSITY = 2；CLUSTER_ NONINFORMATIVE_RATIO = 2； ORDER_MIN_N_RES_IN_TRUN = 5； ORDER_MIN_N_RES_IN_ SHREDS = 5. After this step, placement and orientation errors exhibiting obvious discrete chromatin interaction patterns were manually adjusted.

2.5 Genome annotation analysis

Due to the relatively poorly conservation of interspecies repeat sequences, it is necessary to construct a particular repeat sequence database for predicting the repeats sequences of specific species. LTR_FINDER v1.05[15] and RepeatScout v1.0.5[16] were used to construct the repetitive sequence database based on the structure prediction and de novo sequencing theory for '*A.nanchuanensis*' with default parameters. Then, the database was classified by PASTEClassifier (default parameters) and merged with the Repbase 19.06[17] (null) as the final repetitive sequence database, finally RepeatMasker (parameters -nolow -no_is -norna -engine wublast -qq -frag 20000)[18] software was used to predict the repetitive sequence of this genome based on the constructed repetitive sequence database.

The structure of Coding genes in the genome were predicted by ab initio prediction, homologous species prediction and Unigene prediction three different strategies. Genscan_3.1[19], Augustus_3.1[20], GlimmerHMM[21] v3.0.4, GeneID[22] v1.4 and SNAP[23] (version 2006-07-28) were used for ab initio prediction with default parameters. GeMoMa[2425] v1.3.1 (default parameters) was used for homologous species prediction; Hisat[26] v2.0.4 (parameters --max-intronlen 20000，--min-intronlen 20) and Stringtie[27] v1.2.3 (default parameters) were used for assembly based on reference transcripts. TransDecoder v2.0 and GeneMarkS-T[28] v5.1 were used for gene prediction with default parameters. PASA[29] v2.0.2 (parameters -align_tools gmap，-maxIntronLen 20000) was used to predict Unigene sequences based on transcriptome data unreferenced assembly. Finally, EVM[30] v1.1.1 (default parameters) was used to integrate the prediction results obtained by the above three methods, and PASA v2.0.2 was used to modify the prediction results.

The non-coding RNAs were predicted by different strategies according to the structural characteristics of different non-coding RNAs. Blastn was used to identify microRNAs and rRNAs by genome-wide comparison based on Rfam[31] database. tRNAscan-SE[32] was used to identify tRNA.

By comparing the predicted protein sequences with GenBlastA[33] v1.0.4 (parameter for blast: The e-value), and search for immature stop codon and

transcoding mutation in the gene sequence to obtain pseudogenes by GeneWise[34] 2.4.1 (default parameters).

The predicted gene sequences were aligned to the Non-redundant protein sequences (NR)[35], eukaryotic orthologous groups of proteins (KOG)[36], Gene ontology (GO)[37], Kyoto Encyclopedia of Genes and Genomes (KEGG)[38], TrEMBL[39] and other functional databases by BLAST[40] v2.2.31 (-evalue 1e-5), preform the KEGG pathway, KOG functional, GO functional and other gene functional annotation analysis, to functionally annotate the predicted genes.

2.6 Gene family and phylogenetic analysis.

The protein sequences of *A.nanchuanensis* and their related species (*A.thaliana*, *A.trichopoda*, *P.trichocarpa*, *A.chinensis*, *V.vinifera*, *M.notabilis*, *T.cacao*) were aligned. And based on the sequence alignment results, the known gene sequences and structures were compared to analyzed gene replication within the species, the evolution between species and the classification of species-specific genes. OrthoMCL[41] v2.0.9 software was used to classify the protein sequences of *A.nanchuanensis*, *A.thaliana*, *A.trichopoda*, *P.trichocarpa*, *A.chinensis*, *V.vinifera*, *M.notabilis*, *T.cacao*, as well as to find out the gene family that unique to *A.nanchuanensis*. The evolutionary relationship, time of inter-species differentiation and gene family contraction and expansion analysis were estimated by PGYML[42], Mcmctree, and CAFE 4.2[43] (lambda -l 0.002). The selection pressure of single-copy gene in each species were analyzed by the Branch model of CodeML[44] 4.7a module in PAML. LTR_FINDER and PS SCAN[45] softwares were applied to search for LTR sequences with scores greater than or equal to 6 points in the genome, and filtered repeated results in LTR_FINDER. The LTR flanking sequences were compared by MUSCLE[46], and the distance was calculated by DistMat software Kimura model with 7.3*10-9 molecular clock.

3 Results and discussion

3.1 Initial characterization of the *A.nanchuanensis* genome

A total of 128.71 gigabases (Gb) reads were generated by the Nanopore platform, and 123.38 Gb clean data were obtained after quality control. The reads average length reached 17.48 kb, the N50 reads length was 19.18 kb, and the total sequencing depth was about 160.34 ×. Clean data obtained by filtering out the low-quality data was 7,057,335 reads. Details were shown in Table 1. The second-generation Illumina sequencing obtained 51.76 Gb data, and the total sequencing depth was about 68.01 ×.

Then the total sequencing depth should be 228.35 ×.

3.2 Genome assembly and assembled completeness evaluation

Sequenced by Nanopore three-generation sequencing, corrected by Canu, assembled by SMARTdenovo and polished by Racon, Pilon software, 769.44 Mb total length genome sequences with 1087 Contig number, 2.09 Mb Contig N50, and 402 kb Contig N90 were eventually generated (Table 1). Through statistical alignment analysis of second-generation sequencing reads, clean reads located on the reference genome accounting for 99.41% of the total clean reads (363,371,475/365,545,724). Dual-ended sequencing sequences that located on the reference genome with the proper distance corresponding to the length distribution of the sequencing fragment accounted for 93.56% of the total clean Reads (341,995,184/365,545,724). The core gene integrity assessment is performed by CEGMA v2.59, the number of 458 CEG present in assembly accounted for 97.16% of all 458 CEGs (445/458), while 232 highly conserved CEG present in assembly accounted for 93.55% of all 248 CEGs (232/248). The database in BUSCO v4.0.6 contains 1,614 conserved core genes, and the number of complete genes present in assembly is 1583 (98.08%). The above data all suggest that genome assembly of the *A.nanchuanensis* work well.

3.3 Hybrid assembly, scaffolding, and chromosome anchoring

We obtained 137.5 Gb clean Hi-C data (about 62 × depth of the estimated genome). The clean Hi-C reads accounted for 179-fold coverage of 769.44Mb genome through Illumina platform for subsequent analysis. To assess the quality of Hi-C data, we performed an insertion fragments length assessment, which showed a relatively narrow unimodal length distribution with the highest peak around 300 bp, indicating that the dispersion degree of the inserted fragment length is small, the inserted fragment size is normal and the purification of Magnetic beads during library construction function efficient (Fig. 3). 728,487,984 pairs were genome-related mapping reads, accounting for 79.37% of the clean data. 236,274,160 Mb pairs were uniquely correlated to the genome, including 56,964,635 (24.11%) pairs valid Hi-C data. Details were shown in Table 2 and Table 3. Alignment efficiency, insert fragment length and effective Hi-C data volume evaluation all indicated that the Hi-C libraries constructed well.

After Hi-C assembly and manual adjustment, a total of 766.50 Mb genomic sequences were located on 28 chromosomes through scaffolds correction, clustered, ordered and orientated, accounting for 99.62% genomic sequences, the corresponding sequences number was 1,336 (97.95%); Among the sequences located on the

chromosome, the sequence length that could determine the order and direction was 697.71Mb, accounting for 91.02% of the total length of sequences on chromosomes (Table 4). Contig N50 and Scaffold N50 were 1.78Mb and 25.15Mb respectively after error correction (Table 5). Final pseudo-chromosomes were constructed after manually adjusted.

The genomes of *A.nanchuanensis* and *F. microcarpa* (*Ficus.microcarpa*) were compared to verify the accuracy of the 28 chromosomes overlapping arrangement group, the collinearity circle diagram indicates a high continuity between each other (Fig. 4). A heat map was drawn to evaluate the structure and quality of Hi-C assembly (Fig. 5), the figure indicated that the 28 pseudo-chromosomes could be distinguished easily and the interaction signal intensity at the diagonal is significantly stronger than at other locations within each pseudochromosome, suggesting that the genome assembly quality of *A.nanchuanensis* is high.

3.4 Repeat annotation, gene prediction and gene annotation

A total of 422.78 Mb (54.94%) repeat sequences was detected, among these repeat elements, long terminal repeats (LTR) was the predominant type, ClassI/LTR/Copia and ClassI/LTR/Gypsy respectively accounted for 19.17% (147.52 Mb) and 16.86% (129.74 Mb). The details of repeat sequence were shown in Table 6.

The 41,636 protein-coding genes were predicted with 3,797.54 bp average gene length, 1,509.16 bp average exon length, and 2,288.38 bp average intron length by *Ab initio*-based, homolog-based, and RNA-seq-based combine methods (Table 7, Table 8). Among the genes integrated by EVM, 27,262 genes were obtained by the three prediction methods, details were shown in Fig. 6. By GenBlastA v1.0.4 and GeneWise2.4.1, finally 1,905 pseudogenes were obtained, their total length and average length were 4,825,668 Kb and 2,533.16 Kb respectively.

39,596 genes were successfully annotated in the functional databases, accounting for 95.10% (39,596/41636) of the predicted genes, details were shown in Table 9. According to the non-coding RNA predicted results, the miRNAs number was 138, belonging to 24 RNA families; rRNAs was 409, belonging to 4 RNA families; and tRNA was 512, belonging to 24 families (Table 10).

The homologous gene of *Artocarpus nanchuanensis* and *morous notabilious* was 30510, accounting for 77.14%, based on Nr homologous species distribution, indicating the high homology (Fig.7). KOG database is based on the phylogenetic relationships of protein-coding bacteria, algae, and eukaryotes with complete genomes to classify the gene products in lineal homology and classify the genes in the

functional level. 21,567 (51.80%) *A.nanchuanensis* genes were annotated in the KOG database (Table 9), and the annotation classification details were shown in Fig. 8, the three dominant genes are mainly involved the function of general function prediction only, posttranslational modification, protein turnover, chaperones, and signal transduction mechanisms. KEGG is the main public database of Pathway, and through KEGG data retrieval annotation of predicted genes, 129 metabolic pathways of *A.nanchuanensis* were finally obtained. The GO database defined and described the genes and proteins. Through GO analysis, genes can be classified according to their involvement in biological processes, the components that make up cells, and the molecular functions they perform (Fig. 9).

3.5 Comparative genomics

The protein sequences between *A.nanchuanensis* and its related species (*A.thaliana*, *A.trichopoda*, *P.trichocarpa*, *A.chinensis*, *V.vinifera*, *M.notabilis*, *T.cacao*) were compared, and 33925 genes of the predicted 41,636 *A.nanchuanensis* genes were clustered into 15436 gene families, of which 512 were *A.nanchuanensis* unique gene family (Table 11 and Fig. 10). Five related species were clustered together in the phylogenetic tree, and the differentiation time between *A.nanchuanensis* and four other species was around 18.66 million years ago (Mya) by Mcmctree estimated (Fig. 11, Fig. 12). According to the species evolutionary relationship and the result of gene family clustering, 309 expanded gene families and 559 contracted gene families in *A.nanchuanensis* were detected comparing with related plant species (Fig. 13).

The functional annotation details of expanding and contracting gene families in the studied species were shown in Table 12, F-box domain, Cystatin domain, Protein kinase domain and Ring finger domain functions were involved. EVM0035972.1, EVM0031735.1, EVM0026117.1 and EVM0015119.1 were the rapidly evolving gene, details of rapid evolutionary genes and its annotated function were shown in Table 13 and Fig. 14. 4DTV is a quadruple degeneracy site, the third base of a codon encodes the same amino acid site no matter what nucleotide it is converted to. According to the homologous gene pairs between two species or between species and species themselves, the ratio of each homologous gene to 4DTV mutation site was calculated and 4DTV distribution map was made (Fig. 15). The details of LTR insertion time were shown in Fig. 16.

4. Conclusion

The high-quality genome assembly and annotation information for

A.nanchuanensis were firstly reported, that was also the first reference genome of the Artocarpus genus. 123.38 Gb clean reads were obtained and 769.44 Mb genome was assembled, that was larger than the sequenced mulberry and Paper Mulberry species. With the help of Oxford Nanopore technology, the contig N50 of the assembled genome achieved 2.09 Mb, and the longest contig was 8.88 Mb. The high-coverage Nanopore sequencing and Illumina data polishing composite strategy effectively produced the highly contiguous genome assembly. The contigs were clustered and ordered onto 28 pseudo-chromosomes with Hi-C data. 41,636 protein-coding genes were predicted and 95.10% genes were annotated. This high quality genome of *A.nanchuanensis* will lay a solid foundation for the conservation and development of the critically endangered species in the future.

Acknowledgements

This work was supported by Key Laboratory of Bio-Resources and Eco-Environment of Ministry of Education, College of Life Sciences, Sichuan University, Chengdu 610065, Sichuan, P.R.China. and Chongqing Nanchuan biotechnology research institute, Bio-resource Research and Utilization Joint Key Laboratory of Sichuan and Chongqing, Sichuan and Chongqing, P.R.China.

References

1. Ｒong-, L. I. U. Studies on Chemical Constituents Occurring in Twigs of Artocarpus nanchuanensis. 2–6 (2013).
2. Ren, G. *et al.* Chemical constituents from the fruiting branches of Artocarpus nanchuanensis endemic to China. *Biochem. Syst. Ecol.* **51**, 98–100 (2013).
3. He, N. *et al.* Draft genome sequence of the mulberry tree Morus notabilis. (2013). doi:10.1038/ncomms3445
4. Peng, X. *et al.* A Chromosome-Scale Genome Assembly of Paper Mulberry (Broussonetia papyrifera) Provides New Insights into Its Forage and Papermaking Usage. *Mol. Plant* **12**, 661–677
5. Bian, L. *et al.* Chromosome-level genome assembly of the greenfin horse-faced filefish (Thamnaconus septentrionalis) using Oxford Nanopore PromethION sequencing and Hi-C technology . *Mol. Ecol. Resour.* 1–25 (2020). doi:10.1111/1755-0998.13183
6. Rao, S. S. P., Huntley, M. H., Durand, N. C. & Stamenova, E. K. Article A 3D Map of the Human Genome at Kilobase Resolution Reveals Principles of Chromatin Looping. *Cell* 1–16 (2014). doi:10.1016/j.cell.2014.11.021
7. Koren, S. *et al.* Canu : scalable and accurate long- - - read assembly via adaptive k - - - mer weighting and repeat separation. 1–36
8. Vaser, R., Sovi, I., Nagarajan, N. & Šiki, M. Fast and accurate de novo genome assembly from long uncorrected reads.
9. Walker, B. J. *et al.* Pilon : An Integrated Tool for Comprehensive Microbial Variant Detection and Genome Assembly Improvement. **9**, (2014).
10. Li, H. & Durbin, R. Fast and accurate short read alignment with Burrows – Wheeler transform. **25**, 1754–1760 (2009).
11. Parra, G., Bradnam, K. & Korf, I. Genome analysis CEGMA : a pipeline to

accurately annotate core genes in eukaryotic genomes. **23**, 1061–1067 (2007).
12. Simão, F. A., Waterhouse, R. M., Ioannidis, P. & Kriventseva, E. V. BUSCO : assessing genome assembly and annotation complete- ness with single-copy orthologs. 9–10 (2015).
13. Servant, N. *et al.* HiC-Pro : an optimized and flexible pipeline for Hi-C data processing. 1–11 (2015). doi:10.1186/s13059-015-0831-x
14. Burton, J. N. *et al.* Chromosome-scale scaffolding of de novo genome assemblies based on chromatin interactions. (2013). doi:10.1038/nbt.2727
15. Xu, Z. & Wang, H. LTR _ FINDER : an efficient tool for the prediction of full-length LTR retrotransposons. **35**, 265–268 (2007).
16. Price, A. L., Jones, N. C. & Pevzner, P. A. De novo identification of repeat families in large genomes. **21**, 351–358 (2005).
17. Jurka, J. *et al.* Diversity of Retrotransposable Elements Repbase Update , a database of eukaryotic repetitive elements. **467**, 462–467 (2005).
18. Tarailo-graovac, M. & Chen, N. Using RepeatMasker to Identify Repetitive Elements in Genomic Sequences. 1–14 (2009). doi:10.1002/0471250953.bi0410s25
19. Burge, C. & Karlin, S. Prediction of Complete Gene Structures in Human Genomic DNA. 78–94 (1997).
20. Stanke, M. & Waack, S. Gene prediction with a hidden Markov model and a new intron submodel. **19**, 215–225 (2003).
21. Majoros, W. H., Pertea, M. & Salzberg, S. L. TigrScan and GlimmerHMM : two open source ab initio eukaryotic gene-finders. **20**, 2878–2879 (2004).
22. Blanco, E., Parra, G. & Guigó, R. Using geneid to Identify Genes. *Curr. Protoc. Bioinforma.* 1–28 (2007). doi:10.1002/0471250953.bi0403s18
23. Korf, I. Gene finding in novel genomes. **9**, 1–9 (2004).
24. Keilwagen, J. *et al.* Using intron position conservation for homology-based gene prediction. 1–11 (2016). doi:10.1093/nar/gkw092
25. Keilwagen, J., Hartung, F., Paulini, M., Twardziok, S. O. & Grau, J. Combining RNA-seq data and homology-based gene prediction for plants , animals and fungi. (2018).
26. Kim, D., Langmead, B. & Salzberg, S. L. HISAT : a fast spliced aligner with low memory requirements. *Nat. Methods* (2015). doi:10.1038/nmeth.3317
27. Pertea, M. *et al.* StringTie enables improved reconstruction of a transcriptome from RNA-seq reads. (2015). doi:10.1038/nbt.3122
28. Tang, S., Lomsadze, A., Borodovsky, M. & Tech, J. G. Identification of protein coding regions in RNA transcripts. **43**, 1–10 (2015).
29. Campbell, M. A., Haas, B. J., Hamilton, J. P., Mount, S. M. & Buell, C. R. Comprehensive analysis of alternative splicing in rice and comparative analyses with Arabidopsis. **17**, 1–17 (2006).
30. Haas, B. J. *et al.* Open Access Automated eukaryotic gene structure annotation using EVidenceModeler and the Program to Assemble Spliced. **9**, 1–22 (2008).
31. Griffiths-jones, S. *et al.* Rfam : annotating non-coding RNAs in complete genomes. **33**, 121–124 (2005).
32. Lowe, T. M. & Eddy, S. R. tRNAscan-SE : a program for improved detection of transfer RNA genes in genomic sequence. **25**, 955–964 (1997).
33. She, R., Chu, J. S., Wang, K., Pei, J. & Chen, N. genBlastA : Enabling BLAST to identify homologous gene sequences. 143–149 (2009). doi:10.1101/gr.082081.108.4
34. Birney, E., Clamp, M. & Durbin, R. GeneWise and Genomewise. 988–995 (2004). doi:10.1101/gr.1865504.quickly
35. Marchler-bauer, A. *et al.* CDD : a Conserved Domain Database for the functional annotation of proteins. **39**, 225–229 (2011).
36. Koonin, E. V *et al.* A comprehensive evolutionary classification of proteins encoded in complete eukaryotic genomes. **5**, (2004).
37. Dimmer, E. C. *et al.* The UniProt-GO Annotation database in 2011. **40**, 565–570 (2012).
38. Kanehisa, M. & Goto, S. KEGG : Kyoto Encyclopedia of Genes and Genomes.

28, 27–30 (2000).
39. Boeckmann, B. *et al.* The SWISS-PROT protein knowledgebase and its supplement TrEMBL in 2003. **31**, 365–370 (2003).
40. Altschup, S. F., Gish, W., Pennsylvania, T. & Park, U. Basic Local Alignment Search Tool 2Department of Computer Science. 403–410 (1990).
41. Li, L. *et al.* OrthoMCL : Identification of Ortholog Groups for Eukaryotic Genomes OrthoMCL : Identification of Ortholog Groups for Eukaryotic Genomes. 2178–2189 (2003). doi:10.1101/gr.1224503
42. Uindon, P. G. & Ranc, J. E. A. N. New Algorithms and Methods to Estimate Maximum-Likelihood Phylogenies : Assessing the Performance of PhyML 3 . 0. **59**, 307–321 (2010).
43. Bie, T. De, Cristianini, N., Demuth, J. P. & Hahn, M. W. CAFE : a computational tool for the study of gene family evolution. **22**, 1269–1271 (2006).
44. Schabauer, H., Valle, M., Pacher, C. & Stockinger, H. SlimCodeML : An Optimized Version of CodeML for the Branch-Site Model. (2012). doi:10.1109/IPDPSW.2012.88
45. Prestridge, D. S. SIGNAL SCAN : a computer program that scans DNA sequences for eukaryotic transcriptional elements. **7**, 203–206 (1991).
46. Edgar, R. C., Drive, R. M. & Valley, M. MUSCLE : multiple sequence alignment with high accuracy and high throughput. **32**, 1792–1797 (2004).

Data Accessibility

The whole raw sequence reads produced by Illumina novaseq, Pacbio sequel II and ONT PromethlON Beta, have been deposited at NCBI Sequence Read Archive (SRA) under BioProject number PRJNA624965 and BioSample from SAMN14589993 for A.nanchuanensis. Raw sequencing data (Nanopore, Illumina, Hi-C, RNA-seq data) have been deposited in SRA database as SRR11671532, SRR11659666, SRR11659674, SRR11623450/SRR11668249.

Author contributions

J.H., S.B., X.D., K.K. and C.C. conceived and designed the study; J.H., S.B., X.D., J.D. X.W. and Q.L. collected the samples; Q.L., Z.S. and Y.L. performed DNA sequencing and Hi-C experiments; Y.C. and L.F. performed RNA sequencing; J.H., Q.L. and Z.S. estimated the genome size, assembled the genome, and assessed the assembly quality; Y.C. and L.F. performed the genome annotation and functional genomic analysis. S.X., J.H. and X.D. wrote the manuscript. All authors read, edited, and approved the final manuscript for submission.

Competing interests

The authors declare no competing interests.

Table Click here to access/download;Table;table .pdf

Table 1 Statistics of the Nanopore three-generation sequencing and genome assembly of Artocarpus Nanchuanensis.

Nanopore three-generation sequencing results		Statistics on assembly results	
SumBase	123,375,925,044	Contig length	769,440,982 bp
MaxLen	216,661 bp	Contig max	8,879,419 bp
SeqNum	7,057,335	Contig number	1,087
N50Len	19,177 bp	Contig N50	2,094,024 bp
N90Len	11,029 bp	Contig N90	402,757 bp

Note: SumBase means total base number of sequencing data; MaxLen means the longest reads length of sequencing data; SeqNum means the total read number of sequencing data; N50Len means the N50 length of sequencing data reads; N90Len means the N90 length of sequencing data reads; Contig length means the length of Contig in the middle of more than 1Kb of scaffolding; Contig Max means the length of the longest Contig; Contig Number means the Number of Contig in the middle of more than 1Kb scaffolding; Contig N50 means the length of Contig N50; Contig N90 means the length of Contig N90.

Table 2 Statistical table of Clean Data and genome comparison results.

**Library**	Mapping Type	Number	Ratio （%）
	Total Read Pairs	458,907,479	100
	Mapped Reads	728,487,984	79.37
	Unique Mapped Read Pairs	236,274,160	51.45

Table 3 Statistical table of Hi-C sequencing data types.

Library	Type	Number	Ratio (%)
	Unique Paired Alignments	236,274,160	100
	Valid Interaction Pairs	56,964,635	24.11
	Dangling End Pairs	138,663,715	58.69
	Re-ligation Pairs	15,169,250	6.42
	Self-cycle Pairs	975,544	0.41
	Dumped Pairs	24,501,016	10.37

Note: Unique Paired Alignments: The Unique Read Pairs alignment to the genome; Valid Interaction Pairs: the Valid Interaction Read Pairs; Dangling End Pairs: the Dangling End read pairs in the invalid data; Re-ligation Pairs: the re-ligation Read Pairs in the invalid data; Self-circle Ligation Pairs: Self-circle Ligation Read Pairs in the invalid data; Dumped Pairs: the dumped Read Pairs in the invalid data.

Table 4 The statistics table of Hi-C assembly data.

Group	Cluster Num	Cluster Len (bp)	Order Num	Order Len (bp)
LG01	46	26,514,107	24	24,676,255
LG02	41	26,638,661	16	24,489,134
LG03	30	24,254,703	16	23,044,270
LG04	34	22,404,888	13	20,644,200
LG05	33	21,646,681	16	20,177,649
LG06	35	29,133,579	18	27,822,153
LG07	69	32,924,820	27	29,467,719
LG08	45	29,858,101	20	27,605,363
LG09	77	29,556,483	29	25,185,028
LG10	45	22,896,788	20	20,243,522
LG11	67	25,833,105	20	21,750,724
LG12	37	24,385,337	15	22,370,729
LG13	47	23,481,896	24	21,098,278
LG14	46	29,162,015	19	26,857,340
LG15	61	28,431,484	30	25,341,045
LG16	32	21,965,556	16	20,879,538
LG17	41	25,915,114	19	24,032,910
LG18	49	34,941,454	27	32,502,827
LG19	54	29,520,137	21	25,685,935
LG20	50	32,513,478	18	29,815,261
LG21	50	28,639,915	21	25,613,043
LG22	42	27,392,871	24	25,873,084
LG23	42	28,655,389	16	26,447,344
LG24	52	27,753,222	24	25,148,606
LG25	46	23,720,417	16	21,152,151
LG26	63	33,995,937	28	30,220,329
LG27	58	28,458,315	24	25,577,647
LG28	44	25,907,258	22	23,985,053
Total (Ratio%)	1336 (97.95%)	766501711 (99.62%)	583 (43.64%)	697707137 (91.02%)

Note: the statistics do not include 100 Ns added by artificially connected pseudochromosomes.

Table 5 The genomic statistics after Hi-C assembly.

Scaffold number	Scaffold length (bp)	Scaffold N50 (bp)	Scaffold N90 (bp)	Scaffold max (bp)	Gap total length (bp)
809	769,496,482	25,150,906	20,179,149	32,505,427	55,500
Contig number	Contig length (bp)	Contig N50 (bp)	Contig N90 (bp)	Contig max (bp)	GC content (%)
1,364	769,440,982	1,778,064	200,000	8,646,128	32.34

Note: Scaffold represents the Scaffold generated after Hi-C connection; Contig represents the Contig after Hi-C error corrected. Scaffold number: the number of scaffolds above 1 Kb; Scaffold length (bp): the length of a Scaffold exceeding 1 Kb; Scaffold N50 (bp): length of Scaffold N50 above 1 Kb; Scaffold N90 (bp): length of Scaffold N90 above 1 Kb; Scaffold Max (bp): the length of the longest Scaffold exceeding 1 Kb; Gap total length (bp): the total length of Gap; Contig Number: the Number of Contig in the scaffold above 1 Kb; Contig length (bp): the length of Contig in the middle of the scaffold above 1 Kb; Contig N50 (bp): the length of Contig N50; Contig N90 (bp): the length of Contig N90; Contig Max (bp): the length of the longest Contig; GC content (%): the GC content percentage.

Table 6 The statistics of repeat sequences.

Type	Number	Length	Rate (%)
ClassI	715,880	354,430,815	46.06
ClassI/DIRS	26,959	23,880,069	3.10
ClassI/LARD	238,559	75,579,627	9.82
ClassI/LINE	12,327	3,488,322	0.45
ClassI/LTR/Copia	210,964	147,520,139	19.17
ClassI/LTR/Gypsy	209,038	129,742,980	16.86
ClassI/LTR/Unknown	3,210	597,880	0.08
ClassI/PLE	1,322	456,964	0.06
ClassI/SINE	8,910	2,272,141	0.30
ClassI/TRIM	4,219	1,617,309	0.21
ClassI/Unknown	372	66,025	0.01
ClassII	103,050	33,806,173	4.39
ClassII/Crypton	17	1,003	0.00
ClassII/Helitron	22,320	5,961,597	0.77
ClassII/MITE	1,183	204,923	0.03
ClassII/Maverick	1,392	1,762,270	0.23
ClassII/TIR	66,336	25,334,820	3.29
ClassII/Unknown	11,802	879,933	0.11
PotentialHostGene	30,328	8,227,684	1.07
SSR	10,909	2,628,783	0.34
Unknown	187,171	55,237,782	7.18
Total	1,047,338	422,782,698	54.94

Note: Type: repeat sequence Type; Number: The Number of repeats that obtained; Length: the total Length of the predicted repeating sequence; Rate (%): the proportion of repetitive sequences in the total genome.

Table 7 The gene prediction results statistics.

Method	Software	Species	Gene number
Ab initio	Genscan	-	42,670
	Augustus	-	47,491
	GlimmerHMM	-	74,635
	GeneID	-	70,861
	SNAP	-	88,742
Homology-based	GeMoMa	Arabidopsis_thaliana	33,745
		Morus_notabilis	41,079
		Prunus_avium	37,127
		Rosa_chinensis	37,535
RNAseq	TransDecoder	-	84,498
	GeneMarkS-T	-	52,056
	PASA	-	48,695
Integration	EVM	-	41,636

Table 8 The statistical table of genetic information.

GeneNum	Genelen	AveGenlen	ExonLen	AveExonLen
41,636	158,114,419	3,797.54	62,835,343	1,509.16
ExonNum	AveExonNum	CDSLen	AveCDSlen	CDSNum
233,559	5.61	50,445,441	1,211.58	226,727
AveCDSNum	IntronLen	AveIntronLen	IntronNum	AveIntronnum
5.45	95,279,076	2,288.38	191,923	4.61

Note: Gene Num: the number of predicted gene; GeneLen (bp): the total number of gene; AveGeneLen(bp): the average length of gene; ExonLen (bp): the total number of exons; AveExonLen (bp): the average of the total exon length of each gene; ExonNum: the number of exons; AveExonNum: he average number of exons per gene; CDSLen (bp): total length of CDS ; AveCDSLen (bp): average CDS length; IntronLen (bp): total intron length; AveIntronLen (bp): the total intron mean length for each gene; IntronNum: the number of introns; AveIntronnum: the introns average number in per gene.

Table 9 The gene function annotated statistics.

Annotation database	Annotated number	Percentage (%)
GO_Annotation	21,275	51.10
KEGG_Annotation	13,903	33.39
KOG_Annotation	21,567	51.80
TrEMBL_Annotation	39,571	95.04
nr_Annotation	39,553	95.00
All_Annotated	39,596	95.10

Note: Annotation database: Functional annotation database; Annotated number: the number of genes Annotated to the corresponding database; Percentage (%): The percentage of genes annotated to the total number of databases.

Table 10 The statistical results of non-coding RNA.

RNA classification	Number	Family
miRNA	138	24
rRNA	409	4
tRNA	512	24

Note: RNA classification: The classification of RNA; Number: The predicted RNA number; Family: The RNA family number.

Table 11 Statistical classification of gene families.

Name	Total gene	Cluster num	Total family	Unifamily
A.thaliana	27,369	23,106	12,753	726
A.trichopoda	16,986	15,058	11,147	254
P.trichocarpa	41,335	33,270	14,725	950
A.chinensis	39,040	25,888	12,648	1,327
V.vinifera	26,346	19,238	12,682	665
M.notabilis	26,965	20,423	14,794	524
T.cacao	21,432	20,070	13,810	176
A. Nanchuanensis	41,636	33,925	15,436	512

Note: Name: the name of Species; Total gene: the number of total gene; Cluster num: the number of genes that involved in family classification; Total family number: the number of gene families that can be divided; Uni family: the number of unique gene families.

Table 12 The annotation of protein gene family.

GeneFamily	Pfam	Function
GF_12673	PF00646.28	F-box domain
GF_10548	PF00031.16	Cystatin domain
GF_8	PF00069.20	Protein kinase domain
GF_13176	PF13639.1	Ring finger domain

Note: Gene family : the gene family cluster; Pfam: the ID of protein family alignment to the Pfam database； Function： the function of the protein family that can be alignmented.

Table 13 The rapidly evolving genes selected by CodeML.

GeneID	P-value	Sites
EVM0035972.1	0.05	298,G,0.993**
EVM0031735.1	0.06	74,E,0.984*
EVM0026117.1	0.35	68,K,0.997**
EVM0015119.1	0.00	232,E,0.990**

Note: Gene ID mean the ID of gene, ω0 mean ka/ks for the studied Species, ω1 mean the average ka/ks for other species, ω2 mean ka/ks for the whole evolutionary tree.

Click here to access/download;Figure;figure 10-23.pdf

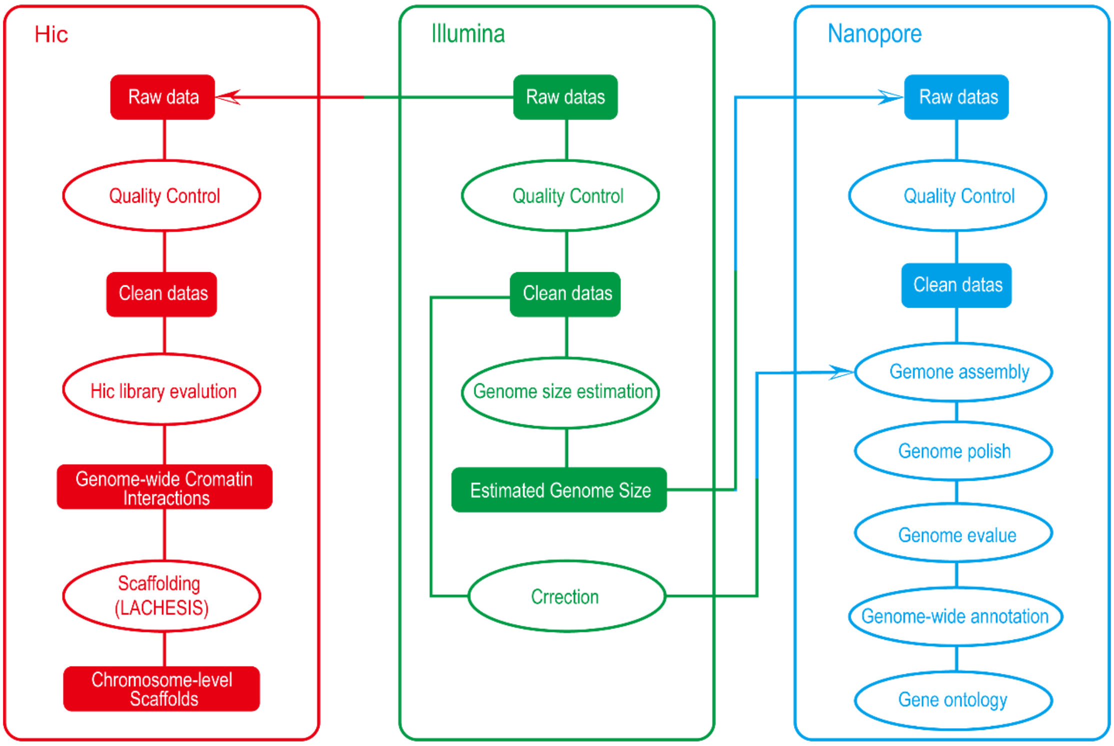

Fig. 1 The flowchart of A.nanchuanensis genome assembly and annotation process.

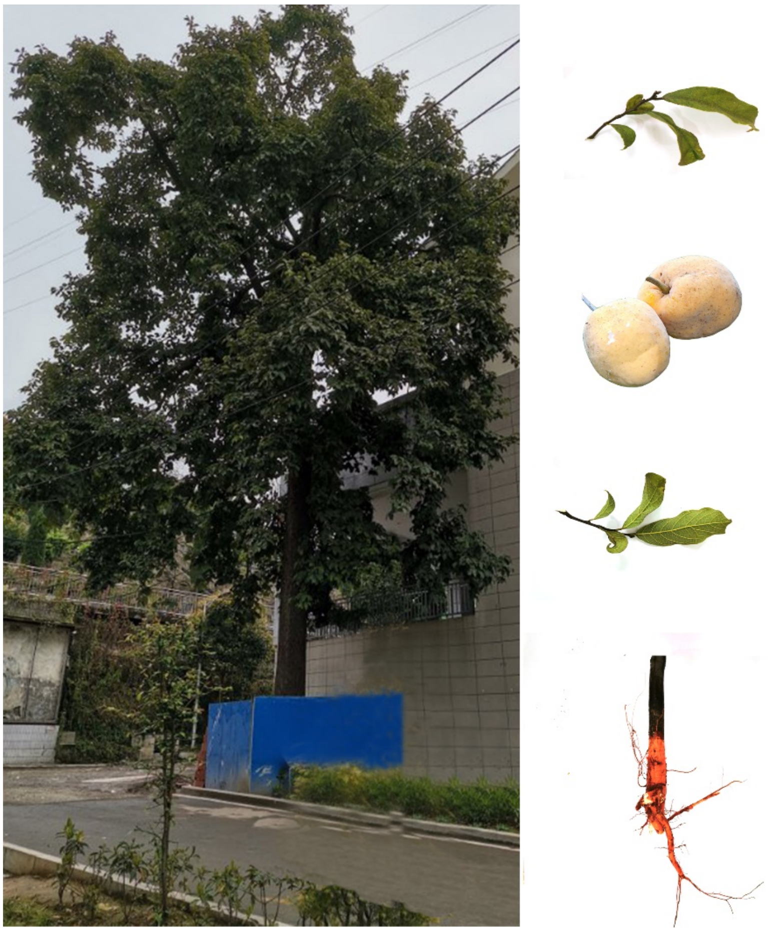

Fig. 2 The A.nanchuanensis tree used in this study.

Note: This picture was taken in 2019.

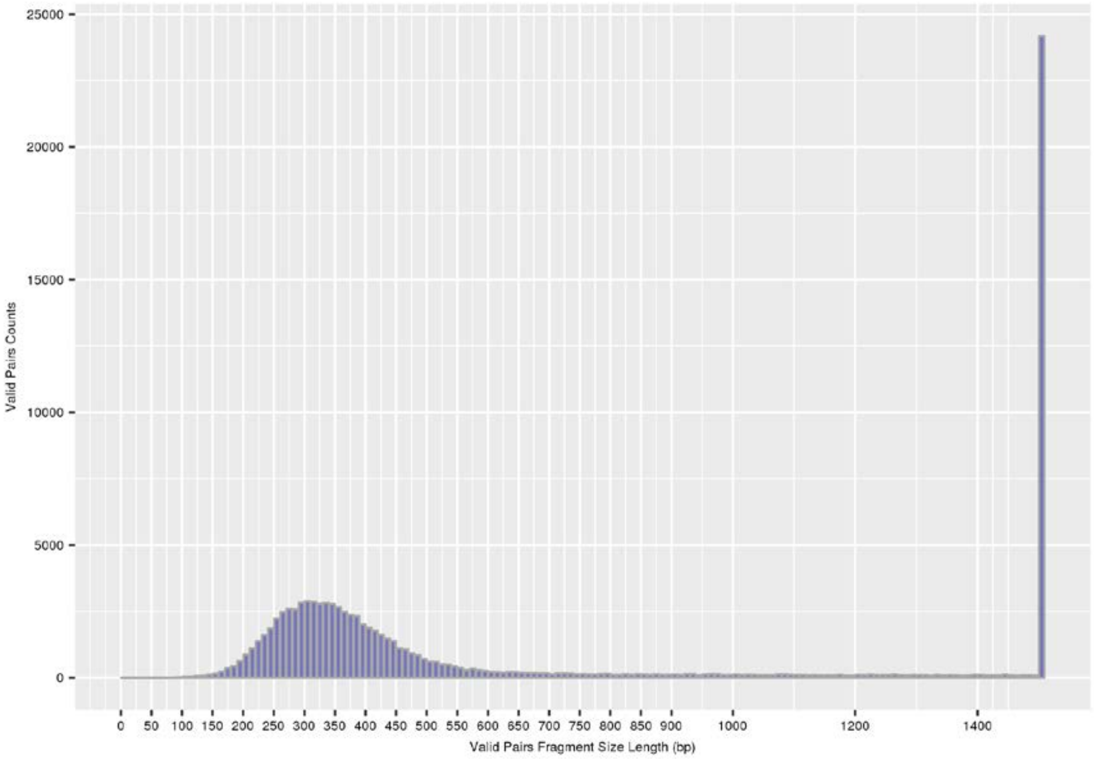

Fig. 3 The length distribution of library insert fragment.

Note: The abscissa axis is the sum of the distance between double ends Reads on the assembly genome and the nearest enzyme-cutting site. The vertical coordinate is the number of random extracted 100,000 pairs of Reads corresponding to the insert fragment of different length.

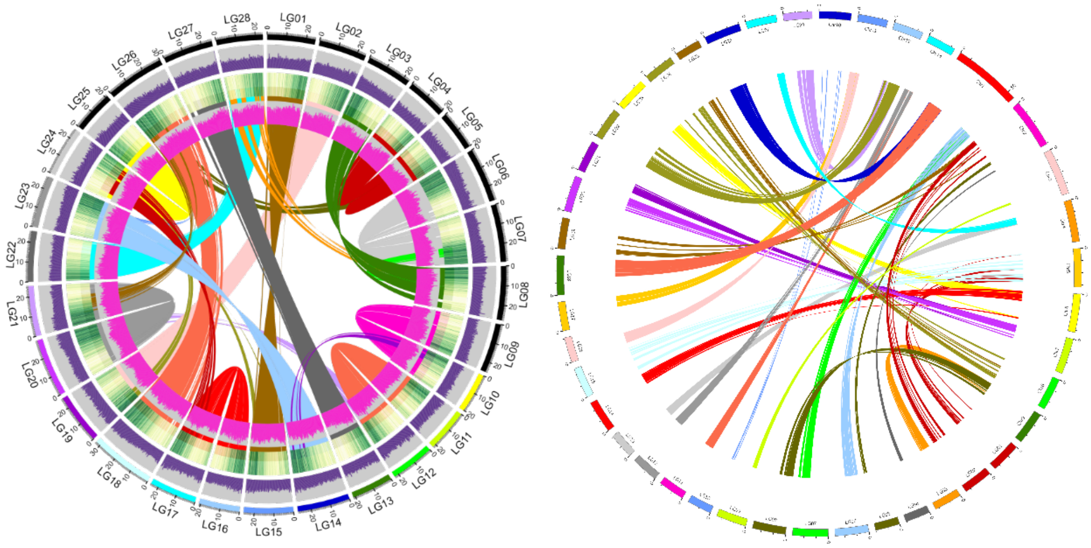

a b

Fig. 4 Circos plot of the reference genome of A. Nanchuanensis and syntenic relationship with the Ficus_microcarpa genome.

Note: (a) Circos plot of A. Nanchuanensis 28 chromosomes. The tracks from outside to inutside are 28 chromosome-level scaffolds, the GC content of chromosomes (purple), the gene density of chromosomes (green), the TE ratio of chromosomes (pink), and the lines of different colors in the innermost circle represent the collinearity within themselves. (b) Genes collinearity circle of A. Nanchuanensis and F.microcarpa. Each coloured represents a collinear block of each chromosome, with at least five collinearity genes in each block.

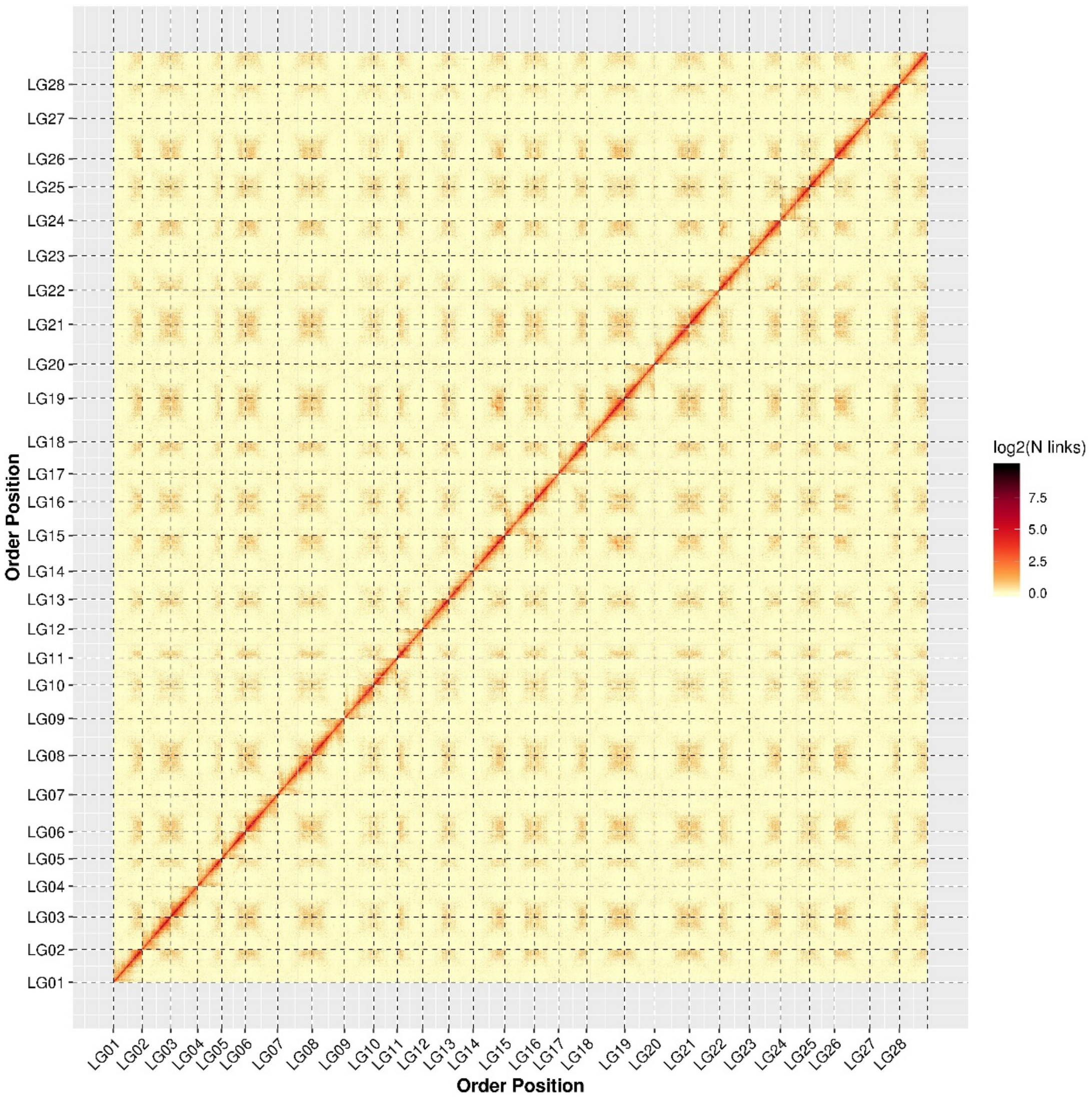

Fig. 5 The interaction heat map of Hi-C assembly chromosome.

Note: The LG01-LG28 represents Lachesis group 01-28; The abscissa axis and vertical coordinates represent the Order of each bin on the corresponding chromosome group.

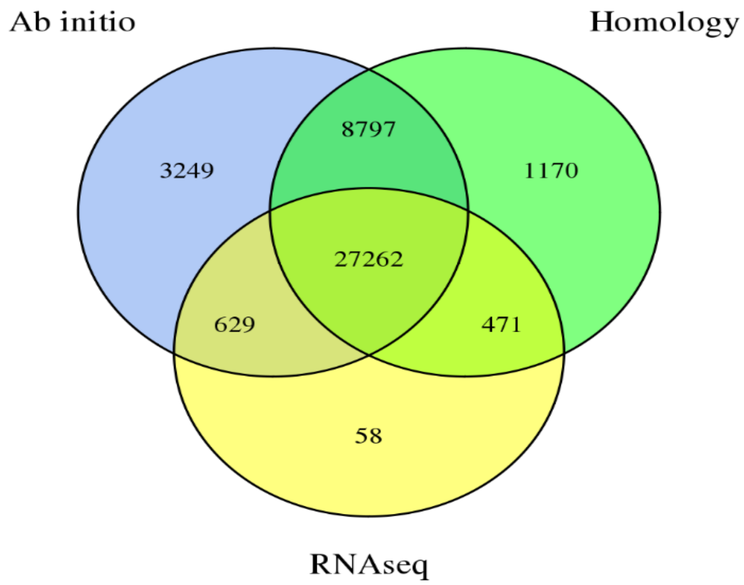

Fig. 6 The integrated genes were derived from the distribution of the three prediction methods.

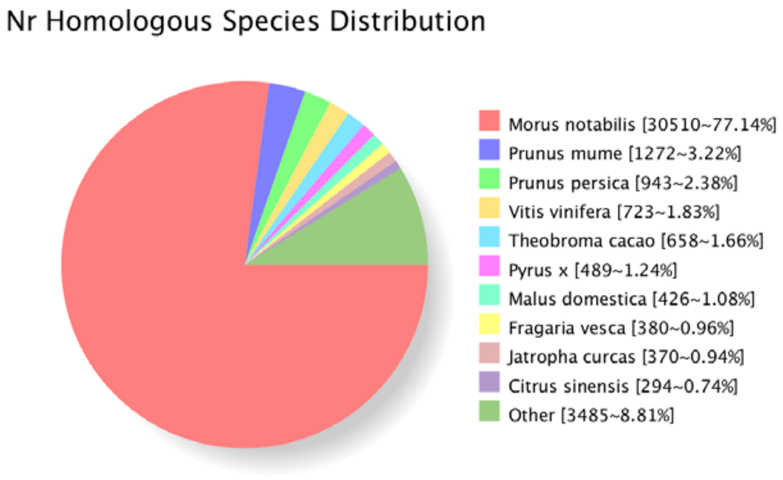

Fig. 7 The Nr homologous species distribution.

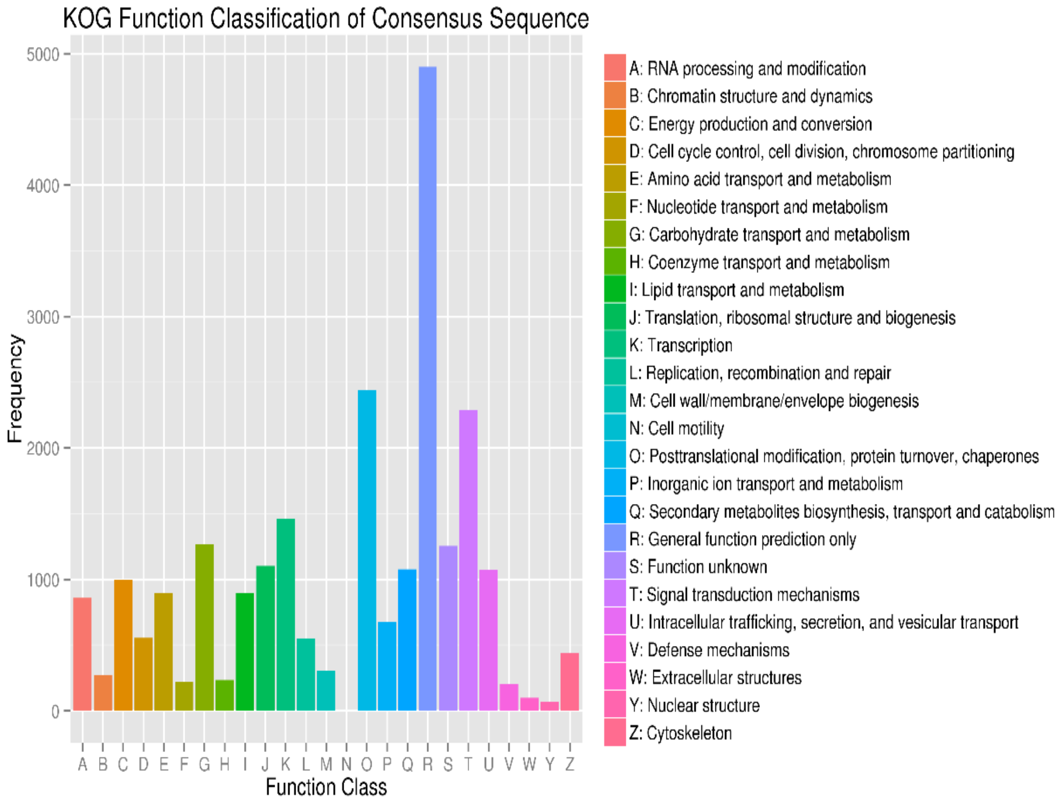

Fig. 8 The statistical graph of KOG functional annotation classification.

Note: The abscissa axis is the contents of each KOG classification, and the vertical coordinate is the number of genes.

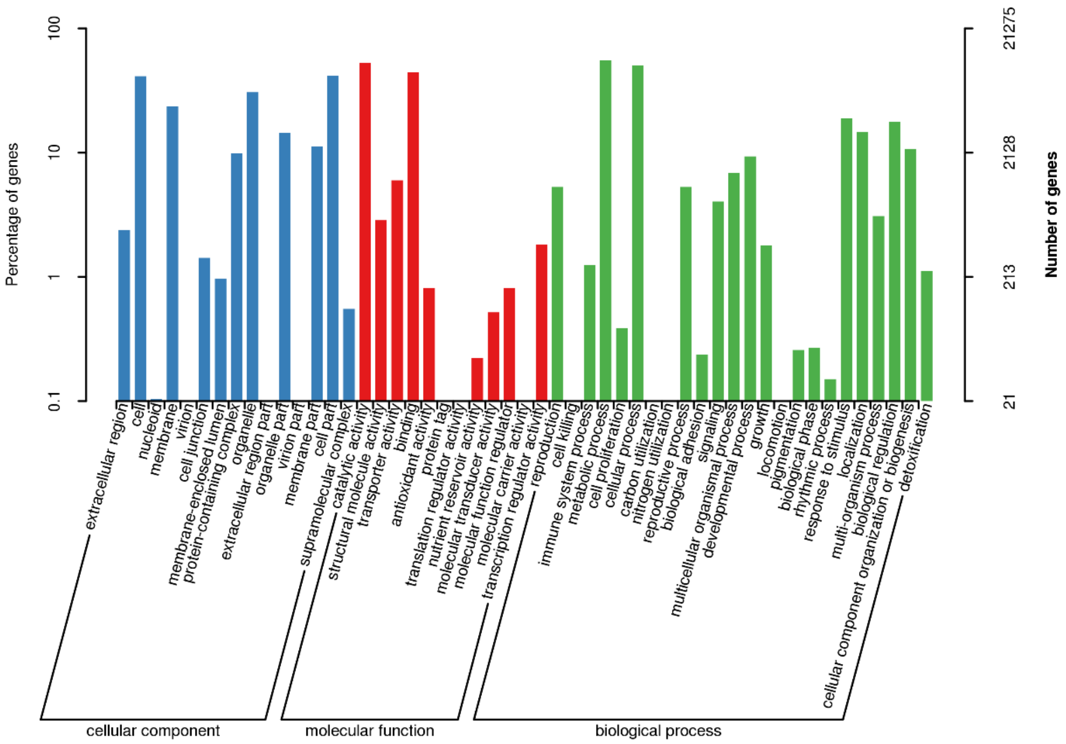

Fig. 9 The GO secondary node annotation classification statistics diagram.

Note: The abscissa axis is the contents of each category of GO; The vertical coordinates mean the percentage of the number of genes; And the right side is the number of genes. This figure shows the enrichment of each secondary function of gene GO.

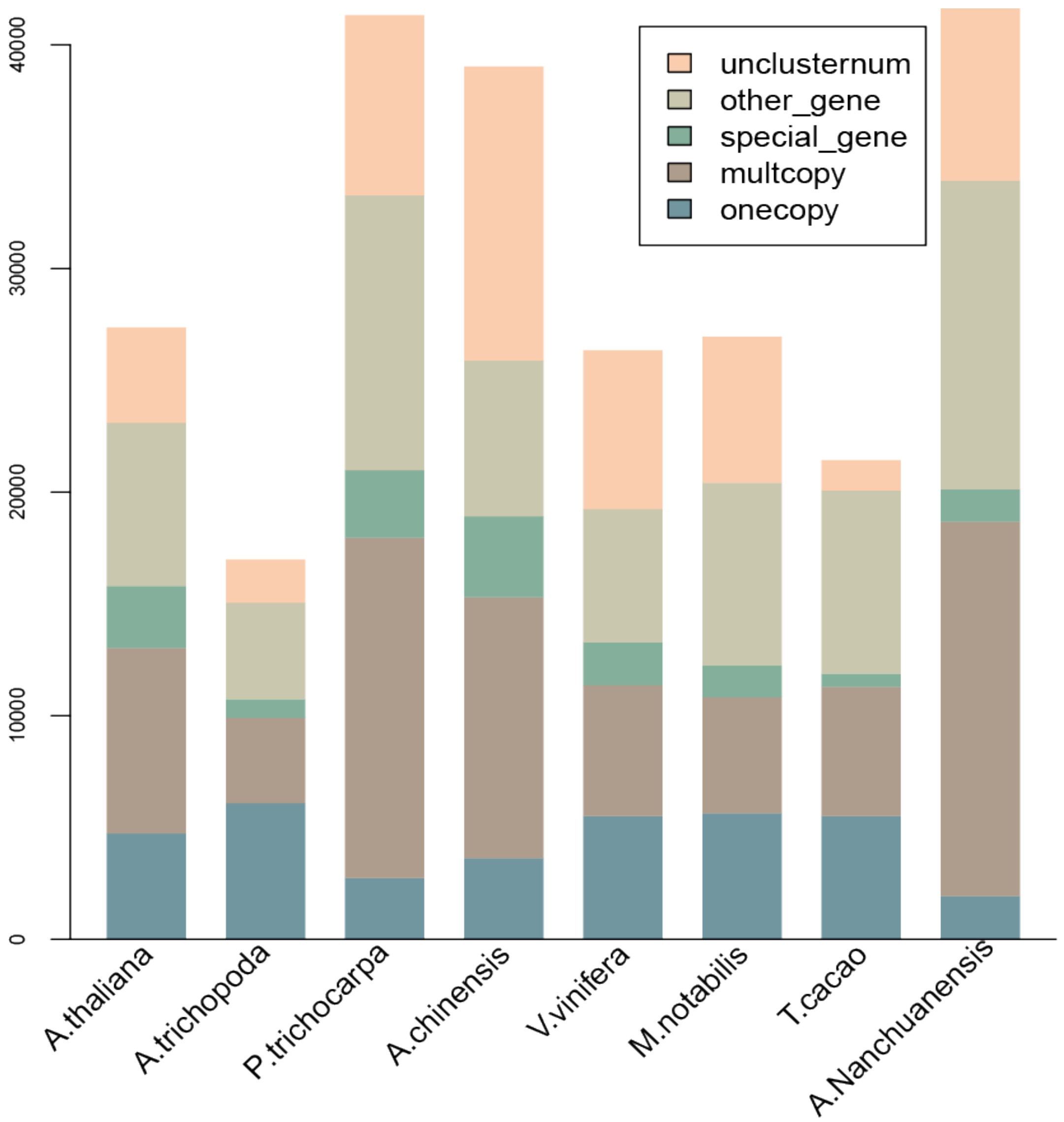

Fig. 10 The histogram of family clustering statistics.

Note: The abscissa axis mean the species name; The vertical coordinates mean the number of genes; Unclusternum mean the gene of unclustered to any family; Other gene mean all other genes; Special gene: the species specific genes in the family; Multcopy: the number of multiple copies of homologous genes in a common gene family; Onecopy: the number of single-copy homologous genes in the family of genes shared by species.

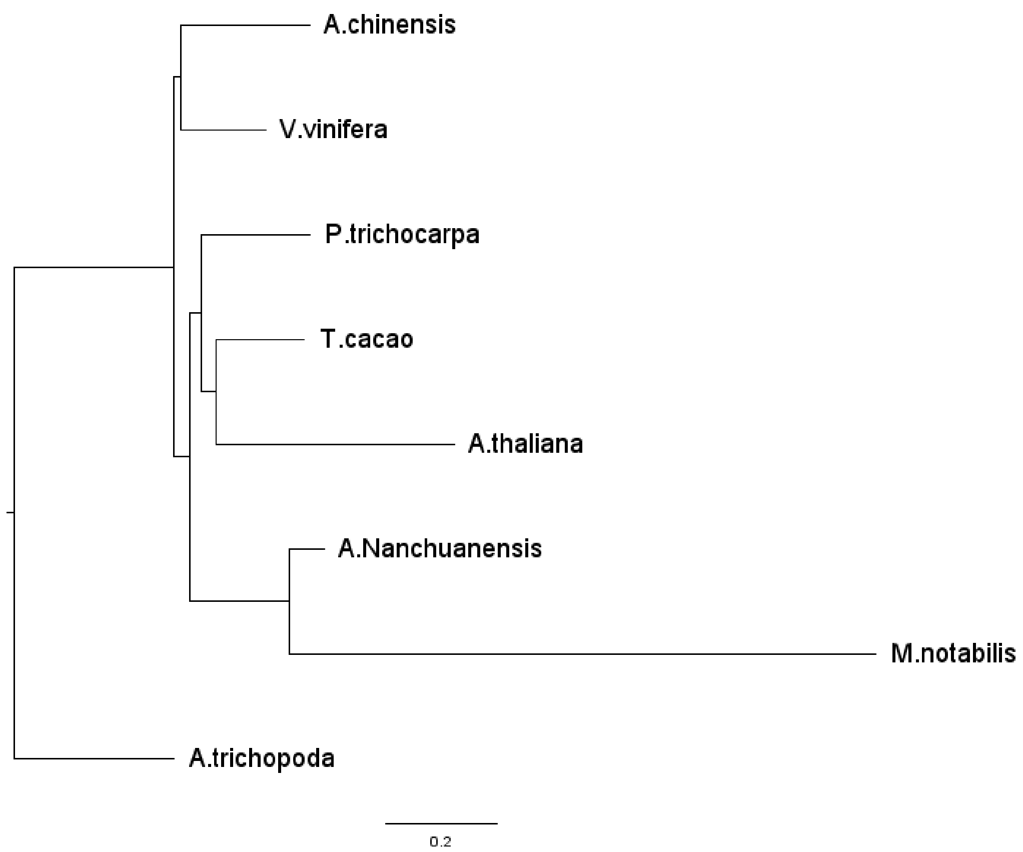

Fig. 11 The evolutionary relationships among species.

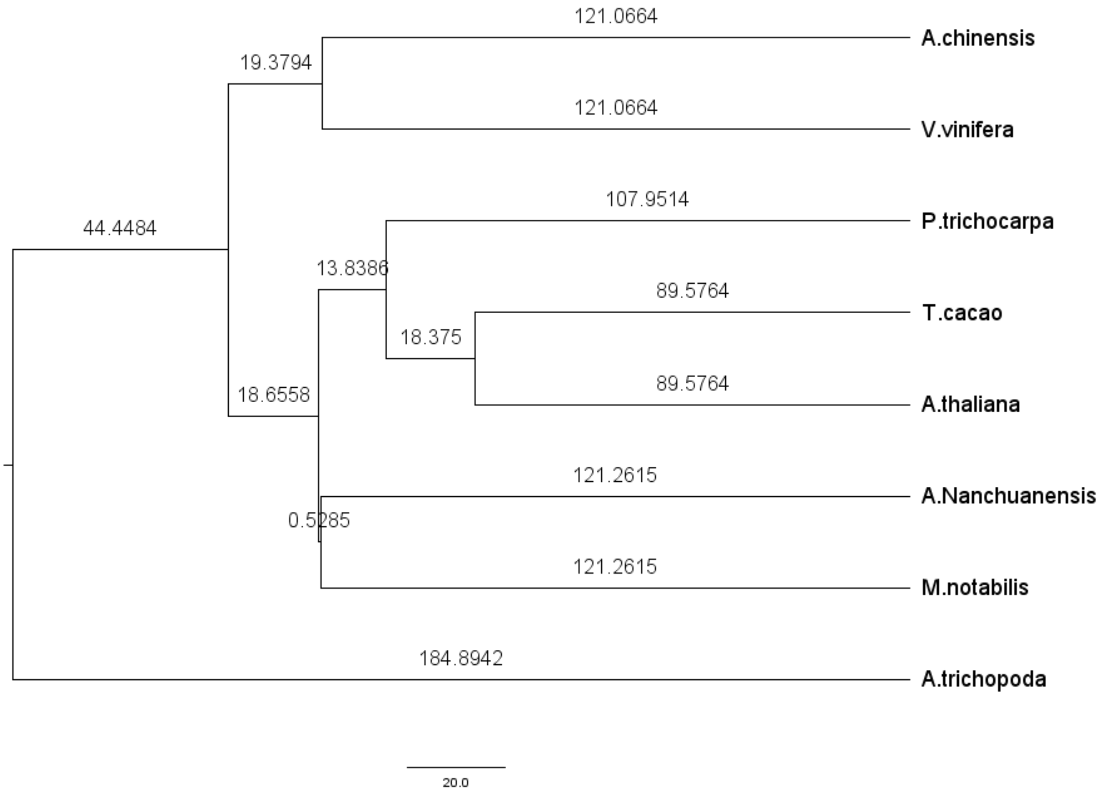

Fig. 12 The temporal relationship of species differentiation (unit: million years).

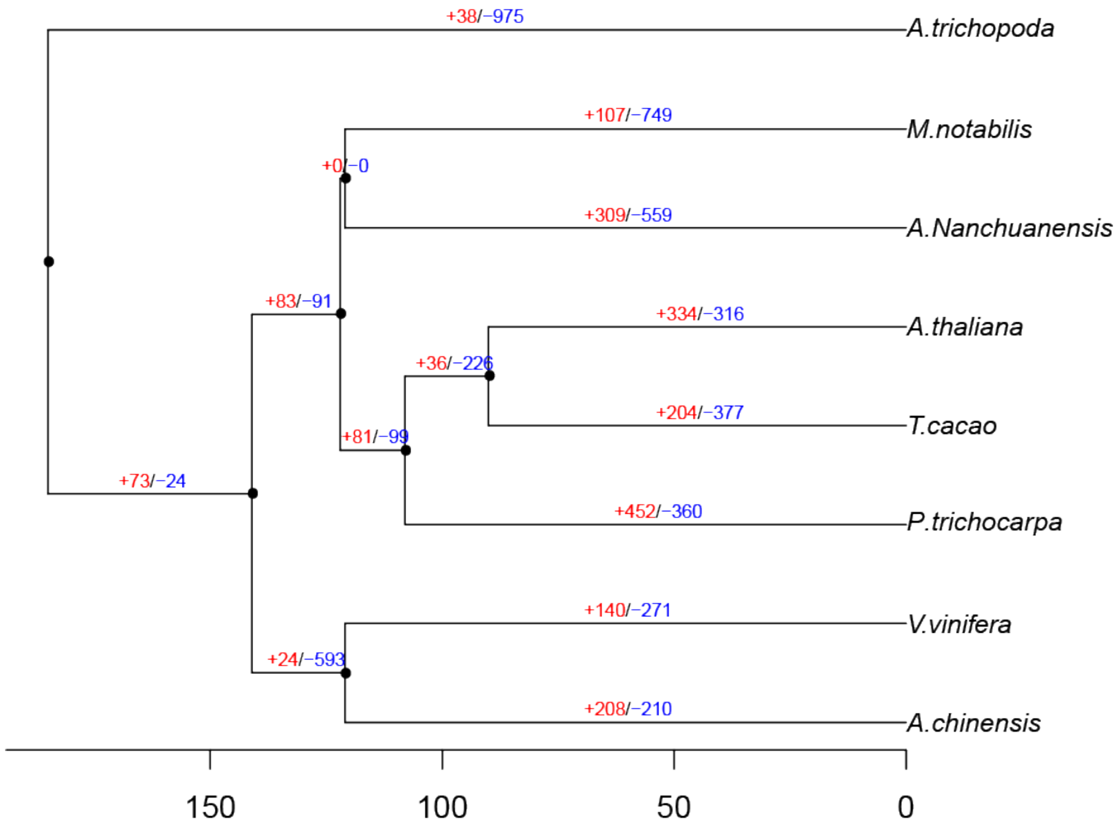

Fig. 13 The contraction and expansion of gene family.

Note: "+" represents the number of gene families expanding on this node, and "-" represents the number of gene families contracting on this node. The black dot refers to the common ancestor.

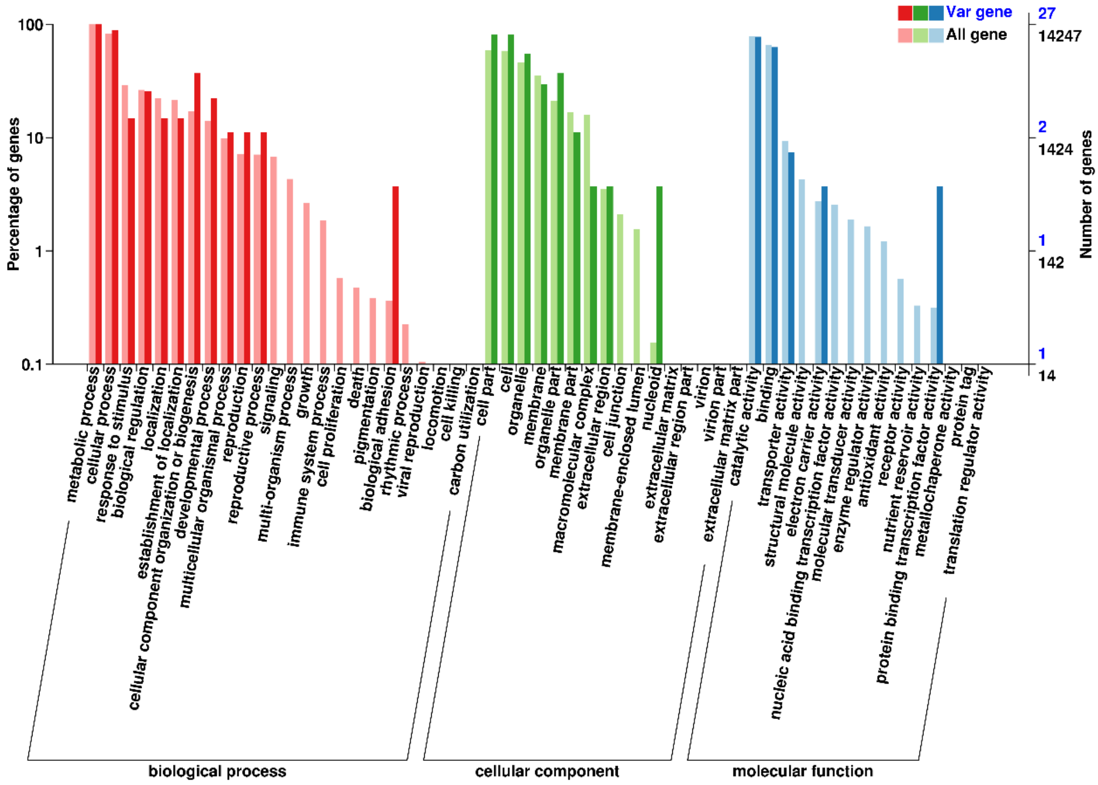

Fig. 14 The classification annotation statistics for GO.

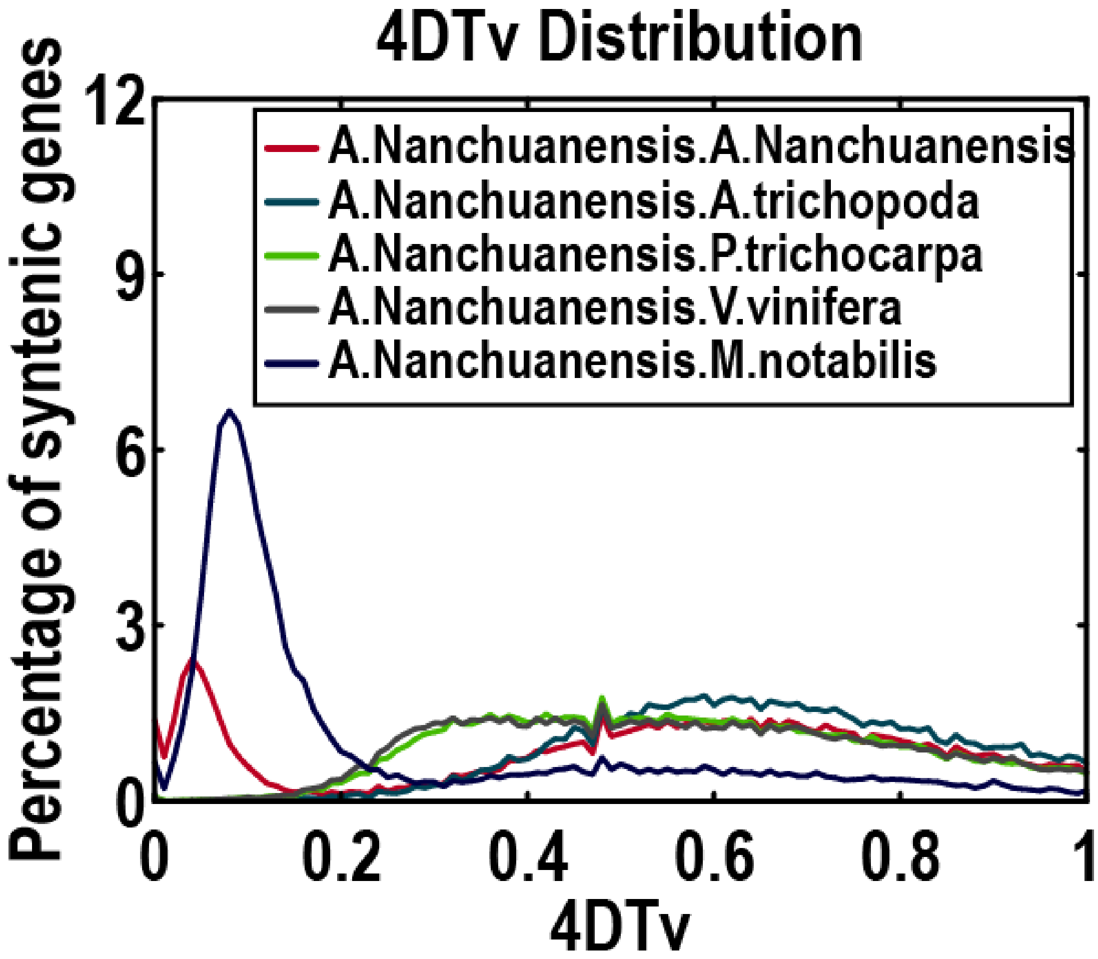

Fig. 15 The 4DTV distribution graphic.

Note: The abscissa axis represents the mutation rate of homologous genes to 4DTV, and the vertical coordinates represents the proportion of homologous gene pairs.

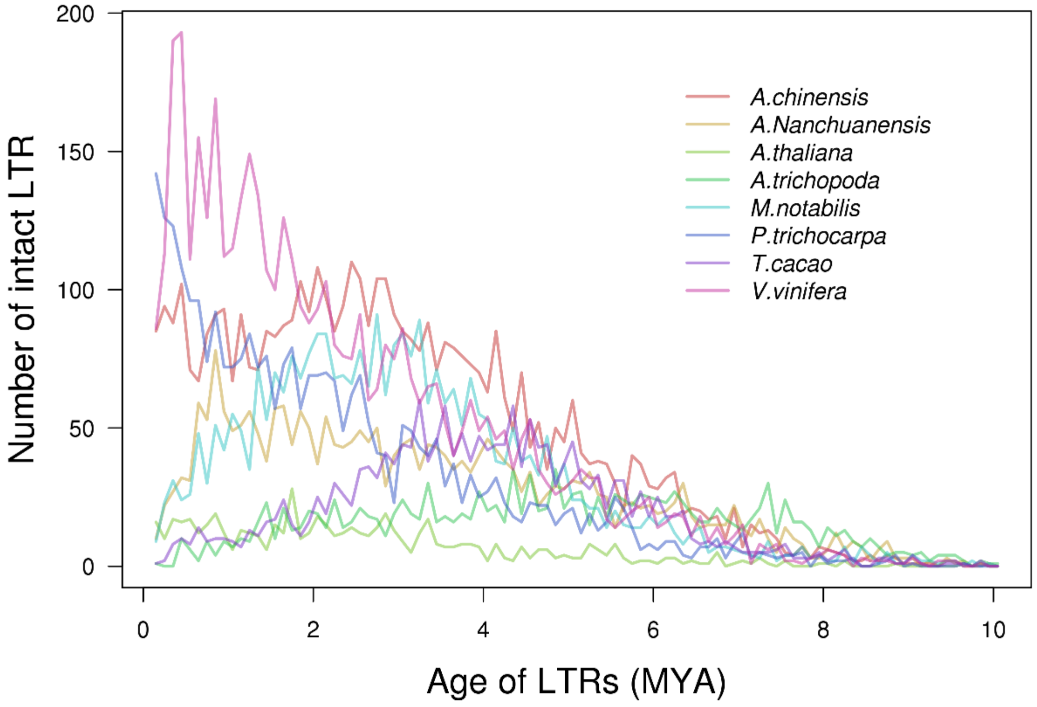

Fig. 16 The analysis graphic of LTR insertion time.

Personal Cover Click here to access/download;Personal Cover;cover letter.docx

Dear Editors:

We would like to submit an original article entitled "Chromosome genome assembly and annotation of Artocarpus Nanchuanensis with Nanopore and Hi-C sequencing data" for consideration for publication in GigaScience.

The Artocarpus Nanchuanensis (Moraceae) is an extremely endangered tree species in China. Its fruit and bark have been used as the treatment for skin in Chongqing Nanchuan for a long time, and the fruit has a good control effect on the constipation and other intestinal diseases, those features persistent cause the attention of the researchers, but the molecular mechanisms involved is little known. In our work, we revealed a high-quality chromosome-scale genome assembly and annotation for Artocarpus Nanchuanensis with Nanopore and Hi-C sequencing data. The disclosure of Artocarpus Nanchuanensis genome sequence information provides an important resource to expand our understanding of the molecular mechanism in its unique biological processes and nutritional, medicinal benefits.

This article has not been published elsewhere in whole or in part. All authors have read and approved the content, and agree to submit for consideration for publication in GigaScience. There is not any conflict involved in the article. I hope this paper is suitable for GigaScience.

We deeply appreciate your consideration of our manuscript, and we look forward to receiving comments from the reviewers. If you have any queries, please don't hesitate to contact me at the address below.

With kind regards

Yours sincerely

Xianping Ding

Corresponding Author:

Xianping Ding

Key Laboratory of Bio-Resources and Eco-Environment of Ministry of Education, College of Life Sciences, Sichuan University, Chengdu 610065, Sichuan, P.R.China;

Tel. and Fax: +86-028-85413096; Email: brainding@scu.edu.cn
