# Supplementary material for: A chromosome-level genome assembly of Artocarpus nanchuanensis (Moraceae), an extremely endangered fruit tree
Source: Gigascience. 2022 Jun 14;11:giac042. doi: 10.1093/gigascience/giac042 (PMC9197682; doi:10.1093/gigascience/giac042)
Supplement: giac042_GIGA-D-21-00106_Revision_1 [file giac042_giga-d-21-00106_revision_1.pdf]

GigaScience

A chromosome-level genome assembly of Artocarpus nanchuanensis

--Manuscript Draft--

**Manuscript Number:**	GIGA-D-21-00106R1
**Full Title:**	A chromosome-level genome assembly of Artocarpus nanchuanensis
**Article Type:**	Research
**Funding Information:**	
**Abstract:**	Artocarpus nanchuanensis (Moraceae), which is naturally distributed in China, is a representative and extremely endangered tree species. In this study, we obtained a high-quality chromosome-scale genome assembly and annotation information for A. nanchuanensis using integrated approaches, including Illumina, Nanopore sequencing platform and Hi-C. A total of 128.71 gigabases (Gb) of raw Nanopore reads were generated from 20 kb libraries, and 123.38 Gb of clean reads were obtained after filtration with 160.34x coverage depth and a 17.48 kb average read length. The final assembled A. nanchuanensis genome was 769.44 Mb with a 2.09 Mb contig N50, and 99.62% (766.50 Mb) of the assembled data was assigned to 28 pseudochromosomes. 39,596 genes (95.10%, 39,596/41636) were successfully annotated, and 129 metabolic pathways were detected. Plants disease resistance/insect resistance genes, plant-pathogen interaction metabolic pathways, and abundant biosynthesis pathways of vitamins, flavonoid and gingerol were detected. Unigene reveals the basis of species-specific functions, and gene family in contraction and expansion generally implies strong functional differences in the evolution. Compared with other related species, a total of 512 unigenes, 309 gene families in contraction and 559 gene families in expansion were detected in A. nanchuanensis . This A. nanchuanensis genome information provides an important resource to expand our understanding of the unique biological processes, nutritional and medicinal benefits, and evolutionary relationship of this species. The study of gene function and metabolic pathway in A. nanchuanensis may reveal the theoretical basis of special trait in A. nanchuanensis , and promote the study and utilization of its rare medicinal value.
**Corresponding Author:**	Xianping Ding<br>Sichuan University<br>Chengdu , Sichuan, CHINA
**Corresponding Author Secondary Information:**	
**Corresponding Author's Institution:**	Sichuan University
**Corresponding Author's Secondary Institution:**	
**First Author:**	Jiaoyu He
**First Author Secondary Information:**	
**Order of Authors:**	Jiaoyu He
	Shanfei Bao
	Junhang Deng
	Qiufu Li
	Shiyu Ma
	Yanru Cui
	Yiran Liu
	Yuqi Zhu
	Xia Wei

Powered by Editorial Manager® and ProduXion Manager® from Aries Systems Corporation

	Xianping Ding
	Kehui Ke
	Chaojie Chen
**Order of Authors Secondary Information:**	
**Response to Reviewers:**	We would like to thank the reviewers very much for taking the time and effort to review our paper so thoroughly. We have carefully considered their comments, which have helped us to make improvements to our work. Our point by point responses to their comments are presented below. The reviewers' comments are italicized and our responses are in red regular font. The changes we made in the text are also highlighted in red.<br>Reviewer: 1<br>Comments to the Author<br>Reviewer #1: He et al. contributed their study of "A chromosome-level genome assembly of Artocarpus nanchuanensis". This study present valuable and high quality genome resources for important plant group. The study has significant values for publication. While, problems remain especially in English expression and writing.<br>I will recommend to accept it after a major revision.<br>Response: Thanks to reviewers for the valuable comments and suggestions, which helped us improve the overall presentation of our work. English expression and writing have been carefully revised to ensure that the article is well understood.<br><br>Major comments:<br>1. There is significantly large space for the English expression to be improved. Some sentences were not well developed, and some others were wrong in expression.<br>As an example, can you pick the error in the sentence in line 28? Do you think "persistent" was right here? And I think there is better way to restructure this sentence.<br>Another example, lines 33 page 1 - line 1 page 2 in the main text, after reading this sentence, I am confused. I would like to know who do include 78.34 billion high-quality bases. It is so huge to have such large number of bases for a genome assembly. It should be the data generated for genome assembly. But, the sentence is truly problematic in expression. Please take care of writing, as it is very important.<br>Response: Thank you very much. Manusript has been revised and polished by a language company. "Those features persistent cause the attention of researchers" has been revised as "These features have attracted the attention of researchers" and "The draft genome sequence of mulberry tree, including 78.34 billion high-quality bases, were assembled into 330.79-Mb mulberry genome with a scaffold N50 length of 390,115 bp and contig N50 length of 34,476 bp." has been revised as "In the draft genome sequence of the mulberry tree Morus notabilis (M. notabilis), 78.34 Gb of high-quality data were obtained and assembled into a 330.79 Mb mulberry genome with a 390,115 bp scaffold N50 and 34,476 bp contig N50". The typo together with other mistakes have been revised. (please see line 2 of page 3, and line 7-9 of page 3).<br><br>2. line numbers are not consistently put, and no page number. It is very hard to put comments.<br>Response: Thank you, the line numbers and page number in the manuscript have been revised. (Please see manuscript)<br><br>3. Other than English expression, the quality of figure and table need to be significantly improved.<br>Response: We thank the reviewer for the valuable comments and suggestions, the quality of figures and tables have been improved. We replaced all the figures with the high quality edition, and tables have been revised. (Please see Table, Figure, Supplemntary table and Supplemntary figure)<br><br>4. Detailed discription is needed for some specific analyses, such us 4DTV, LTR insertion time. Why did you do these analyses? what did you find? How you results could be compared to others? What is new findings, what is the differences and the commons?<br>Response: Thank for the valuable comments and suggestions. Transversions on fourfold degenerate synonymous sites (4DTv) are neutral genetic distances that can be used to estimate the relative timing of evolutionary events. According to the homologous gene pairs between two species or within species themselves, the ratio of

	each homologous gene to the 4DTV mutation site was calculated, and a 4DTV distribution map was made. The peak of the 4DTv distribution among A. nanchuanensis and M. notabilis was closer to the current than that of A. nanchuanensis and other species, indicating that the differentiation time of A. nanchuanensis and M. notabilis appeared recently, suggesting a closer genetic relationship between them. At ancient time, the 4DTV distribution curves of A. nanchuanensis and other species were similar, which reflected these species might share similar whole-genome duplication (WGD) events. Moreover, the 4DTV distribution of A. nanchuanensis had a small peak at 0.05, which suggested that some genomic fragments duplicated recently. The purpose of long terminal repeat (LTR) accumulation is to cope with stresses, and LTR accumulation is able to reflect that the species may cope with some environmental stresses on its survival. LTR insertion time among A. nanchuanensis and other 7 related species show that the living environment of A. nanchuanensis is relatively stable. The narrow peak of LTR insertion time around 1 Mya indicated some environment stress or environment change has been imposed on A. nanchuanensis and the living environment. ((please see line 34-35 of page 11, and line 1-17 of page 12)<br><br>5. A general discussion or comparsion to related results previously published is needed for many parts, like quality and characterization of genome assembly, phylogeny, gene families in expansion and contraction, divergent time, 4DTV (if whole genome duplication is an objective of the authors), LTR insertion time.<br>Response: Thank you. The general discussion or comparsion to related results previously published of genome assembly quality and characterization, phylogeny, gene families in expansion and contraction, divergent time, 4DTV, LTR insertion time have been added in the manuscript, they helped us to improve the overall presentation of our work. (please see line 11-16 of page 8, line 19-34 of page 10, line 1, 12-31, 34-35 of page 11, line 1-17 of page 12, and line 7-13 of page 13)<br><br>6. Conclusion should not be the only summary of results. You should point out key points/values for future/related fields. And most probably, the significance and implications of this study should be provided. Or any shortage/limiation you could see.<br>Response: Thank you, the conclusion has been revised, according to the comments and suggestions of reviewers The points/values for future/related fields, and the significance and implications of this study have been provided in the manuscript. (please see line 19-35 of page 12 and line 1-20 of page 13).<br><br>7. No problem found for open data sharing, by checking the liked data the authors provided.<br>Response: Thanks you very much.<br><br>Minor ones:<br>1. line 24, do you think the reference No 12 is refered here? or just the No 1 and No 2?<br>Response: Thank you very much. The reference No 12 in line 24 is refered as the No 1 and No 2 reference. The typo wrong together with other mistakes have been revised. (please see line 32 of page 2)<br><br>2. On Latin names. please change such "A.nanchuanensis" expression to "A. nanchuanensis". This means you may need to add a space after ".".<br>Response: Thank you, and the typo together with other mistakes have been revised.<br><br>3. lines 21-24, A.nanchuanensis -> Artocarpus nanchuanensis<br>line 2, Artocarpus.nanchuanensis -> Artocarpus nanchuanensis<br>Response: Thank you. The typo together with other mistakes have been revised. (please see line 2 of page 2, line 28 of page 2)<br><br>4. line 32, "Moraceae Mulberry and Paper Mulberry", do not put them in Italic, if they are not Latin names.<br>Response: Thank you. "Moraceae Mulberry and Paper Mulberry" has been revised as "Morus notabilis" and "Broussonetia papyrifera". The typo together with other mistake have been revised. (please see line 7, 11 of page 3)<br><br>5. last paragraph in Introduction, the sentence is confusing. One confusiong woule be I do not know who could provide the necessary resources for the genome size selection.

Powered by Editorial Manager® and ProduXion Manager® from Aries Systems Corporation

And do you think genome size selection is important? And many more confusions here. Please improve the expression.
Response: Thank you. The above confusion is caused by the typo mistake, and "that not only provide the necessary resources for the genome size selection" has been revised as "These genomic data not only provide the necessary resources for the determination of genome size". (please see line 20-21 of page 3)

6. Version information should be provided for softwares used in analyses.
Response: Thank you. The version information of softwares used in analyses have been provided in the manuscript. (please see materials and methods)

7. many typos in references list, such as "k - - - mer", no publication year provided,
Response: We thank the reviewer for the valuable comments and suggestions, all the references in the manuscript have been checked carefully to ensure that the year of publication is provided. (please see References)

8. I do not know the requirment from GigaScience on the number of main figures and tables. But, it seems they are too many now in the manuscipt. Please do a reasonable re-examination, and then put some of the figures and tables to the supplementary, such as figure 1. And you may need to merge some of them into a compact figure or tables, such as figre 2 and figure 4. Also, figures (11, 12, 13) need to be improved, they are ugly and it is not hard for improvement.
Response: Thank you. Based on the enlightening comments and suggestions of reviewers, the figures and tables have been rearranged in the manuscript. We have merged some of figures/tables into a compact figure/table, put some figures/tables into supplemntary figure/table, and the quality of figure/table have been significantly improved. (Please see Table, Figure, Supplemntary table and Supplemntary figure)

9. I may be interested to see a talbe comparing the quality/properties of genomes assemblies released for any other Moraceae and the currently reported one, on parameters like sequencing tech and depth, Contig/Scaffold N50, N90, annotated genes, repeat composion.
Response: Thank you. The genomes assemblies quality of A.nanchuanensis and its related Moraceae plants (M. notabilis, B. papyrifera, A. nanchuanensis) have been collated into Table 3. (Please see Table 3)

10. Other than Table 2 & 3, it would be interesting to present mapping rate and genome coverage (like 10 fold coverage) of those ONT and Illumina data used for assembly.
Response: Thank you. The mapping rate and genome coverage of Illumina data are 99.41% and 68.01 fold, the genome coverage of ONT data are 160.34 fold. According to the suggestions of reviewers, the above contents have been added to Table 1. (Please see Table 1)

11. When the plant Latin names shown up for the first time, you may need to show the full names. And the right citations should be provided if genomic data were used in this study, such as the genomic data for A.thaliana Arabidopsis thaliana (L.) Heynh, A.trichopoda Amborella trichopoda, P.trichocarpa populus trichocarpa, A.chinensis Actinidia chinensis Planch, V.vinifera Vitis vinifera L, M.notabilis Morus notabilis Schneid , T.cacao Theobroma cacao L.
Response: We thank the reviewer for the valuable comments and suggestions, the full Latin name of A. thaliana, A. trichopoda, P. trichocarpa, A. chinensis, V. vinifera, M. notabilis and T. cacao have been shown up for the first time, and the right citations of genomic data have been provided in the manuscript. (please see line 32-34 of page 6 and line 1 of page 7).

12. I am wondering some symbols are in format of not a routinely English format (like ； in Section 2.4 line 34).
Response: We thank the reviewer for the valuable comments and suggestions, the format of symbols have been careful checked and revised to ensure the symbol format is right. (please see line 21-24 of page 5)

13. Section 3.5 Comparative genomics line 23, "expanding and contracting gene families" is not good expression. It may be better to be changed to "gene families in

contraction and expansion". There are many such bugs in expression across the whole paper. Please check related paper for improvement in English expression.
Response: Thank you for the valuable comments and suggestions, "expanding and contracting gene families" has been replaced as "gene families in contraction and expansion", and related expression bugs have also been checked and modified. (please see line 8-35 of page 11 and line 1-17 of page 12)

Reviewer: 2
Comments to the Author
Reviewer #2: This article is presenting the genome assembly of Artocarpus nanchuanensis. This work could be of great interest because of its endangered status and its nutritional and medicinal value.
The results seem to be of quality but the manuscript has to be improved. The methods seem to be well used. The major concern is that the reading is difficult because the sentences are often too long and contain many writing errors. In addition, the methods lack detailed information.
Here is a list of remarks, questions and comments. This is not an exhaustive list of all the corrections to be made to the manuscript.
Response: We thank the reviewer for the valuable comments and suggestions, the typo together with some other mistakes have been corrected in the revised manuscript.

1. - Abstract Line 5: it could be more suitable to cite the methods as follow: whole genome sequencing using Illumina and Oxford Nanopore Technology platforms and chromosomal conformation capture technique
Response: Thank you very much. Due to the abstract limitations, whole genome sequencing using Illumina and Oxford Nanopore Technology platforms and chromosomal conformation capture technique has been cited in introduction. (Please see line 20 of page 3)

2. - Abstract Line 6: "Nanopore Sequel reads" should be replaced by "Nanopore reads"
Response: We thank the reviewer for the valuable comments and suggestions, "Nanopore Sequel reads" has been replaced by "Nanopore reads". (Please see line 6 of page 2.)

3. - Abstract Line 12: "genome assembly integrity" should be replaced by "genome assembly completeness".
Response: We thank the reviewer for the valuable comments and suggestions, the relevant expressions in the manuscript as "genome assembly completeness" (Please see line 9 of page 5) .

4. - Introduction Lines 12-13: sentence has to be improved. It is not appropriate to write that the genomes "have been made in detail". The two following sentences have to be improved too.
Response: Thank you. The sentence in introduction lines 12-13 and the two following sentences have been revised as "In the draft genome sequence of the mulberry tree Morus notabilis (M. notabilis), 78.34 Gb of high-quality data were obtained and assembled into a 330.79 Mb mulberry genome with a 390,115 bp scaffold N50 and 34,476 bp contig N503. The assembled genome of Broussonetia papyrifera (B. papyrifera) was 386.83 Mb with a 29.48 Mb scaffold N50 and 171.17 Kb contig N504. The genome data analysis of M. notabilis and B. papyrifera provides a theoretical basis for the study of fibre development, lignin and flavonoid metabolism, nitrogen metabolism, important metal tolerance functions and stress resistance evolution, but the genomic details of A. nanchuanensis remain unknown." (Please see line 7-15 of page 2)

5. - Introduction Lines 21-28. Too long sentence. I'm not sure that "high-pass" is the good term.
Response: We thank the reviewer for the valuable comments and suggestions, "high-pass" has been replaced as "High-throughput/resolution chromosome conformation capture" and the long sentences have been revised. (Please see line 16-24 of page 3)

6. - Figure 1:
o Chromatin instead of "Cromatin"

	o "Correction" instead of "Crrection"<br>o "Genome" instead of "Gemone"<br>o "polishing" instead of "polish"<br>o What is "Genome evalue"?<br>o This figure presents a flow chart. The HiC part should be placed after the nanopore part<br>o Hi-C instead of "Hic"<br>Response: Thank you. All the typo together with some other mistakes in the Figure 1 have been corrected, and the flow chart has been modified as reviewer's suggestion. (please see Fig. 1)<br><br>7. - Sample and DNA extraction:<br>o "The samples of genome were young leaves" could be replaced by "For genome sequencing, DNA was extracted from young leaves" for example.<br>Response: We thank the reviewer for the valuable comments and suggestions, "The samples of genome were young leaves" has been replaced by "For genome sequencing, DNA was extracted from 100 mg young leaves by the CTAB method". (please see line 30-31 of page 3)<br><br>o What is the quantity of young leaves used in the CTAB extraction process?<br>Response: Thank you, the quantity of young leaves used in the CTAB extraction process is 100 mg and the detail of DNA extraction have been added in Sample and DNA, RNA extraction. (please see line 30-34of page 3)<br><br>o Can you more detail this process or provide a reference?<br>Response: Thank you for the valuable comments and suggestions. The process detail and reference of DNA and RNA extraction have been added in Sample and DNA, RNA extraction. (please see line 30-34 of page 3, and 1-12 of page 4)<br><br>o "for transcriptome analysis" could be replaced by "for RNA extraction"<br>Response; Thank you, "for transcriptome analysis" has been replaced as "for RNA extraction". (please see line 2 of page 4)<br><br>o "ONT Library with 20Kb fragment length was constructed following the manufacturer's protocol"<br>Response: We thank the reviewer for the valuable comments and suggestions. "ONT Library with 20 Kb insertion size were constructed for the Nanopore platform according to the manufacturers' protocols" has been replaced by "ONT Library with a 20 kb fragment length was constructed following the manufacturer's protocol" (please see line 14-15 of page 4)<br><br>o the rest of the paragraph should be reworded.<br>Response: Thank you. According to reviewer's valuable comments and suggestions, the rest of the paragraph has been reworded. (please see line 27-34 of page 3 and line 1-12 of page 4)<br><br>o Integrity of DNA was "checked on" pulsed field electrophoresis.<br>Response: Thank you, "the integrity of DNA was detected by pulsed field electrophoresis" has been replaced by "the integrity of the DNA was checked on pulsed field electrophoresis". (please see line 32-33 of page 3)<br><br>o R9 flow cell instead of "R9 cell"<br>Response: Thank you, "R9 cell" has been replaced as "R9 flow cell". (please see line 19 of page 4)<br><br>o Preparation instead of "prep"<br>Response: Thank you, "prep" has been replaced as "Preparation". (please see line 17 of page 4)<br><br>o How do you realize the genome size estimation? Do you estimate the heterozygosity rate?<br>Response: Thanks for reviewer's valuable comments and suggestions. A total of 51.76 Gb of high-quality A. nanchuanensis data were obtained from the Illumina sequencing platform with an approximately 68× sequencing depth, and the genome size was

calculated to be 761.07Mb. Based on 4 ^ K/genome>200, a kmer distribution map of K = 17 was constructed. The amount of repeated sequences content was estimated to be approximately 55.80%, and the heterozygosity was estimated to be approximately 0.93%, indicating that the A. nanchuanensis genome was highly heterozygotic and complex. The above content has been added to Initial characterization of A. nanchuanensis genome. (please see Fig. 3, line 29-35 of page 7).

o How do you perform the read cleaning? Softwares?
Response: We thank the reviewer for the valuable comments and suggestions, the data sequenced by the sequencer is raw data. Clean data obtain from raw data after two steps as fellow: firstly, remove the reads containing joints; secondly, remove low-quality reads with a proportion of N greater than 10% and a mass value of Q≤10 that account for more than 50% of the whole read.

o Figure 2: caption is missing.
Response: Thank you. The caption of Figure 2 has been replaced as “The A. nanchuanensis sample and genomic interaction analysis”. (please see Fig. 2)

8. - There is no detail on the RNA extraction (quantity engaged in the extraction, protocol used) and on the sequencing library preparation.
Response: Thank you for the comments and suggestions, the detail of the RNA extraction and sequencing library preparation were added in the Samples and DNA, RNA extraction, Library construction and high-throughput sequencing of Materials and methods. (please see in line 1-12 and line 21-27 of page 4)

9. - Genome assembly and quality assessment:
o "three rounds calibration by racon and Pilon", did you mean assembly correction?
Response: Thank you very much. Yes, "three rounds calibration by racon and Pilon" mean assembly correction procession.

o "BWA software was used to align short sequences on the reference genome" could be better than this long sentence.
Response: We thank the reviewer for the valuable comments and suggestions, "BWA software was used to compare the short sequences obtained from second-generation sequencing with the reference genome" has been replaced as "BWA software was used to align short sequences on the reference genome". (please see line 6-7 of page 5)

o BUSCO is used to evaluate the completeness of the assembly
Response: Thank you, “CEGMA v2.5 (default parameters) database and the BUSCO v2.0 software were used to evaluated the integrity of the assembled genome” has been replaced as “The CEGMA v2.5 (default parameters) database and BUSCO v4.0.6 (parameters: odb10, -c 24 -e 1e-3) were used to evaluate the completeness of the assembly". (please see line 7-9 of page 5)

10. - In general, there is a lack of validation of the assembly. Can you add some evaluations such as KAT histogramms which reflect the completeness of the assembly process (https://kat.readthedocs.io/en/latest/) and use Merqury to evaluate the quality of the assembly? (https://genomebiology.biomedcentral.com/articles/10.1186/s13059-020-02134-9).
Response: Thanks to the reviewers for the valuable comments and suggestions. Genome assembly completeness evaluation was conducted by second-generation sequencing reads mapped analysis and core gene integrity assessment by bwa, CEGMA v2.59 and BUSCO v4.0.6. Statistical alignment analysis of second-generation sequencing reads showed that clean reads located on the reference genome accounted for 99.41% of the total clean reads (363,371,475/365,545,724). The paired-end sequences of the correct size that were located on the reference genome, accounted for 93.56% of the total clean reads (341,995,184/365,545,724). The core gene integrity assessment was performed by CEGMA v2.59. Here, 445 CEGs were present in assembly, accounting for 97.16% of all CEGs (445/458), while 232 highly conserved CEGs were present in the assembly, accounting for 93.55% of all CEGs (232/248). The database in BUSCO v4.0.6 contains 1,614 conserved core genes, and the number of complete genes present in the assembly is 1583 (98.08%). (please see line 17-26 of page 8 and Fig. 4)

Powered by Editorial Manager® and ProduXion Manager® from Aries Systems Corporation

11. - Genome annotation analysis:
o this section contains very long sentences.
o "based on transcriptome data unreferenced assembly": I'm not sure to understand what authors mean.
o "parameter for blast: The e-value" please clarify
o Lines 28-31: please correct the sentence.
Response: Thank you for the valuable comments. Long sentences have been revised as several short sentences, "based on transcriptome data unreferenced assembly" has been revised as "based on the transcriptome data of a nonreference assembly", "parameter for blast: The e-value" has been clarified as "parameter: e-value -e 1e-5", and the sentence in Lines 28-31 has been corrected as "Noncoding RNAs were predicted by different strategies based on their structural characteristics. Rfam v12.1 (parameters: 1e-5) was used to identify microRNAs and rRNAs, and tRNAscan-SE v1.3.1 (parameters: 1e-5) was used to identify tRNAs". (please see line 27-34 of page 5 and line 1-30 of page 6)

12. - Gene family and phylogenetic analysis :
o Some sentences have to be reworded
Response: Thank you for the valuable comments and suggestions. The sentences of Gene family and phylogenetic analysis have been reworded. (please see line 32-34 of page 6 and line 1-26 of page 7)

13. - Genome assembly and assembled completeness evaluation
o "assembled" could be removed from this title
o The third first sentences should be reworded.
o Table 1: I'm not sure that the authors have to mention "three-generation" each time if not they have to put "third generation". The names of the lanes could be more precise and the caption could be removed. I don't understand the meaning of the sentence "Contig length means the length of Contig in the middle of more than 1Kb of scaffolding".
o The last sentence could be rephrased.
Response: Thank you. “Genome assembly and assembled completeness evaluation" has been revised as "Genome assembly and completeness evaluation". The third first sentences and the last sentence have been rephrased. Table 1 and Table 2 have been rearranged to make the data easier to read, "Nanopore three-generation sequencing results" has been revised as "Nanopore", the names of the lanes have been revised to be more precise and useless captions have been removed. "Contig length means the length of Contig in the middle of more than 1Kb of scaffolding" has been revised as "the length of contig in the scaffold, and scaffold length exceeds 1 Kb". (please see line 8-26 of page 8, Table1 and Table 2)

14. - Figure 3: the title should include Hi-C. "The Y axis represents the number of reads" could replace the last sentence.
Response: Thank you for the valuable comments and suggestions. The figure tittle has been revised as “The analysis of Hi-C library construction and heat map”, and "The vertical coordinate is the number of random extracted 100,000 pairs of Reads corresponding to the insert fragment of different length" has been revised as "The Y axis represents the number of reads". (please see Fig. 5)

15. - Hybrid assembly, scaffolding and chromosome anchoring:
o the writing of this paragraph should be improved. For example, "236,274,160 Mb pairs were uniquely correlated to the genome, including 56,964,635 pairs valid Hi-C data" could be rephrased as: "236,274,160 paired reads were uniquely mapped on the genome assembly, including 56,964,635 valid Hi-C paired reads"
Response: Thank you. The writing of this paragraph has been revised, and "236,274,160 Mb pairs were uniquely correlated to the genome, including 56,964,635 pairs valid Hi-C data" has been replaced as "236,274,160 paired reads were uniquely mapped on the genome assembly, including 56,964,635 valid Hi-C paired reads". (please see line 2-4 of page 9)

o Table 3: the caption brings nothing. Rather, it should explain what each class of reads represents.
Response: We thank the reviewer for the valuable comments and suggestions. The

	caption has been revised as "The Hi-C sequencing data types and proportion of A. nanchuanensis", and each class of reads have been represented in the note of Supplementary Table 2. (please see Supplementary Table 2)<br><br>o "manually adjusted" could be replaced by "manual adjustment"<br>Response: Thank you, "manually adjusted" has been replaced by "manual adjustment". (please see line 15 of page 9)<br><br>o Figure 4: "inside" instead of "inutside", "Each color" instead of "Each coloured"<br>Response: Thank you very much. The typo along with other mistakes have been revised, "inutside" has been revised as "inside", "Each coloured" has been revised as "Each color". (Please see Fig. 2.)<br><br>o Figure 5: "order" instead of "Order"<br>o Table 5: "correction" instead of "corrected". The sentence "the length of Contig in the middle of the scaffold above 1Kb" should be reworded<br>Response: Thank you for the valuable suggestions. The typo along with other mistakes have been revised. "Order" has been revised as "order", "corrected" has been revised as "correction", and "the length of Contig in the middle of the scaffold above 1Kb" has been revised as "length of contig in the scaffold, and scaffold length exceeds 1 Kb". (Please see Fig. 5 and Table 2)<br><br>16. - Repeat annotation, gene prediction and gene annotation:<br>o Table 8: I think that the name of the columns could be more precise in order to remove the caption. "The introns average number in per gene" should be replaced for example by "the average number of intron per gene".<br>Response: We thank the reviewer for the valuable comments and suggestions, the name of the columns have been revised to be more precise, and the table caption has been removed. (Please see table 5)<br><br>o Table 9: the title could be "Functional annotation statistics"<br>Response: Thank you, the title has been revised as "Functional annotation statistics of A. nanchuanensis". (Please see Supplementary Table 7)<br><br>o "The homologous gene of Artocarpus nanchuanensis and morous notabilious was" could be replaced by "The number of homologous genes between A. nanchuanensis and Morous notabilious was"<br>Response: Thank you. "The homologous gene of Artocarpus nanchuanensis and morous notabilious was" has been replaced by "The number of homologous genes between A. nanchuanensis and Morous notabilious was". (please see line 7-8 of page 10)<br><br>o Figure 6: the title should be "distribution of the number of genes among the three methods"<br>Response: Thanks for the valuable comments and suggestions, the title has been revised as "Distribution of the number of genes among the three methods". (Please see Supplementary Fig. 1)<br><br>o Why do you mean by "the three dominant genes"? The three top over expressed genes?<br>Response: Thanks to reviewers for the valuable comments and suggestions, "the three dominant genes" was replaced as "The top three over expressed genes". (please see line 14 of page 10)
**Additional Information:**	
**Question**	**Response**
Are you submitting this manuscript to a special series or article collection?	No
**Experimental design and statistics**	Yes

Full details of the experimental design and statistical methods used should be given in the Methods section, as detailed in our Minimum Standards Reporting Checklist. Information essential to interpreting the data presented should be made available in the figure legends.<br><br>Have you included all the information requested in your manuscript?	
**Resources**<br><br>A description of all resources used, including antibodies, cell lines, animals and software tools, with enough information to allow them to be uniquely identified, should be included in the Methods section. Authors are strongly encouraged to cite Research Resource Identifiers (RRIDs) for antibodies, model organisms and tools, where possible.<br><br>Have you included the information requested as detailed in our Minimum Standards Reporting Checklist?	Yes
**Availability of data and materials**<br><br>All datasets and code on which the conclusions of the paper rely must be either included in your submission or deposited in publicly available repositories (where available and ethically appropriate), referencing such data using a unique identifier in the references and in the "Availability of Data and Materials" section of your manuscript.<br><br>Have you have met the above requirement as detailed in our Minimum Standards Reporting Checklist?	Yes

Powered by Editorial Manager® and ProduXion Manager® from Aries Systems Corporation

Manuscript
Click here to access/download;Manuscript;paper_revise_10-29.doc

A chromosome-level genome assembly of *Artocarpus nanchuanensis*

Jiaoyu He[1,2,3], Shanfei Bao[1,2,3], Junhang Deng[1,2,3], Qiufu Li[1,2,3], Shiyu Ma[1,2,3], Yiran Liu[1,2,3], Yanru Cui[1,2,3], Yuqi Zhu[1,2,3,4], Xia Wei[1,2,3], Xianping Ding[1,2,3]*, Kehui Ke[5], Chaojie Chen[5].

1 Key Laboratory of Bio-Resources and Eco-Environment of Ministry of Education, College of Life Sciences, Sichuan University, Chengdu 610065, Sichuan, P.R.China.

2 Chongqing Jinfo Shan Advanced Research Institute, Chongqing, P.R.China.

3 Bio-resource Research and Utilization Joint Key Laboratory of Sichuan and Chongqing, Sichuan and Chongqing, P.R.China.

4 Wood Comprehensive Factory of Chengdu, Sichuan, P.R.China.

5 Biomarker Technologies Corporation, Beijing 101300, China.

Address for Correspondence: Institute of Medical Genetics, College of Life Sciences, Sichuan University, Chengdu 610064, China.

* Corresponding author:

Institute of Medical Genetics, College of Life Sciences, Sichuan University, Chengdu 610064, China.

E-mail: brainding@scu.edu.cn

Telephone: 86-028-85413096

Fax: 86-028-85415895

Email address:

Jiaoyu He: 1061355567@qq.com; Shanfei Bao: 715714892@qq.com;
Junhang Deng: 1916358148@qq.com; Qiufu Li: lqf1192069072@126.com;
Shiyu Ma: 895686227@qq.com; Yanru Cui: 512927123@qq.com;
Yiran Liu: 532154290@qq.com; Yuqi Zhu: 408843724@qq.com;
Xia Wei: 531197860@qq.com; Xianping Ding: brainding@scu.edu.cn;
Kehui Ke: kehui.ke@outlook.com; Chaojie Chen: 352300595@qq.com.

Abstract

Artocarpus nanchuanensis (Moraceae), which is naturally distributed in China, is a representative and extremely endangered tree species. In this study, we obtained a high-quality chromosome-scale genome assembly and annotation information for *A. nanchuanensis* using integrated approaches, including Illumina, Nanopore sequencing platform and Hi-C. A total of 128.71 gigabases (Gb) of raw Nanopore reads were generated from 20 kb libraries, and 123.38 Gb of clean reads were obtained after filtration with 160.34x coverage depth and a 17.48 kb average read length. The final assembled *A. nanchuanensis* genome was 769.44 Mb with a 2.09 Mb contig N50, and 99.62% (766.50 Mb) of the assembled data was assigned to 28 pseudochromosomes.

39,596 genes (95.10%, 39,596/41636) were successfully annotated, and 129 metabolic pathways were detected. Plants disease resistance/insect resistance genes, plant-pathogen interaction metabolic pathways, and abundant biosynthesis pathways of vitamins, flavonoid and gingerol were detected. Unigene reveals the basis of species-specific functions, and gene family in contraction and expansion generally implies strong functional differences in the evolution. Compared with other related species, a total of 512 unigenes, 309 gene families in contraction and 559 gene families in expansion were detected in *A. nanchuanensis*.

This *A. nanchuanensis* genome information provides an important resource to expand our understanding of the unique biological processes, nutritional and medicinal benefits, and evolutionary relationship of this species. The study of gene function and metabolic pathway in *A. nanchuanensis* may reveal the theoretical basis of special trait in *A. nanchuanensis*, and promote the study and utilization of its rare medicinal value.

Key words: *A. nanchuanensis*, sequencing, Illumina, Nanopore, Hi-C, genome assembly, gene annotation, gene family.

1 Introduction

Artocarpus nanchuanensis (*A. nanchuanensis*), which is mainly distributed in Chongqing Nanchuan, is part of a new generation of southern urban greening tree species; this species has high quality and excellent fast-growing characteristics, that allow it to live in acidic soil and environments with heavy atmospheric pollution due to it strong ability to resist pollution and disease[1,2]. The fruit of *A. nanchuanensis* contains a variety of polysaccharides, amino acids, trace elements and vitamins, which have a good control effect on constipation and other intestinal diseases[2]. The

fruit and bark have been used in the treatment of skin diseases in Chongqing Nanchuan for a long time. These features have attracted the attention of researchers[1], and promoted the steady progress of relevant research. As research has developed, high-quality genome data are needed for this valuable species to promote studies of the molecular mechanisms related to its nutritional and medicinal value, as well as those of individual genome structure, genome evolution and species diversity.

In the draft genome sequence of the mulberry tree *Morus notabilis* (*M. notabilis*), 78.34 Gb of high-quality data were obtained and assembled into a 330.79 Mb mulberry genome with a 390,115 bp scaffold N50 and 34,476 bp contig N50[3]. The assembled genome of *Broussonetia papyrifera* (*B. papyrifera*) was 386.83 Mb with a 29.48 Mb scaffold N50 and 171.17 Kb contig N50[4]. The genome data analysis of *M. notabilis* and *B. papyrifera* provides a theoretical basis for the study of fibre development, lignin and flavonoid metabolism, nitrogen metabolism, important metal tolerance functions and stress resistance evolution, but the genomic details of *A. nanchuanensis* remain unknown.

To protect this species and make full use of its rare value, we applied a combined strategy involving Illumina sequencing, Nanopore single molecule sequencing and High-throughput/resolution chromosome conformation capture (Hi-C) technologies to generate sequencing data for the chromosomal genome construction and annotation of *A. nanchuanensis*[5–8] (Fig.1). These genomic data not only provide the necessary resources for the determination of genome size, but also provide convenience for research on reproduction and species evolution based on speciation and the local environment, which is beneficial to studies on the medicinal and economically valuable traits.

2 Materials and methods

2.1 Samples and DNA, RNA extraction

The oldest *A. nanchuanensis* tree surviving in Nanchuan district was selected as the sampling source (Fig. 2). Its fruits, young leaves and roots were preserved in liquid nitrogen until DNA, RNA extraction.

For genome sequencing, DNA was extracted from 100 mg young leaves by the CTAB method[9]. The concentration and purity of the extracted DNA from the sample was detected by NanoDrop and Qubit; the integrity of the DNA was checked on pulsed field electrophoresis[10]; and the extracted high-quality DNA was prepared for subsequent sequencing[10].

The leaves and fruits in the different growth stages were uniformly mixed, and a 100 mg mixture was used for RNA extraction by the RNAprep Pure Plant Kit (Tiangen, Beijing). The quality and concentration of the RNA were detected by Nanodrop. High-quality mRNA was purified by mRNA capture beads, and first-strand synthesis reaction buffer, random primers, and reverse transcription reagents were added to purified mRNA for mRNA fragmentation and cDNA synthesis. The synthesized and purified cDNA was incubated with end repair reaction buffer and end repair enzyme mix for end repair and 3'-end A addition in the PCR instrument. The joint, ligase and USER enzymes were added to the reaction products for joint connection and joint opening, and magnetic beads were used for fragment selection. Finally, the selected fragments were amplified by PCR, and the products were purified for sequencing.

2.2 Library construction and high-throughput sequencing

An ONT library with a 20 Kb fragment length was constructed following the manufacturer's protocol. The large segments of the extracted DNA were filtered by the BluePippin™ System, and the large segments of DNA, ONT Template Preparation Kit (SQK-LSK109) and NEB Next FFPE DNA Repair Mix Kit were used to prepare a library. The high-quality library was sequenced on the ONT PromethION Beta platform with a corresponding R9 flow cell and ONT sequencing reagent kit (EXP-FLP001.PRO.6).

An Illumina sequencing library was prepared for genome size estimation, genome assembly correction and evaluation. The paired-end (PE) library with a 350 bp insertion size was prepared for the Illumina platform according to the manufacturers' protocols (San Diego, 112 CA, USA) and subjected to PE (2 × 150 bp) sequencing on an Illumina NovaSeq platform (Illumina, San Diego, CA, USA). The low-quality bases, adapter sequences, and duplicated sequence reads were filtered out to obtain clean reads for subsequent analysis.

Hi-C fragment libraries were constructed with 300-700 bp insertion sizes, as illustrated in Rao et al[11], and sequenced by sequencing by synthesis (SBS) using the Illumina platform. Briefly, adapter sequences of raw reads were trimmed and low-quality PE reads were removed to generate clean data.

2.3 Genome assembly and quality assessment

Nanopore next-generation clean sequencing data were obtained by Canu v1.5[12] software. In the correction step, Canu v1.5 first selected longer seed reads with the

settings 'genomeSize=780000000' and 'corOutCoverage=50'. SMARTdenovo (default parameters) software was used to assemble the corrected data, and then the next-generation sequencing data were used to conduct three rounds of calibration by Racon v1.4.21 (default parameters)[13] and Pilon[14] v1.22 (parameters: --mindepth 10 --changes --threads 4 --fix bases) software. The assembly results were evaluated by the read alignment rate, core gene integrity, and BUSCO evaluation. BWA[15] software was used to align short sequences on the reference genome. The CEGMA[16] v2.5 (default parameters) database and BUSCO v4.0.6 (parameters: odb10, -c 24 -e 1e-3)[17] were used to evaluate the completeness of the assembly.

2.4 Chromosomal-level genome assembly using Hi-C data

Before chromosome assembly, we first performed a preassembly for error correction of scaffolds, which required splitting scaffolds into segments of 50 kb on average. The Hi-C data were mapped to these segments using BWA (version 0.7.10-r789, default parameters) software. Only uniquely alignable read pairs whose mapping quality was greater than 20 were retained for further analysis. Invalid read pairs, including dangling-end and self-cycle, re-ligation and dumped products, were filtered by HiC-Prov2.8.1 (default parameters)[18]. The uniquely mapped data were retained to perform assembly with LACHESIS[19] software. Any two segments that showed inconsistent connections with information from the raw scaffold were checked manually. These corrected scaffolds were assembled by LACHESIS. Parameters for running LACHESIS included CLUSTER_MIN_RE_SITES = 5; CLUSTER_MAXLINK_D ENSITY = 2; CLUSTER_ NONINFORMATIVE_RATIO = 2; ORDER_MIN_N_R ES_IN_TRUN = 5; and ORDER_MIN_N_RES_IN_ SHREDS = 5. After this step, placement and orientation errors exhibiting obvious discrete chromatin interaction patterns were manually adjusted.

2.5 Genome annotation analysis

Due to the relatively poor conservation of interspecies repeat sequences, it is necessary to construct a unique repeat sequence database for predicting repeat sequences of specific species. LTR_FINDER[20] v1.05 (default parameters) and RepeatScout[21] v1.0.5 (default parameters) were used to construct the repetitive sequence database of *A. nanchuanensis* based on structure prediction and de novo sequencing theory. Then, the database was classified by PASTEClassifier v1.0 (default parameters)[22] and merged with Repbase19.06[23] (null) as the final repetitive sequence database. Finally, RepeatMasker[24] (parameters: -nolow -no_is -norna

-engine wublast -qq -frag 20000) software was used to predict the repetitive sequences in the *A. nanchuanensis* genome based on the constructed repetitive sequence database.

The structures of coding genes were predicted by ab initio prediction, homologous species prediction and unigene prediction using three different strategies.. Genscan[25] v3.1, Augustus[26] v2.4, GlimmerHMM[27] v3.0.4, GeneID[28] v1.4 and SNAP[29] (version 2006-07-28) were used for ab initio prediction with default parameters. GeMoMa[30,31] v1.3.1 (default parameters) was used for homologous species prediction; Hisat[32] v2.0.4 (parameters --max-intronlen 20000，--min-intronlen 20) and Stringtie[33] v1.2.3 (default parameters) were used for assembly based on reference transcripts. TransDecoder v2.0 and GeneMarkS-T[34] v5.1 were used for gene prediction with default parameters. PASA[35] v2.0.2 (parameters: -align_tools gmap, -maxIntronLen 20000) was used to predict unigene sequences based on the transcriptome data of a nonreference assembly. Finally, EVM[36] v1.1.1 (default parameters) was used to integrate the prediction results obtained by the above three methods, and PASA v2.0.2 (parameters: -align_tools gmap, -maxIntronLen 20000) was used to modify the prediction results.

Noncoding RNAs were predicted by different strategies based on their structural characteristics. Rfam[37] v12.1 (parameters: 1e-5) was used to identify microRNAs and rRNAs, and tRNAscan-SE[38] v1.3.1 (parameters: 1e-5) was used to identify tRNAs.

The predicted protein sequences were compared with GenBlastA[39] v1.0.4 (parameter: e-value -e 1e-5), and immature stop codons and transcoding mutations in the gene sequences were searched to obtain pseudogenes by GeneWise[40] 2.4.1 (default parameters).

The predicted gene sequences were aligned to the nonredundant protein sequences (NR)[41], eukaryotic orthologous groups of proteins (KOG)[42], Gene Ontology (GO)[43], Kyoto Encyclopedia of Genes and Genomes (KEGG)[44], TrEMBL[45] and other functional databases by BLAST[46] v2.2.31 (parameters: -evalue 1e-5), to perform KEGG pathway, KOG functional, GO functional and other gene functional annotation analyses.

2.6 Gene family and phylogenetic analysis.

The protein sequences of *A. nanchuanensis* and their related species *(Arabidopsis thaliana (A. thaliana)*[47], *Amborella trichopoda (A. trichopoda)*[48], *Populus trichocarpa (P. trichocarpa)*[49], *Actinidia chinensis (A. chinensis)*[50], *Vitis vinifera (V. vinifera)*[51],

Morus notabilis Schneid (M. notabilis)[52], *and Theobroma cacao (T. cacao)*[53]) were aligned to analyse gene replication within the species, the evolution between species and the classification of species-specific genes. OrthoMCL[54] v2.0.9 (parameters: PercentMatchCutoff 50, EvalueExponentCutoff -5) software was used to classify the protein sequences of *A. nanchuanensis*, *A. thaliana*, *A. trichopoda*, *P. trichocarpa*, *A. chinensis*, *V. vinifera*, *M. notabilis*, and *T. cacao* to determine unique gene families in *A. nanchuanensis*.

PHYML[55] (version: 20151210, parameters: -gapRatio 0.5 -badRatio 0.25 -model HKY85 -bootstrap 1000) was used to construct the evolutionary tree based on the single-copy protein sequences of *A. nanchuanensis* and 7 other species to study the evolutionary relationships among species. TimeTree (http://www.timetree.org/) was used to select the known taxa for time calibration, and Mcmctree (parameter: default) was used to estimate the time of interspecies differentiation. CAFE 4.2[56] (parameter: lambda -l 0.002) was used to conduct gene family contraction and expansion analysis. The Branch model of the CodeML[57] module in PAML 4.7a (parameters: noisy = 3, verbose = 1, runmode = 0, seqtype = 1, CodonFreq = 2, clock = 0, aaDist = 0, model = 2, NSsites = 2, icode = 0, Mgene = 0, fix_kappa = 0, kappa = .3, fix_omega = 0, omega = 1, ncatG = 2, getSE = 0, RateAncestor = 0, Small_Diff = .45e-6, cleandata = 1, and fix_blength = 0) was used to analyses the selection pressure of single-copy genes and conduct the functional annotation and enrichment analysis.

LTR_FINDER v1.07 (parameter: default) and PS SCAN[58] (version: 3.8.31, parameter: default) software were applied to search for LTR sequences in the genome with scores greater than or equal to 6 points, and the repeated results were filtered with LTR_FINDER. The LTR flanking sequences were compared with MUSCLE[59] (version: 3.8.31, parameter: default), and the distance was calculated by DistMat software using a Kimura model with a 7.3*10-9 molecular clock.

3 Results and discussion

3.1 Initial characterization of the *A. nanchuanensis* genome

A total of 51.76 Gb of high-quality *A. nanchuanensis* data were obtained from the Illumina sequencing platform with an approximately 68× sequencing depth, and the genome size was calculated to be 761.07Mb. Based on 4 ^ K/genome>200, a kmer distribution map of K = 17 was constructed (Fig. 3). The amount of repeated sequences content was estimated to be approximately 55.80%, and the heterozygosity was estimated to be approximately 0.93%, indicating that the *A. nanchuanensis* genome was highly heterozygotic and complex. Details are shown in Table 1.

A total of 128.71 gigabases (Gb) of reads were generated by the Nanopore platform, and 123.38 Gb of clean data were obtained after quality control. The average read length reached 17.48 kb, the N50 read length was 19.18 kb, and the total sequencing depth was approximately 160.34 ×. Clean data obtained by filtering out the low-quality data reached 7,057,335 reads. Details are shown in Table 1.

The total sequencing depth of the Illumina and Nanopore platforms was 228.35×.

3.2 Genome assembly and completeness evaluation

After sequencing by Nanopore three-generation sequencing, correction by Canu, assembly by SMARTdenovo and polishing by Racon, Pilon software, a total of 769.44 Mb of *A. nanchuanensis* genome sequences was generated with 1087 contigs, a 2.09 Mb contig N50 and a 402 kb contig N90 (Table 2). The contig N50/ N90 and scaffold N50/ N90 of *M. notabilis* were 34,476 bp/2,231 bp and 390,115 bp/11,563 bp; contig N50/ N90 and scaffold N50/ N90 of *B. papyrifera* were 171.17 kb/ 38.90 kb and 29.48 Mb/17.97 Mb; contig N50/ N90 of *F. microcarpa* were 907,868 bp/113,961bp (Table 3). Compared with other reported moraceae plants, *A. nanchuanensis* has a bigger genome size with a better assembly quality (Table 2).

Statistical alignment analysis of second-generation sequencing reads showed that clean reads located on the reference genome accounted for 99.41% of the total clean reads (363,371,475/365,545,724). The paired-end sequences of the correct size that were located on the reference genome, accounted for 93.56% of the total clean reads (341,995,184/365,545,724). The core gene integrity assessment was performed by CEGMA v2.59. Here, 445 CEGs were present in assembly, accounting for 97.16% of all CEGs (445/458), while 232 highly conserved CEGs were present in the assembly, accounting for 93.55% of all CEGs (232/248). The database in BUSCO v4.0.6 contains 1,614 conserved core genes, and the number of complete genes present in the assembly is 1583 (98.08%); details are shown in Fig. 4.

3.3 Hybrid assembly, scaffolding, and chromosome anchoring

We obtained 137.5 Gb clean Hi-C data (approximately 62 × depth of the estimated genome). The clean Hi-C reads accounted for 179-fold coverage of the 769.44 Mb genome estimated by the Illumina platform for subsequent analysis (Table 1). To assess the quality of Hi-C data, we performed an insertion fragment length assessment, which showed a relatively narrow unimodal length distribution with the highest peak at approximately 300 bp, indicating that the dispersion degree of the inserted fragment length was small, the inserted fragment size was normal and the purification of magnetic beads during library construction functioned efficiency (Fig.

5). A total of 728,487,984 paired reads were genome-related mapping reads, accounting for 79.37% of the clean data. A total of 236,274,160 paired reads were uniquely mapped on the genome assembly, including 56,964,635 valid Hi-C paired reads. Details are shown in Table 2 and Supplementary Table 1, 2. Alignment efficiency, insert fragment length and effective Hi-C data volume evaluation all indicated that the Hi-C libraries were constructed well.

After Hi-C assembly and manual adjustment, a total of 766.50 Mb of genomic sequences were located on 28 chromosomes through scaffold correction, clustered, ordered and orientated, accounting for 99.62% of all genomic sequences, and the corresponding number of sequences was 1,336 (97.95%). Among the sequences located on the chromosome, the sequence length based on order and direction was 697.71 Mb, accounting for 91.02% of the total length of the sequences on the chromosomes (Table 4). The contig N50 and Scaffold N50 were 1.78 Mb and 25.15 Mb, respectively, after error correction (Table 1). The final pseudochromosomes were constructed after manual adjustment.

The genomes of *A. nanchuanensis* and *Ficus.microcarpa* (*F. microcarpa*) were compared to verify the accuracy of the overlap across the 28 chromosomes, and the collinearity circle diagram indicates a high similarity of genes order between them (Fig. 2). A heatmap was drawn to evaluate the structure and quality of Hi-C assembly (Fig. 5). The figure indicated that the 28 pseudochromosomes could be distinguished easily and the interaction signal intensity at the diagonal was significantly stronger than that at other locations within each pseudochromosome.

3.4 Gene prediction and annotation

A total of 422.78 Mb (54.94%) of repeat sequences was detected; among these repeat elements, long terminal repeats (LTRs) were the predominant type, whereas Class I/LTR/Copia and Class I/LTR/Gypsy accounted for 19.17% (147.52 Mb) and 16.86% (129.74 Mb). The details of the repeat sequences are shown in Supplementary Table 3.

A total of 41,636 protein-coding genes were predicted with a 3,797.54 bp average gene length, a 1,509.16 bp average exon length, and a 2,288.38 bp average intron length by ab initio-based, homologue-based, and RNA-seq-based methods (Table 5). Among the genes integrated by EVM, 27,262 genes were obtained by the three prediction methods (Supplementary Fig. 1). Based on GenBlastA v1.0.4 and GeneWise2.4.1, 1,905 pseudogenes were obtained, and their total length and average length were 4,825,668 kb and 2,533.16 kb, respectively (Supplemntary table 5).

A total of 39,596 genes were successfully annotated in the functional databases, accounting for 95.10% (39,596/41636) of the predicted genes; details are shown in Supplementary Table 7. According to the noncoding RNA prediction results, the number of miRNAs was 138, belonging to 24 RNA families; there were 409 rRNAs, belonging to 4 RNA families; and there were 512 tRNAs, belonging to 24 families (Supplementary Table 6).

The number of homologous genes between *A. nanchuanensis* and *M. notabilis* was 30,510, accounting for 77.14%, based on the Nr homologous species distribution, indicating high homology (Fig. 6). The KOG database is based on the phylogenetic relationships of protein-coding genes in bacteria, algae, and eukaryotes with complete genomes and classifies the gene products based on linear homology and at the functional level. A total of 21,567 (51.80%) *A. nanchuanensis* genes were annotated in the KOG database (Supplementary Table 7), and the annotation classification details are shown in Supplementary Fig. 2. The top three over expressed genes were mainly involved in posttranslational modification, protein turnover, chaperones, signal transduction mechanisms and transcription. The GO database was used to define and describe the genes and proteins, according to their involvement in biological processes, the components that make up cells, and the molecular functions they perform (Supplementary Fig. 3). Annotated gene number and repeat sequence size of *A. nanchuanensis* were 41,636 and 422.78Mb, that are bigger than that of in previously reported *M. notabilis* (29,338, 127.98 Mb), *F. microcarpa* (29,416, 198.23 Mb) and *B. papyrifera* (30,512, 190.23 Mb), indicating the high quality of sequencing and annotation for *A. nanchuanensis* (Table 3).

Nucleotide-binding site and leucine-rich repeat (NBS-LRR) has been well known as major plants disease resistance gene, the gene number of NBS-LRR in papaya, watermelon, Arabidopsis, grape, tomato, and notabilis were 55, 44, 166, 251, and 142 respectively, while *A. nanchuanensis* is 10[50,52]. As particular NBS-LRR genes recognize specific pathogen effectors, fewer the number of NBS-LRR genes may represent less potential for pathogen recognition, indicate that the NBS-LRR genes of *A. nanchuanensis* are not under strong selection pressure, may due to fewer pathogens were evolved to *A. nanchuanensis* adaption. For minimize the dangers of insect infestation, plants evolved a defence mechanism by expressing plant protease inhibitors (PIs) to interfere digestive systems of insects, and 8 Glu S.griseus protease inhibitor genes were detected in *A. nanchuanensis*[49]. PIs and NBS-LRR genes are

reasonably important for defense response in *A. nanchuanensis* ancient species.

KEGG is the main public database of pathway, and 129 metabolic pathways of *A. nanchuanensis* were finally obtained. Plant-pathogen interaction metabolic pathways may closely relate to the resistance of disease and insect pests. Abundant biosynthesis pathways of Vitamins, flavonoid and gingerol may reveal the theoretical basis of *A. nanchuanensis* rare medicinal value.

3.5 Comparative genomics

The protein sequences between *A. nanchuanensis* and its related species (*A. thaliana*, *A. trichopoda*, *P. trichocarpa*, *A. chinensis*, *V. vinifera*, *M. notabilis*, and *T. cacao*) were compared, and 33,925 genes out of 41,636 *A. nanchuanensis* predicted genes were clustered into 15,436 gene families, of which 512 were unique to *A. nanchuanensis* (Table 6 and Supplementary Fig. 4). In the phylogenetic tree of *A. nanchuanensis* and its related species, *A. nanchuanensis* diverged from *M. notabilis* approximately 0.5285 million years ago (Mya) by Mcmctree estimation, diverged from *A. chinensis* and *V.vinifera* approximately 19.3794 Mya and from *A. thaliana, T. cacao, and P. trichocarpa* approximately 18.6558 Mya, which support the close relationship between A. *nanchuanensis and M. notabilis* (Fig. 7). This result was confirmed by the analysis of homologous species distribution, transversions at fourfold degenerate sites (4DTv) and chromosome genes order.

In the evolutionary process, gene families in contraction and expansion generally implies strong functional changes. According to the evolutionary relationships among species and the results of gene family clustering, 309 gene families in contraction and 559 gene families in expansion were detected in *A. nanchuanensis* after divergence from mulberry (Fig. 7). These gene families in contraction are mainly related to F-box domain, cystatin domain, protein kinase domain and ring finger domain functions (Table 7). Refers to the common ancestor, except for *A. thaliana* and *P. trichocarpa*, the number of gene families in contraction is bigger than that of in expansion among other species, suggesting that more gene families in most species experienced contraction than expansion during adaptive evolution, and the living environment of *A. thaliana* and *P. trichocarpa* may be challengeable, that expand their gene family to cope with the living environment.

EVM0035972.1, EVM0031735.1, EVM0026117.1 and EVM0015119.1 were found to be rapidly evolving genes, and details on these genes and their annotated functions are shown in Table 8 and Supplementary Fig. 5. 4DTv are neutral genetic distances that can be used to estimate the relative timing of evolutionary events[60].

According to the homologous gene pairs between two species or within species themselves, the ratio of each homologous gene to the 4DTV mutation site was calculated, and a 4DTV distribution map was made (Fig. 8). The peak of the 4DTv distribution among *A. nanchuanensis* and *M. notabilis* was closer to the current than that of *A. nanchuanensis* and other species, indicating that the differentiation time of *A. nanchuanensis* and *M. notabilis* appeared recently, suggesting a closer genetic relationship between them. At ancient time, the 4DTV distribution curves of *A. nanchuanensis* and other species were similar, which reflected these species might share similar whole-genome duplication (WGD) events. Moreover, the 4DTV distribution of *A. nanchuanensis* had a small peak at 0.05, which suggested that some genomic fragments duplicated recently.

LTR accumulation is able to reflect that the species may cope with some environmental stresses on its survival[20]. LTR insertion time among *A. nanchuanensis* and other 7 related species show that the living environment of *A. nanchuanensis* is relatively stable. The narrow peak of LTR insertion time around 1 Mya indicated some environment stress or environment change has been imposed on *A. nanchuanensis* and its living environment (Fig. 8).

4. Conclusion

In this study, a high-quality genome assembly and annotation information for *A. nanchuanensis* were first reported, resulting in the first reference genome for the Artocarpus genus. A total of 123.38 Gb of clean reads were obtained and a 769.44 Mb genome was assembled, which was larger than that of the sequenced *M. notabilis* and *B. papyrifera*. The clean reads mapped percentage (99.41%), CEGs and highly conserved CEG present in assemblies (97.16%, 93.55%), and BUSCO conserved gene core set coverage (98.08%) indicated that the current assembly covers most of the *A. nanchuanensis* genome; The *A. nanchuanensis* genome size estimated by k-mer analysis was 761.07 Mb, and the assembly was 769.44 Mb; These data suggested that this assembly was mostly representative of the complete *A. nanchuanensis* genome and indicated the high quality of *A. nanchuanensis* genome assembly. *A. nanchuanensis, F. microcarpa* and *M. notabilis* are both composed of 7 chromosome pairs, and their high similarity in genes order indicated high continuity between *A. nanchuanensis, F. microcarpa* and *M. notabilis,* as well as the high quality of the *A. nanchuanensis* genome assembly.

A small number of NBS-LRR and PIs plants disease and insect resistance genes were detected in the gene prediction and annotation analysis of *A. nanchuanensis*,

indicating that no strong selection pressure was applied to the species, possibly due to fewer pathogens and insects were evolved to *A. nanchuanensis* adaption. Several anti-inflammatory metabolism and anti-inflammatory substance synthesis pathways were detected, which may be related to the unique antiallergic function of *A. nanchuanensis*. Study of relevant functions and metabolic pathways reveal fruit maturation, nutrient metabolism, disease resistance of *A. nanchuanensis*.

Gene families in contraction and expansion generally implies strong functional changes, unigene indicate special species function, the in-depth study of above gene provides the research foundation for *A. nanchuanensis* unique features. Meanwhile, the small number of disease and insect resistance genes, and the LTR insertment analysis may indicate the stability of ecological environment that *A. nanchuanensis* living in. Species genome analysis not only reveal their functions and evolutionary relationships, but also reflect their growth environment.

This high-quality genome of *A. nanchuanensis* will lay a solid foundation for the conservation, rational development, and utilization of critically endangered species in the future. It is a valuable resource for the genetic improvement and better understanding of *A. nanchuanensis* genomic evolution. This genome will also be invaluable in developing new varieties and addressing issues of agronomic and/or biological importance such as fruit development and maturation, nutrient metabolism of fruits, and disease resistance of *A. nanchuanensis* and related plant spices.

Acknowledgements

This work was supported by Key Laboratory of Bio-Resources and Eco-Environment of Ministry of Education, College of Life Sciences, Sichuan University, Chengdu 610065, Sichuan, P.R.China. and Chongqing Nanchuan biotechnology research institute, Bio-resource Research and Utilization Joint Key Laboratory of Sichuan and Chongqing, Sichuan and Chongqing, P.R.China.

References

1. Rong-, L. I. U. Studies on Chemical Constituents Occurring in Twigs of Artocarpus nanchuanensis. 2–6 (2013).
2. Ren, G. *et al.* Chemical constituents from the fruiting branches of Artocarpus nanchuanensis endemic to China. *Biochem. Syst. Ecol.* **51**, 98–100 (2013).
3. He, N. *et al.* Draft genome sequence of the mulberry tree Morus notabilis. (2013). doi:10.1038/ncomms3445
4. Peng, X. *et al.* A Chromosome-Scale Genome Assembly of Paper Mulberry (Broussonetia papyrifera) Provides New Insights into Its Forage and Papermaking Usage. *Mol. Plant* **12**, 661–677 (2019). doi.org/10.1016/j.molp.2019.01.021.
5. Sevim, V. *et al.* Shotgun metagenome data of a defined mock community using

Oxford Nanopore, PacBio and Illumina technologies. *Sci. Data* **6**, 1–9 (2019).
6. Branton, D. *et al.* The potential and challenges of nanopore sequencing. *Nat. Biotechnol.* **26**, 1146–1153 (2008).
7. Belton, J. M. *et al.* Hi-C: A comprehensive technique to capture the conformation of genomes. *Methods* **58**, 268–276 (2012).
8. van Berkum, N. L. *et al.* Hi-C: A method to study the three-dimensional architecture of genomes. *J. Vis. Exp.* 1–7 (2010). doi:10.3791/1869
9. Gawel, N. J. & Jarret, R. L. A Modified CTAB DNA Extraction Procedure for Musa and Ipomoea. Plant Mol. Biol. 9, 262–266 (1991).
10. Bian, L. *et al.* Chromosome- level genome assembly of the greenfin horse- faced filefish (Thamnaconus septentrionalis) using Oxford Nanopore PromethION sequencing and Hi- C technology . *Mol. Ecol. Resour.* 1–25 (2020). doi:10.1111/1755-0998.13183
11. Rao, S. S. P., Huntley, M. H., Durand, N. C. & Stamenova, E. K. Article A 3D Map of the Human Genome at Kilobase Resolution Reveals Principles of Chromatin Looping. *Cell* 1–16 (2014). doi:10.1016/j.cell.2014.11.021
12. Koren, S. et al. Canu: scalable and accurate long-read assembly via adaptive k-mer weighting and repeat separation. Genome Res. 27, 722–36 (2017)
13. Vaser, R., Sovi, I., Nagarajan, N. & Šiki, M. Fast and accurate de novo genome assembly from long uncorrected reads. Genome Research (2017) doi:10.1101/gr.214270.1162017.
14. Walker, B. J. *et al.* Pilon : An Integrated Tool for Comprehensive Microbial Variant Detection and Genome Assembly Improvement. **9**, (2014).
15. Li, H. & Durbin, R. Fast and accurate short read alignment with Burrows – Wheeler transform. **25**, 1754–1760 (2009).
16. Parra, G., Bradnam, K. & Korf, I. Genome analysis CEGMA : a pipeline to accurately annotate core genes in eukaryotic genomes. **23**, 1061–1067 (2007).
17. Simão, F. A., Waterhouse, R. M., Ioannidis, P. & Kriventseva, E. V. BUSCO : assessing genome assembly and annotation complete- ness with single-copy orthologs. 9–10 (2015).
18. Servant, N. *et al.* HiC-Pro : an optimized and flexible pipeline for Hi-C data processing. 1–11 (2015). doi:10.1186/s13059-015-0831-x
19. Burton, J. N. *et al.* Chromosome-scale scaffolding of de novo genome assemblies based on chromatin interactions. (2013). doi:10.1038/nbt.2727
20. Xu, Z. & Wang, H. LTR _ FINDER : an efficient tool for the prediction of full-length LTR retrotransposons. **35**, 265–268 (2007).
21. Price, A. L., Jones, N. C. & Pevzner, P. A. De novo identification of repeat families in large genomes. **21**, 351–358 (2005).
22. Abel, L. W. Planning a dynamic kill. *JPT, J. Pet. Technol.* **48**, 422–426 (1996).
23. Jurka, J. *et al.* Diversity of Retrotransposable Elements Repbase Update , a database of eukaryotic repetitive elements. **467**, 462–467 (2005).
24. Tarailo-graovac, M. & Chen, N. Using RepeatMasker to Identify Repetitive Elements in Genomic Sequences. 1–14 (2009). doi:10.1002/0471250953.bi0410s25
25. Burge, C. & Karlin, S. Prediction of Complete Gene Structures in Human Genomic DNA. 78–94 (1997).
26. Stanke, M. & Waack, S. Gene prediction with a hidden Markov model and a new intron submodel. **19**, 215–225 (2003).
27. Majoros, W. H., Pertea, M. & Salzberg, S. L. TigrScan and GlimmerHMM : two open source ab initio eukaryotic gene-finders. **20**, 2878–2879 (2004).
28. Blanco, E., Parra, G. & Guigó, R. Using geneid to Identify Genes. *Curr. Protoc. Bioinforma.* 1–28 (2007). doi:10.1002/0471250953.bi0403s18
29. Korf, I. Gene finding in novel genomes. BMC Bioinformatics. **9**, 1–9 (2004).
30. Keilwagen, J. *et al.* Using intron position conservation for homology-based gene prediction. 1–11 (2016). doi:10.1093/nar/gkw092
31. Keilwagen, J., Hartung, F., Paulini, M., Twardziok, S. O. & Grau, J. Combining RNA-seq data and homology-based gene prediction for plants , animals and fungi. (2018).

32. Kim, D., Langmead, B. & Salzberg, S. L. HISAT : a fast spliced aligner with low memory requirements. *Nat. Methods* (2015). doi:10.1038/nmeth.3317
33. Pertea, M. *et al.* StringTie enables improved reconstruction of a transcriptome from RNA-seq reads. (2015). doi:10.1038/nbt.3122
34. Tang, S., Lomsadze, A., Borodovsky, M. & Tech, J. G. Identification of protein coding regions in RNA transcripts. **43**, 1–10 (2015).
35. Campbell, M. A., Haas, B. J., Hamilton, J. P., Mount, S. M. & Buell, C. R. Comprehensive analysis of alternative splicing in rice and comparative analyses with Arabidopsis. **17**, 1–17 (2006).
36. Haas, B. J. *et al.* Open Access Automated eukaryotic gene structure annotation using EVidenceModeler and the Program to Assemble Spliced. **9**, 1–22 (2008).
37. Griffiths-jones, S. *et al.* Rfam : annotating non-coding RNAs in complete genomes. **33**, 121–124 (2005).
38. Lowe, T. M. & Eddy, S. R. tRNAscan-SE : a program for improved detection of transfer RNA genes in genomic sequence. **25**, 955–964 (1997).
39. She, R., Chu, J. S., Wang, K., Pei, J. & Chen, N. genBlastA : Enabling BLAST to identify homologous gene sequences. 143–149 (2009). doi:10.1101/gr.082081.108.4
40. Birney, E., Clamp, M. & Durbin, R. GeneWise and Genomewise. 988–995 (2004). doi:10.1101/gr.1865504.quickly
41. Marchler-bauer, A. *et al.* CDD : a Conserved Domain Database for the functional annotation of proteins. **39**, 225–229 (2011).
42. Koonin, E. V *et al.* A comprehensive evolutionary classification of proteins encoded in complete eukaryotic genomes. Genome Biology. **5**, (2004).
43. Dimmer, E. C. *et al.* The UniProt-GO Annotation database in 2011. **40**, 565–570 (2012). doi:10.1093/nar/gkr1048.
44. Kanehisa, M. & Goto, S. KEGG : Kyoto Encyclopedia of Genes and Genomes. Nucleic Acids Research. **28**, 27–30 (2000).
45. Boeckmann, B. *et al.* The SWISS-PROT protein knowledgebase and its supplement TrEMBL in 2003. Nucleic Acids Research. **31**, 365–370 (2003). doi: 10.1093/nar/gkg095.
46. Altschup, S. F., Gish, W., Pennsylvania, T. & Park, U. Basic Local Alignment Search Tool 2Department of Computer Science. J. Mol. Biol. 403–410 (1990).
47. Wang, J. *et al.* Genome-Wide Analysis of the Distinct Types of Chromatin Interactions in Arabidopsis thaliana. Plant Cell Physiol. **2**, 57–70 (2017).
48. DePamphilis, C. W. *et al.* The Amborella genome and the evolution of flowering plants. *Science (80-.).* **342**, (2013).
49. Tuskan, G. A. *et al.* The genome of black cottonwood, Populus trichocarpa (Torr. & Gray). *Science (80-.).* **313**, 1596–1604 (2006).
50. Huang, S. *et al.* Draft genome of the kiwifruit Actinidia chinensis. *Nat. Commun.* **4**, (2013). doi: 10.1038/ncomms3640.
51. Zhang, J. *et al.* Stress response proteins' differential expression in embryogenic and non-embryogenic callus of Vitis vinifera L. cv. Cabernet Sauvignon-A proteomic approach. *Plant Sci.* **177**, 103–113 (2009).
52. He, N. *et al.* Draft genome sequence of the mulberry tree Morus notabilis. *Nat. Commun.* **4**, (2013). doi: 10.1038/ncomms3445.
53. Argout, X. *et al.* The genome of Theobroma cacao. *Nat. Genet.* **43**, 101–108 (2011). doi:10.1038/ng.736.
54. Li, L. *et al.* OrthoMCL : Identification of Ortholog Groups for Eukaryotic Genomes OrthoMCL : Identification of Ortholog Groups for Eukaryotic Genomes. 2178–2189 (2003). doi:10.1101/gr.1224503
55. Uindon, P. G. & Ranc, J. E. A. N. New Algorithms and Methods to Estimate Maximum-Likelihood Phylogenies : Assessing the Performance of PhyML 3 . 0. **59**, 307–321 (2010).
56. Bie, T. De, Cristianini, N., Demuth, J. P. & Hahn, M. W. CAFE : a computational tool for the study of gene family evolution. Bioinformatics. **22**, 1269–1271 (2006).
57. Schabauer, H., Valle, M., Pacher, C. & Stockinger, H. SlimCodeML : An

Optimized Version of CodeML for the Branch-Site Model. (2012). doi:10.1109/IPDPSW.2012.88
58. Prestridge, D. S. SIGNAL SCAN : a computer program that scans DNA sequences for eukaryotic transcriptional elements. **7**, 203–206 (1991).
59. Edgar, R. C., Drive, R. M. & Valley, M. MUSCLE : multiple sequence alignment with high accuracy and high throughput. **32**, 1792–1797 (2004).
60. Montero-pau, De novo assembly of the zucchini genome reveals a whole genome duplication associated with the origin of the Cucurbita genus. J. et al. (2017) 0–2 doi:10.1111/ijlh.12860

Data Accessibility

The whole raw sequence reads produced by Illumina novaseq, Pacbio sequel II and ONT PromethlON Beta, have been deposited at NCBI Sequence Read Archive (SRA) under BioProject number PRJNA624965 and BioSample from SAMN14589993 for *A. nanchuanensis*. Raw sequencing data (Nanopore, Illumina, Hi-C, RNA-seq data) have been deposited in SRA database as SRR11671532, SRR11659666, SRR11659674, SRR11623450/SRR11668249.

Author contributions

J.H., S.B., X.D., K.K. and C.C. conceived and designed the study; J.H., S.B., X.D., J.D. X.W. and Q.L. collected the samples; Q.L., Y.Z. and Y.L. performed DNA sequencing and Hi-C experiments; Y.C. and L.F. performed RNA sequencing; J.H., Q.L. and Y.Z. estimated the genome size, assembled the genome, and assessed the assembly quality; Y.C. and L.F. performed the genome annotation and functional genomic analysis. S.X., J.H. and X.D. wrote the manuscript. All authors read, edited, and approved the final manuscript for submission.

Competing interests

The authors declare no competing interests.

Table

Table 1 The sequence statistics of *Artocarpus nanchuanensis.*

Table 2 Nanopore and Hi-C genome assembly statistics of Artocarpus nanchuanensis.

Table 3 The genomes assemblies quality comparison of *A.nanchuanensis* and its related Moraceae plants.

Table 4 The Hi-C assembly datastatistics table of Artocarpus nanchuanensis.

Table 5 The prediction analysis of *A. nanchuanensis* coding gene.

Table 6 Statistical classification of gene families.

Table 7 The annotation of protein gene family.

Table 8 The rapidly evolving genes selected by CodeML.

Figure

Fig. 1 The flowchart of *A. nanchuanensis* genome assembly and annotation process.

Fig. 2 The *A. nanchuanensis* sample and genomic interaction analysis.

Fig. 3 The Kmer distribution map of *A. nanchuanensis.*

Fig. 4 The BUSCO genome assembly evaluation.

Fig. 5 The analysis of Hi-C library construction and heat map.

Fig.6 The Nr homologous species distribution of *A. nanchuanensis*.

Supplementary Table

Supplementary Table 1 The clean data and genome comparison results of *A. nanchuanensis*.

Supplementary Table 2 The Hi-C sequencing data types and proportion of *A. nanchuanensis*.

Supplementary Table 3 The repeat sequences analysis of *A. nanchuanensis*.

Supplementary Table 4 The gene prediction results of *A. nanchuanensis*.

Supplementary Table 5 Pseudogene annotation statistics of *A. nanchuanensis*.

Supplementary Table 6 The statistical results of non-coding RNA.

Supplementary figure

Supplementary Fig. 1 Distribution of the number of genes among the three methods.

Supplementary Fig. 2 The KOG functional annotation classification of *A. nanchuanensis*.

Supplementary Fig. 3 The GO secondary node annotation classification of *A. nanchuanensis.*

Supplementary Fig. 4 The family clustering statistics among different species.

Supplementary Fig. 5 The classification annotation statistics for GO.

 Click here to access/download;Table;table.docx

Table 1 The sequence statistics of *Artocarpus nanchuanensis.*

Illumina		Nanopore		Hi-c	
Data*	51.76 Gb	Data*	123.38Gb	Data*	137.5 Gb
Depth/genome coverage	68.01 X	Depth/genome coverage	160.34 X	Depth	62 X
Total Kmer	45,202,482,693	MaxLen	216,661 bp	Total Read Pairs	458,907,479
Genome	761.07 Mb	SeqNum	7,057,335	Genome	769.44 Mb
Heterozygosity	0.93%	N50Len	19,177 bp	Contig N50	1.78 Mb
Repeated	55.80%	N90Len	11,029 bp	Scaffold N50	25.15 Mb
Mapping rate	99.41%				

Note: Data* mean the data has been filter to be clean data; Depth/genome coverage means depth of sequencing data; MaxLen means the longest reads length of sequencing data; SeqNum means the total read number of sequencing data; N50Len means the N50 length of sequencing data reads; N90Len means the N90 length of sequencing data reads.

Table 2 Nanopore and Hi-C genome assembly statistics of Artocarpus nanchuanensis.

Nanopore assembly results		Hi-C Assembly results	
Contig number	1,087	Scaffold / Contig number	809 / 1,364
Contig length	769,440,982 bp	Scaffold / Contig length (bp)	769,496,482/769,440,982
Contig N50	2,094,024 bp	Scaffold / Contig N50 (bp)	25,150,906/1,778,064
Contig N90	402,757 bp	Scaffold /Contig N90 (bp)	20,179,149 / 200,000
Contig max	8,879,419 bp	Scaffold / Contig max (bp)	32,505,427/ 8,646,128
		Gap total length (bp)	55,500
		GC content (%)	32.34

Note: Contig represents the contig after error correction. Scaffold represents the scaffold generated after connection, and scaffold length exceeds 1 Kb. Scaffold/Contig number represents the number of scaffold and contig in the scaffold; Scaffold/Contig length represents the length of scaffold and contig in the scaffold; Scaffold/Contig N50 represents length of scaffold N50 and contig N50; Scaffold/Contig N90 represents length of scaffold N90 and contig N90; Scaffold / Contig max represents the length of the longest scaffold and longest contig; GC content represents the GC content percentage.

Table 3 The genomes assemblies quality comparison of *A.nanchuanensis* and its related Moraceae plants.

Genus names	Latin name	Sequencing technology	Sequencing depth	Contig/Scaffold N50	Contig/Scaffold N90	Annotated genes	Repeat composition
Artocarpus	*A.nanchuanensis*	Illumina, Nanopore, Hi-C	160.34 X (123.83 Gb, Nanopore)	2.09 Mb/25.15 Mb	402.76 Kb/20.18 Mb	41,636	422.78Mb
Morus Linn	*Morus notabilis*	Illumina HiSeq 2000	236.82 X (78.34 Gb, Illumina)	34,476 bp/390,115 bp	2,231 bp/11,563 bp	29,338	127.98Mb
Ficus	*Ficus microcarpa*	Illumina, PacBio RS II, Hi-C	86.55 X (36.87 Gb, Pacbio)	907,868 bp / None	113,961bp / None	29,416	198.23Mb
Broussonetia	*Broussonetia papyrifera*	Illumina, PacBio RS II, Hi-C, Bionano, genetic maps	22.47 X (8.54 Gb, Pacbio)	171.17 kb/29.48 Mb	38.90 kb/17.97 Mb	30,512	190.23 Mb .

Table 4 The Hi-C assembly datastatistics table of Artocarpus nanchuanensis.

Group	Cluster number	Cluster length (bp)	Order number	Order length (bp)
LG01	46	26,514,107	24	24,676,255
LG02	41	26,638,661	16	24,489,134
LG03	30	24,254,703	16	23,044,270
LG04	34	22,404,888	13	20,644,200
LG05	33	21,646,681	16	20,177,649
LG06	35	29,133,579	18	27,822,153
LG07	69	32,924,820	27	29,467,719
LG08	45	29,858,101	20	27,605,363
LG09	77	29,556,483	29	25,185,028
LG10	45	22,896,788	20	20,243,522
LG11	67	25,833,105	20	21,750,724
LG12	37	24,385,337	15	22,370,729
LG13	47	23,481,896	24	21,098,278
LG14	46	29,162,015	19	26,857,340
LG15	61	28,431,484	30	25,341,045
LG16	32	21,965,556	16	20,879,538
LG17	41	25,915,114	19	24,032,910
LG18	49	34,941,454	27	32,502,827
LG19	54	29,520,137	21	25,685,935
LG20	50	32,513,478	18	29,815,261
LG21	50	28,639,915	21	25,613,043
LG22	42	27,392,871	24	25,873,084
LG23	42	28,655,389	16	26,447,344
LG24	52	27,753,222	24	25,148,606
LG25	46	23,720,417	16	21,152,151
LG26	63	33,995,937	28	30,220,329
LG27	58	28,458,315	24	25,577,647
LG28	44	25,907,258	22	23,985,053
Total (Ratio%)	1336 (97.95%)	766501711 (99.62%)	583 (43.64%)	697707137 (91.02%)

Note: the statistics do not include 100 Ns added by artificially connected pseudochromosomes.

Table 5 The prediction analysis of *A. nanchuanensis* coding gene.

Prediction style and proportion			
Gene Number	41,636	CDS length	50,445,441 bp
Gene length	158,114,419 bp	CDS average length	1,211.58 bp
Gene average length	3,797.54 bp	CDS number	226,727
Exon length	62,835,343 bp	CDS average number	5.45
Exon average length	1,509.16 bp	Intron length	95,279,076 bp
Exon number	233,559	Intron average length	2,288.38 bp
Exon average number	5.61	Intron number	191,923
		Intron average number	4.61

Table 6 Statistical classification of gene families.

Name	Total gene	Cluster	Total family	Unifamily
A. thaliana	27,369	23,106	12,753	726
A. trichopoda	16,986	15,058	11,147	254
P. trichocarpa	41,335	33,270	14,725	950
A. chinensis	39,040	25,888	12,648	1,327
V. vinifera	26,346	19,238	12,682	665
M. notabilis	26,965	20,423	14,794	524
T. cacao	21,432	20,070	13,810	176
A. nanchuanensis	41,636	33,925	15,436	512

Note:Total gene: the number of total gene; Cluster: the number of genes that involved in family classification; Total family number: the number of gene families that can be divided; Uni family: the number of unique gene families.

Table 7 The annotation of protein gene family.

GeneFamily	Pfam	Function
GF_12673	PF00646.28	F-box domain
GF_10548	PF00031.16	Cystatin domain
GF_8	PF00069.20	Protein kinase domain
GF_13176	PF13639.1	Ring finger domain

Note: Gene family : the gene family cluster; Pfam: the ID of protein family alignment to the Pfam

database；Function：the function of the protein family that can be alignmented.

Table 8 The rapidly evolving genes selected by CodeML.

GeneID	P-value	Sites
EVM0035972.1	0.05	298,G,0.993**
EVM0031735.1	0.06	74,E,0.984*
EVM0026117.1	0.35	68,K,0.997**
EVM0015119.1	0.00	232,E,0.990**

Note: Gene ID mean the ID of gene, ω0 mean ka/ks for the studied Species, ω1 mean the average ka/ks for other species, ω2 mean ka/ks for the whole evolutionary tree.

 Click here to access/download;Figure;figure revised 10-27.docx

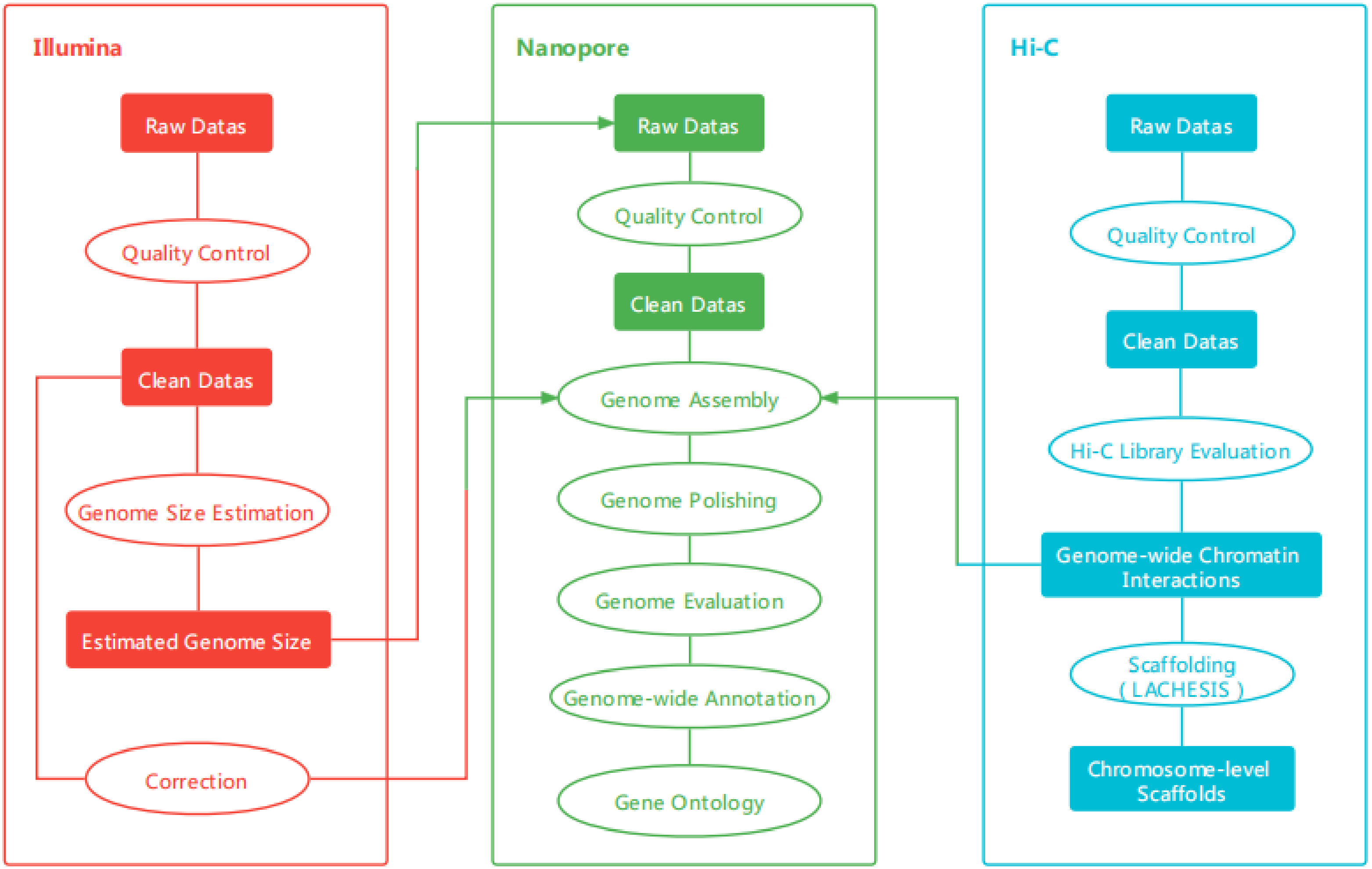

Fig. 1 The flowchart of *A. nanchuanensis* genome assembly and annotation process.

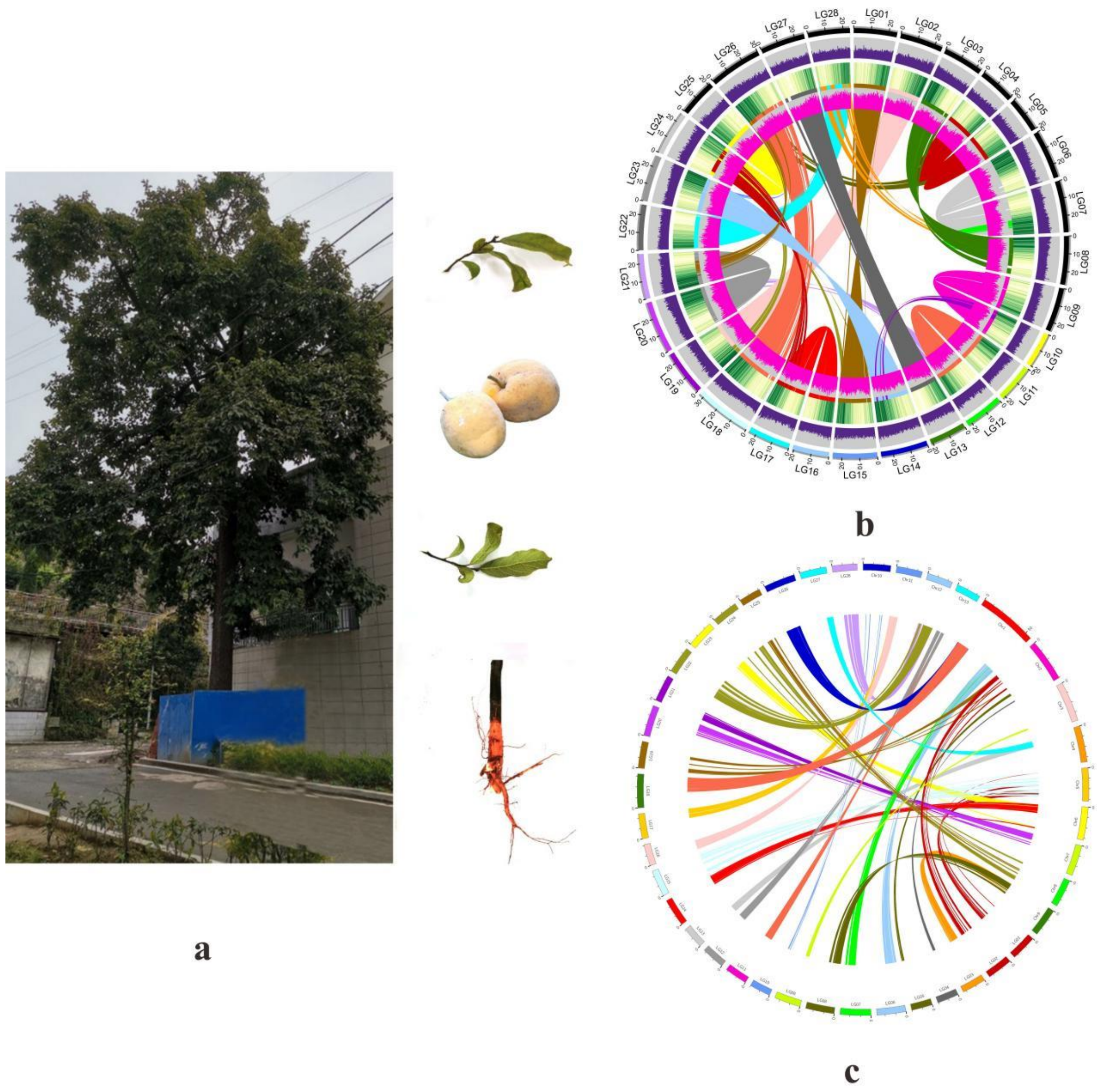

Fig. 2 The *A. nanchuanensis* sample and genomic interaction analysis.

Note: (a) The picture of the *A. nanchuanensis* tree study used in this study, and the picture was taken in 2019. (b) Circos plot of *A. nanchuanensis* 28 chromosomes. The tracks from outside to inside are 28 chromosome-level scaffolds, the GC content of chromosomes (purple), the gene density of chromosomes (green), the TE ratio of chromosomes (pink), and the lines of different colors in the innermost circle represent the collinearity within themselves. (c) Genes collinearity circle of *A. nanchuanensis* and *F. microcarpa*. Each color represents a collinear block of each chromosome, with at least five collinearity genes in each block.

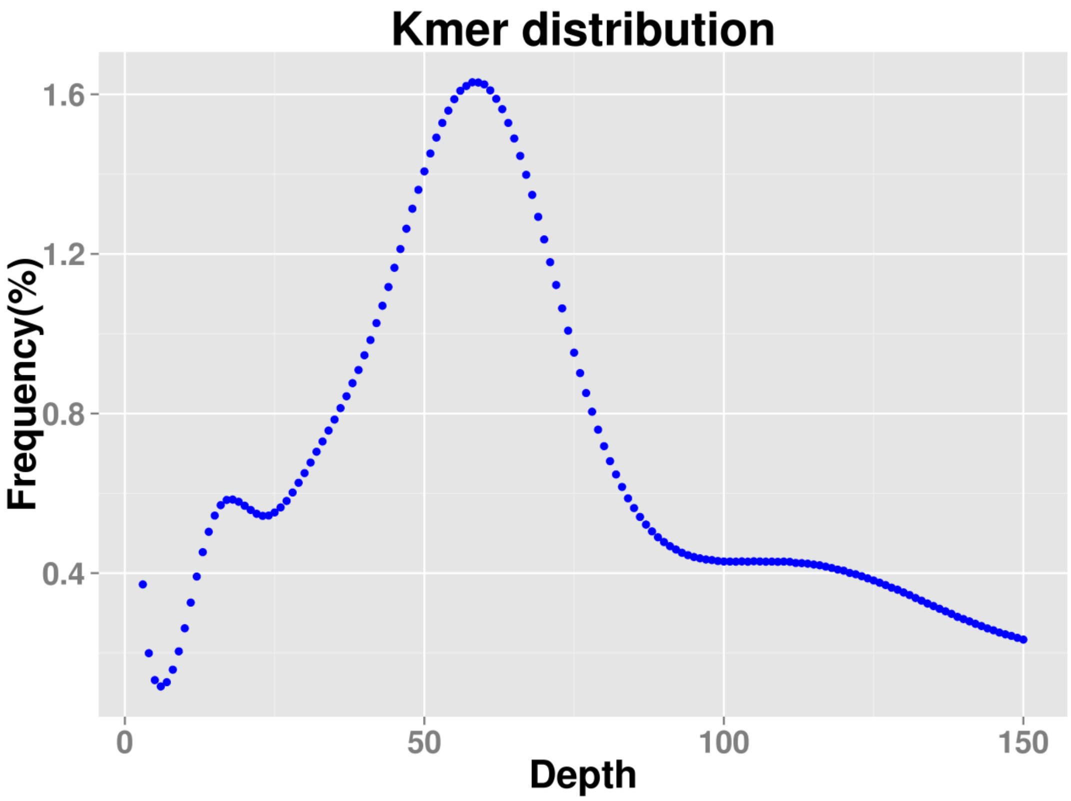

Fig. 3 The Kmer distribution map of *A. nanchuanensis.*

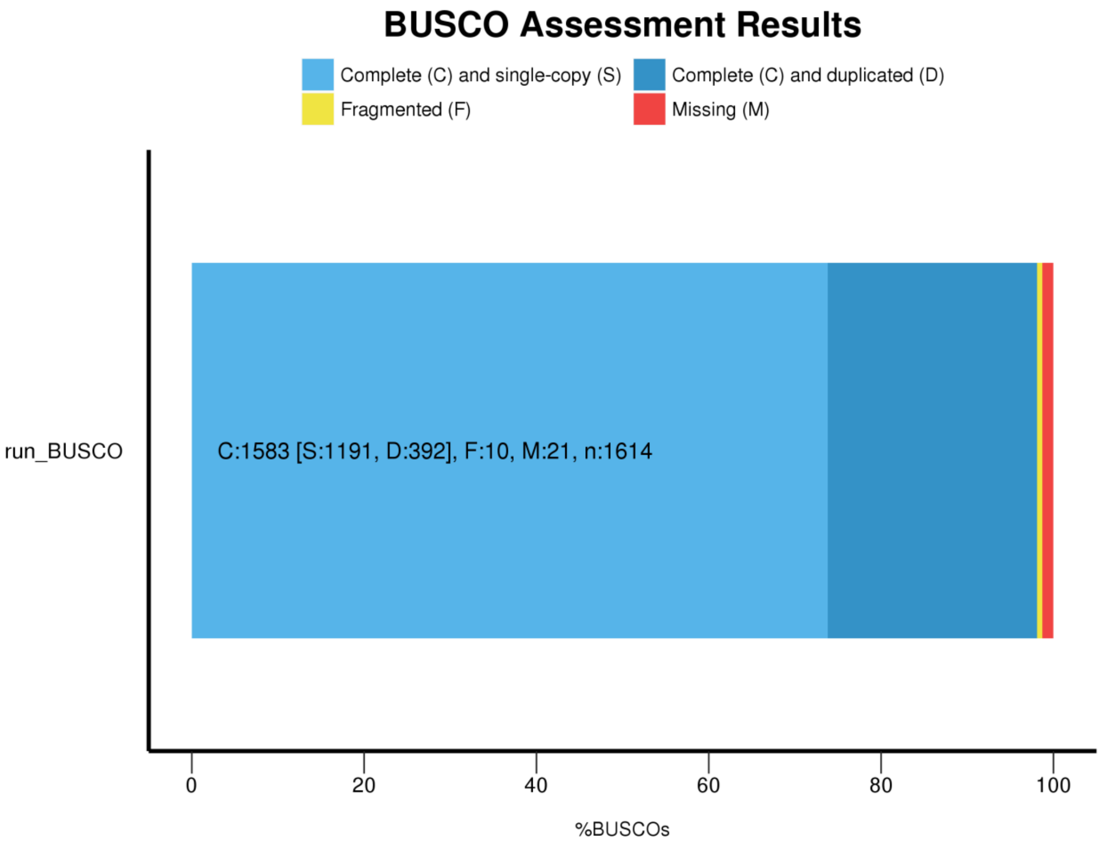

Fig. 4 The BUSCO genome assembly evaluation.

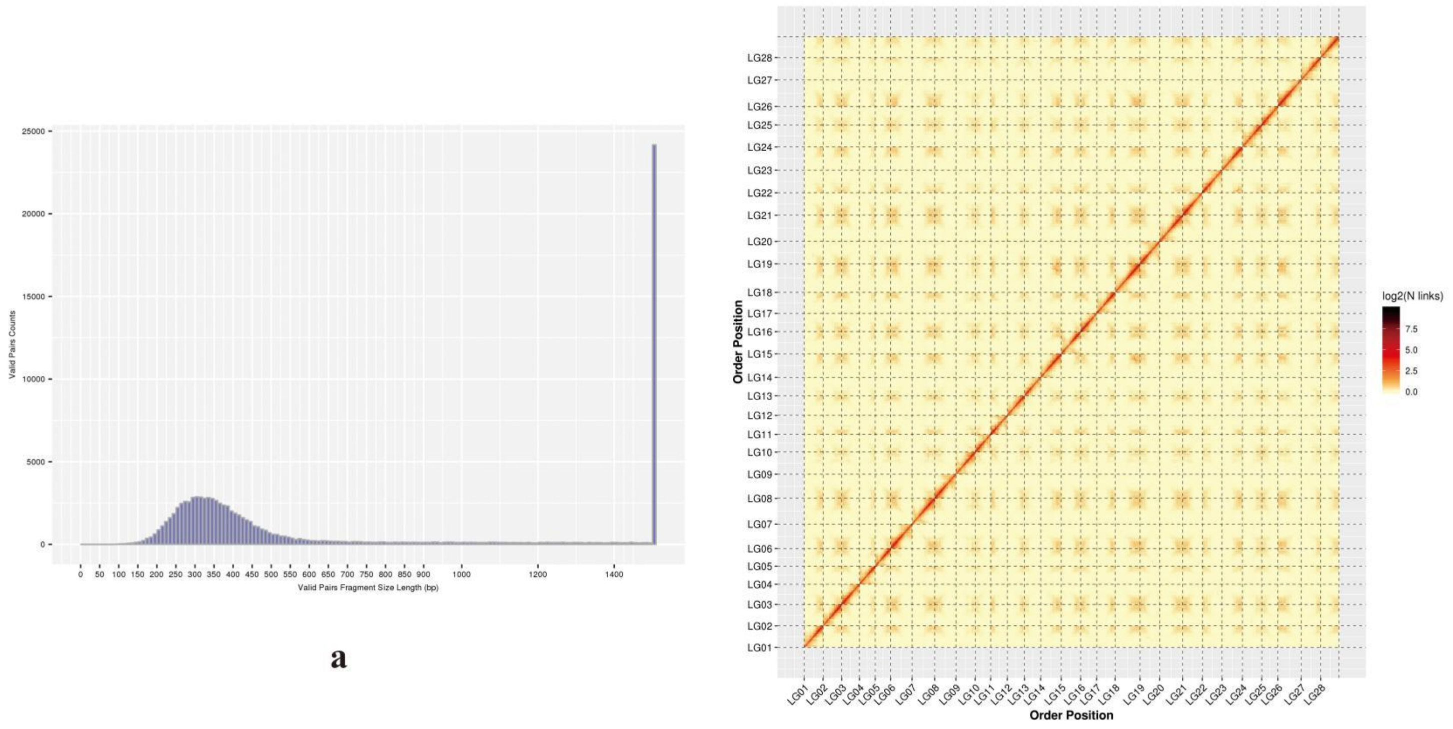

a

b

Fig. 5 The analysis of Hi-C library construction and heat map.

Note: a means the length distribution of Hi-C library insert fragment, X-axis represents the sum of the distance between double ends reads on the assembly genome and the nearest enzyme-cutting site, Y-axis represents the reads number. b means the interaction heat map of Hi-C links among chromosome groups for *A. nanchuanensi*s, the assembled genome of *A. nanchuanensis* was divided into 100-kb non-overlapping windows (or bins), and valid interaction links of Hi-C data were calculated between each pair of bins. The binary logarithm of each link number is coded using colors ranging from light yellow to dark red, indicating the frequency of Hi-C interaction links from low to high. LG01-LG28 represents the 28 chromosome groups inferred by LACHESIS; The X-axis and Y-axis represent the order of each bin on the corresponding chromosome group.

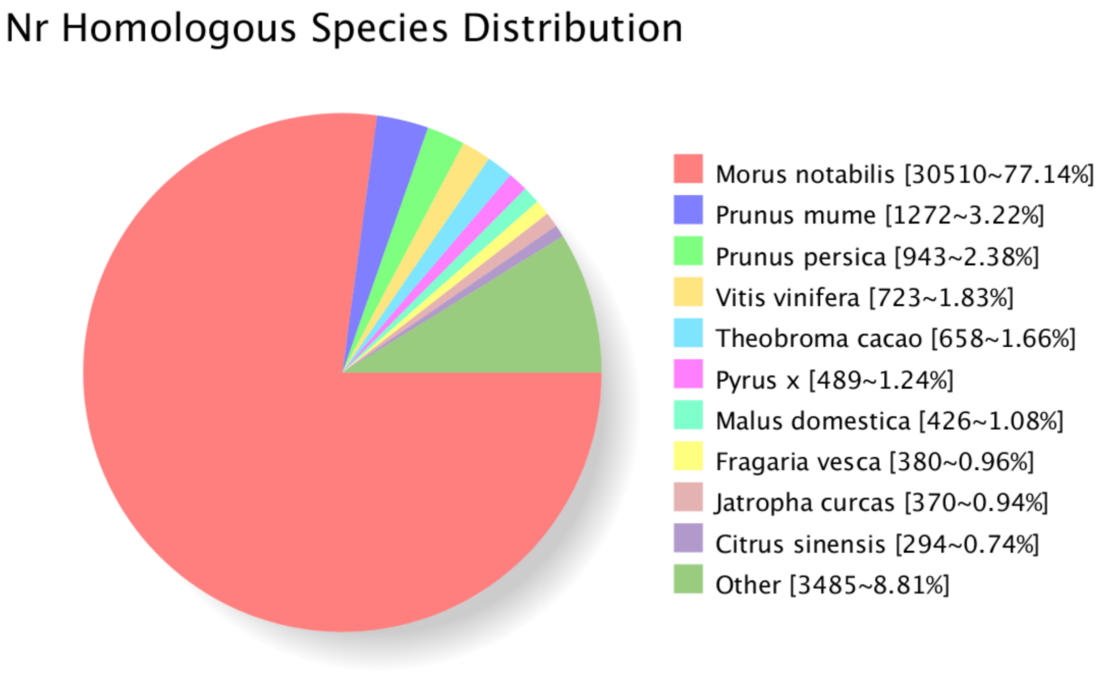

Fig.6 The Nr homologous species distribution of *A. nanchuanensis*.

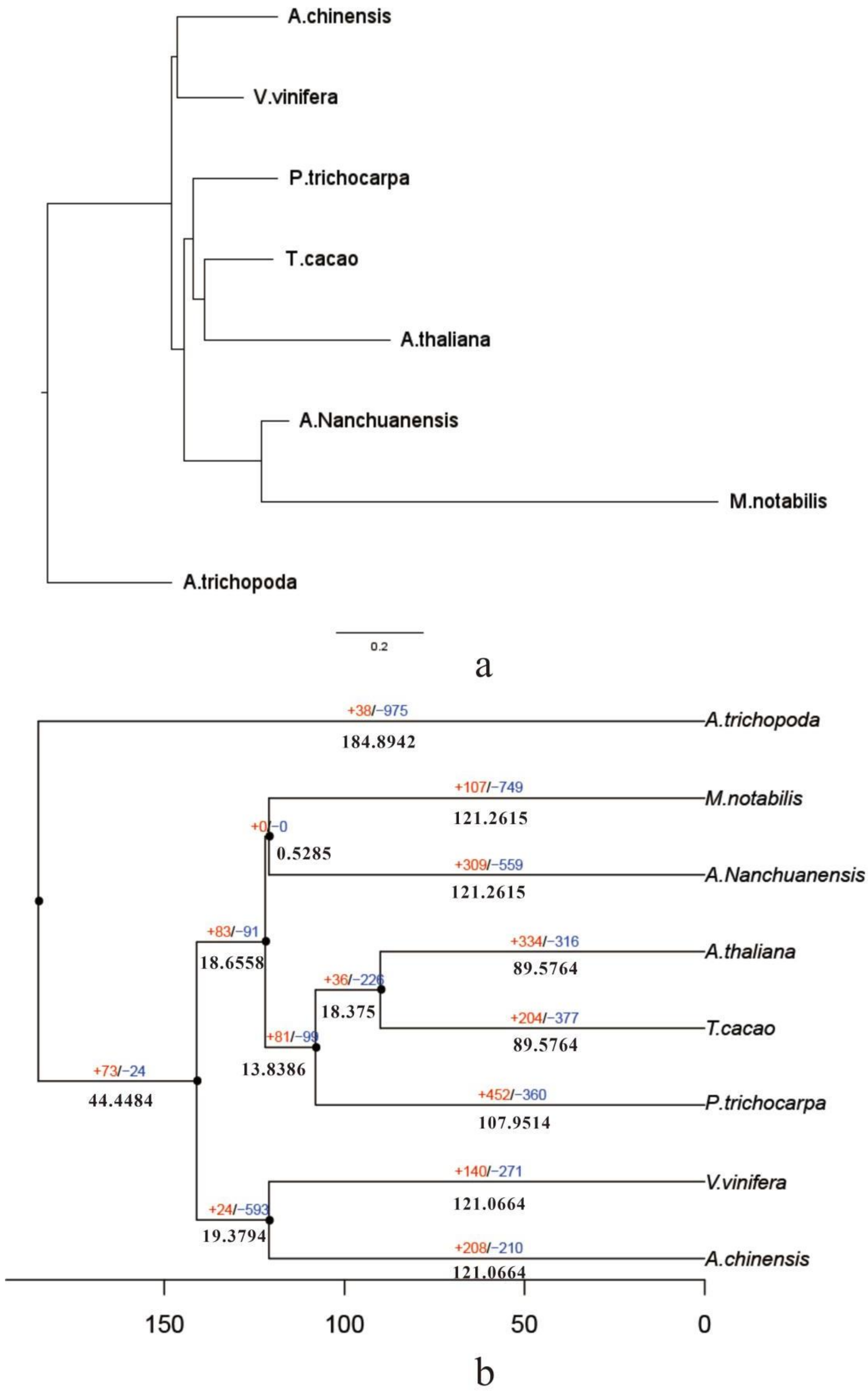

Fig.7 The phylogenetic and gene families analysis of *A. nanchuanensis* and related species.

Note: a represent species phylogenetic ananlysis of *A. nanchuanensis* and related species. b represent analysis of species differentiation time and gene families in contraction and expansion. black font represents the temporal relationship of species differentiation, unit is million years. "+" represents the number of gene families expanding on this node, "-" represents the number of gene families contracting on this node, and the black dot refers to the common ancestor.

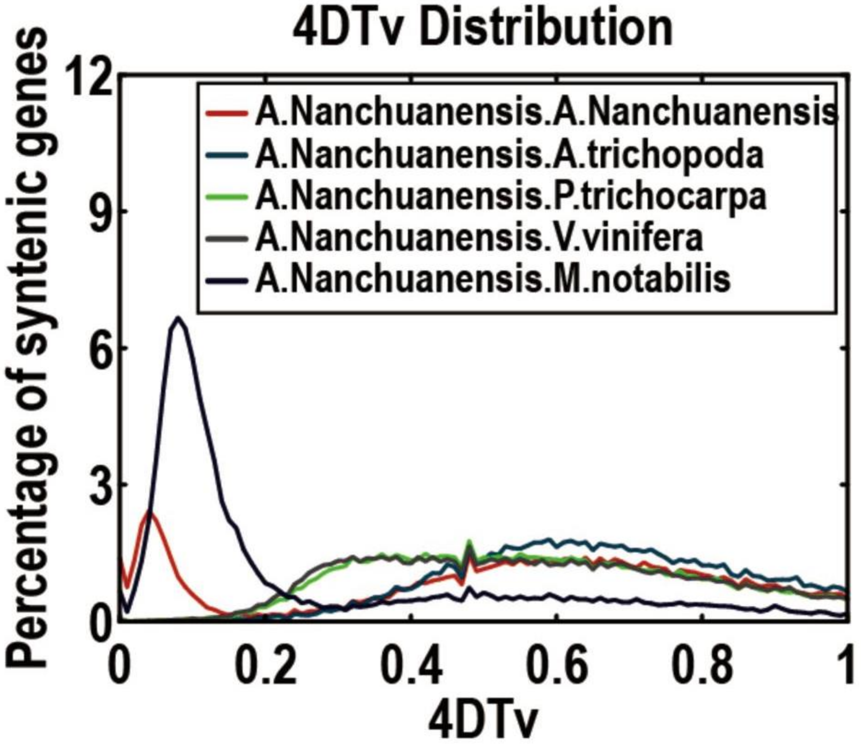

a

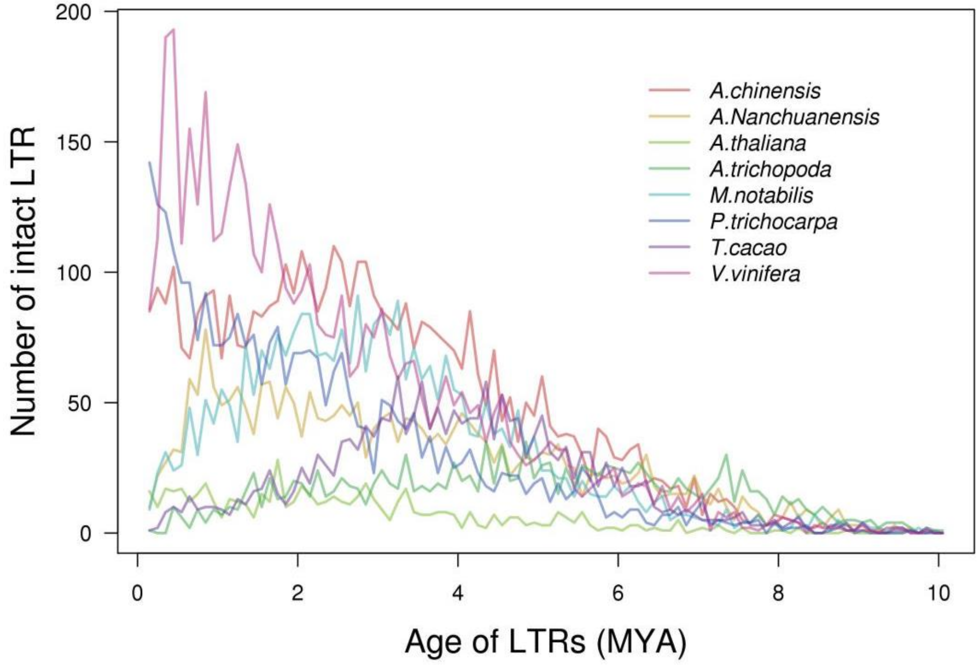

b

Fig. 8 The 4DTV distribution and LTR insertion time analysis among *A. nanchuanensis* and other related species.

Note: a represent 4DTV distribution analysis among A. nanchuanensis and other four species, the x axis represents the mutation rate of homologous genes to 4DTV, and the Y axis represents the proportion of homologous gene pairs. B represents the analysis of LTR insertion time among *A. nanchuanensis* and other seven species.

Click here to access/download
Supplementary Material
supplementary figure.docx

Click here to access/download
Supplementary Material
response for nanchuan 10-24.docx

Click here to access/download
Supplementary Material
Supplementary table .docx

Personal Cover Click here to access/download;Personal Cover;cover letter.docx

Dear Editors:

We would like to submit an original article entitled "Chromosome genome assembly and annotation of Artocarpus Nanchuanensis with Nanopore and Hi-C sequencing data" for consideration for publication in GigaScience.

The Artocarpus Nanchuanensis (Moraceae) is an extremely endangered tree species in China. Its fruit and bark have been used as the treatment for skin in Chongqing Nanchuan for a long time, and the fruit has a good control effect on the constipation and other intestinal diseases, those features persistent cause the attention of the researchers, but the molecular mechanisms involved is little known. In our work, we revealed a high-quality chromosome-scale genome assembly and annotation for Artocarpus Nanchuanensis with Nanopore and Hi-C sequencing data. The disclosure of Artocarpus Nanchuanensis genome sequence information provides an important resource to expand our understanding of the molecular mechanism in its unique biological processes and nutritional, medicinal benefits.

This article has not been published elsewhere in whole or in part. All authors have read and approved the content, and agree to submit for consideration for publication in GigaScience. There is not any conflict involved in the article. I hope this paper is suitable for GigaScience.

We deeply appreciate your consideration of our manuscript, and we look forward to receiving comments from the reviewers. If you have any queries, please don't hesitate to contact me at the address below.

With kind regards

Yours sincerely

Xianping Ding

Corresponding Author:

Xianping Ding

Key Laboratory of Bio-Resources and Eco-Environment of Ministry of Education, College of Life Sciences, Sichuan University, Chengdu 610065, Sichuan, P.R.China;

Tel. and Fax: +86-028-85413096; Email: brainding@scu.edu.cn
