# Supplementary material for: A chromosome-level genome assembly of Artocarpus nanchuanensis (Moraceae), an extremely endangered fruit tree
Source: Gigascience. 2022 Jun 14;11:giac042. doi: 10.1093/gigascience/giac042 (PMC9197682; doi:10.1093/gigascience/giac042)
Supplement: giac042_GIGA-D-21-00106_Revision_2 [file giac042_giga-d-21-00106_revision_2.pdf]

GigaScience

A chromosome-level genome assembly of Artocarpus nanchuanensis (Moraceae), an extremely endangered fruit tree

--Manuscript Draft--

**Manuscript Number:**	GIGA-D-21-00106R2
**Full Title:**	A chromosome-level genome assembly of Artocarpus nanchuanensis (Moraceae), an extremely endangered fruit tree
**Article Type:**	Research
**Funding Information:**	
**Abstract:**	Abstract<br>Artocarpus nanchuanensis (Moraceae), which is naturally distributed in China, is a representative and extremely endangered tree species. In this study, we obtained a high-quality chromosome-scale genome assembly and annotation information for A. nanchuanensis using integrated approaches, including<br>Illumina, Nanopore sequencing platform and Hi-C. A total of 128.71 gigabases (Gb) of raw Nanopore reads were generated from 20 kb libraries, and 123.38 Gb of clean reads were obtained after filtration with 160.34x coverage<br>depth and a 17.48 kb average read length. The final assembled A. nanchuanensis genome was 769.44 Mb with a 2.09 Mb contig N50, and 99.62% (766.50 Mb) of the assembled data was assigned to 28 pseudochromosomes.<br>39,596 genes (95.10%, 39,596/41636) were successfully annotated, and 129 metabolic pathways were detected. Plants disease resistance/insect resistance genes, plant-pathogen interaction metabolic pathways, and abundant biosynthesis pathways of vitamins, flavonoid and gingerol were detected. Unigene reveals the basis of species-specific functions, and gene family in contraction and expansion generally implies strong functional differences in the evolution. Compared with other related species, a total of 512 unigenes, 309 gene families in contraction and 559 gene families in expansion were detected in A. nanchuanensis .<br>This A. nanchuanensis genome information provides an important resource to expand our understanding of the unique biological processes, nutritional and medicinal benefits, and evolutionary relationship of this species. The study of gene function and metabolic pathway in A. nanchuanensis may reveal the theoretical basis of special trait in A. nanchuanensis , and promote the study and utilization of its rare medicinal value.<br>Key words: A. nanchuanensis , sequencing, Illumina, Nanopore, Hi-C, genome assembly, gene annotation, gene family.
**Corresponding Author:**	Xianping Ding<br>Sichuan University<br>Chengdu , Sichuan, CHINA
**Corresponding Author Secondary Information:**	
**Corresponding Author's Institution:**	Sichuan University
**Corresponding Author's Secondary Institution:**	
**First Author:**	Jiaoyu He
**First Author Secondary Information:**	
**Order of Authors:**	Jiaoyu He
	Shanfei Bao
	Junhang Deng
	Qiufu Li
	Shiyu Ma
	Yanru Cui
	Yiran Liu

Powered by Editorial Manager® and ProduXion Manager® from Aries Systems Corporation

	Yuqi Zhu
	Xia Wei
	Xianping Ding
	Kehui Ke
	Chaojie Chen
**Order of Authors Secondary Information:**	
**Response to Reviewers:**	We would like to thank the reviewers very much for taking the time and effort to review our paper so thoroughly. We have carefully considered their comments, which have helped us to make improvements to our work. Our point by point responses to their comments are presented below. The reviewers' comments are italicized and our responses are in red regular font. The changes we made in the text are also highlighted in red.<br><br>Reviewer reports:<br>Reviewer #1:<br>1. He et al. contributed their revised manuscript of "A chromosome-level genome assembly of Artocarpus nanchuanensis". All my comments and suggestions have been carefully treated. And, it is now ready to be accepted for publication.<br>Response: Thank you very much.<br><br>2. I have one more suggestion on improvement of title. A title with more info may be better. Do you think this my suggested titile would be better? "A chromosome-level genome assembly of Artocarpus nanchuanensis (Moraceae), an extremely endangered fruit tree"<br>Response: Thanks for your enlightening suggestion, the tittle has been revised as "A chromosome-level genome assembly of Artocarpus nanchuanensis (Moraceae), an extremely endangered fruit tree".<br><br>Reviewer #2:<br>I thank the authors for having taken into account all my remarks and suggestions. The quality of the writing has been greatly improved but there are still some mistakes, especially in the figures and tables.<br>Response: Thank you very much.<br><br>In addition, I still have a few questions.<br>1. For the RNA extraction, did the authors use a commercial kit? Please add the name of the kit and the manufacturer.<br>Response: Thank you very much. Yes, we used a commercial kit for RNA extraction, the name of the kit and the manufacturer has been added in the manuscript. (Please see line 3 of page 4)<br><br>2. Some quality filtering have been performed on Illumina reads. Please indicate the softwares used (version, parameters...).<br>Response: Thank you very much. Based on the valuable suggestions from reviewer, the softwares used (version, parameters...) for quality filtering of Illumina reads have been added in the manuscript. (Please see line 25-31 of page 4)<br><br>3. In the sentence "the next-generation sequencing data were used to conduct three rounds of calibration by Racon v1.4.21 (default parameters) and Pilon v1.22", I think that "calibration" should be replaced by "correction".<br>Response: Thank you very much. "calibration" in "the next-generation sequencing data were used to conduct three rounds of calibration by Racon v1.4.21 (default parameters) and Pilon v1.22" has been replaced by "correction". (Please see line 8 of page 5)<br><br>4. In the paragraph Genome annotation analysis, I still don't really understand the sentence" PASA v2.0.2 was used to predict unigene sequences based on the transcriptome data of a nonreference assembly". What the authors mean by "nonreference assembly".<br>Response: Thank you. "nonreference assembly" means the assembly data of nonparametric transcriptome. "PASA v2.0.2 was used to predict unigene sequences

	based on the transcriptome data of a nonreference assembly" has been revised as "PASA v2.0.2 was used to predict unigene sequences based on the assembly data of nonparametric transcriptome". (Please see line 18, 19 of page 6)<br><br>5. For the assembly quality assessment, I still suggest running Merqury (https://genomebiology.biomedcentral.com/articles/10.1186/s13059-020-02134-9) which is now commonly used. In the table 3, the authors compare several genome assembly statistics. I think it is not an assembly quality comparison but only metrics comparison. It is informative but cannot be used as a quality comparison. To compare the quality, it would be necessary to use Merqury or other tool on each assembly.<br>Response: Thank you for your valuable comments and suggestions. The Merqury has been used to conduct the assembly quality assessment of Artocarpus nanchuanensis, Ficus microcarpa and Morus notabilis, and the details of assembly quality comparison has been summarized and shown in table 3.<br>We used Merqury to evaluate our Artocarpus nanchuanensis genome as the reviewer advised and found that the value of qv and completeness are 27.6449 and 68.8838%. While, the ngs reads of our Artocarpus nanchuanensis genome were mapped onto our genome using bwa, 99.41% of reads were mapped and the reads that are properly paired are 93.56%. It indicated that if we ignore some mismatches and gaps between reads and genome, almost all of reads could be mapped onto our genome.<br>The value of completeness is relatively low comparing with 95.2558% and 79.0855% of Morus notabilis and Ficus microcarpa. We study the algorithm of Merqury and found the completeness is: It counts how many solid k-mers of reads occurring in the genome. This algorithm required that the complete same k-mers must exist in the reads and the genome at the same time. Therefore, we could notice that the completeness of Morus notabilis is the highest because it's assembled with NGS reads totally. The accuracy of read bases of NGS reads is higher than that of Pacbio/Nanopore long reads. And the completeness of Ficus microcarpa and our genome is not so high, because they were assembled with Pacbio and Nanopore long reads respectively. The ngs reads of our genome were mapped onto our genome using bwa, 99.41% of reads were mapped and the reads that are properly paired are 93.56%. It indicated that if we ignore some mismatches and gaps between reads and genome, almost all of reads could be mapped onto our genome.<br>Ficus microcarpa was reported to have 13 pseudochromosomes and our genome are 28 pseudochromosomes. It indicated that another WGS (whole genome duplication) seems to occur in A. nanchuanensis, which produces lots of similar but not identical sequences. And only one copy of some of these similar sequences was assembled into our genome because the assembler tools could seperate these highly similar sequences. Therefore, as we evaluated our genome with Merqury using ngs reads, Merqury couldn't find out the totally same sequences in our genome for k-mers in ngs reads, then Merqury reported a relative low completeness.<br><br>6. In the paragraph "Hybrid assembly, scaffolding, and chromosome anchoring", the sentence "the purification of magnetic beads during library construction functioned efficiency" should be replaced by "the purification of magnetic beads during library construction worked efficiently".<br>Response: Thank you. "the purification of magnetic beads during library construction functioned efficiency" in the "Hybrid assembly, scaffolding, and chromosome anchoring" has been revised as "the purification of magnetic beads during library construction worked efficiently". (Please see line 7 of page 9)<br><br>7. In the paragraph "Gene prediction and annotation", in the sentence "the gene number of NBS-LRR in papaya, watermelon, Arabidopsis, grape, tomato, and notabilis were 55, 44, 166, 251, and 142 respectively, while A. nanchuanensis is 1050,52", It miss one value and the value for A. nanchuanensis looks strange.<br>Response: Thank you. Based on the common domains of R genes, the number of NBS-LRR in A. nanchuanensis has been corrected as 316, and "the gene number of NBS-LRR in papaya, watermelon, Arabidopsis, grape, tomato, and notabilis were 55, 44, 166, 251, and 142 respectively, while A. nanchuanensis is 1050,52" has been revised as "the gene number of NBS-LRR in papaya, watermelon, arabidopsis, grape, tomato, and notabilis were 55, 44, 166, 504, 251, and 142 respectively, while A. nanchuanensis was 316. (Please see line 33, 34 of page 10)<br><br>8. In the title "Table 4 The Hi-C assembly datastatistics table of Artocarpus

Powered by Editorial Manager® and ProduXion Manager® from Aries Systems Corporation

	nanchuanensis.", "data" should be removed.<br>Response: Thank you very much. Sorry for my typo mistake, the title of Table 4 has been revised as "The Hi-C assembly statistics table of A. nanchuanensis". (Please see Table 4)<br><br>9. In the table 7, "alignmented" should be replaced by "aligned".<br>Response: Thank you very much. "alignmented" in the table 7 has be revised as "aligned". (Please see Table 7)<br><br>10. In the Fig 2, "The picture of the A. nanchuanensis tree study used in this study" should be replaced by "The picture of the A. nanchuanensis tree used in this study".<br>Response: Thank you very much. "The picture of the A. nanchuanensis tree study used in this study" in the Fig 2 has been revised as "The picture of the A. nanchuanensis tree used in this study". (Please see Fig. 2)<br><br>11. In the Fig 2, the two circos plots are very small and it is difficult to read the chromosome names.<br>Response: Thank you very much. The circle plots have been replaced with a higher quality figure based on your valuable suggestions. (Please see Fig. 2)<br><br>12. I suggest to move figures 3 and 4 to supp data.<br>Response: Thank you for your valuable comments and suggestions, the figures 3 and 4 have been moved to supp data. (Please see Supplementary Fig. 1 and Fig. 2)<br><br>13. In the figure 5a, the legends are very small too. I should replace "a means" by "a shows".<br>Response: Thank you for your valuable suggestion. The legends of figure have been replaced by a bigger one to make it clearer to read and "means" in the figure has been revised as "shows". (Please see Fig. 3)<br><br>14. In the figure 7, "ananlysis" should be replaced by "analysis".<br>Response: Thank you. The typo along with other mistakes have been revised. "ananlysis" in the figure 7 has been replaced as "analysis". (Please see Fig. 5)
**Additional Information:**	
**Question**	**Response**
Are you submitting this manuscript to a special series or article collection?	No
**Experimental design and statistics**<br><br>Full details of the experimental design and statistical methods used should be given in the Methods section, as detailed in our Minimum Standards Reporting Checklist. Information essential to interpreting the data presented should be made available in the figure legends.<br><br>Have you included all the information requested in your manuscript?	Yes
**Resources**	Yes

Powered by Editorial Manager® and ProduXion Manager® from Aries Systems Corporation

A description of all resources used, including antibodies, cell lines, animals and software tools, with enough information to allow them to be uniquely identified, should be included in the Methods section. Authors are strongly encouraged to cite Research Resource Identifiers (RRIDs) for antibodies, model organisms and tools, where possible.<br><br>Have you included the information requested as detailed in our Minimum Standards Reporting Checklist?	
**Availability of data and materials**<br><br>All datasets and code on which the conclusions of the paper rely must be either included in your submission or deposited in publicly available repositories (where available and ethically appropriate), referencing such data using a unique identifier in the references and in the “Availability of Data and Materials” section of your manuscript.<br><br>Have you have met the above requirement as detailed in our Minimum Standards Reporting Checklist?	Yes

Powered by Editorial Manager® and ProduXion Manager® from Aries Systems Corporation

Manuscript
Click here to access/download;Manuscript;3-26 revised 20220325153051.doc

A chromosome-level genome assembly of *Artocarpus nanchuanensis* (Moraceae), an extremely endangered fruit tree

Jiaoyu He[1,2,3], Shanfei Bao[1,2,3], Junhang Deng[1,2,3], Qiufu Li[1,2,3], Shiyu Ma[1,2,3], Yiran Liu[1,2,3], Yanru Cui[1,2,3], Yuqi Zhu[1,2,3,4], Xia Wei[1,2,3], Xianping Ding[1,2,3]*, Kehui Ke[5], Chaojie Chen[5].

1 Key Laboratory of Bio-Resources and Eco-Environment of Ministry of Education, College of Life Sciences, Sichuan University, Chengdu 610065, Sichuan, P.R.China.

2 Chongqing Jinfo Shan Advanced Research Institute, Chongqing, P.R.China.

3 Bio-resource Research and Utilization Joint Key Laboratory of Sichuan and Chongqing, Sichuan and Chongqing, P.R.China.

4 Wood Comprehensive Factory of Chengdu, Sichuan, P.R.China.

5 Biomarker Technologies Corporation, Beijing 101300, China.

Address for Correspondence: Institute of Medical Genetics, College of Life Sciences, Sichuan University, Chengdu 610064, China.

* Corresponding author:

Institute of Medical Genetics, College of Life Sciences, Sichuan University, Chengdu 610064, China.

E-mail: brainding@scu.edu.cn

Telephone: 86-028-85413096

Fax: 86-028-85415895

Email address:

Jiaoyu He: 1061355567@qq.com; Shanfei Bao: 715714892@qq.com;

Junhang Deng: 1916358148@qq.com; Qiufu Li: lqf1192069072@126.com;

Shiyu Ma: 895686227@qq.com; Yanru Cui: 512927123@qq.com;

Yiran Liu: 532154290@qq.com; Yuqi Zhu: 408843724@qq.com;

Xia Wei: 531197860@qq.com; Xianping Ding: brainding@scu.edu.cn;

Kehui Ke: kehui.ke@outlook.com; Chaojie Chen: 352300595@qq.com.

ORCIDs:

Xianping Ding [0000-0003-1555-5028]; Jiaoyu He [000-0003-4585-1354];

Abstract

Artocarpus nanchuanensis (Moraceae), which is naturally distributed in China, is a representative and extremely endangered tree species. In this study, we obtained a high-quality chromosome-scale genome assembly and annotation information for *A. nanchuanensis* using integrated approaches, including Illumina, Nanopore sequencing platform and Hi-C. A total of 128.71 gigabases (Gb) of raw Nanopore reads were generated from 20 kb libraries, and 123.38 Gb of clean reads were obtained after filtration with 160.34x coverage depth and a 17.48 kb average read length. The final assembled *A. nanchuanensis* genome was 769.44 Mb with a 2.09 Mb contig N50, and 99.62% (766.50 Mb) of the assembled data was assigned to 28 pseudochromosomes.

39,596 genes (95.10%, 39,596/41636) were successfully annotated, and 129 metabolic pathways were detected. Plants disease resistance/insect resistance genes, plant-pathogen interaction metabolic pathways, and abundant biosynthesis pathways of vitamins, flavonoid and gingerol were detected. Unigene reveals the basis of species-specific functions, and gene family in contraction and expansion generally implies strong functional differences in the evolution. Compared with other related species, a total of 512 unigenes, 309 gene families in contraction and 559 gene families in expansion were detected in *A. nanchuanensis*.

This *A. nanchuanensis* genome information provides an important resource to expand our understanding of the unique biological processes, nutritional and medicinal benefits, and evolutionary relationship of this species. The study of gene function and metabolic pathway in *A. nanchuanensis* may reveal the theoretical basis of special trait in *A. nanchuanensis*, and promote the study and utilization of its rare medicinal value.

Key words: *A. nanchuanensis*, sequencing, Illumina, Nanopore, Hi-C, genome assembly, gene annotation, gene family.

1 Introduction

Artocarpus nanchuanensis (NCBI:txid1745975), which is mainly distributed in Chongqing Nanchuan, is part of a new generation of southern urban greening tree species; this species has high quality and excellent fast-growing characteristics, that allow it to live in acidic soil and environments with heavy atmospheric pollution due to it strong ability to resist pollution and disease [1,2]. The fruit of *A. nanchuanensis* contains a variety of polysaccharides, amino acids, trace elements and vitamins,

which have a good control effect on constipation and other intestinal diseases [2]. The fruit and bark have been used in the treatment of skin diseases in Chongqing Nanchuan for a long time. These features have attracted the attention of researchers [1], and promoted the steady progress of relevant research. As research has developed, high-quality genome data are needed for this valuable species to promote studies of the molecular mechanisms related to its nutritional and medicinal value, as well as those of individual genome structure, genome evolution and species diversity.

In the draft genome sequence of the mulberry tree *Morus notabilis* (*M. notabilis*), 78.34 Gb of high-quality data were obtained and assembled into a 330.79 Mb mulberry genome with a 390,115 bp scaffold N50 and 34,476 bp contig N50 [3]. The assembled genome of *Broussonetia papyrifera* (*B. papyrifera*) was 386.83 Mb with a 29.48 Mb scaffold N50 and 171.17 Kb contig N50 [4]. The genome data analysis of *M. notabilis* and *B. papyrifera* provides a theoretical basis for the study of fibre development, lignin and flavonoid metabolism, nitrogen metabolism, important metal tolerance functions and stress resistance evolution, but the genomic details of *A. nanchuanensis* remain unknown.

To protect this species and make full use of its rare value, we applied a combined strategy involving Illumina sequencing, Nanopore single molecule sequencing and High-throughput/resolution chromosome conformation capture (Hi-C) technologies to generate sequencing data for the chromosomal genome construction and annotation of *A. nanchuanensis* [5–8] (Fig.1). These genomic data not only provide the necessary resources for the determination of genome size, but also provide convenience for research on reproduction and species evolution based on speciation and the local environment, which is beneficial to studies on the medicinal and economically valuable traits.

2 Materials and methods

2.1 Samples and DNA, RNA extraction

The oldest *A. nanchuanensis* tree surviving in Nanchuan district was selected as the sampling source (Fig. 2). Its fruits, young leaves and roots were preserved in liquid nitrogen until DNA, RNA extraction.

For genome sequencing, DNA was extracted from 100 mg young leaves by the CTAB method [9]. The concentration and purity of the extracted DNA from the sample was detected by NanoDrop and Qubit; the integrity of the DNA was checked on pulsed field electrophoresis [10]; and the extracted high-quality DNA was

prepared for subsequent sequencing [10].

The leaves, fruits and roots in the same growth stages were uniformly mixed, and a 100 mg mixture was used for RNA extraction by the Polysaccharides&Polyphenolics-rich RNAprep Pure Plant Kit (Tiangen, Beijing). The quality and concentration of the RNA were detected by Nanodrop. High-quality mRNA was purified by mRNA capture beads, and first-strand synthesis reaction buffer, random primers, and reverse transcription reagents were added to purified mRNA for mRNA fragmentation and cDNA synthesis. The synthesized and purified cDNA was incubated with end repair reaction buffer and end repair enzyme mix for end repair and 3'-end A addition in the PCR instrument. The joint, ligase and USER enzymes were added to the reaction products for joint connection and joint opening, and magnetic beads were used for fragment selection. Finally, the selected fragments were amplified by PCR, and the products were purified for sequencing.

2.2 Library construction and high-throughput sequencing

An ONT library with a 20 Kb fragment length was constructed following the manufacturer's protocol. The large segments of the extracted DNA were filtered by the BluePippin™ System, and the large segments of DNA, ONT Template Preparation Kit (SQK-LSK109) and NEB Next FFPE DNA Repair Mix Kit were used to prepare a library. The high-quality library was sequenced on the ONT PromethION Beta platform (PromethION, RRID:SCR_017987) with a corresponding R9 flow cell and ONT sequencing reagent kit (EXP-FLP001.PRO.6).

An Illumina sequencing library was prepared for genome size estimation, genome assembly correction and evaluation. The paired-end (PE) library with a 350 bp insertion size was prepared for the Illumina platform according to the manufacturers' protocols (San Diego, 112 CA, USA) and subjected to PE (2 × 150 bp) sequencing on an Illumina NovaSeq 6000 sequencing platform (Illumina, San Diego, CA, USA; RRID:SCR_016387)). For RNA, the joint and low-quality bases were filtered out with the fastp parameters (-q 10 -u 50 -y -g -Y 10 -e 20 -l 100 -b 150 -B 150 2), the rRNA was filtered by soap (parameters: soap -a 1.fq -b 2.fq -D /share/nas2/database/sRNA_database/current/ncRNA_integer.fasta. index -o out.pe -2 out.se -m 100 -x 1000 -u unmap.fa). For DNA, the joint and low-quality bases were filtered out with the fastp parameters (-q 10 -u 50 -y -g -Y 10 -e 20 -l 100 -b 150 -B 150). The filtered clean reads were used for subsequent analysis.

Hi-C fragment libraries were constructed with 300-700 bp insertion sizes, as

illustrated in Rao et al [11], and sequenced by sequencing by synthesis (SBS) using the Illumina platform. Briefly, adapter sequences of raw reads were trimmed and low-quality PE reads were removed to generate clean data.

2.3 Genome assembly and quality assessment

Nanopore next-generation clean sequencing data were obtained by Canu v1.5 [12] software (Canu, RRID:SCR_015880). In the correction step, Canu v1.5 first selected longer seed reads with the settings 'genomeSize=780000000' and 'corOutCoverage=50'. SMARTdenovo [13](default parameters) software was used to assemble the corrected data, and then the next-generation sequencing data were used to conduct three rounds of correction by Racon v1.4.21 (RRID:SCR_017642; default parameters) [14] and Pilon [15] v1.22 (RRID:SCR_014731; parameters: --mindepth 10 --changes --threads 4 --fix bases) software. The assembly results were evaluated by the read alignment rate, core gene integrity, and BUSCO evaluation. BWA [16] software (BWA, RRID:SCR_010910) was used to align short sequences on the reference genome. The CEGMA [17] v2.5 (default parameters) database and BUSCO v4.0.6 (RRID:SCR_015008; parameters: odb10, -c 24 -e 1e-3) [18] were used to evaluate the completeness of the assembly.

2.4 Chromosomal-level genome assembly using Hi-C data

Before chromosome assembly, we first performed a preassembly for error correction of scaffolds, which required splitting scaffolds into segments of 50 kb on average. The Hi-C data were mapped to these segments using BWA (version 0.7.10-r789, default parameters) software. Only uniquely alignable read pairs whose mapping quality was greater than 20 were retained for further analysis. Invalid read pairs, including dangling-end and self-cycle, re-ligation and dumped products, were filtered by HiC-Prov2.8.1 (default parameters) [19]. The uniquely mapped data were retained to perform assembly with LACHESIS [20] software (LACHESIS, RRID:SCR_017644). Any two segments that showed inconsistent connections with information from the raw scaffold were checked manually. These corrected scaffolds were assembled by LACHESIS. Parameters for running LACHESIS included CLUSTER_MIN_RE_SITES = 5; CLUSTER_MAXLINK_D ENSITY = 2; CLUSTER_ NONINFORMATIVE_RATIO = 2; ORDER_MIN_N_R ES_IN_TRUN = 5; and ORDER_MIN_N_RES_IN_ SHREDS = 5. After this step, placement and orientation errors exhibiting obvious discrete chromatin interaction patterns were manually adjusted.

2.5 Genome annotation analysis

Due to the relatively poor conservation of interspecies repeat sequences, it is necessary to construct a unique repeat sequence database for predicting repeat sequences of specific species. LTR_FINDER [21] v1.05 (RRID:SCR_015247; default parameters) and RepeatScout [22] v1.0.5 (RRID:SCR_014653; default parameters) were used to construct the repetitive sequence database of *A. nanchuanensis* based on structure prediction and de novo sequencing theory. Then, the database was classified by PASTEClassifier v1.0 (RRID:SCR_017645; default parameters) [23] and merged with Repbase19.06 [24] (null) as the final repetitive sequence database. Finally, RepeatMasker [25] (RRID:SCR_012954; parameters: -nolow -no_is -norna -engine wublast -qq -frag 20000) software was used to predict the repetitive sequences in the *A. nanchuanensis* genome based on the constructed repetitive sequence database.

The structures of coding genes were predicted by ab initio prediction, homologous species prediction and unigene prediction using three different strategies. Genscan [26] v3.1 (GENSCAN, RRID:SCR_013362), Augustus [27] v2.4 (Augustus, RRID:SCR_008417), GlimmerHMM [28] v3.0.4 (GlimmerHMM, RRID:SCR_002654), GeneID [29] v1.4 and SNAP [30] (version 2006-07-28) were used for ab initio prediction with default parameters. GeMoMa [31, 32] v1.3.1 (RRID:SCR_017646; default parameters) was used for homologous species prediction; Hisat [33] v2.0.4 (RRID:SCR_015530; parameters --max-intronlen 20000, --min-intronlen 20) and Stringtie [34] v1.2.3 (RRID:SCR_016323; default parameters) were used for assembly based on reference transcripts. TransDecoder v2.0 (TransDecoder, RRID:SCR_017647) and GeneMarkS-T [35] v5.1 (GeneMarkS-T, RRID:SCR_017648) were used for gene prediction with default parameters. PASA [36] v2.0.2 (RRID:SCR_014656; parameters: -align_tools gmap, -maxIntronLen 20000) was used to predict unigene sequences based on the assembly data of nonparametric transcriptome. Finally, EVM [37] v1.1.1 (default parameters) was used to integrate the prediction results obtained by the above three methods, and PASA v2.0.2 (parameters: -align_tools gmap, -maxIntronLen 20000) was used to modify the prediction results.

Noncoding RNAs were predicted by different strategies based on their structural characteristics. Rfam [38] v12.1 (RRID:SCR_007891; parameters: 1e-5) was used to identify microRNAs and rRNAs, and tRNAscan-SE [39] v1.3.1 (RRID:SCR_010835; parameters: 1e-5) was used to identify tRNAs.

The predicted protein sequences were compared with GenBlastA [40] v1.0.4 (RRID:SCR_020951; parameter: e-value -e 1e-5), and immature stop codons and transcoding mutations in the gene sequences were searched to obtain pseudogenes by GeneWise [41] 2.4.1 (RRID:SCR_015054; default parameters).

The predicted gene sequences were aligned to the nonredundant protein sequences (NR) [42], eukaryotic orthologous groups of proteins (KOG) [43], Gene Ontology (GO) [44], Kyoto Encyclopedia of Genes and Genomes (KEGG) [45], TrEMBL [46] and other functional databases by BLAST [47] v2.2.31 (parameters: -evalue 1e-5), to perform KEGG pathway, KOG functional, GO functional and other gene functional annotation analyses.

2.6 Gene family and phylogenetic analysis.

The protein sequences of *A. nanchuanensis* and their related species *(Arabidopsis thaliana (A. thaliana)* [48], *Amborella trichopoda (A. trichopoda)* [49], *Populus trichocarpa (P. trichocarpa)* [50], *Actinidia chinensis (A. chinensis)* [51], *Vitis vinifera (V. vinifera)* [52], *Morus notabilis Schneid (M. notabilis)* [3], *and Theobroma cacao (T. cacao)* [53]) were aligned to analyse gene replication within the species, the evolution between species and the classification of species-specific genes. OrthoMCL [54] v2.0.9 (parameters: PercentMatchCutoff 50, EvalueExponentCutoff -5) software was used to classify the protein sequences of *A. nanchuanensis*, *A. thaliana*, *A. trichopoda*, *P. trichocarpa*, *A. chinensis*, *V. vinifera*, *M. notabilis*, and *T. cacao* to determine unique gene families in *A. nanchuanensis*.

PHYML [55] (RRID:SCR_014629; version: 20151210, parameters: -gapRatio 0.5 -badRatio 0.25 -model HKY85 -bootstrap 1000) was used to construct the evolutionary tree based on the single-copy protein sequences of *A. nanchuanensis* and 7 other species to study the evolutionary relationships among species. TimeTree (TimeTree, RRID:SCR_021162) [56] was used to select the known taxa for time calibration, and Mcmctree (parameter: default) was used to estimate the time of interspecies differentiation. CAFE 4.2 [57] (RRID:SCR_005983; parameter: lambda -l 0.002) was used to conduct gene family contraction and expansion analysis. The Branch model of the CodeML [58] module in PAML 4.7a (parameters: noisy = 3, verbose = 1, runmode = 0, seqtype = 1, CodonFreq = 2, clock = 0, aaDist = 0, model = 2, NSsites = 2, icode = 0, Mgene = 0, fix_kappa = 0, kappa = .3, fix_omega = 0, omega = 1, ncatG = 2, getSE = 0, RateAncestor = 0, Small_Diff = .45e-6, cleandata = 1, and fix_blength = 0) was used to analyses the selection pressure of single-copy

genes and conduct the functional annotation and enrichment analysis.

LTR_FINDER v1.07 (RRID:SCR_015247; parameter: default) and PS SCAN [59] (version: 3.8.31, parameter: default) software were applied to search for LTR sequences in the genome with scores greater than or equal to 6 points, and the repeated results were filtered with LTR_FINDER. The LTR flanking sequences were compared with MUSCLE [60] (version: 3.8.31, parameter: default), and the distance was calculated by DistMat software using a Kimura model with a 7.3*10-9 molecular clock.

3 Results and discussion

3.1 Initial characterization of the *A. nanchuanensis* genome

A total of 51.76 Gb of high-quality *A. nanchuanensis* data were obtained from the Illumina sequencing platform with an approximately 68× sequencing depth, and the genome size was calculated to be 761.07Mb. Based on 4 ^ K/genome>200, a kmer distribution map of K = 17 was constructed (Supplementary Fig. 1). The amount of repeated sequences content was estimated to be approximately 55.80%, and the heterozygosity was estimated to be approximately 0.93%, indicating that the *A. nanchuanensis* genome was highly heterozygotic and complex. Details are shown in Table 1.

A total of 128.71 gigabases (Gb) of reads were generated by the Nanopore platform, and 123.38 Gb of clean data were obtained after quality control. The average read length reached 17.48 kb, the N50 read length was 19.18 kb, and the total sequencing depth was approximately 160.34 ×. Clean data obtained by filtering out the low-quality data reached 7,057,335 reads. Details are shown in Table 1.

The total sequencing depth of the Illumina and Nanopore platforms was 228.35×.

3.2 Genome assembly and completeness evaluation

After sequencing by Nanopore three-generation sequencing, correction by Canu, assembly by SMARTdenovo and polishing by Racon, Pilon software, a total of 769.44 Mb of *A. nanchuanensis* genome sequences was generated with 1087 contigs, a 2.09 Mb contig N50 and a 402 kb contig N90 (Table 2). The contig N50/ N90 and scaffold N50/ N90 of *M. notabilis* were 34,476 bp/2,231 bp and 390,115 bp/11,563 bp; contig N50/ N90 and scaffold N50/ N90 of *B. papyrifera* were 171.17 kb/ 38.90 kb and 29.48 Mb/17.97 Mb [4]; contig N50/ N90 of *F. microcarpa* were 907,868 bp/113,961bp (Table 3) [61]. Compared with other reported moraceae plants, the genome size of *A. nanchuanensis* is bigger. (Table 3).

Statistical alignment analysis of second-generation sequencing reads showed that

clean reads located on the reference genome accounted for 99.41% of the total clean reads (363,371,475/365,545,724). The paired-end sequences of the correct size that were located on the reference genome, accounted for 93.56% of the total clean reads (341,995,184/365,545,724). The core gene integrity assessment was performed by CEGMA v2.59. Here, 445 CEGs were present in assembly, accounting for 97.16% of all CEGs (445/458), while 232 highly conserved CEGs were present in the assembly, accounting for 93.55% of all CEGs (232/248). The database in BUSCO v4.0.6 contains 1,614 conserved core genes, and the number of complete genes present in the assembly is 1583 (98.08%); details are shown in Supplementary Fig. 2.

Table 1 The sequence statistics of *Artocarpus nanchuanensis.*

Illumina		Nanopore		Hi-c	
Data*	51.76 Gb	Data*	123.38Gb	Data*	137.5 Gb
Depth/genome coverage	68.01 X	Depth/genome coverage	160.34 X	Depth	62 X
Total Kmer	45,202,482,693	MaxLen	216,661 bp	Total Read Pairs	458,907,479
Genome	761.07 Mb	SeqNum	7,057,335	Genome	769.44 Mb
Heterozygosity	0.93%	N50Len	19,177 bp	Contig N50	1.78 Mb
Repeated	55.80%	N90Len	11,029 bp	Scaffold N50	25.15 Mb
Mapping rate	99.41%				

Note: Data* mean the data has been filter to be clean data; Depth/genome coverage means depth of sequencing data; MaxLen means the longest reads length of sequencing data; SeqNum means the total read number of sequencing data; N50Len means the N50 length of sequencing data reads; N90Len means the N90 length of sequencing data reads.

Table 2 Nanopore and Hi-C genome assembly statistics of *Artocarpus nanchuanensis*.

Nanopore assembly results		Hi-C Assembly results	
Contig number	1,087	Scaffold / Contig number	809 / 1,364
Contig length	769,440,982 bp	Scaffold / Contig length (bp)	769,496,482/769,440,982
Contig N50	2,094,024 bp	Scaffold / Contig N50 (bp)	25,150,906/1,778,064
Contig N90	402,757 bp	Scaffold /Contig N90 (bp)	20,179,149 / 200,000
Contig max	8,879,419 bp	Scaffold / Contig max (bp)	32,505,427/ 8,646,128
		Gap total length (bp)	55,500
		GC content (%)	32.34

Note: Contig represents the contig after error correction. Scaffold represents the scaffold generated after connection, and scaffold length exceeds 1 Kb. Scaffold/Contig number represents the number of scaffold and contig in the scaffold; Scaffold/Contig length represents the length of scaffold and contig in the scaffold; Scaffold/Contig N50 represents length of scaffold N50 and contig N50;

Scaffold/Contig N90 represents length of scaffold N90 and contig N90; Scaffold / Contig max represents the length of the longest scaffold and longest contig; GC content represents the GC content percentage.

Table 3 The genomes assemblies quality comparison of *A.nanchuanensis* and its related Moraceae plants.

Latin name (genus)	*Morus notabilis* (Morus Linn)	*Ficus microcarpa* (Ficus)	*A.nanchuanensis* (Artocarpus)
Sequencing technology	Illumina HiSeq 2000	Illumina, PacBio RS II, Hi-C	Illumina, Nanopore, Hi-C
Sequencing depth	236.82 X (78.34 Gb, Illumina)	86.55 X (36.87 Gb, Pacbio)	160.34 X (123.83 Gb, Nanopore)
Contig/Scaffold N50	34,476 bp/390,115 bp	907,868 bp / None	2.09 Mb/25.15 Mb
Contig/Scaffold N90	2,231 bp/11,563 bp	113,961 bp / None	402.76 Kb/20.18 Mb
Annotated genes	29,338	29,416	41,636
Repeat composition	127.98Mb	198.23Mb	422.78Mb
Unique kmer in genome	2198905	5732202	26038922
Kmer in genome and reads	303168905	425981208	769420329
QV	34.6019	31.9049	27.6449
Error rate	0.000346583	0.000644926	0.00171993
solid kmer in genome	210228161	250755719	520815448
total solid kmer in reads	220698486	317069158	756078105
Complete (%)	95.2558	79.0855	68.8838

3.3 Hybrid assembly, scaffolding, and chromosome anchoring

We obtained 137.5 Gb clean Hi-C data (approximately 62 × depth of the estimated genome). The clean Hi-C reads accounted for 179-fold coverage of the 769.44 Mb genome estimated by the Illumina platform for subsequent analysis (Table 1). To assess the quality of Hi-C data, we performed an insertion fragment length assessment, which showed a relatively narrow unimodal length distribution with the highest peak at approximately 300 bp, indicating that the dispersion degree of the inserted fragment length was small, the inserted fragment size was normal and the purification of magnetic beads during library construction worked efficiently (Fig. 3). A total of 728,487,984 paired reads were genome-related mapping reads, accounting for 79.37% of the clean data. A total of 236,274,160 paired reads were uniquely

mapped on the genome assembly, including 56,964,635 valid Hi-C paired reads. Details are shown in Supplementary Table 1, 2. Alignment efficiency, insert fragment length and effective Hi-C data volume evaluation all indicated that the Hi-C libraries were constructed well.

After Hi-C assembly and manual adjustment, a total of 766.50 Mb of genomic sequences were located on 28 chromosomes through scaffold correction, clustered, ordered and orientated, accounting for 99.62% of all genomic sequences, and the corresponding number of sequences was 1,336 (97.95%). Among the sequences located on the chromosome, the sequence length based on order and direction was 697.71 Mb, accounting for 91.02% of the total length of the sequences on the chromosomes (Table 4). The contig N50 and Scaffold N50 were 1.78 Mb and 25.15 Mb, respectively, after error correction (Table 1). The final pseudochromosomes were constructed after manual adjustment.

The genomes of *A. nanchuanensis* and *Ficus.microcarpa* (*F. microcarpa*) were compared to verify the accuracy of the overlap across the 28 chromosomes, and the collinearity circle diagram indicates a high similarity of genes order between them (Fig. 2). A heatmap was drawn to evaluate the structure and quality of Hi-C assembly (Fig. 3). The figure indicated that the 28 pseudochromosomes could be distinguished easily and the interaction signal intensity at the diagonal was significantly stronger than that at other locations within each pseudochromosome.

3.4 Gene prediction and annotation

A total of 422.78 Mb (54.94%) of repeat sequences was detected; among these repeat elements, long terminal repeats (LTRs) were the predominant type, whereas Class I/LTR/Copia and Class I/LTR/Gypsy accounted for 19.17% (147.52 Mb) and 16.86% (129.74 Mb). The details of the repeat sequences are shown in Supplementary Table 3.

A total of 41,636 protein-coding genes were predicted with a 3,797.54 bp average gene length, a 1,509.16 bp average exon length, and a 2,288.38 bp average intron length by ab initio-based, homologue-based, and RNA-seq-based methods; 27,262 genes were both obtained by the above three prediction methods (Table 5, Supplementary Fig. 3 and Supplementary Table 4). Based on GenBlastA v1.0.4 and GeneWise2.4.1, 1,905 pseudogenes were obtained, and their total length and average length were 4,825,668 kb and 2,533.16 kb, respectively (Supplementary Table 5).

A total of 39,596 genes were successfully annotated in the functional databases, accounting for 95.10% (39,596/41636) of the predicted genes; details are shown in

Supplementary Table 7. According to the noncoding RNA prediction results, the number of miRNAs was 138, belonging to 24 RNA families; there were 409 rRNAs, belonging to 4 RNA families; and there were 512 tRNAs, belonging to 24 families (Supplementary Table 6).

Table 4 The Hi-C assembly statistics table of *A. nanchuanensis*.

Group	Cluster number	Cluster length (bp)	Order number	Order length (bp)
LG01	46	26,514,107	24	24,676,255
LG02	41	26,638,661	16	24,489,134
LG03	30	24,254,703	16	23,044,270
LG04	34	22,404,888	13	20,644,200
LG05	33	21,646,681	16	20,177,649
LG06	35	29,133,579	18	27,822,153
LG07	69	32,924,820	27	29,467,719
LG08	45	29,858,101	20	27,605,363
LG09	77	29,556,483	29	25,185,028
LG10	45	22,896,788	20	20,243,522
LG11	67	25,833,105	20	21,750,724
LG12	37	24,385,337	15	22,370,729
LG13	47	23,481,896	24	21,098,278
LG14	46	29,162,015	19	26,857,340
LG15	61	28,431,484	30	25,341,045
LG16	32	21,965,556	16	20,879,538
LG17	41	25,915,114	19	24,032,910
LG18	49	34,941,454	27	32,502,827
LG19	54	29,520,137	21	25,685,935
LG20	50	32,513,478	18	29,815,261
LG21	50	28,639,915	21	25,613,043
LG22	42	27,392,871	24	25,873,084
LG23	42	28,655,389	16	26,447,344
LG24	52	27,753,222	24	25,148,606
LG25	46	23,720,417	16	21,152,151
LG26	63	33,995,937	28	30,220,329
LG27	58	28,458,315	24	25,577,647
LG28	44	25,907,258	22	23,985,053
Total (Ratio%)	1336 (97.95%)	766501711 (99.62%)	583 (43.64%)	697707137 (91.02%)

Note: the statistics do not include 100 Ns added by artificially connected pseudochromosomes.

Table 5 The prediction analysis of *A. nanchuanensis* coding gene.

Prediction style and proportion			
Gene Number	41,636	CDS length	50,445,441 bp
Gene length	158,114,419 bp	CDS average length	1,211.58 bp
Gene average length	3,797.54 bp	CDS number	226,727
Exon length	62,835,343 bp	CDS average number	5.45
Exon average length	1,509.16 bp	Intron length	95,279,076 bp
Exon number	233,559	Intron average length	2,288.38 bp
Exon average number	5.61	Intron number	191,923
		Intron average number	4.61

The number of homologous genes between *A. nanchuanensis* and *M. notabilis* was 30,510, accounting for 77.14%, based on the Nr homologous species distribution, indicating high homology (Fig. 4). The KOG database is based on the phylogenetic relationships of protein-coding genes in bacteria, algae, and eukaryotes with complete genomes and classifies the gene products based on linear homology and at the functional level. A total of 21,567 (51.80%) *A. nanchuanensis* genes were annotated in the KOG database (Supplementary Table 7), and the annotation classification details are shown in Supplementary Fig. 4. The top three over expressed genes were mainly involved in posttranslational modification, protein turnover, chaperones, signal transduction mechanisms and transcription. The GO database was used to define and describe the genes and proteins, according to their involvement in biological processes, the components that make up cells, and the molecular functions they perform (Supplementary Fig. 5). Annotated gene number and repeat sequence size of *A. nanchuanensis* were 41,636 and 422.78Mb, that are bigger than that of in previously reported *M. notabilis* (29,338, 127.98 Mb), *F. microcarpa* (29,416, 198.23 Mb) and *B. papyrifera* (30,512, 190.23 Mb) [3,4,61], indicating the high quality of sequencing and annotation for *A. nanchuanensis* (Table 3).

Nucleotide-binding site and leucine-rich repeat (NBS-LRR) has been well known as major plants disease resistance gene, the gene number of NBS-LRR in papaya, watermelon, arabidopsis, grape, tomato, and notabilis were 55, 44, 166, 504, 251, and 142 respectively, while *A. nanchuanensis* was 316 [3,51]. As particular *NBS-LRR* genes recognize specific pathogen effectors, the number of *NBS-LRR* genes may represent good potential for pathogen recognition, that is consistent with the strong

resistance to disease of *A. nanchuanensis*. For minimize the dangers of insect infestation, plants evolved a defence mechanism by expressing plant protease inhibitors (PIs) to interfere digestive systems of insects, and 8 Glu S.griseus protease inhibitor genes were detected in *A. nanchuanensis* [50]. *PIs* and *NBS-LRR* genes are reasonably important for defense response in *A. nanchuanensis* ancient species.

KEGG is the main public database of pathway, and 129 metabolic pathways of *A. nanchuanensis* were finally obtained. Plant-pathogen interaction metabolic pathways may closely relate to the resistance of disease and insect pests. Abundant biosynthesis pathways of Vitamins, flavonoid and gingerol may reveal the theoretical basis of *A. nanchuanensis* rare medicinal value.

3.5 Comparative genomics

The protein sequences between *A. nanchuanensis* and its related species (*A. thaliana, A. trichopoda*, *P. trichocarpa*, *A. chinensis, V. vinifera*, *M. notabilis,* and *T. cacao*) were compared, and 33,925 genes out of 41,636 *A. nanchuanensis* predicted genes were clustered into 15,436 gene families, of which 512 were unique to *A. nanchuanensis* (Table 6 and Supplementary Fig. 6). In the phylogenetic tree of *A. nanchuanensis* and its related species, *A. nanchuanensis* diverged from *M. notabilis* approximately 0.5285 million years ago (Mya) by Mcmctree estimation, diverged from *A. chinensis* and *V.vinifera* approximately 19.3794 Mya and from *A. thaliana, T. cacao, and P. trichocarpa* approximately 18.6558 Mya, which support the close relationship between A. *nanchuanensis and M. notabilis* (Fig. 5). This result was confirmed by the analysis of homologous species distribution, transversions at fourfold degenerate sites (4DTv) and chromosome genes order.

In the evolutionary process, gene families in contraction and expansion generally implies strong functional changes. According to the evolutionary relationships among species and the results of gene family clustering, 309 gene families in contraction and 559 gene families in expansion were detected in *A. nanchuanensis* after divergence from mulberry (Fig. 5). These gene families in contraction are mainly related to F-box domain, cystatin domain, protein kinase domain and ring finger domain functions (Table 7). Refers to the common ancestor, except for *A. thaliana* and *P. trichocarpa*, the number of gene families in contraction is bigger than that of in expansion among other species, suggesting that more gene families in most species experienced contraction than expansion during adaptive evolution, and the living environment of *A. thaliana* and *P. trichocarpa* may be challengeable, that expand their gene family to cope with the living environment.

Table 6 Statistical classification of gene families.

Name	Total gene	Cluster	Total family	Unifamily
A. thaliana	27,369	23,106	12,753	726
A. trichopoda	16,986	15,058	11,147	254
P. trichocarpa	41,335	33,270	14,725	950
A. chinensis	39,040	25,888	12,648	1,327
V. vinifera	26,346	19,238	12,682	665
M. notabilis	26,965	20,423	14,794	524
T. cacao	21,432	20,070	13,810	176
A. nanchuanensis	41,636	33,925	15,436	512

Note:Total gene: the number of total gene; Cluster: the number of genes that involved in family classification; Total family number: the number of gene families that can be divided; Uni family: the number of unique gene families.

Table 7 The annotation of protein gene family.

GeneFamily	Pfam	Function
GF_12673	PF00646.28	F-box domain
GF_10548	PF00031.16	Cystatin domain
GF_8	PF00069.20	Protein kinase domain
GF_13176	PF13639.1	Ring finger domain

Note: Gene family : the gene family cluster; Pfam: the ID of protein family alignment to the Pfam database；Function：the function of the protein family that can be aligned.

EVM0035972.1, EVM0031735.1, EVM0026117.1 and EVM0015119.1 were found to be rapidly evolving genes, and details on these genes and their annotated functions are shown in Table 8 and Supplementary Fig. 7. 4DTv are neutral genetic distances that can be used to estimate the relative timing of evolutionary events [62]. According to the homologous gene pairs between two species or within species themselves, the ratio of each homologous gene to the 4DTV mutation site was calculated, and a 4DTV distribution map was made (Fig. 6). The peak of the 4DTv distribution among *A. nanchuanensis* and *M. notabilis* was closer to the current than that of *A. nanchuanensis* and other species, indicating that the differentiation time of *A. nanchuanensis* and *M. notabilis* appeared recently, suggesting a closer genetic relationship between them. At ancient time, the 4DTV distribution curves of *A.*

nanchuanensis and other species were similar, which reflected these species might share similar whole-genome duplication (WGD) events. Moreover, the 4DTV distribution of *A. nanchuanensis* had a small peak at 0.05, which suggested that some genomic fragments duplicated recently.

Table 8 The rapidly evolving genes selected by CodeML.

GeneID	P-value	Sites
EVM0035972.1	0.05	298,G,0.993**
EVM0031735.1	0.06	74,E,0.984*
EVM0026117.1	0.35	68,K,0.997**
EVM0015119.1	0.00	232,E,0.990**

Note: Gene ID mean the ID of gene, ω0 mean ka/ks for the studied Species, ω1 mean the average ka/ks for other species, ω2 mean ka/ks for the whole evolutionary tree.

LTR accumulation is able to reflect that the species may cope with some environmental stresses on its survival [21]. LTR insertion time among *A. nanchuanensis* and other 7 related species show that the living environment of *A. nanchuanensis* is relatively stable. The narrow peak of LTR insertion time around 1 Mya indicated some environment stress or environment change has been imposed on *A. nanchuanensis* and its living environment (Fig. 6).

4. Conclusion

In this study, a high-quality genome assembly and annotation information for *A. nanchuanensis* were first reported, resulting in the first reference genome for the Artocarpus genus. A total of 123.38 Gb of clean reads were obtained and a 769.44 Mb genome was assembled, which was larger than that of the sequenced *M. notabilis* and *B. papyrifera*. The clean reads mapped percentage (99.41%), CEGs and highly conserved CEG present in assemblies (97.16%, 93.55%), and BUSCO conserved gene core set coverage (98.08%) indicated that the current assembly covers most of the *A. nanchuanensis* genome; The *A. nanchuanensis* genome size estimated by k-mer analysis was 761.07 Mb, and the assembly was 769.44 Mb; These data suggested that this assembly was mostly representative of the complete *A. nanchuanensis* genome and indicated the high quality of *A. nanchuanensis* genome assembly. *A. nanchuanensis* and *M. notabilis* are both composed of 7 chromosome pairs, and their high similarity in genes order indicated high continuity between *A. nanchuanensis* and *M. notabilis,* as well as the high quality of the *A. nanchuanensis* genome assembly.

NBS-LRR, *PIs* plants disease and insect resistance genes were detected in the

gene prediction and annotation analysis of *A. nanchuanensis,* represent good potential for pathogen recognition, that is consistent with the inherent strong resistance to pests and diseases of *A. nanchuanensis*. Several anti-inflammatory metabolism and anti-inflammatory substance synthesis pathways were detected, which may be related to the unique antiallergic function of *A. nanchuanensis*. Study of relevant functions and metabolic pathways reveal fruit maturation, nutrient metabolism, disease resistance of *A. nanchuanensis*.

Gene families in contraction and expansion generally implies strong functional changes, unigene indicate special species function, the in-depth study of above gene provides the research foundation for *A. nanchuanensis* unique features. Meanwhile, the LTR insertment analysis may indicate the stability of ecological environment that *A. nanchuanensis* living in. Species genome analysis not only reveal their functions and evolutionary relationships, but also reflect their growth environment.

This high-quality genome of *A. nanchuanensis* will lay a solid foundation for the conservation, rational development, and utilization of critically endangered species in the future. It is a valuable resource for the genetic improvement and better understanding of *A. nanchuanensis* genomic evolution. This genome will also be invaluable in developing new varieties and addressing issues of agronomic and/or biological importance such as fruit development and maturation, nutrient metabolism of fruits, and disease resistance of *A. nanchuanensis* and related plant spices.

Data Availability

The whole raw sequence reads produced by Illumina novaseq, Pacbio sequel II and ONT PromethlON Beta, have been deposited at NCBI Sequence Read Archive (SRA) under BioProject number PRJNA624965 and BioSample from SAMN14589993, SAMN26429610 for *A. nanchuanensis*. Raw sequencing data (Nanopore, Illumina, Hi-C, RNA-seq data) have been deposited in SRA database as SRR11671532, SRR11659666, SRR11659674, SRR11623450/SRR11668249. All supporting data and materials are available in the *GigaScience* GigaDB database [63].

List of abbreviations

4DTv, Transversions at fourfold degenerate sites; Gb, Gigabases; GO, Gene Ontology; Hi-C, High-throughput/resolution chromosome conformation capture; KEGG, Kyoto Encyclopedia of Genes and Genomes; KOG, Eukaryotic orthologous

groups of proteins; LTRs, Long terminal repeats; Mya, Million years ago; NBS-LRR, Nucleotide-binding site and leucine-rich repeat; NR, Nonredundant protein sequences; PE, Paired-end; PIs, Plant protease inhibitors; SBS, Sequencing by synthesis; SRA, Sequence Read Archive; WGD, Whole-genome duplication.

Author contributions

J.H., S.B., X.D., K.K. and C.C. conceived and designed the study; J.H., S.B., X.D., J.D. X.W. and Q.L. collected the samples; Q.L., Y.Z. and Y.L. performed DNA sequencing and Hi-C experiments; Y.C. and L.F. performed RNA sequencing; J.H., Q.L. and Y.Z. estimated the genome size, assembled the genome, and assessed the assembly quality; Y.C. and L.F. performed the genome annotation and functional genomic analysis. S.X., J.H. and X.D. wrote the manuscript. All authors read, edited, and approved the final manuscript for submission.

Competing interests

The authors declare no competing interests.

Funding

This work was supported by the funding of Chinese Ministry of Education, Chongqing Nanchuan biotechnology research institute, Sichuan and Chongqing government.

Acknowledgements

This work was supported by Key Laboratory of Bio-Resources and Eco-Environment of Ministry of Education, College of Life Sciences, Sichuan University, Chengdu 610065, Sichuan, P.R.China. and Chongqing Nanchuan biotechnology research institute, Bio-resource Research and Utilization Joint Key Laboratory of Sichuan and Chongqing, Sichuan and Chongqing, P.R.China.

References

1. Rong-, L. I. U. Studies on Chemical Constituents Occurring in Twigs of Artocarpus nanchuanensis. Chinese Journal of Experimental Traditional Medical Formulae. 2–6 (2013). doi:10.11653 /syfj2013220092
2. Ren, G. et al. Chemical constituents from the fruiting branches of Artocarpus nanchuanensis endemic to China. Biochem. Syst. Ecol. 51, 98–100 (2013). doi:10.1016/j.bse.2013.08.019
3. He, N. et al. Draft genome sequence of the mulberry tree Morus notabilis. nature communcation. (2013). doi:10.1038/ncomms3445
4. Peng, X. et al. A Chromosome-Scale Genome Assembly of Paper Mulberry (Broussonetia papyrifera) Provides New Insights into Its Forage and

Papermaking Usage. Mol. Plant 12, 661–677 (2019). doi.org/10.1016/j.molp.2019.01.021.

5. Sevim, V. et al. Shotgun metagenome data of a defined mock community using Oxford Nanopore, PacBio and Illumina technologies. Sci. Data 6, 1–9 (2019). doi:10.1038/s41597-019-0287-z
6. Branton, D. et al. The potential and challenges of nanopore sequencing. Nat. Biotechnol. 26, 1146–1153 (2008). doi:10.1038/nbt.1495
7. Belton, J. M. et al. Hi-C: A comprehensive technique to capture the conformation of genomes. Methods. 58, 268–276 (2012). doi:10.1016/j.ymeth.2012.05.001
8. van Berkum, N. L. et al. Hi-C: A method to study the three-dimensional architecture of genomes. J. Vis. Exp. 1–7 (2010). doi:10.3791/1869
9. Gawel, N. J. & Jarret, R. L. A Modified CTAB DNA Extraction Procedure for Musa and Ipomoea. Plant Mol. Biol. 9, 262–266 (1991). doi:10.1007/BF02672076
10. Bian, L. et al. Chromosome- level genome assembly of the greenfin horse- faced filefish (Thamnaconus septentrionalis) using Oxford Nanopore PromethION sequencing and Hi- C technology. Mol. Ecol. Resour. 1–25 (2020). doi:10.1111/1755-0998.13183
11. Rao, S. S. P., Huntley, M. H., Durand, N. C. & Stamenova, E. K. Article A 3D Map of the Human Genome at Kilobase Resolution Reveals Principles of Chromatin Looping. Cell. 1–16 (2014). doi:10.1016/j.cell.2014.11.021
12. Koren, S. et al. Canu: scalable and accurate long-read assembly via adaptive k-mer weighting and repeat separation. Genome Res. 27, 722–36 (2017). doi:10.1101/gr.215087.116
13. Hailin Liu, Shigang Wu, Alun Li, Jue Ruan, SMARTdenovo: a de novo assembler using long noisy reads, Gigabyte, 2021. doi:10.46471/gigabyte.15
14. Vaser, R., Sovi, I., Nagarajan, N. & Šiki, M. Fast and accurate de novo genome assembly from long uncorrected reads. Genome Research (2017) doi:10.1101/gr.214270.1162017.
15. Walker, B. J. *et al.* Pilon : An Integrated Tool for Comprehensive Microbial Variant Detection and Genome Assembly Improvement.Plos one. **9**, (2014). doi:10.1371/journal.pone.0112963
16. Li, H. & Durbin, R. Fast and accurate short read alignment with Burrows – Wheeler transform. Bioinformatics. **25**, 1754–1760 (2009). doi:10.1093/bioinformatics/btp324
17. Parra, G., Bradnam, K. & Korf, I. Genome analysis CEGMA : a pipeline to accurately annotate core genes in eukaryotic genomes. Bioinformatics. **23**, 1061–1067 (2007). doi:10.1093/bioinformatics/btm071
18. Simão, F. A., Waterhouse, R. M., Ioannidis, P. & Kriventseva, E. V. BUSCO: assessing genome assembly and annotation complete- ness with single-copy orthologs. Bioinformatics. 9–10 (2015). doi:10.1093/bioinformatics/btv351
19. Servant, N. et al. HiC-Pro: an optimized and flexible pipeline for Hi-C data processing. Genome biology. 1–11 (2015). doi:10.1186/s13059-015-0831-x
20. Burton, J. N. et al. Chromosome-scale scaffolding of de novo genome assemblies based on chromatin interactions. Nature biotechnology. (2013). doi:10.1038/nbt.2727
21. Xu, Z. & Wang, H. LTR_FINDER: an efficient tool for the prediction of full-length LTR retrotransposons. Nucleic acids research. 35, 265–268 (2007). doi:10.1093/nar/gkm286
22. Price, A. L., Jones, N. C. & Pevzner, P. A. De novo identification of repeat families in large genomes. Bioinformatics. 21, 351–358 (2005). doi:10.1093/bioinformatics/bti1018
23. Abel, L. W. Planning a dynamic kill. JPT, J. Pet. Technol. 48, 422–426 (1996). doi:10.2118/36071-JPT
24. Jurka, J. et al. Diversity of Retrotransposable Elements Repbase Update , a database of eukaryotic repetitive elements. Cytogenetic and Genome Research. 467, 462–467 (2005). doi:10.1159/000084979

25. Tarailo-graovac, M. & Chen, N. Using RepeatMasker to Identify Repetitive Elements in Genomic Sequences. Current Protocols in Bioinformatics. 1–14 (2009). doi:10.1002/0471250953.bi0410s25
26. Burge, C. & Karlin, S. Prediction of Complete Gene Structures in Human Genomic DNA. J. Mol.Biol. 78–94 (1997). doi:10.1006/jmbi.1997.0951
27. Stanke, M. & Waack, S. Gene prediction with a hidden Markov model and a new intron submodel. Bioinformatics. 19, 215 – 225 (2003). doi:10.1093/ bioinformatics/btg1080
28. Majoros, W. H., Pertea, M. & Salzberg, S. L. TigrScan and GlimmerHMM : two open source ab initio eukaryotic gene-finders. Bioinformatics. 20, 2878–2879 (2004). doi: 10.1093/bioinformatics/bth315
29. Blanco, E., Parra, G. & Guigó, R. Using geneid to Identify Genes. Curr. Protoc. Bioinforma. 1–28 (2007). doi:10.1002/0471250953.bi0403s18
30. Korf, I. Gene finding in novel genomes. BMC Bioinformatics. 9, 1–9 (2004). doi: 10.1186/1471-2105-5-59
31. Keilwagen, J. et al. Using intron position conservation for homology-based gene prediction. Nucleic acids research. 1–11 (2016). doi:10.1093/nar/gkw092
32. Keilwagen, J., Hartung, F., Paulini, M., Twardziok, S. O. & Grau, J. Combining RNA-seq data and homology-based gene prediction for plants, animals and fungi. Bioinformatics. (2018). doi:10.1186/s12859-018-2203-5
33. Kim, D., Langmead, B. & Salzberg, S. L. HISAT : a fast spliced aligner with low memory requirements. Nat. Methods (2015). doi:10.1038/nmeth.3317
34. Pertea, M. et al. StringTie enables improved reconstruction of a transcriptome from RNA-seq reads.Nature Biotechnology. (2015). doi:10.1038/nbt.3122
35. Tang, S., Lomsadze, A., Borodovsky, M. & Tech, J. G. Identification of protein coding regions in RNA transcripts.Nucleic Acids Research. 43, 1–10 (2015). doi:10.1093/nar/gkv227
36. Campbell, M. A., Haas, B. J., Hamilton, J. P., Mount, S. M. & Buell, C. R. Comprehensive analysis of alternative splicing in rice and comparative analyses with Arabidopsis. Bmc Genomics. 17, 1–17 (2006). doi:10.1186/1471-2164 -7-327
37. Haas, B. J. et al. Open Access Automated eukaryotic gene structure annotation using EVidenceModeler and the Program to Assemble Spliced. Genome Biology. 9, 1–22 (2008). doi:10.1186/gb-2008-9-1-r7
38. Griffiths-jones, S. et al. Rfam: annotating non-coding RNAs in complete genomes. Nucleic Acids Research. 33, 121–124 (2005). doi:10.1093/nar/gki081
39. Lowe, T. M. & Eddy, S. R. tRNAscan-SE : a program for improved detection of transfer RNA genes in genomic sequence. Nucleic Acids Research. 25, 955–964 (1997). doi:10.1093/nar/25.5.0955
40. She, R., Chu, J. S., Wang, K., Pei, J. & Chen, N. genBlastA : Enabling BLAST to identify homologous gene sequences. Genome Research. 143–149 (2009). doi:10.1101/gr.082081.108.4
41. Birney, E., Clamp, M. & Durbin, R. GeneWise and Genomewise. Genome Research. 988–995 (2004). doi:10.1101/gr.1865504.quickly
42. Marchler-bauer, A. et al. CDD : a Conserved Domain Database for the functional annotation of proteins. Nucleic Acids Research. 39, 225–229 (2011). doi: 10.1093/nar/gkq1189
43. Koonin, E. V et al. A comprehensive evolutionary classification of proteins encoded in complete eukaryotic genomes. Genome Biology. 5, (2004). doi:10.1186/gb-2004-5-2-r7
44. Dimmer, E. C. et al. The UniProt-GO Annotation database in 2011. nucleic Acids Research. 40, 565–570 (2012). doi:10.1093/nar/gkr1048.
45. Kanehisa, M. & Goto, S. KEGG: Kyoto Encyclopedia of Genes and Genomes. Nucleic Acids Research. 28, 27–30 (2000).
46. Boeckmann, B. et al. The SWISS-PROT protein knowledgebase and its supplement TrEMBL in 2003. Nucleic Acids Research. 31, 365–370 (2003). doi: 10.1093/nar/gkg095.
47. Altschup, S. F., Gish, W., Pennsylvania, T. & Park, U. Basic Local Alignment

Search Tool 2Department of Computer Science. J. Mol. Biol. 403–410 (1990).
48. Wang, J. et al. Genome-Wide Analysis of the Distinct Types of Chromatin Interactions in Arabidopsis thaliana. Plant Cell Physiol. 2, 57−70 (2017). doi:10.1093/pcp/pcw194
49. DePamphilis, C. W. et al. The Amborella genome and the evolution of flowering plants. Science (80-.). 342, (2013). doi:10.1126/science.1241089
50. Tuskan, G. A. et al. The genome of black cottonwood, Populus trichocarpa (Torr. & Gray). Science (80-.). 313, 1596–1604 (2006). doi:10.1126/science.1128691
51. Huang, S. et al. Draft genome of the kiwifruit Actinidia chinensis. Nat. Commun. 4, (2013). doi: 10.1038/ncomms3640.
52. Zhang, J. et al. Stress response proteins' differential expression in embryogenic and non-embryogenic callus of Vitis vinifera L. cv. Cabernet Sauvignon-A proteomic approach. Plant Sci. 177, 103–113 (2009). doi:10.1016/j.plantsci. 2009.04.003
53. Argout, X. et al. The genome of Theobroma cacao. Nat. Genet. 43, 101–108 (2011). doi:10.1038/ng.736.
54. Li, L. et al. OrthoMCL: Identification of Ortholog Groups for Eukaryotic Genomes. Genome Research. 2178–2189 (2003). doi:10.1101/gr.1224503
55. Gascuel O. New Algorithms and Methods to Estimate Maximum-Likelihood Phylogenies: Assessing the Performance of PhyML 3.0[J]. Systematic Biology. 2010, 59(3):307-321. doi:10.1093/sysbio/syq010
56. Sudhir K, Glen S, Michael S, et al. TimeTree: A Resource for Timelines, Timetrees, and Divergence Times[J]. Molecular Biology & Evolution, 2017(7):1812. doi:10.1093/molbev/msx116
57. Bie, T. De, Cristianini, N., Demuth, J. P. & Hahn, M. W. CAFE : a computational tool for the study of gene family evolution. Bioinformatics. Bioinformatics. 22, 1269 – 1271 (2006). doi:https://doi.org/10.1093/ bioinformatics/btl097
58. Schabauer, H., Valle, M., Pacher, C. & Stockinger, H. SlimCodeML : An Optimized Version of CodeML for the Branch-Site Model. IEEE Computer Society (2012). doi:10.1109/IPDPSW.2012.88
59. Prestridge, D. S. SIGNAL SCAN: a computer program that scans DNA sequences for eukaryotic transcriptional elements. Computer Applications in the Biosciences Cabios. 7, 203–206 (1991). doi:10.1093/bioinformatics/7.2.203
60. Edgar, R. C., Drive, R. M. & Valley, M. MUSCLE: multiple sequence alignment with high accuracy and high throughput. Nucleic Acids Research. 32,1792–1797 (2004).doi: 10.2460/ajvr.69.1.82
61. Zhang et al., Genomes of the Banyan Tree and Pollinator Wasp Provide Insights into Fig-Wasp Coevolution, Cell (2020), https://doi.org/10.1016/j.cell. 2020.09.043
62. Montero-Pau J , Blanca J , Bombarely A , et al. De-novo assembly of zucchini genome reveals a whole genome duplication associated with the origin of the Cucurbita genus[J]. Plant Biotechnology Journal, 2018, 16(6). doi: 10.1111/pbi.12860
63. He J; Bao S; Deng J; Li Q; Ma S; Liu Y; Cui Y; Zhu Y; Wei X; Ding X; Ke K; Chen C (2022): Supporting data for "A chromosome-level genome assembly of Artocarpus nanchuanensis (Moraceae), an extremely endangered fruit tree" GigaScience Database. http://dx.doi.org/10.5524/102200.

Figures

Fig. 1 The flowchart of *A. nanchuanensis* genome assembly and annotation process.

Fig. 2 The *A. nanchuanensis* sample and genomic interaction analysis.

Fig. 3 The analysis of Hi-C library construction and heat map.

Fig. 4 The Nr homologous species distribution of *A. nanchuanensis*.
Fig. 5 The phylogenetic and gene families analysis of *A. nanchuanensis* and related species.
Fig. 6 The 4DTV distribution and LTR insertion time analysis among *A. nanchuanensis* and other related species.

Supplementary Table

Supplementary Table 1 The clean data and genome comparison results of *A. nanchuanensis*.
Supplementary Table 2 The Hi-C sequencing data types and proportion of *A. nanchuanensis*.
Supplementary Table 3 The repeat sequences analysis of *A. nanchuanensis*.
Supplementary Table 4 The gene prediction results of *A. nanchuanensis*.
Supplementary Table 5 Pseudogene annotation statistics of *A. nanchuanensis*.
Supplementary Table 6 The statistical results of non-coding RNA.
Supplementary Table 7 Functional annotation statistics of *A. nanchuanensis*.

Supplementary figure

Supplementary Fig. 1 The Kmer distribution map of *A. nanchuanensis.*
Supplementary Fig. 2 The BUSCO genome assembly evaluation.
Supplementary Fig. 3 Distribution of the number of genes among the three methods.
Supplementary Fig. 4 The KOG functional annotation classification of *A. nanchuanensis*.
Supplementary Fig. 5 The GO secondary node annotation classification of *A. nanchuanensis.*
Supplementary Fig. 6 The family clustering statistics among different species.
Supplementary Fig. 7 The classification annotation statistics for GO.

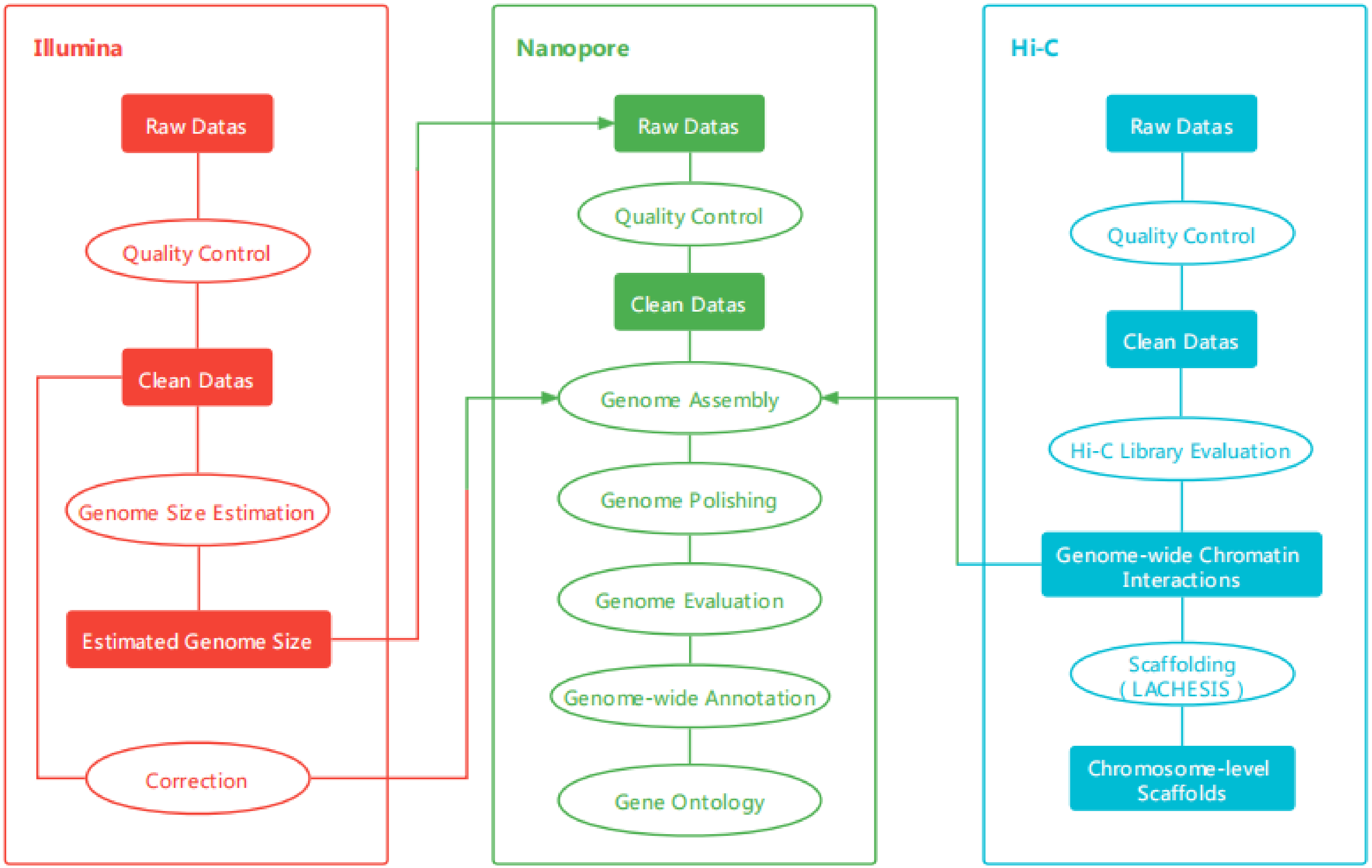

Fig. 1 The flowchart of *A. nanchuanensis* genome assembly and annotation process.

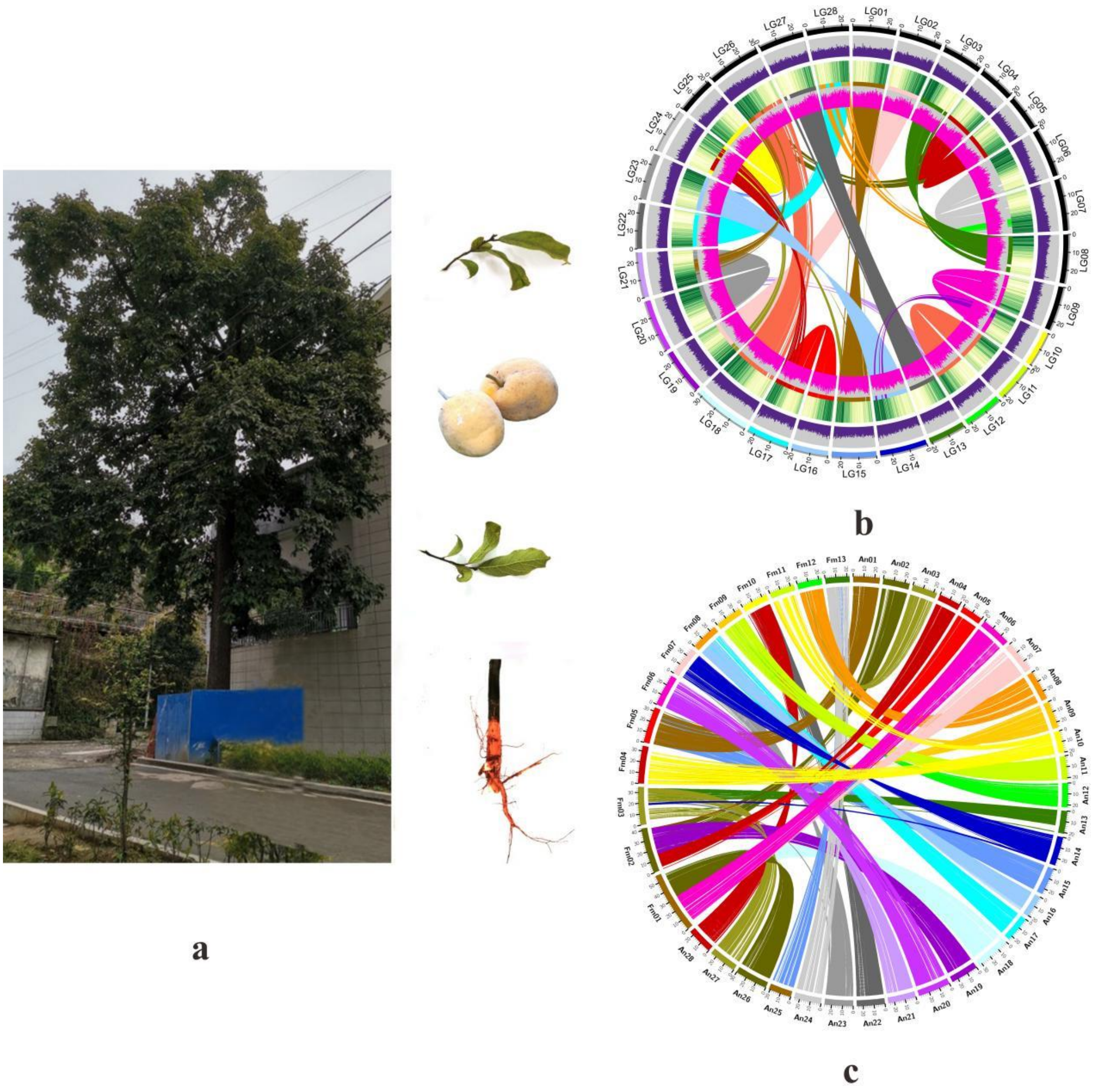

Fig. 2 The *A. nanchuanensis* sample and genomic interaction analysis.

Note: (a) The picture of the *A. nanchuanensis* tree used in this study, and the picture was taken in 2019. (b) Circos plot of *A. nanchuanensis* 28 chromosomes. The tracks from outside to inside are 28 chromosome-level scaffolds, the GC content of chromosomes (purple), the gene density of chromosomes (green), the TE ratio of chromosomes (pink), and the lines of different colors in the innermost circle represent the collinearity within themselves. (c) Genes collinearity circle of *A. nanchuanensis* and *F. microcarpa*. Each color represents a collinear block of each chromosome, with at least five collinearity genes in each block.

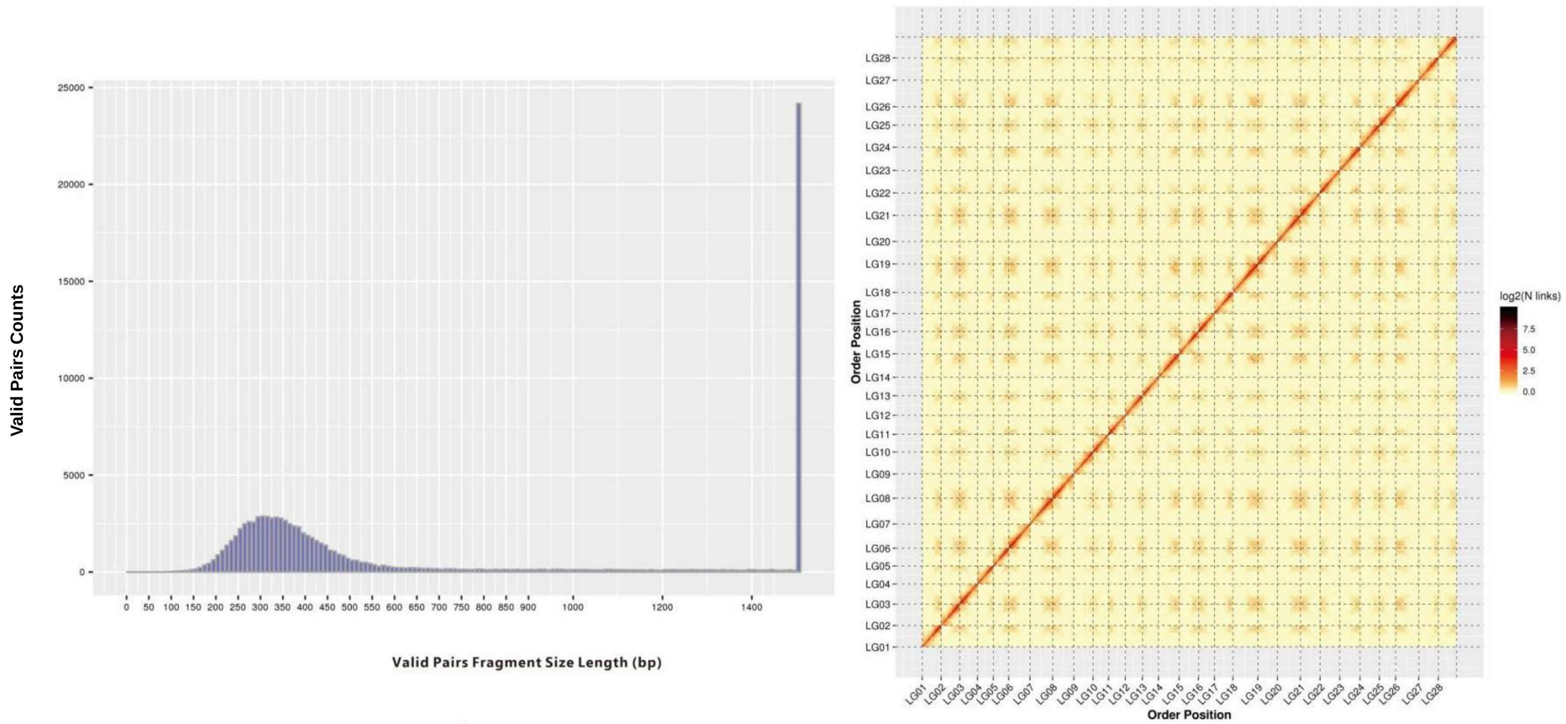

a

b

Fig. 3 The analysis of Hi-C library construction and heat map.

Note: a shows the length distribution of Hi-C library insert fragment, X-axis represents the sum of the distance between double ends reads on the assembly genome and the nearest enzyme-cutting site, Y-axis represents the reads number. b shows the interaction heat map of Hi-C links among chromosome groups for *A. nanchuanensis*, the assembled genome of *A. nanchuanensis* was divided into 100-kb non-overlapping windows (or bins), and valid interaction links of Hi-C data were calculated between each pair of bins. The binary logarithm of each link number is coded using colors ranging from light yellow to dark red, indicating the frequency of Hi-C interaction links from low to high. LG01-LG28 represents the 28 chromosome groups inferred by LACHESIS; The X-axis and Y-axis represent the order of each bin on the corresponding chromosome group.

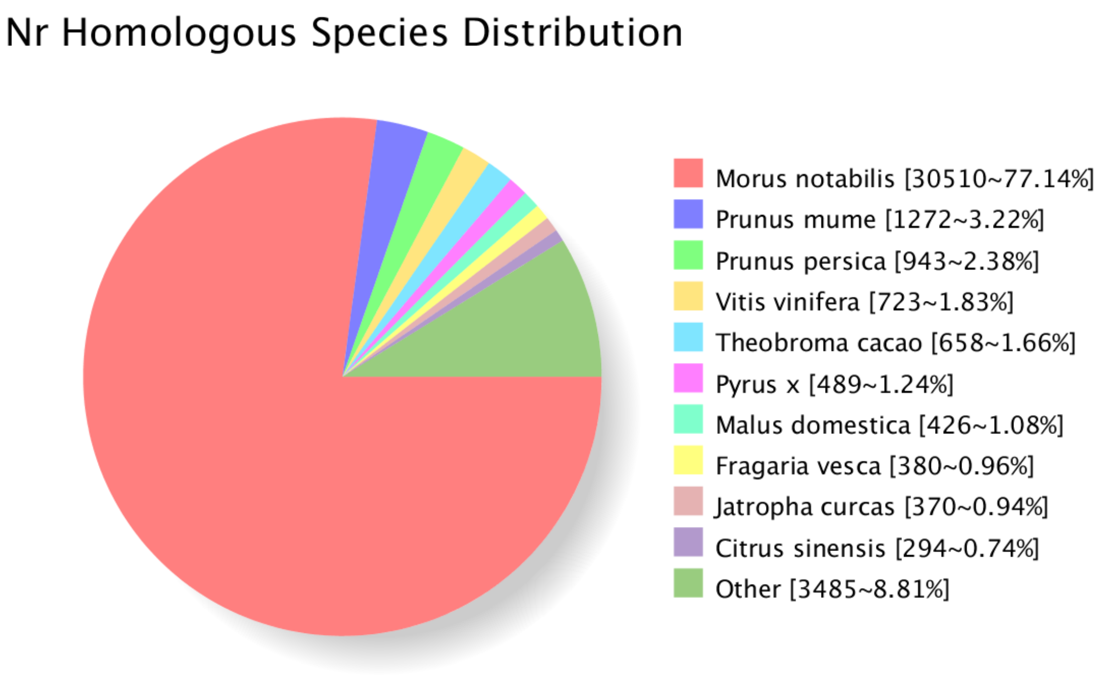

Fig.4 The Nr homologous species distribution of *A. nanchuanensis*.

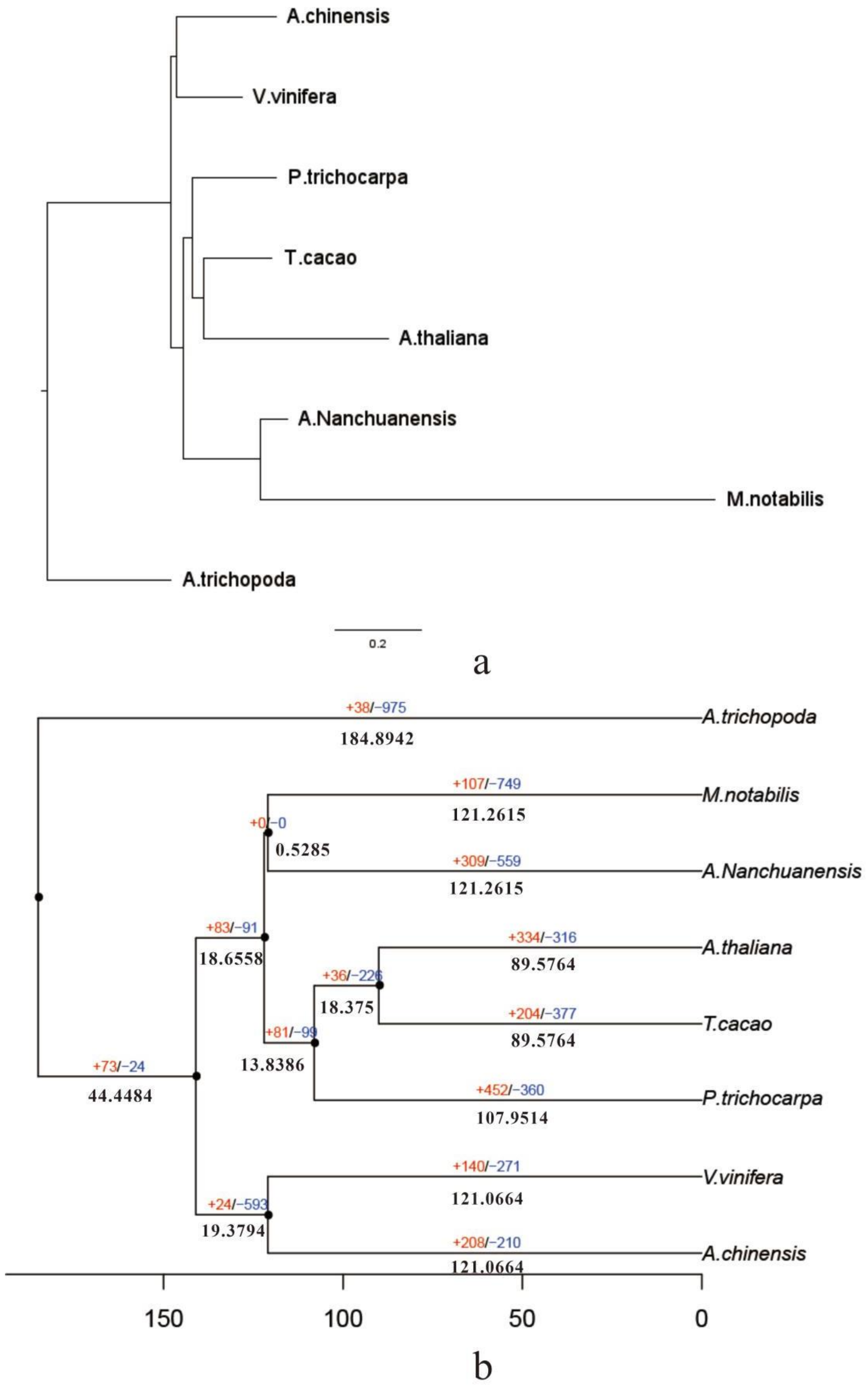

Fig.5 The phylogenetic and gene families analysis of *A. nanchuanensis* and related species. Note: a represent species phylogenetic analysis of *A. nanchuanensis* and related species. b represent analysis of species differentiation time and gene families in contraction and expansion. black font represents the temporal relationship of species differentiation, unit is million years. "+" represents the number of gene families expanding on this node, "-" represents the number of gene families contracting on this node, and the black dot refers to the common ancestor.

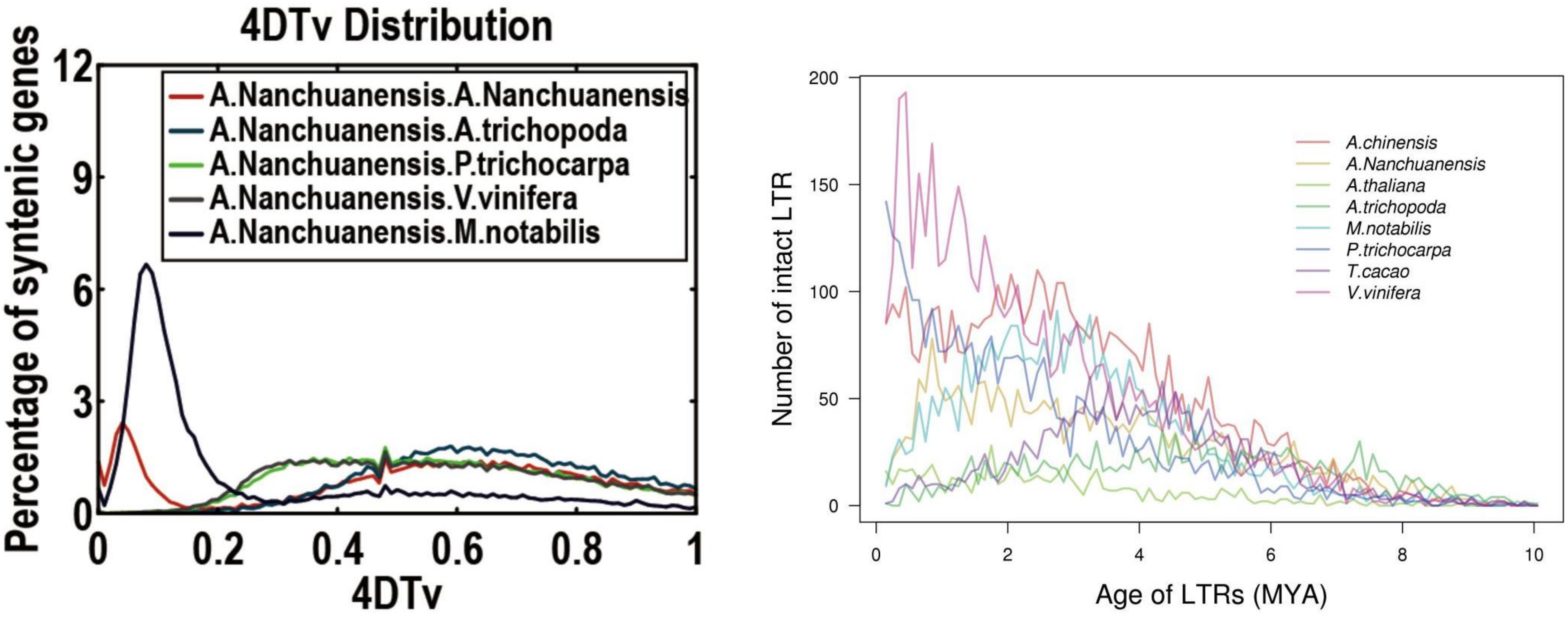

a **b**

Fig. 6 The 4DTV distribution and LTR insertion time analysis among *A. nanchuanensis* and other related species.

Note: a represent 4DTV distribution analysis among A. nanchuanensis and other four species, the x axis represents the mutation rate of homologous genes to 4DTV, and the Y axis represents the proportion of homologous gene pairs. B represents the analysis of LTR insertion time among *A. nanchuanensis* and other seven species.

Click here to access/download
Supplementary Material
Supplementary table 2-6.docx

Click here to access/download
Supplementary Material
supplementary figure 2-6.docx

Click here to access/download
Supplementary Material
response to nanchuan revised 2-6.docx

Personal Cover Click here to access/download;Personal Cover;cover letter.docx

Dear Editors:

We would like to submit an original article entitled "Chromosome genome assembly and annotation of Artocarpus Nanchuanensis with Nanopore and Hi-C sequencing data" for consideration for publication in GigaScience.

The Artocarpus Nanchuanensis (Moraceae) is an extremely endangered tree species in China. Its fruit and bark have been used as the treatment for skin in Chongqing Nanchuan for a long time, and the fruit has a good control effect on the constipation and other intestinal diseases, those features persistent cause the attention of the researchers, but the molecular mechanisms involved is little known. In our work, we revealed a high-quality chromosome-scale genome assembly and annotation for Artocarpus Nanchuanensis with Nanopore and Hi-C sequencing data. The disclosure of Artocarpus Nanchuanensis genome sequence information provides an important resource to expand our understanding of the molecular mechanism in its unique biological processes and nutritional, medicinal benefits.

This article has not been published elsewhere in whole or in part. All authors have read and approved the content, and agree to submit for consideration for publication in GigaScience. There is not any conflict involved in the article. I hope this paper is suitable for GigaScience.

We deeply appreciate your consideration of our manuscript, and we look forward to receiving comments from the reviewers. If you have any queries, please don't hesitate to contact me at the address below.

With kind regards

Yours sincerely

Xianping Ding

Corresponding Author:

Xianping Ding

Key Laboratory of Bio-Resources and Eco-Environment of Ministry of Education, College of Life Sciences, Sichuan University, Chengdu 610065, Sichuan, P.R.China;

Tel. and Fax: +86-028-85413096; Email: brainding@scu.edu.cn
