# Supplementary material for: A chromosome-level genome assembly of Artocarpus nanchuanensis (Moraceae), an extremely endangered fruit tree
Source: Gigascience. 2022 Jun 14;11:giac042. doi: 10.1093/gigascience/giac042 (PMC9197682; doi:10.1093/gigascience/giac042)
Supplement: giac042_Supplemental_Figures_and_Tables [file giac042_supplemental_figures_and_tables.zip › supplementary figure 2-6.docx]

*
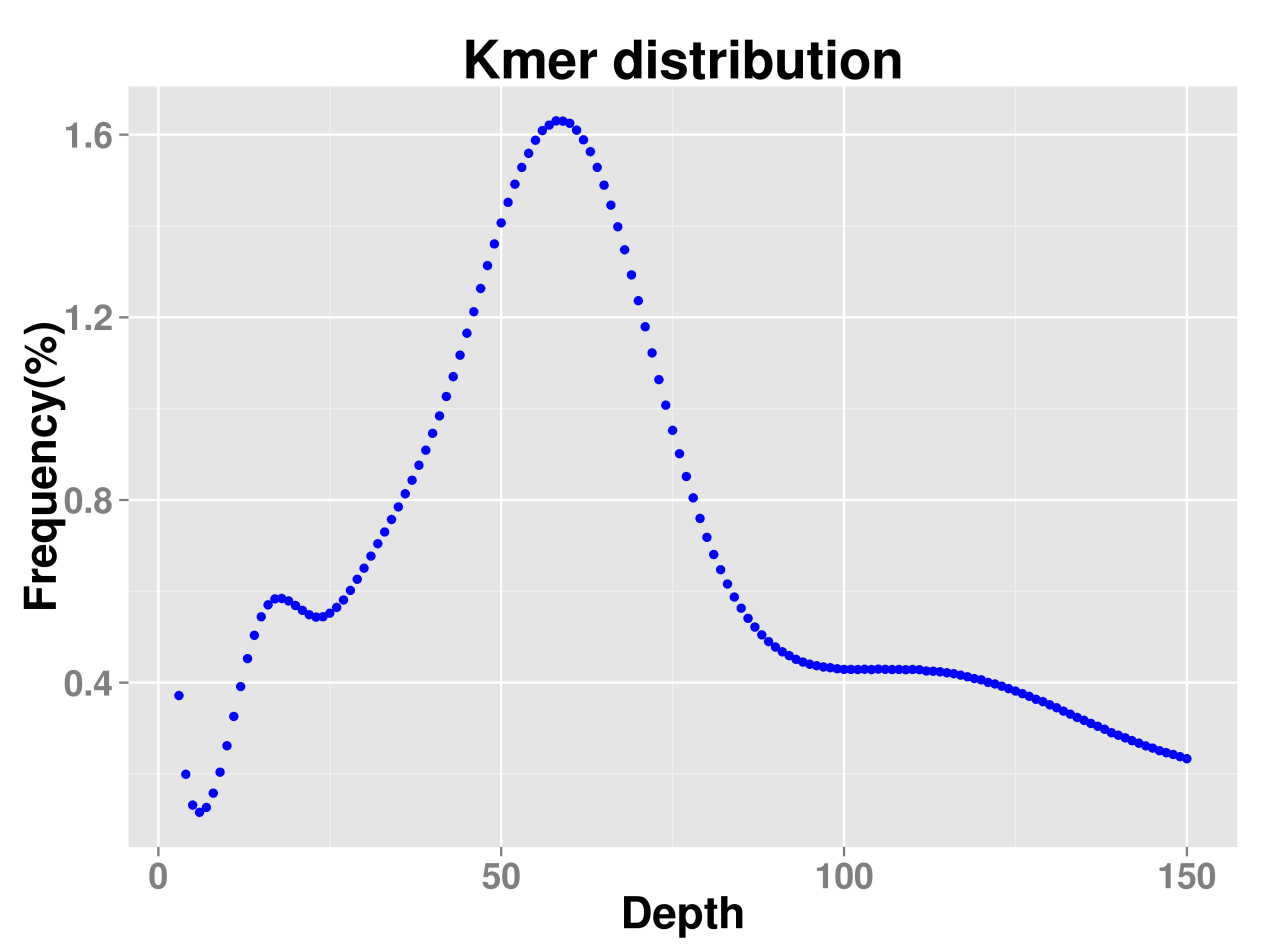
*

Supplementary Fig. 1 The Kmer distribution map of *A. nanchuanensis.*


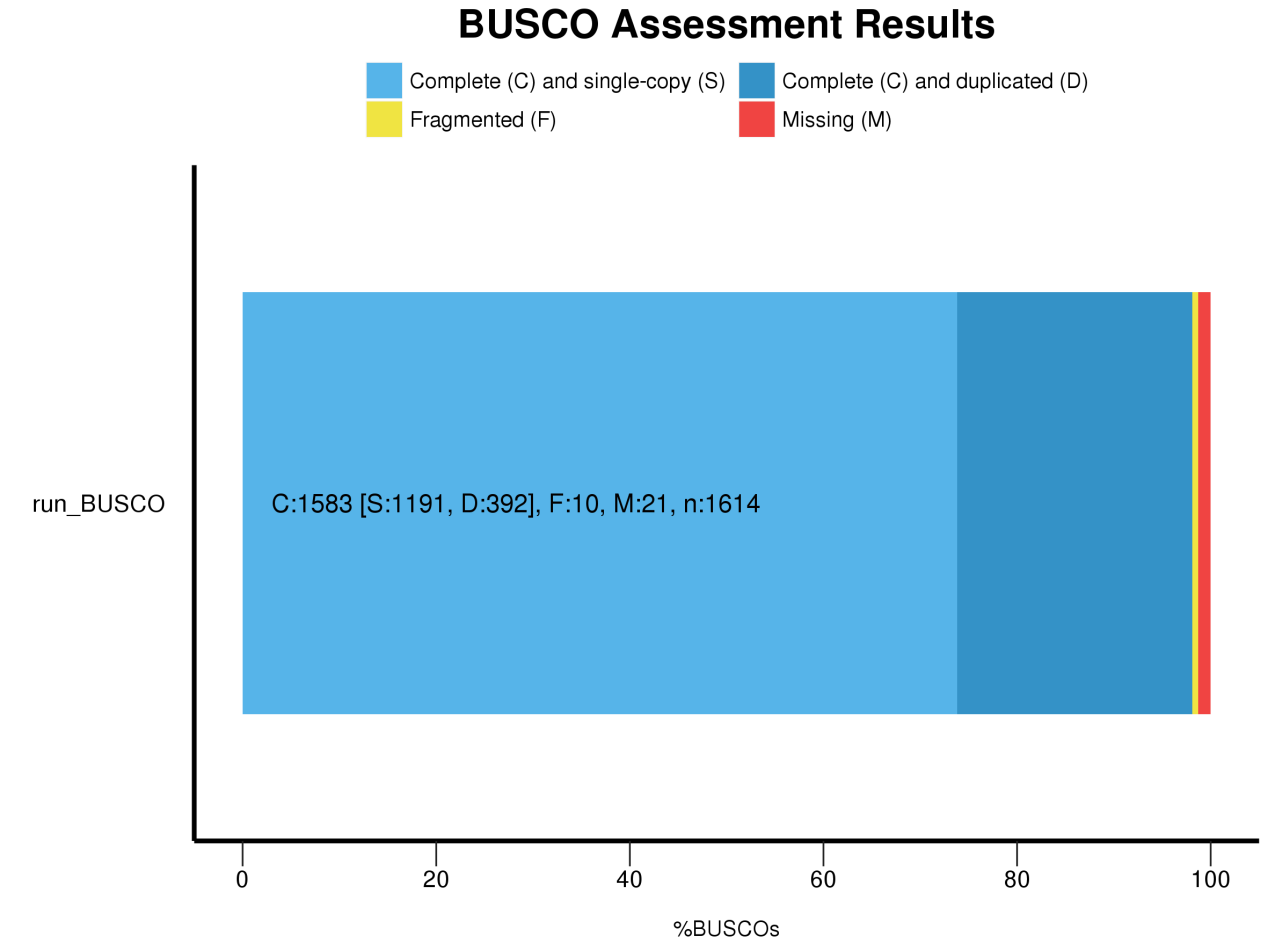


Supplementary Fig. 2 The BUSCO genome assembly evaluation.


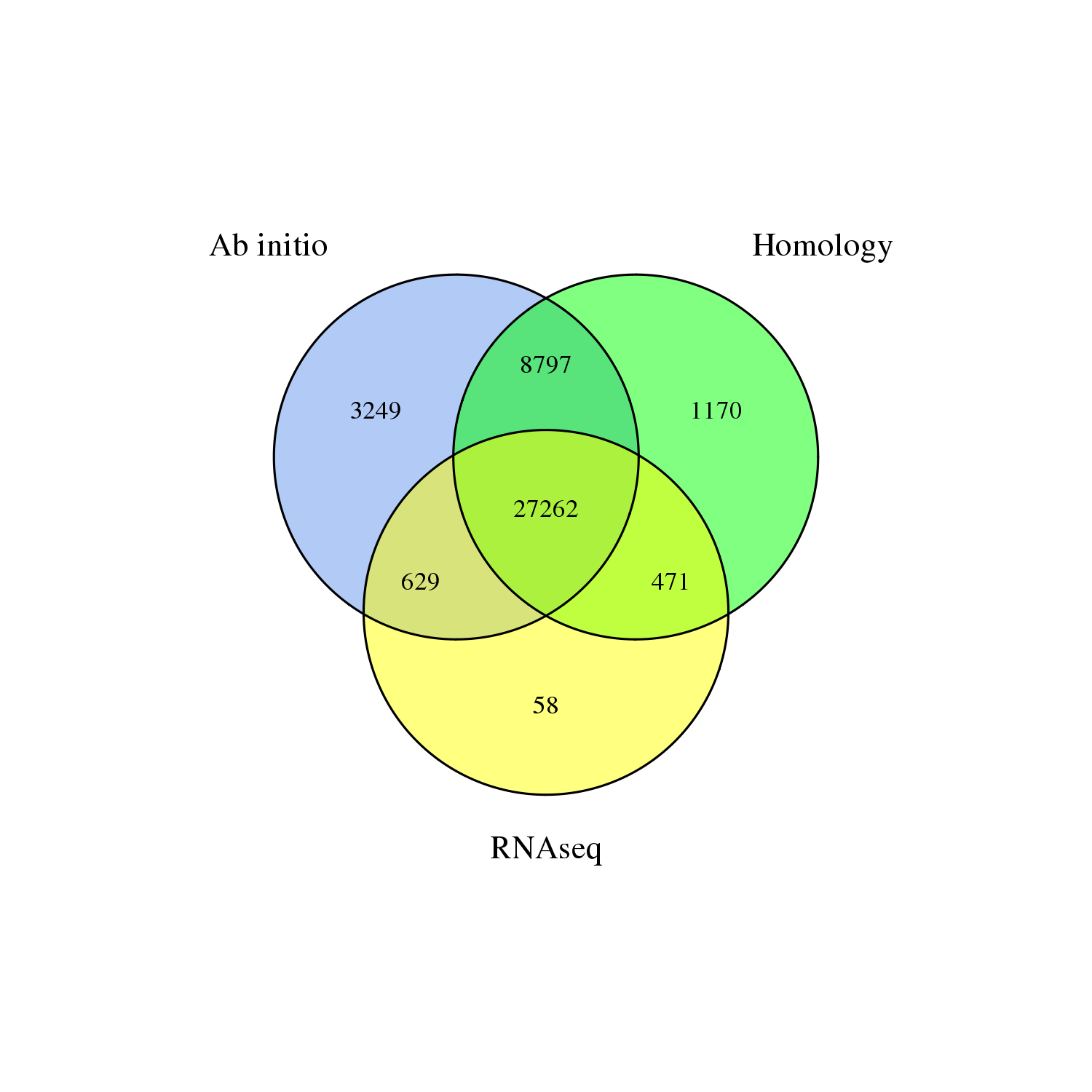


Supplementary Fig. 3 Distribution of the number of genes among the three methods.


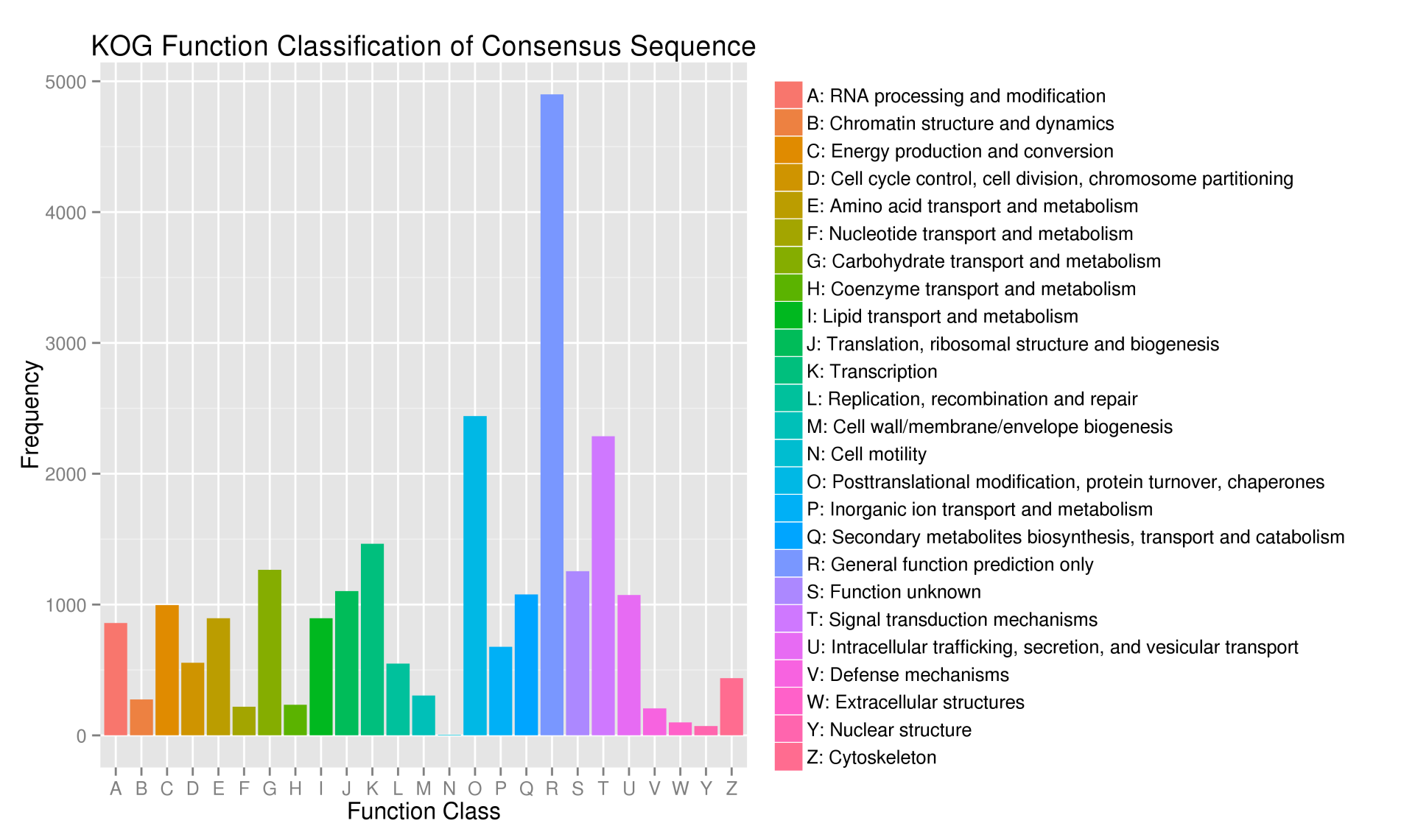


Supplementary Fig. 4 The KOG functional annotation classification of *A. nanchuanensis*.

Note: The X-axis is the contents of each KOG classification, and Y-axis is the number of genes.


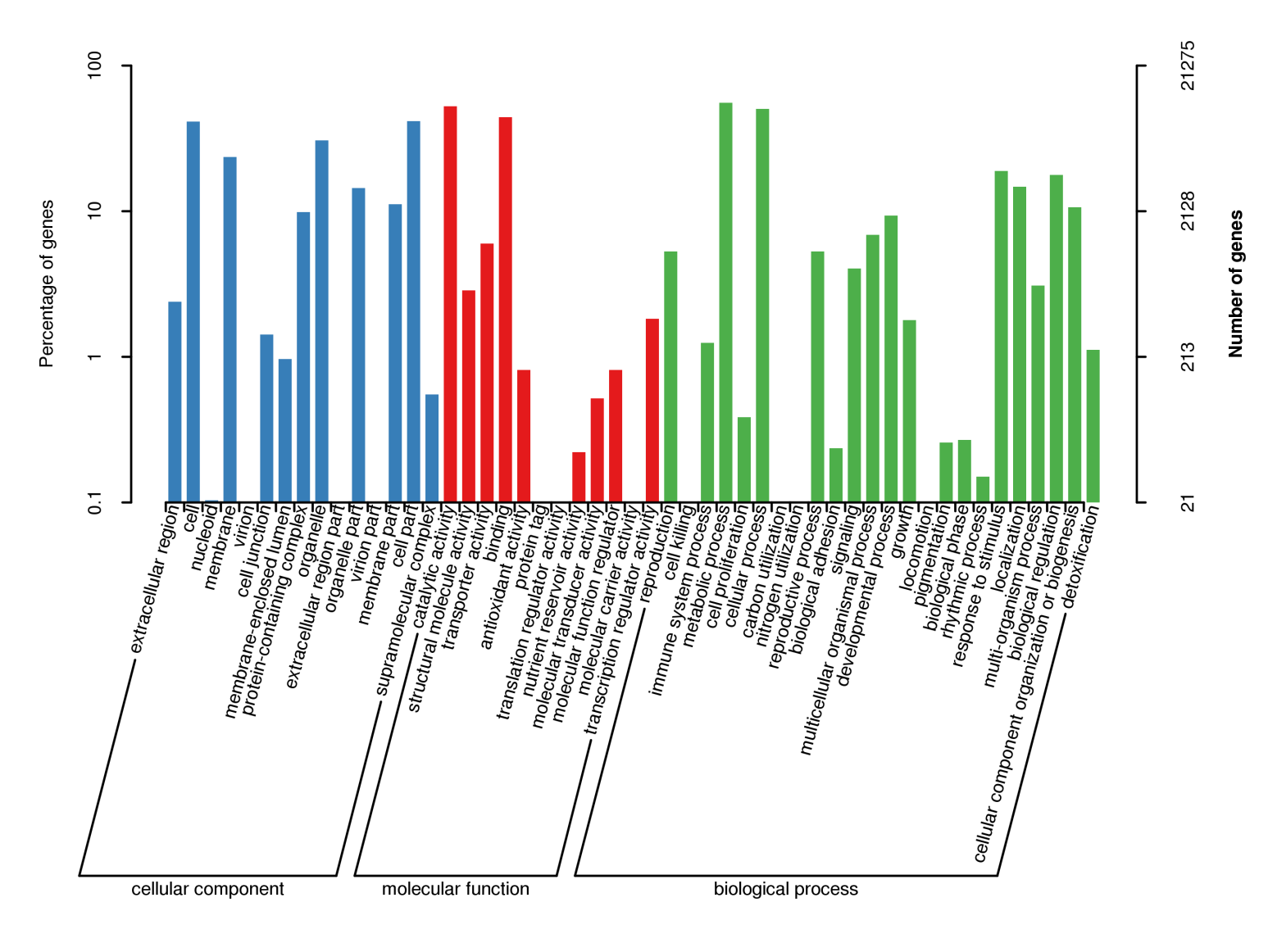


Supplementary Fig. 5 The GO secondary node annotation classification of *A. nanchuanensis.*

Note: The X-axis is the contents of each category of GO; The Y-axis mean the percentage of the number of genes; And the right side is the number of genes. This figure shows the enrichment of each secondary function of gene GO.


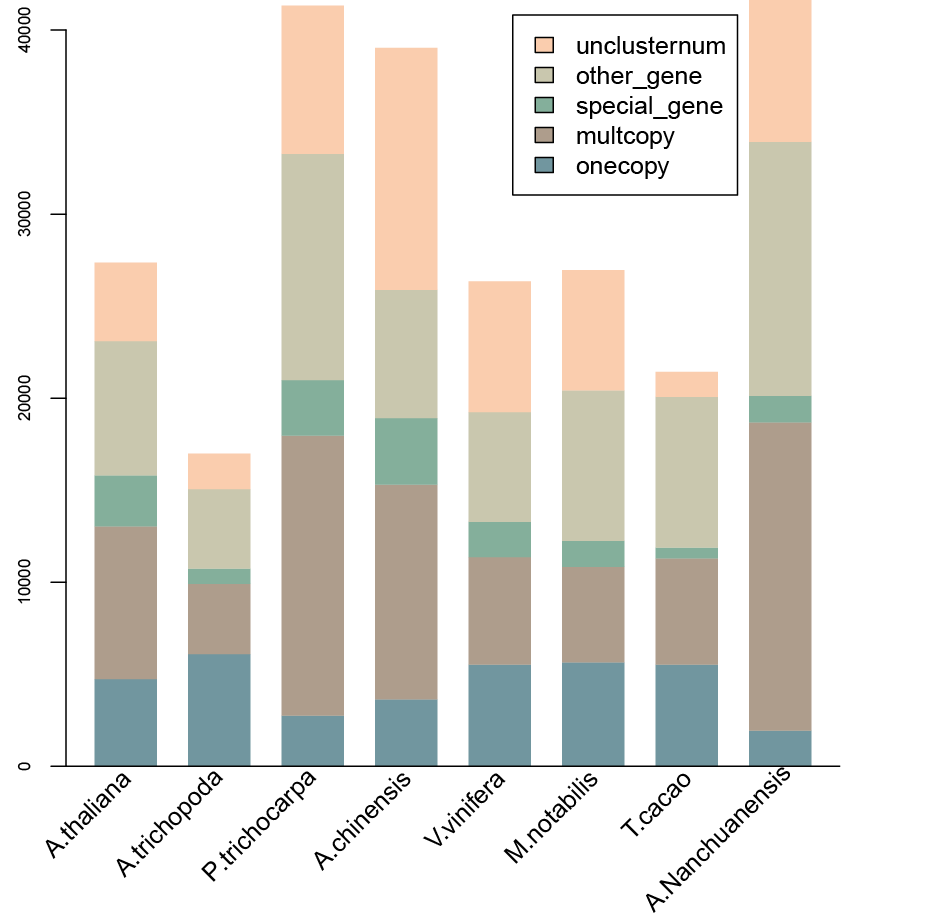


Supplementary Fig. 6 The family clustering statistics among different species.

Note: X-axis mean the species name; Y-axis mean the number of genes; Unclusternum mean the gene of unclustered to any family; Other gene mean all other genes; Special gene: the species specific genes in the family; Multcopy: the number of multiple copies of homologous genes in a common gene family; Onecopy: the number of single-copy homologous genes in the family of genes shared by species.


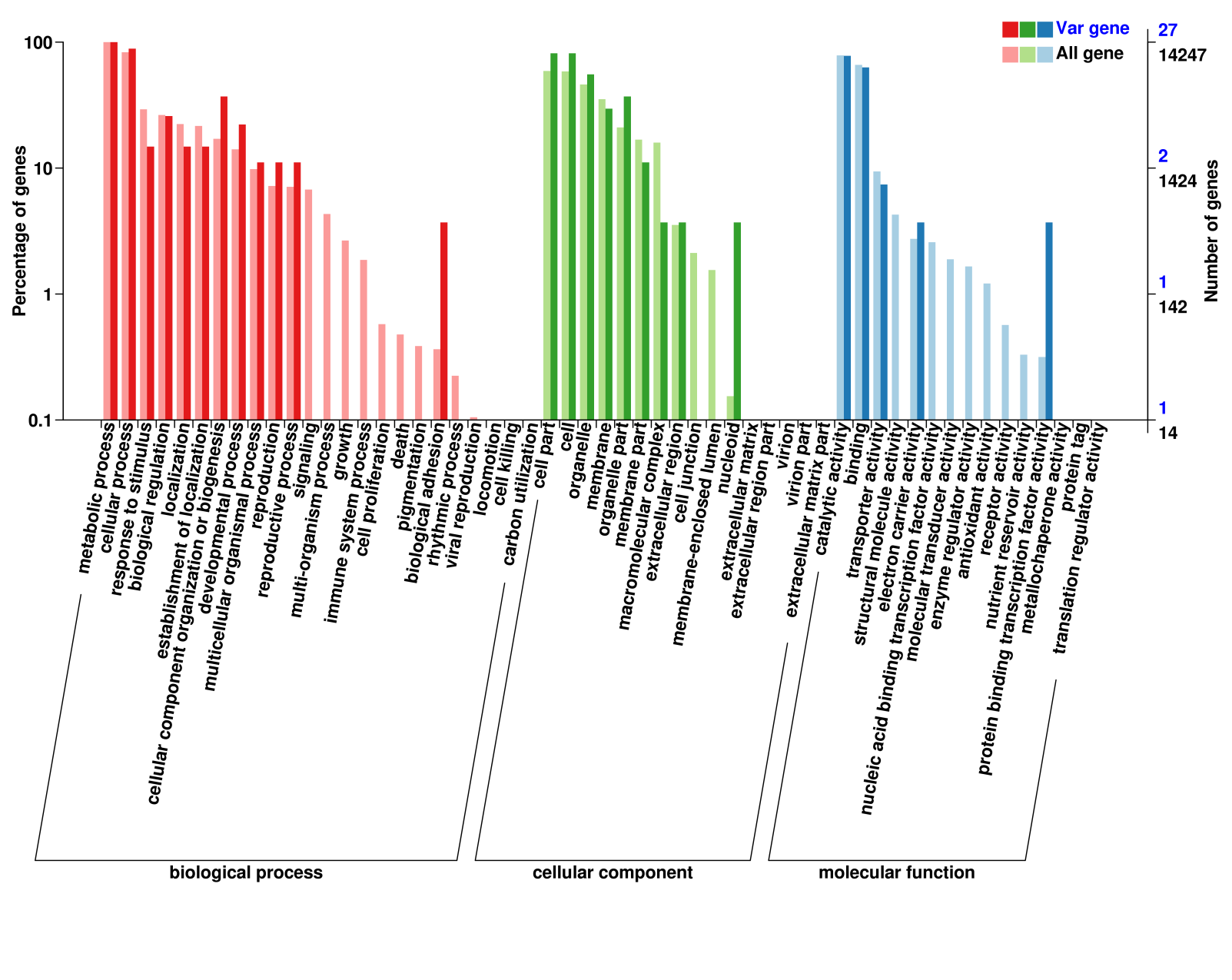


Supplementary Fig. 7 The classification annotation statistics for GO.
