# Supplementary material for: A chromosome-level genome assembly of Artocarpus nanchuanensis (Moraceae), an extremely endangered fruit tree
Source: Gigascience. 2022 Jun 14;11:giac042. doi: 10.1093/gigascience/giac042 (PMC9197682; doi:10.1093/gigascience/giac042)
Supplement: giac042_Supplemental_Figures_and_Tables [file giac042_supplemental_figures_and_tables.zip › Supplementary table 2-6.docx]

**Supplementary Table 1 The clean data and genome comparison results of *A. nanchuanensis*.**

| Library | Mapping Type | Number | Ratio（%） |
| --- | --- | --- | --- |
|  | Total Read Pairs | 458,907,479 | 100 |
|  | Mapped Reads | 728,487,984 | 79.37 |
|  | Unique Mapped Read Pairs | 236,274,160 | 51.45 |

**Supplementary Table 2 The Hi-C sequencing data types and proportion of *A. nanchuanensis*.**

| Library | Type | Number | Ratio (%) |
| --- | --- | --- | --- |
|  | Unique Paired Alignments | 236,274,160 | 100 |
|  | Valid Interaction Pairs | 56,964,635 | 24.11 |
|  | Dangling End Pairs | 138,663,715 | 58.69 |
|  | Re-ligation Pairs | 15,169,250 | 6.42 |
|  | Self-cycle Pairs | 975,544 | 0.41 |
|  | Dumped Pairs | 24,501,016 | 10.37 |

Note: Unique Paired Alignments: the unique read pairs alignment to the genome of A. nanchuanensis; Valid Interaction Pairs: the valid interaction read pairs; Dangling End Pairs: the dangling end read pairs in the invalid data; Re-ligation Pairs: the re-ligation read pairs in the invalid data; Self-circle Ligation Pairs: self-circle ligation read pairs in the invalid data; Dumped Pairs: the dumped read pairs in the invalid data.

**Supplementary Table 3 The repeat sequences analysis of *A. nanchuanensis*.**

| Type | Number | Length | Rate (%) |
| --- | --- | --- | --- |
| ClassI | 715,880 | 354,430,815 | 46.06 |
| ClassI/DIRS | 26,959 | 23,880,069 | 3.10 |
| ClassI/LARD | 238,559 | 75,579,627 | 9.82 |
| ClassI/LINE | 12,327 | 3,488,322 | 0.45 |
| ClassI/LTR/Copia | 210,964 | 147,520,139 | 19.17 |
| ClassI/LTR/Gypsy | 209,038 | 129,742,980 | 16.86 |
| ClassI/LTR/Unknown | 3,210 | 597,880 | 0.08 |
| ClassI/PLE | 1,322 | 456,964 | 0.06 |
| ClassI/SINE | 8,910 | 2,272,141 | 0.30 |
| ClassI/TRIM | 4,219 | 1,617,309 | 0.21 |
| ClassI/Unknown | 372 | 66,025 | 0.01 |
| ClassII | 103,050 | 33,806,173 | 4.39 |
| ClassII/Crypton | 17 | 1,003 | 0.00 |
| ClassII/Helitron | 22,320 | 5,961,597 | 0.77 |
| ClassII/MITE | 1,183 | 204,923 | 0.03 |
| ClassII/Maverick | 1,392 | 1,762,270 | 0.23 |
| ClassII/TIR | 66,336 | 25,334,820 | 3.29 |
| ClassII/Unknown | 11,802 | 879,933 | 0.11 |
| PotentialHostGene | 30,328 | 8,227,684 | 1.07 |
| SSR | 10,909 | 2,628,783 | 0.34 |
| Unknown | 187,171 | 55,237,782 | 7.18 |
| Total | 1,047,338 | 422,782,698 | 54.94 |

Note: Type: repeat sequence Type; Number: The Number of repeats that obtained; Length: the total Length of the predicted repeating sequence; Rate (%): the proportion of repetitive sequences in the total genome.

**Supplementary Table 4 The gene prediction results of *A. nanchuanensis*.**

| Method | Software | Species | Gene number |
| --- | --- | --- | --- |
| Ab initio | Genscan | - | 42,670 |
|  | Augustus | - | 47,491 |
|  | GlimmerHMM | - | 74,635 |
|  | GeneID | - | 70,861 |
|  | SNAP | - | 88,742 |
| Homology-based | GeMoMa | Arabidopsis_thaliana | 33,745 |
|  |  | Morus_notabilis | 41,079 |
|  |  | Prunus_avium | 37,127 |
|  |  | Rosa_chinensis | 37,535 |
| RNAseq | TransDecoder | - | 84,498 |
|  | GeneMarkS-T | - | 52,056 |
|  | PASA | - | 48,695 |
| Integration | EVM | - | 41,636 |

**Supplementary Table 5 Pseudogene annotation statistics of *A. nanchuanensis*.**

| Software | Number | Total length | Average length |
| --- | --- | --- | --- |
| GeneWise | 1,905 | 4,825,668 | 2,533.16 |

**Supplementary Table 6 The statistical results of non-coding RNA.**

| RNA classification | Number | Family |
| --- | --- | --- |
| miRNA | 138 | 24 |
| rRNA | 409 | 4 |
| tRNA | 512 | 24 |

Note: RNA classification: The classification of RNA; Number: The predicted RNA number; Family: The RNA family number.

**Supplementary Table 7** **Functional annotation statistics of *A. nanchuanensis*.**

| Annotation database | Annotated number | Percentage (%) |
| --- | --- | --- |
| GO_Annotation | 21,275 | 51.10 |
| KEGG_Annotation | 13,903 | 33.39 |
| KOG_Annotation | 21,567 | 51.80 |
| TrEMBL_Annotation | 39,571 | 95.04 |
| nr_Annotation | 39,553 | 95.00 |
| All_Annotated | 39,596 | 95.10 |

Note: Annotation database: Functional annotation database; Annotated number: the number of genes annotated to the corresponding database; Percentage (%): the percentage of genes annotated to the total number of databases.
