# Supplementary material for: A chromosome-level genome assembly of Artocarpus nanchuanensis (Moraceae), an extremely endangered fruit tree
Source: Gigascience. 2022 Jun 14;11:giac042. doi: 10.1093/gigascience/giac042 (PMC9197682; doi:10.1093/gigascience/giac042)
Supplement: giac042_Response_to_Reviewer_Comments_Original_Submission [file giac042_response_to_reviewer_comments_original_submission.pdf]

Author's Response To Reviewer Comments

Close

We would like to thank the reviewers very much for taking the time and effort to review our paper so thoroughly. We have carefully considered their comments, which have helped us to make improvements to our work. Our point by point responses to their comments are presented below. The reviewers' comments are italicized and our responses are in red regular font. The changes we made in the text are also highlighted in red.
Reviewer: 1
Comments to the Author
Reviewer #1: He et al. contributed their study of "A chromosome-level genome assembly of Artocarpus nanchuanensis". This study present valuable and high quality genome resources for important plant group. The study has significant values for publication. While, problems remain especially in English expression and writing.
I will recommend to accept it after a major revision.
Response: Thanks to reviewers for the valuable comments and suggestions, which helped us improve the overall presentation of our work. English expression and writing have been carefully revised to ensure that the article is well understood.

Major comments:
1. There is significantly large space for the English expression to be improved. Some sentences were not well developed, and some others were wrong in expression.
As an example, can you pick the error in the sentence in line 28? Do you think "persistent" was right here? And I think there is better way to restructure this sentence.
Another example, lines 33 page 1 - line 1 page 2 in the main text, after reading this sentence, I am confused. I would like to know who do include 78.34 billion high-quality bases. It is so huge to have such large number of bases for a genome assembly. It should be the data generated for genome assembly. But, the sentence is truly problematic in expression. Please take care of writing, as it is very important.
Response: Thank you very much. Manusript has been revised and polished by a language company. "Those features persistent cause the attention of researchers" has been revised as "These features have attracted the attention of researchers" and "The draft genome sequence of mulberry tree, including 78.34 billion high-quality bases, were assembled into 330.79-Mb mulberry genome with a scaffold N50 length of 390,115 bp and contig N50 length of 34,476 bp." has been revised as "In the draft genome sequence of the mulberry tree Morus notabilis (M. notabilis), 78.34 Gb of high-quality data were obtained and assembled into a 330.79 Mb mulberry genome with a 390,115 bp scaffold N50 and 34,476 bp contig N50". The typo together with other mistakes have been revised. (please see line 2 of page 3, and line 7-9 of page 3).

2. line numbers are not consistently put, and no page number. It is very hard to put comments.
Response: Thank you, the line numbers and page number in the manuscript have been revised. (Please see manuscript)

3. Other than English expression, the quality of figure and table need to be significantly improved.
Response: We thank the reviewer for the valuable comments and suggestions, the quality of figures and tables have been improved. We replaced all the figures with the high quality edition, and tables have been revised. (Please see Table, Figure, Supplemntary table and Supplemntary figure)

4. Detailed discription is needed for some specific analyses, such us 4DTV, LTR insertion time. Why did you do these analyses? what did you find? How you results could be compared to others? What is new findings, what is the differences and the commons?
Response: Thank for the valuable comments and suggestions. Transversions on fourfold degenerate synonymous sites (4DTv) are neutral genetic distances that can be used to estimate the relative timing of evolutionary events. According to the homologous gene pairs between two species or within species themselves, the ratio of each homologous gene to the 4DTV mutation site was calculated, and a 4DTV distribution map was made. The peak of the 4DTv distribution among A. nanchuanensis and M. notabilis

was closer to the current than that of A. nanchuanensis and other species, indicating that the differentiation time of A. nanchuanensis and M. notabilis appeared recently, suggesting a closer genetic relationship between them. At ancient time, the 4DTV distribution curves of A. nanchuanensis and other species were similar, which reflected these species might share similar whole-genome duplication (WGD) events. Moreover, the 4DTV distribution of A. nanchuanensis had a small peak at 0.05, which suggested that some genomic fragments duplicated recently. The purpose of long terminal repeat (LTR) accumulation is to cope with stresses, and LTR accumulation is able to reflect that the species may cope with some environmental stresses on its survival. LTR insertion time among A. nanchuanensis and other 7 related species show that the living environment of A. nanchuanensis is relatively stable. The narrow peak of LTR insertion time around 1 Mya indicated some environment stress or environment change has been imposed on A. nanchuanensis and the living environment. ((please see line 34-35 of page 11, and line 1-17 of page 12)

5. A general discussion or comparsion to related results previously published is needed for many parts, like quality and characterization of genome assembly, phylogeny, gene families in expansion and contraction, divergent time, 4DTV (if whole genome duplication is an objective of the authors), LTR insertion time.
Response: Thank you. The general discussion or comparsion to related results previously published of genome assembly quality and characterization, phylogeny, gene families in expansion and contraction, divergent time, 4DTV, LTR insertion time have been added in the manuscript, they helped us to improve the overall presentation of our work. (please see line 11-16 of page 8, line 19-34 of page 10, line 1, 12-31, 34-35 of page 11, line 1-17 of page 12, and line 7-13 of page 13)

6. Conclusion should not be the only summary of results. You should point out key points/values for future/related fields. And most probably, the significance and implications of this study should be provided. Or any shortage/limiation you could see.
Response: Thank you, the conclusion has been revised, according to the comments and suggestions of reviewers The points/values for future/related fields, and the significance and implications of this study have been provided in the manuscript. (please see line 19-35 of page 12 and line 1-20 of page 13).

7. No problem found for open data sharing, by checking the liked data the authors provided.
Response: Thanks you very much.

Minor ones:
1. line 24, do you think the reference No 12 is refered here? or just the No 1 and No 2?
Response: Thank you very much. The reference No 12 in line 24 is refered as the No 1 and No 2 reference. The typo wrong together with other mistakes have been revised. (please see line 32 of page 2)

2. On Latin names. please change such "A.nanchuanensis" expression to "A. nanchuanensis". This means you may need to add a space after ".".
Response: Thank you, and the typo together with other mistakes have been revised.

3. lines 21-24, A.nanchuanensis -> Artocarpus nanchuanensis
line 2, Artocarpus.nanchuanensis -> Artocarpus nanchuanensis
Response: Thank you. The typo together with other mistakes have been revised. (please see line 2 of page 2, line 28 of page 2)

4. line 32, "Moraceae Mulberry and Paper Mulberry", do not put them in Italic, if they are not Latin names.
Response: Thank you. "Moraceae Mulberry and Paper Mulberry" has been revised as "Morus notabilis" and "Broussonetia papyrifera". The typo together with other mistake have been revised. (please see line 7, 11 of page 3)

5. last paragraph in Introduction, the sentence is confusing. One confusiong woule be I do not know who could provide the necessary resources for the genome size selection. And do you think genome size selection is important? And many more confusions here. Please improve the expression.
Response: Thank you. The above confusion is caused by the typo mistake, and "that not only provide the necessary resources for the genome size selection" has been revised as "These genomic data not only provide the necessary resources for the determination of genome size". (please see line 20-21 of page 3)

6. Version information should be provided for softwares used in analyses.
Response: Thank you. The version information of softwares used in analyses have been provided in the manuscript. (please see materials and methods)

7. many typos in references list, such as "k - - - mer", no publication year provided,
Response: We thank the reviewer for the valuable comments and suggestions, all the references in the manuscript have been checked carefully to ensure that the year of publication is provided. (please see References)

8. I do not know the requirment from GigaScience on the number of main figures and tables. But, it seems they are too many now in the manuscipt. Please do a reasonable re-examination, and then put some of the figures and tables to the supplementary, such as figure 1. And you may need to merge some of them into a compact figure or tables, such as figre 2 and figure 4. Also, figures (11, 12, 13) need to be improved, they are ugly and it is not hard for improvement.
Response: Thank you. Based on the enlightening comments and suggestions of reviewers, the figures and tables have been rearranged in the manuscript. We have merged some of figures/tables into a compact figure/table, put some figures/tables into supplemntary figure/table, and the quality of figure/table have been significantly improved. (Please see Table, Figure, Supplemntary table and Supplemntary figure)

9. I may be interested to see a talbe comparing the quality/properties of genomes assemblies released for any other Moraceae and the currently reported one, on parameters like sequencing tech and depth, Contig/Scaffold N50, N90, annotated genes, repeat composion.
Response: Thank you. The genomes assemblies quality of A.nanchuanensis and its related Moraceae plants (M. notabilis, B. papyrifera, A. nanchuanensis) have been collated into Table 3. (Please see Table 3)

10. Other than Table 2 & 3, it would be interesting to present mapping rate and genome coverage (like 10 fold coverage) of those ONT and Illumina data used for assembly.
Response: Thank you. The mapping rate and genome coverage of Illumina data are 99.41% and 68.01 fold, the genome coverage of ONT data are 160.34 fold. According to the suggestions of reviewers, the above contents have been added to Table 1. (Please see Table 1)

11. When the plant Latin names shown up for the first time, you may need to show the full names. And the right citations should be provided if genomic data were used in this study, such as the genomic data for A.thaliana Arabidopsis thaliana (L.) Heynh, A.trichopoda Amborella trichopoda, P.trichocarpa populus trichocarpa, A.chinensis Actinidia chinensis Planch, V.vinifera Vitis vinifera L, M.notabilis Morus notabilis Schneid , T.cacao Theobroma cacao L.
Response: We thank the reviewer for the valuable comments and suggestions, the full Latin name of A. thaliana, A. trichopoda, P. trichocarpa, A. chinensis, V. vinifera, M. notabilis and T. cacao have been shown up for the first time, and the right citations of genomic data have been provided in the manuscript. (please see line 32-34 of page 6 and line 1 of page 7).

12. I am wondering some symbols are in format of not a routinely English format (like ； in Section 2.4 line 34).
Response: We thank the reviewer for the valuable comments and suggestions, the format of symbols have been careful checked and revised to ensure the symbol format is right. (please see line 21-24 of page 5)

13. Section 3.5 Comparative genomics line 23, "expanding and contracting gene families" is not good expression. It may be better to be changed to "gene families in contraction and expansion". There are many such bugs in expression across the whole paper. Please check related paper for improvement in English expression.
Response: Thank you for the valuable comments and suggestions, "expanding and contracting gene families" has been replaced as "gene families in contraction and expansion", and related expression bugs have also been checked and modified. (please see line 8-35 of page 11 and line 1-17 of page 12)

Reviewer: 2
Comments to the Author
Reviewer #2: This article is presenting the genome assembly of Artocarpus nanchuanensis. This work could be of great interest because of its endangered status and its nutritional and medicinal value.

The results seem to be of quality but the manuscript has to be improved. The methods seem to be well used. The major concern is that the reading is difficult because the sentences are often too long and contain many writing errors. In addition, the methods lack detailed information.
Here is a list of remarks, questions and comments. This is not an exhaustive list of all the corrections to be made to the manuscript.
Response: We thank the reviewer for the valuable comments and suggestions, the typo together with some other mistakes have been corrected in the revised manuscript.

1. - Abstract Line 5: it could be more suitable to cite the methods as follow: whole genome sequencing using Illumina and Oxford Nanopore Technology platforms and chromosomal conformation capture technique
Response: Thank you very much. Due to the abstract limitations, whole genome sequencing using Illumina and Oxford Nanopore Technology platforms and chromosomal conformation capture technique has been cited in introduction. (Please see line 20 of page 3)

2. - Abstract Line 6: "Nanopore Sequel reads" should be replaced by "Nanopore reads"
Response: We thank the reviewer for the valuable comments and suggestions, "Nanopore Sequel reads" has been replaced by "Nanopore reads". (Please see line 6 of page 2.)

3. - Abstract Line 12: "genome assembly integrity" should be replaced by "genome assembly completeness".
Response: We thank the reviewer for the valuable comments and suggestions, the relevant expressions in the manuscript as "genome assembly completeness" (Please see line 9 of page 5) .

4. - Introduction Lines 12-13: sentence has to be improved. It is not appropriate to write that the genomes "have been made in detail". The two following sentences have to be improved too.
Response: Thank you. The sentence in introduction lines 12-13 and the two following sentences have been revised as "In the draft genome sequence of the mulberry tree Morus notabilis (M. notabilis), 78.34 Gb of high-quality data were obtained and assembled into a 330.79 Mb mulberry genome with a 390,115 bp scaffold N50 and 34,476 bp contig N503. The assembled genome of Broussonetia papyrifera (B. papyrifera) was 386.83 Mb with a 29.48 Mb scaffold N50 and 171.17 Kb contig N504. The genome data analysis of M. notabilis and B. papyrifera provides a theoretical basis for the study of fibre development, lignin and flavonoid metabolism, nitrogen metabolism, important metal tolerance functions and stress resistance evolution, but the genomic details of A. nanchuanensis remain unknown." (Please see line 7-15 of page 2)

5. - Introduction Lines 21-28. Too long sentence. I'm not sure that "high-pass" is the good term.
Response: We thank the reviewer for the valuable comments and suggestions, "high-pass" has been replaced as "High-throughput/resolution chromosome conformation capture" and the long sentences have been revised. (Please see line 16-24 of page 3)

6. - Figure 1:
o Chromatin instead of "Cromatin"
o "Correction" instead of "Crrection"
o "Genome" instead of "Gemone"
o "polishing" instead of "polish"
o What is "Genome evalue"?
o This figure presents a flow chart. The HiC part should be placed after the nanopore part
o Hi-C instead of "Hic"
Response: Thank you. All the typo together with some other mistakes in the Figure 1 have been corrected, and the flow chart has been modified as reviewer’s suggestion. (please see Fig. 1)

7. - Sample and DNA extraction:
o "The samples of genome were young leaves" could be replaced by "For genome sequencing, DNA was extracted from young leaves" for example.
Response: We thank the reviewer for the valuable comments and suggestions, "The samples of genome were young leaves" has been replaced by "For genome sequencing, DNA was extracted from 100 mg young leaves by the CTAB method". (please see line 30-31 of page 3)

o What is the quantity of young leaves used in the CTAB extraction process?
Response: Thank you, the quantity of young leaves used in the CTAB extraction process is 100 mg and the detail of DNA extraction have been added in Sample and DNA, RNA extraction. (please see line 30-

34of page 3)

o Can you more detail this process or provide a reference?
Response: Thank you for the valuable comments and suggestions. The process detail and reference of DNA and RNA extraction have been added in Sample and DNA, RNA extraction. (please see line 30-34 of page 3, and 1-12 of page 4)

o "for transcriptome analysis" could be replaced by "for RNA extraction"
Response; Thank you, "for transcriptome analysis" has been replaced as "for RNA extraction". (please see line 2 of page 4)

o "ONT Library with 20Kb fragment length was constructed following the manufacturer's protocol"
Response: We thank the reviewer for the valuable comments and suggestions. "ONT Library with 20 Kb insertion size were constructed for the Nanopore platform according to the manufacturers' protocols" has been replaced by "ONT Library with a 20 kb fragment length was constructed following the manufacturer's protocol" (please see line 14-15 of page 4)

o the rest of the paragraph should be reworded.
Response: Thank you. According to reviewer's valuable comments and suggestions, the rest of the paragraph has been reworded. (please see line 27-34 of page 3 and line 1-12 of page 4)

o Integrity of DNA was "checked on" pulsed field electrophoresis.
Response: Thank you, "the integrity of DNA was detected by pulsed field electrophoresis" has been replaced by "the integrity of the DNA was checked on pulsed field electrophoresis". (please see line 32-33 of page 3)

o R9 flow cell instead of "R9 cell"
Response: Thank you, "R9 cell" has been replaced as "R9 flow cell". (please see line 19 of page 4)

o Preparation instead of "prep"
Response: Thank you, "prep" has been replaced as "Preparation". (please see line 17 of page 4)

o How do you realize the genome size estimation? Do you estimate the heterozygosity rate?
Response: Thanks for reviewer's valuable comments and suggestions. A total of 51.76 Gb of high-quality A. nanchuanensis data were obtained from the Illumina sequencing platform with an approximately 68× sequencing depth, and the genome size was calculated to be 761.07Mb. Based on 4 ^ K/genome>200, a kmer distribution map of K = 17 was constructed. The amount of repeated sequences content was estimated to be approximately 55.80%, and the heterozygosity was estimated to be approximately 0.93%, indicating that the A. nanchuanensis genome was highly heterozygotic and complex. The above content has been added to Initial characterization of A. nanchuanensis genome. (please see Fig. 3, line 29-35 of page 7).

o How do you perform the read cleaning? Softwares?
Response: We thank the reviewer for the valuable comments and suggestions, the data sequenced by the sequencer is raw data. Clean data obtain from raw data after two steps as fellow: firstly, remove the reads containing joints; secondly, remove low-quality reads with a proportion of N greater than 10% and a mass value of Q≤10 that account for more than 50% of the whole read.

o Figure 2: caption is missing.
Response: Thank you. The caption of Figure 2 has been replaced as "The A. nanchuanensis sample and genomic interaction analysis". (please see Fig. 2)

8. - There is no detail on the RNA extraction (quantity engaged in the extraction, protocol used) and on the sequencing library preparation.
Response: Thank you for the comments and suggestions, the detail of the RNA extraction and sequencing library preparation were added in the Samples and DNA, RNA extraction, Library construction and high-throughput sequencing of Materials and methods. (please see in line 1-12 and line 21-27 of page 4)

9. - Genome assembly and quality assessment:
o "three rounds calibration by racon and Pilon", did you mean assembly correction?
Response: Thank you very much. Yes, "three rounds calibration by racon and Pilon" mean assembly

correction procession.

o "BWA software was used to align short sequences on the reference genome" could be better than this long sentence.
Response: We thank the reviewer for the valuable comments and suggestions, "BWA software was used to compare the short sequences obtained from second-generation sequencing with the reference genome" has been replaced as "BWA software was used to align short sequences on the reference genome". (please see line 6-7 of page 5)

o BUSCO is used to evaluate the completeness of the assembly
Response: Thank you, "CEGMA v2.5 (default parameters) database and the BUSCO v2.0 software were used to evaluated the integrity of the assembled genome" has been replaced as "The CEGMA v2.5 (default parameters) database and BUSCO v4.0.6 (parameters: odb10, -c 24 -e 1e-3) were used to evaluate the completeness of the assembly". (please see line 7-9 of page 5)

10. - In general, there is a lack of validation of the assembly. Can you add some evaluations such as KAT histogramms which reflect the completeness of the assembly process (https://kat.readthedocs.io/en/latest/) and use Merqury to evaluate the quality of the assembly? (https://genomebiology.biomedcentral.com/articles/10.1186/s13059-020-02134-9).
Response: Thanks to the reviewers for the valuable comments and suggestions. Genome assembly completeness evaluation was conducted by second-generation sequencing reads mapped analysis and core gene integrity assessment by bwa, CEGMA v2.59 and BUSCO v4.0.6. Statistical alignment analysis of second-generation sequencing reads showed that clean reads located on the reference genome accounted for 99.41% of the total clean reads (363,371,475/365,545,724). The paired-end sequences of the correct size that were located on the reference genome, accounted for 93.56% of the total clean reads (341,995,184/365,545,724). The core gene integrity assessment was performed by CEGMA v2.59. Here, 445 CEGs were present in assembly, accounting for 97.16% of all CEGs (445/458), while 232 highly conserved CEGs were present in the assembly, accounting for 93.55% of all CEGs (232/248). The database in BUSCO v4.0.6 contains 1,614 conserved core genes, and the number of complete genes present in the assembly is 1583 (98.08%). (please see line 17-26 of page 8 and Fig. 4)

11. - Genome annotation analysis:
o this section contains very long sentences.
o "based on transcriptome data unreferenced assembly": I'm not sure to understand what authors mean.
o "parameter for blast: The e-value" please clarify
o Lines 28-31: please correct the sentence.
Response: Thank you for the valuable comments. Long sentences have been revised as several short sentences, "based on transcriptome data unreferenced assembly" has been revised as "based on the transcriptome data of a nonreference assembly", "parameter for blast: The e-value" has been clarified as "parameter: e-value -e 1e-5", and the sentence in Lines 28-31 has been corrected as "Noncoding RNAs were predicted by different strategies based on their structural characteristics. Rfam v12.1 (parameters: 1e-5) was used to identify microRNAs and rRNAs, and tRNAscan-SE v1.3.1 (parameters: 1e-5) was used to identify tRNAs". (please see line 27-34 of page 5 and line 1-30 of page 6)

12. - Gene family and phylogenetic analysis :
o Some sentences have to be reworded
Response: Thank you for the valuable comments and suggestions. The sentences of Gene family and phylogenetic analysis have been reworded. (please see line 32-34 of page 6 and line 1-26 of page 7)

13. - Genome assembly and assembled completeness evaluation
o "assembled" could be removed from this title
o The third first sentences should be reworded.
o Table 1: I'm not sure that the authors have to mention "three-generation" each time if not they have to put "third generation". The names of the lanes could be more precise and the caption could be removed. I don't understand the meaning of the sentence "Contig length means the length of Contig in the middle of more than 1Kb of scaffolding".
o The last sentence could be rephrased.
Response: Thank you. "Genome assembly and assembled completeness evaluation" has been revised as "Genome assembly and completeness evaluation". The third first sentences and the last sentence have been rephrased. Table 1 and Table 2 have been rearranged to make the data easier to read, "Nanopore three-generation sequencing results" has been revised as "Nanopore", the names of the lanes have been

revised to be more precise and useless captions have been removed. "Contig length means the length of Contig in the middle of more than 1Kb of scaffolding" has been revised as "the length of contig in the scaffold, and scaffold length exceeds 1 Kb". (please see line 8-26 of page 8, Table1 and Table 2)

14. - Figure 3: the title should include Hi-C. "The Y axis represents the number of reads" could replace the last sentence.
Response: Thank you for the valuable comments and suggestions. The figure tittle has been revised as “The analysis of Hi-C library construction and heat map”, and "The vertical coordinate is the number of random extracted 100,000 pairs of Reads corresponding to the insert fragment of different length" has been revised as "The Y axis represents the number of reads". (please see Fig. 5)

15. - Hybrid assembly, scaffolding and chromosome anchoring:
o the writing of this paragraph should be improved. For example, "236,274,160 Mb pairs were uniquely correlated to the genome, including 56,964,635 pairs valid Hi-C data" could be rephrased as: "236,274,160 paired reads were uniquely mapped on the genome assembly, including 56,964,635 valid Hi-C paired reads"
Response: Thank you. The writing of this paragraph has been revised, and "236,274,160 Mb pairs were uniquely correlated to the genome, including 56,964,635 pairs valid Hi-C data" has been replaced as "236,274,160 paired reads were uniquely mapped on the genome assembly, including 56,964,635 valid Hi-C paired reads". (please see line 2-4 of page 9)

o Table 3: the caption brings nothing. Rather, it should explain what each class of reads represents.
Response: We thank the reviewer for the valuable comments and suggestions. The caption has been revised as "The Hi-C sequencing data types and proportion of A. nanchuanensis", and each class of reads have been represented in the note of Supplementary Table 2. (please see Supplementary Table 2)

o "manually adjusted" could be replaced by "manual adjustment"
Response: Thank you, "manually adjusted" has been replaced by "manual adjustment". (please see line 15 of page 9)

o Figure 4: "inside" instead of "inutside", "Each color" instead of "Each coloured"
Response: Thank you very much. The typo along with other mistakes have been revised, "inutside" has been revised as "inside", "Each coloured" has been revised as "Each color". (Please see Fig. 2.)

o Figure 5: "order" instead of "Order"
o Table 5: "correction" instead of "corrected". The sentence "the length of Contig in the middle of the scaffold above 1Kb" should be reworded
Response: Thank you for the valuable suggestions. The typo along with other mistakes have been revised. "Order" has been revised as "order", "corrected" has been revised as "correction", and "the length of Contig in the middle of the scaffold above 1Kb" has been revised as "length of contig in the scaffold, and scaffold length exceeds 1 Kb". (Please see Fig. 5 and Table 2)

16. - Repeat annotation, gene prediction and gene annotation:
o Table 8: I think that the name of the columns could be more precise in order to remove the caption. "The introns average number in per gene" should be replaced for example by "the average number of intron per gene".
Response: We thank the reviewer for the valuable comments and suggestions, the name of the columns have been revised to be more precise, and the table caption has been removed. (Please see table 5)

o Table 9: the title could be "Functional annotation statistics"
Response: Thank you, the title has been revised as "Functional annotation statistics of A. nanchuanensis". (Please see Supplementary Table 7)

o "The homologous gene of Artocarpus nanchuanensis and morous notabilious was" could be replaced by "The number of homologous genes between A. nanchuanensis and Morous notabilious was"
Response: Thank you. "The homologous gene of Artocarpus nanchuanensis and morous notabilious was" has been replaced by "The number of homologous genes between A. nanchuanensis and Morous notabilious was". (please see line 7-8 of page 10)

o Figure 6: the title should be "distribution of the number of genes among the three methods"
Response: Thanks for the valuable comments and suggestions, the title has been revised as "Distribution of the number of genes among the three methods". (Please see Supplementary Fig. 1)

o Why do you mean by "the three dominant genes"? The three top over expressed genes?
Response: Thanks to reviewers for the valuable comments and suggestions, "the three dominant genes" was replaced as "The top three over expressed genes". (please see line 14 of page 10)

Close
