# Supplementary material for: A chromosome-level genome assembly of Artocarpus nanchuanensis (Moraceae), an extremely endangered fruit tree
Source: Gigascience. 2022 Jun 14;11:giac042. doi: 10.1093/gigascience/giac042 (PMC9197682; doi:10.1093/gigascience/giac042)
Supplement: giac042_Response_to_Reviewer_Comments_Revision_1 [file giac042_response_to_reviewer_comments_revision_1.pdf]

Author's Response To Reviewer Comments

Close

We would like to thank the reviewers very much for taking the time and effort to review our paper so thoroughly. We have carefully considered their comments, which have helped us to make improvements to our work. Our point by point responses to their comments are presented below. The reviewers' comments are italicized and our responses are in red regular font. The changes we made in the text are also highlighted in red.

Reviewer reports:
Reviewer #1:
1. He et al. contributed their revised manuscript of "A chromosome-level genome assembly of Artocarpus nanchuanensis". All my comments and suggestions have been carefully treated. And, it is now ready to be accepted for publication.
Response: Thank you very much.

2. I have one more suggestion on improvement of title. A title with more info may be better. Do you think this my suggested titile would be better? "A chromosome-level genome assembly of Artocarpus nanchuanensis (Moraceae), an extremely endangered fruit tree"
Response: Thanks for your enlightening suggestion, the tittle has been revised as "A chromosome-level genome assembly of Artocarpus nanchuanensis (Moraceae), an extremely endangered fruit tree".

Reviewer #2:
I thank the authors for having taken into account all my remarks and suggestions. The quality of the writing has been greatly improved but there are still some mistakes, especially in the figures and tables.
Response: Thank you very much.

In addition, I still have a few questions.
1. For the RNA extraction, did the authors use a commercial kit? Please add the name of the kit and the manufacturer.
Response: Thank you very much. Yes, we used a commercial kit for RNA extraction, the name of the kit and the manufacturer has been added in the manuscript. (Please see line 3 of page 4)

2. Some quality filtering have been performed on Illumina reads. Please indicate the softwares used (version, parameters...).
Response: Thank you very much. Based on the valuable suggestions from reviewer, the softwares used (version, parameters...) for quality filtering of Illumina reads have been added in the manuscript. (Please see line 25-31 of page 4)

3. In the sentence "the next-generation sequencing data were used to conduct three rounds of calibration by Racon v1.4.21 (default parameters) and Pilon v1.22", I think that "calibration" should be replaced by "correction".
Response: Thank you very much. "calibration" in "the next-generation sequencing data were used to conduct three rounds of calibration by Racon v1.4.21 (default parameters) and Pilon v1.22" has been replaced by "correction". (Please see line 8 of page 5)

4. In the paragraph Genome annotation analysis, I still don't really understand the sentence" PASA v2.0.2 was used to predict unigene sequences based on the transcriptome data of a nonreference assembly". What the authors mean by "nonreference assembly".
Response: Thank you. "nonreference assembly" means the assembly data of nonparametric transcriptome. "PASA v2.0.2 was used to predict unigene sequences based on the transcriptome data of a nonreference assembly" has been revised as "PASA v2.0.2 was used to predict unigene sequences based on the assembly data of nonparametric transcriptome". (Please see line 18, 19 of page 6)

5. For the assembly quality assessment, I still suggest running Merqury (https://genomebiology.biomedcentral.com/articles/10.1186/s13059-020-02134-9) which is now

commonly used. In the table 3, the authors compare several genome assembly statistics. I think it is not an assembly quality comparison but only metrics comparison. It is informative but cannot be used as a quality comparison. To compare the quality, it would be necessary to use Merqury or other tool on each assembly.
Response: Thank you for your valuable comments and suggestions. The Merqury has been used to conduct the assembly quality assessment of Artocarpus nanchuanensis, Ficus microcarpa and Morus notabilis, and the details of assembly quality comparison has been summarized and shown in table 3.
We used Merqury to evaluate our Artocarpus nanchuanensis genome as the reviewer advised and found that the value of qv and completeness are 27.6449 and 68.8838%. While, the ngs reads of our Artocarpus nanchuanensis genome were mapped onto our genome using bwa, 99.41% of reads were mapped and the reads that are properly paired are 93.56%. It indicated that if we ignore some mismatches and gaps between reads and genome, almost all of reads could be mapped onto our genome.
The value of completeness is relatively low comparing with 95.2558% and 79.0855% of Morus notabilis and Ficus microcarpa. We study the algorithm of Merqury and found the completeness is: It counts how many solid k-mers of reads occurring in the genome. This algorithm required that the complete same k-mers must exist in the reads and the genome at the same time. Therefore, we could notice that the completeness of Morus notabilis is the highest because it's assembled with NGS reads totally. The accuracy of read bases of NGS reads is higher than that of Pacbio/Nanopore long reads. And the completeness of Ficus microcarpa and our genome is not so high, because they were assembled with Pacbio and Nanopore long reads respectively. The ngs reads of our genome were mapped onto our genome using bwa, 99.41% of reads were mapped and the reads that are properly paired are 93.56%. It indicated that if we ignore some mismatches and gaps between reads and genome, almost all of reads could be mapped onto our genome.
Ficus microcarpa was reported to have 13 pseudochromosomes and our genome are 28 pseudochromosomes. It indicated that another WGS (whole genome duplication) seems to occur in A. nanchuanensis, which produces lots of similar but not identical sequences. And only one copy of some of these similar sequences was assembled into our genome because the assembler tools could seperate these highly similar sequences. Therefore, as we evaluated our genome with Merqury using ngs reads, Merqury couldn't find out the totally same sequences in our genome for k-mers in ngs reads, then Merqury reported a relative low completeness.

6. In the paragraph "Hybrid assembly, scaffolding, and chromosome anchoring", the sentence "the purification of magnetic beads during library construction functioned efficiency" should be replaced by "the purification of magnetic beads during library construction worked efficiently".
Response: Thank you. "the purification of magnetic beads during library construction functioned efficiency" in the "Hybrid assembly, scaffolding, and chromosome anchoring" has been revised as "the purification of magnetic beads during library construction worked efficiently". (Please see line 7 of page 9)

7. In the paragraph "Gene prediction and annotation", in the sentence "the gene number of NBS-LRR in papaya, watermelon, Arabidopsis, grape, tomato, and notabilis were 55, 44, 166, 251, and 142 respectively, while A. nanchuanensis is 1050,52", It miss one value and the value for A. nanchuanensis looks strange.
Response: Thank you. Based on the common domains of R genes, the number of NBS-LRR in A. nanchuanensis has been corrected as 316, and "the gene number of NBS-LRR in papaya, watermelon, Arabidopsis, grape, tomato, and notabilis were 55, 44, 166, 251, and 142 respectively, while A. nanchuanensis is 1050,52" has been revised as "the gene number of NBS-LRR in papaya, watermelon, arabidopsis, grape, tomato, and notabilis were 55, 44, 166, 504, 251, and 142 respectively, while A. nanchuanensis was 316. (Please see line 33, 34 of page 10)

8. In the title "Table 4 The Hi-C assembly datastatistics table of Artocarpus nanchuanensis.", "data" should be removed.
Response: Thank you very much. Sorry for my typo mistake, the title of Table 4 has been revised as "The Hi-C assembly statistics table of A. nanchuanensis". (Please see Table 4)

9. In the table 7, "alignmented" should be replaced by "aligned".
Response: Thank you very much. "alignmented" in the table 7 has be revised as "aligned". (Please see Table 7)

10. In the Fig 2, "The picture of the A. nanchuanensis tree study used in this study" should be replaced

by "The picture of the A. nanchuanensis tree used in this study".
Response: Thank you very much. "The picture of the A. nanchuanensis tree study used in this study" in the Fig 2 has been revised as "The picture of the A. nanchuanensis tree used in this study". (Please see Fig. 2)

11. In the Fig 2, the two circos plots are very small and it is difficult to read the chromosome names.
Response: Thank you very much. The circle plots have been replaced with a higher quality figure based on your valuable suggestions. (Please see Fig. 2)

12. I suggest to move figures 3 and 4 to supp data.
Response: Thank you for your valuable comments and suggestions, the figures 3 and 4 have been moved to supp data. (Please see Supplementary Fig. 1 and Fig. 2)

13. In the figure 5a, the legends are very small too. I should replace "a means" by "a shows".
Response: Thank you for your valuable suggestion. The legends of figure have been replaced by a bigger one to make it clearer to read and "means" in the figure has been revised as "shows". (Please see Fig. 3)

14. In the figure 7, "ananlysis" should be replaced by "analysis".
Response: Thank you. The typo along with other mistakes have been revised.
"ananlysis" in the figure 7 has been replaced as "analysis". (Please see Fig. 5)

Close
