# Supplementary material for: A chromosome-level genome assembly of Artocarpus nanchuanensis (Moraceae), an extremely endangered fruit tree
Source: Gigascience. 2022 Jun 14;11:giac042. doi: 10.1093/gigascience/giac042 (PMC9197682; doi:10.1093/gigascience/giac042)
Supplement: giac042_Reviewer_1_Report_Original_Submission — Jian-Feng Mao, Ph.D. -- 7/11/2021 Reviewed [file giac042_reviewer_1_report_original_submission.pdf]

Reviewer Report

Title: A chromosome-level genome assembly of Artocarpus nanchuanensis (Moraceae), an extremely endangered fruit tree

Version: Original Submission **Date:** 7/11/2021

Reviewer name: Jian-Feng Mao, Ph.D.

Reviewer Comments to Author:

He et al. contributed their study of "A chromosome-level genome assembly of Artocarpus nanchuanensis". This study present valuable and high quality genome resources for important plant group. The study has significant values for publication. While, problems remain especially in English expression and writing.

I will recommend to accept it after a major revision.

Major comments:

1. There is significantly large space for the English expression to be improved. Some sentences were not well developed, and some others were wrong in expression.

As an example, can you pick the error in the sentence in line 28? Do you think "persistent" was right here? And I think there is better way to restructure this sentence.

Another example, lines 33 page 1 - line 1 page 2 in the main text, after reading this sentence, I am confused. I would like to know who do include 78.34 billion high-quality bases. It is so huge to have such large number of bases for a genome assembly. It should be the data generated for genome assembly. But, the sentence is truly problematic in expression. Please take care of writing, as it is very important.

2. line numbers are not consistently put, and no page number. It is very hard to put comments.
3. Other than English expression, the quality of figure and table need to be significantly improved.
4. Detailed discription is needed for some specific analyses, such us 4DTV, LTR insertion time. Why did you do these analyses? what did you find? How you results could be compared to others? What is new findings, what is the differences and the commons?
5. A general discussion or comparsion to related results previously published is needed for many parts, like quality and characterization of genome assembly, phylogeny, gene families in expansion and contraction, divergent time, 4DTV (if whole genome duplication is an objective of the authors), LTR insertion time.
6. Conclusion should not be the only summary of results. You should point out key points/values for future/related fields. And most probably, the significance and implications of this study should be provided. Or any shortage/limiation you could see.
7. No problem found for open data sharing, by checking the liked data the authors provided.

Minor ones:

1. line 24, do you think the reference No 12 is refered here? or just the No 1 and No 2?
2. On Latin names. please change such "A.nanchuanensis" expression to "A. nanchuanensis". This means you may need to add a space after ".".
3. lines 21-24, A.nanchuanensis -> Artocarpus nanchuanensis

line 2, Artocarpus.nanchuanensis -> Artocarpus nanchuanensis
4. line 32, "Moraceae Mulberry and Paper Mulberry", do not put them in Italic, if they are not Latin names.
5. last paragraph in Introduction, the sentence is confusing. One confusiong woule be I do not know who could provide the necessary resources for the genome size selection. And do you think genome size selection is important? And many more confusions here. Please improve the expression.
6. Version information should be provided for softwares used in analyses.
7. many typos in references list, such as "k - - â€� mer", no publication year provided,
8. I do not know the requirment from GigaScience on the number of main figures and tables. But, it seems they are too many now in the manuscipt. Please do a reasonable re-examination, and then put some of the figures and tables to the supplementary, such as figure 1. And you may need to merge some of them into a compact figure or tables, such as figre 2 and figure 4. Also, figures (11, 12, 13) need to be improved, they are ugly and it is not hard for improvement.
9. I may be interested to see a talbe comparing the quality/properties of genomes assemblies released for any other Moraceae and the currently reported one, on parameters like sequencing tech and depth, Contig/Scaffold N50, N90, annotated genes, repeat composion.
10. Other than Table 2 & 3, it would be interesting to present mapping rate and genome coverage (like 10 fold coverage) of those ONT and Illumina data used for assembly.
11. When the plant Latin names shown up for the first time, you may need to show the full names. And the right citations should be provided if genomic data were used in this study, such as the genomic data for A.thaliana, A.trichopoda, P.trichocarpa, A.chinensis, V.vinifera, M.notabilis, T.cacao.
12. I am wondering some symbols are in format of not a routinely English format (like ï¼› in Section 2.4 line 34).
13. Section 3.5 Comparative genomics line 23, "expanding and contracting gene families" is not good expression. It may be better to be changed to "gene families in contraction and expansion". There are many such bugs in expression across the whole paper. Please check related paper for improvement in English expression.

Methods

Are the methods appropriate to the aims of the study, are they well described, and are necessary controls included? Choose an item.

Conclusions

Are the conclusions adequately supported by the data shown? Choose an item.

Reporting Standards

Does the manuscript adhere to the journal’s guidelines on minimum standards of reporting? Choose an item.

Choose an item.

Statistics

Are you able to assess all statistics in the manuscript, including the appropriateness of statistical tests used? Choose an item.

Quality of Written English

Please indicate the quality of language in the manuscript: Choose an item.

Declaration of Competing Interests

Please complete a declaration of competing interests, considering the following questions:

- Have you in the past five years received reimbursements, fees, funding, or salary from an organisation that may in any way gain or lose financially from the publication of this manuscript, either now or in the future?
- Do you hold any stocks or shares in an organisation that may in any way gain or lose financially from the publication of this manuscript, either now or in the future?
- Do you hold or are you currently applying for any patents relating to the content of the manuscript?
- Have you received reimbursements, fees, funding, or salary from an organization that holds or has applied for patents relating to the content of the manuscript?
- Do you have any other financial competing interests?
- Do you have any non-financial competing interests in relation to this paper?

If you can answer no to all of the above, write 'I declare that I have no competing interests' below. If your reply is yes to any, please give details below.

I declare that I have no competing interests

I agree to the open peer review policy of the journal. I understand that my name will be included on my report to the authors and, if the manuscript is accepted for publication, my named report including any attachments I upload will be posted on the website along with the authors' responses. I agree for my report to be made available under an Open Access Creative Commons CC-BY license (http://creativecommons.org/licenses/by/4.0/). I understand that any comments which I do not wish to be included in my named report can be included as confidential comments to the editors, which will not be published.

Choose an item.

To further support our reviewers, we have joined with Publons, where you can gain additional credit to further highlight your hard work (see: https://publons.com/journal/530/gigascience). On publication of this paper, your review will be automatically added to Publons, you can then choose whether or not to claim your Publons credit. I understand this statement.

Yes Choose an item.
