# Supplementary material for: A chromosome-level genome assembly of Artocarpus nanchuanensis (Moraceae), an extremely endangered fruit tree
Source: Gigascience. 2022 Jun 14;11:giac042. doi: 10.1093/gigascience/giac042 (PMC9197682; doi:10.1093/gigascience/giac042)
Supplement: giac042_Reviewer_2_Report_Original_Submission — Caroline Belser -- 7/26/2021 Reviewed [file giac042_reviewer_2_report_original_submission.pdf]

Reviewer Report

Title: A chromosome-level genome assembly of Artocarpus nanchuanensis (Moraceae), an extremely endangered fruit tree

Version: Original Submission **Date:** 7/26/2021

Reviewer name: Caroline Belser

Reviewer Comments to Author:

This article is presenting the genome assembly of Artocarpus nanchuanensis. This work could be of great interest because of its endangered status and its nutritional and medicinal value.
The results seem to be of quality but the manuscript has to be improved. The methods seem to be well used. The major concern is that the reading is difficult because the sentences are often too long and contain many writing errors. In addition, the methods lack detailed information.
Here is a list of remarks, questions and comments. This is not an exhaustive list of all the corrections to be made to the manuscript.
- Abstract Line 5: it could be more suitable to cite the methods as follow: whole genome sequencing using Illumina and Oxford Nanopore Technology platforms and chromosomal conformation capture technique
- Abstract Line 6: "Nanopore Sequel reads" should be replaced by "Nanopore reads"
- Abstract Line 12: "genome assembly integrity" should be replaced by "genome assembly completeness".
- Introduction Lines 12-13: sentence has to be improved. It is not appropriate to write that the genomes "have been made in detail". The two following sentences have to be improved too.
- Introduction Lines 21-28. Too long sentence. I'm not sure that "high-pass" is the good term.
- Figure 1:
o Chromatin instead of "Cromatin"
o "Correction" instead of "Crrection"
o "Genome" instead of "Gemone"
o "polishing" instead of "polish"
o What is "Genome evalue"?
o This figure presents a flow chart. The HiC part should be placed after the nanopore part
o Hi-C instead of "Hic"
- Sample and DNA extraction:
o "The samples of genome were young leaves" could be replaced by "For genome sequencing, DNA was extracted from young leaves" for example.
o What is the quantity of young leaves used in the CTAB extraction process?
o Can you more detail this process or provide a reference?
o "for transcriptome analysis" could be replaced by "for RNA extraction"
o "ONT Library with 20Kb fragment length was constructed following the manufacturer's protocol"
o the rest of the paragraph should be reworded.

o Integrity of DNA was "checked on" pulsed field electrophoresis.
o R9 flow cell instead of "R9 cell"
o Preparation instead of "prep"
o How do you realize the genome size estimation? Do you estimate the heterozygosity rate?
o How do you perform the read cleaning? Softwares?
o Figure 2: caption is missing.
- There is no detail on the RNA extraction (quantity engaged in the extraction, protocol used) and on the sequencing library preparation.
- Genome assembly and quality assessment:
o "three rounds calibration by racon and Pilon", did you mean assembly correction?
o "BWA software was used to align short sequences on the reference genome" could be better than this long sentence.
o BUSCO is used to evaluate the completeness of the assembly
- In general, there is a lack of validation of the assembly. Can you add some evaluations such as KAT histogramms which reflect the completeness of the assembly process (https://kat.readthedocs.io/en/latest/) and use Merqury to evaluate the quality of the assembly? (https://genomebiology.biomedcentral.com/articles/10.1186/s13059-020-02134-9).
- Genome annotation analysis:
o this section contains very long sentences.
o "based on transcriptome data unreferenced assembly": I'm not sure to understand what authors mean.
o "parameter for blast: The e-value" please clarify
o Lines 28-31: please correct the sentence.
- Gene family and phylogenetic analysis :
o Some sentences have to be reworded
- Genome assembly and assembled completeness evaluation
o "assembled" could be removed from this title
o The third first sentences should be reworded.
o Table 1: I'm not sure that the authors have to mention "three-generation" each time if not they have to put "third generation". The names of the lanes could be more precise and the caption could be removed. I don't understand the meaning of the sentence "Contig length means the length of Contig in the middle of more than 1Kb of scaffolding".
o The last sentence could be rephrased.
- Figure 3: the title should include Hi-C. "The Y axis represents the number of reads" could replace the last sentence.
- Hybrid assembly, scaffolding and chromosome anchoring:
o the writing of this paragraph should be improved. For example, "236,274,160 Mb pairs were uniquely correlated to the genome, including 56,964,635 pairs valid Hi-C data" could be rephrased as: "236,274,160 paired reads were uniquely mapped on the genome assembly, including 56,964,635 valid Hi-C paired reads".
o Table 3: the caption brings nothing. Rather, it should explain what each class of reads represents.
o "manually adjusted" could be replaced by "manual adjustment"

o Figure 4: "inside" instead of "inutside", "Each color" instead of "Each coloured"
o Figure 5: "order" instead of "Order"
o Table 5: "correction" instead of "corrected". The sentence "the length of Contig in the middle of the scaffold above 1Kb" should be reworded
- Repeat annotation, gene prediction and gene annotation:
o Table 8: I think that the name of the columns could be more precise in order to remove the caption. "The introns average number in per gene" should be replaced for example by "the average number of intron per gene".
o Table 9: the title could be "Functional annotation statistics"
o "The homologous gene of Artocarpus nanchuanensis and morous notabilious was" could be replaced by "The number of homologous genes between A. nanchuanensis and Morous notabilious was"
o Figure 6: the title should be "distribution of the number of genes among the three methods"
o Why do you mean by "the three dominant genes"? The three top over expressed genes?

Methods

Are the methods appropriate to the aims of the study, are they well described, and are necessary controls included? Choose an item.

Conclusions

Are the conclusions adequately supported by the data shown? Choose an item.

Reporting Standards

Does the manuscript adhere to the journal's guidelines on minimum standards of reporting? Choose an item.

Choose an item.

Statistics

Are you able to assess all statistics in the manuscript, including the appropriateness of statistical tests used? Choose an item.

Quality of Written English

Please indicate the quality of language in the manuscript: Choose an item.

Declaration of Competing Interests

Please complete a declaration of competing interests, considering the following questions:

- Have you in the past five years received reimbursements, fees, funding, or salary from an organisation that may in any way gain or lose financially from the publication of this manuscript, either now or in the future?
- Do you hold any stocks or shares in an organisation that may in any way gain or lose financially from the publication of this manuscript, either now or in the future?
- Do you hold or are you currently applying for any patents relating to the content of the manuscript?
- Have you received reimbursements, fees, funding, or salary from an organization that holds or has applied for patents relating to the content of the manuscript?
- Do you have any other financial competing interests?
- Do you have any non-financial competing interests in relation to this paper?

If you can answer no to all of the above, write 'I declare that I have no competing interests' below. If your reply is yes to any, please give details below.

I declare that I have no competing interests

I agree to the open peer review policy of the journal. I understand that my name will be included on my report to the authors and, if the manuscript is accepted for publication, my named report including any attachments I upload will be posted on the website along with the authors' responses. I agree for my report to be made available under an Open Access Creative Commons CC-BY license (http://creativecommons.org/licenses/by/4.0/). I understand that any comments which I do not wish to be included in my named report can be included as confidential comments to the editors, which will not be published.

Choose an item.

To further support our reviewers, we have joined with Publons, where you can gain additional credit to further highlight your hard work (see: https://publons.com/journal/530/gigascience). On publication of this paper, your review will be automatically added to Publons, you can then choose whether or not to claim your Publons credit. I understand this statement.

Yes Choose an item.
