# Supplementary material for: A chromosome-level genome assembly of Artocarpus nanchuanensis (Moraceae), an extremely endangered fruit tree
Source: Gigascience. 2022 Jun 14;11:giac042. doi: 10.1093/gigascience/giac042 (PMC9197682; doi:10.1093/gigascience/giac042)
Supplement: giac042_Reviewer_2_Report_Revision_1 — Caroline Belser -- 11/8/2021 Reviewed [file giac042_reviewer_2_report_revision_1.pdf]

Reviewer Report

Title: A chromosome-level genome assembly of Artocarpus nanchuanensis (Moraceae), an extremely endangered fruit tree

Version: Revision 1 **Date:** 11/8/2021

Reviewer name: Caroline Belser

Reviewer Comments to Author:

I thank the authors for having taken into account all my remarks and suggestions. The quality of the writing has been greatly improved but there are still some mistakes, especially in the figures and tables. In addition, I still have a few questions.
For the RNA extraction, did the authors use a commercial kit? Please add the name of the kit and the manufacturer.
Some quality filtering have been performed on Illumina reads. Please indicate the softwares used (version, parameters...).
In the sentence "the next-generation sequencing data were used to conduct three rounds of calibration by Racon v1.4.21 (default parameters) and Pilon v1.22", I think that "calibration" should be replaced by "correction".
In the paragraph Genome annotation analysis, I still don't really understand the sentence " PASA v2.0.2 was used to predict unigene sequences based on the transcriptome data of a nonreference assembly". What the authors mean by "nonreference assembly".
For the assembly quality assessment, I still suggest running Merqury (https://genomebiology.biomedcentral.com/articles/10.1186/s13059-020-02134-9) which is now commonly used. In the table 3, the authors compare several genome assembly statistics. I think it is not an assembly quality comparison but only metrics comparison. It is informative but cannot be used as a quality comparison. To compare the quality, it would be necessary to use Merqury or other tool on each assembly.
In the paragraph "Hybrid assembly, scaffolding, and chromosome anchoring", the sentence "the purification of magnetic beads during library construction functioned efficiency" should be replaced by "the purification of magnetic beads during library construction worked efficiently".
In the paragraph "Gene prediction and annotation", in the sentence "the gene number of NBS-LRR in papaya, watermelon, Arabidopsis, grape, tomato, and notabilis were 55, 44, 166, 251, and 142respectively, while A. nanchuanensis is 1050,52", It miss one value and the value for A. nanchuanensis looks strange.
In the title "Table 4 The Hi-C assembly datastatistics table of Artocarpus nanchuanensis.", "data" should be removed.
In the table 7, "alignmented" should be replaced by "aligned".
In the Fig 2, "The picture of the A. nanchuanensis tree study used in this study" should be replaced by "The picture of the A. nanchuanensis tree used in this study".
In the Fig 2, the two circos plots are very small and it is difficult to read the chromosome names.

I suggest to move figures 3 and 4 to supp data.
In the figure 5a, the legends are very small too. I should replace "a means" by "a shows".
In the figure 7, "ananlysis" should be replaced by "analysis".

Methods

Are the methods appropriate to the aims of the study, are they well described, and are necessary controls included? Choose an item.

Conclusions

Are the conclusions adequately supported by the data shown? Choose an item.

Reporting Standards

Does the manuscript adhere to the journal's guidelines on minimum standards of reporting? Choose an item.

Choose an item.

Statistics

Are you able to assess all statistics in the manuscript, including the appropriateness of statistical tests used? Choose an item.

Quality of Written English

Please indicate the quality of language in the manuscript: Choose an item.

Declaration of Competing Interests

Please complete a declaration of competing interests, considering the following questions:

- Have you in the past five years received reimbursements, fees, funding, or salary from an organisation that may in any way gain or lose financially from the publication of this manuscript, either now or in the future?
- Do you hold any stocks or shares in an organisation that may in any way gain or lose financially from the publication of this manuscript, either now or in the future?
- Do you hold or are you currently applying for any patents relating to the content of the manuscript?
- Have you received reimbursements, fees, funding, or salary from an organization that holds or has applied for patents relating to the content of the manuscript?
- Do you have any other financial competing interests?
- Do you have any non-financial competing interests in relation to this paper?

If you can answer no to all of the above, write 'I declare that I have no competing interests' below. If your reply is yes to any, please give details below.

I declare that I have no competing interests

I agree to the open peer review policy of the journal. I understand that my name will be included on my report to the authors and, if the manuscript is accepted for publication, my named report including any attachments I upload will be posted on the website along with the authors' responses. I agree for my report to be made available under an Open Access Creative Commons CC-BY license (http://creativecommons.org/licenses/by/4.0/). I understand that any comments which I do not wish to be included in my named report can be included as confidential comments to the editors, which will not be published.

Choose an item.

To further support our reviewers, we have joined with Publons, where you can gain additional credit to further highlight your hard work (see: https://publons.com/journal/530/gigascience). On publication of this paper, your review will be automatically added to Publons, you can then choose whether or not to claim your Publons credit. I understand this statement.

Yes Choose an item.
